# Hidden genomic evolution in a morphospecies—The landscape of rapidly evolving genes in *Tetrahymena*

**DOI:** 10.1371/journal.pbio.3000294

**Published:** 2019-06-03

**Authors:** Jie Xiong, Wentao Yang, Kai Chen, Chuanqi Jiang, Yang Ma, Xiaocui Chai, Guanxiong Yan, Guangying Wang, Dongxia Yuan, Yifan Liu, Shelby L. Bidwell, Nikhat Zafar, Michalis Hadjithomas, Vivek Krishnakumar, Robert S. Coyne, Eduardo Orias, Wei Miao

**Affiliations:** 1 Key Laboratory of Aquatic Biodiversity and Conservation, Institute of Hydrobiology, Chinese Academy of Sciences, Wuhan, China; 2 University of Chinese Academy of Sciences, Beijing, China; 3 Department of Pathology, University of Michigan, Ann Arbor, Michigan, United States of America; 4 J. Craig Venter Institute, Rockville, Maryland, United States of America; 5 Department of Molecular, Cellular, and Developmental Biology, University of California, Santa Barbara, California, United States of America; 6 CAS Center for Excellence in Animal Evolution and Genetics, Kunming, China; 7 State Key Laboratory of Freshwater Ecology and Biotechnology of China, Wuhan, China; Fred Hutchinson Cancer Research Center, UNITED STATES

## Abstract

A morphospecies is defined as a taxonomic species based wholly on morphology, but often morphospecies consist of clusters of cryptic species that can be identified genetically or molecularly. The nature of the evolutionary novelty that accompanies speciation in a morphospecies is an intriguing question. Morphospecies are particularly common among ciliates, a group of unicellular eukaryotes that separates 2 kinds of nuclei—the silenced germline nucleus (micronucleus [MIC]) and the actively expressed somatic nucleus (macronucleus [MAC])—within a common cytoplasm. Because of their very similar morphologies, members of the *Tetrahymena* genus are considered a morphospecies. We explored the hidden genomic evolution within this genus by performing a comprehensive comparative analysis of the somatic genomes of 10 species and the germline genomes of 2 species of *Tetrahymena*. These species show high genetic divergence; phylogenomic analysis suggests that the genus originated about 300 million years ago (Mya). Seven universal protein domains are preferentially included among the species-specific (i.e., the youngest) *Tetrahymena* genes. In particular, leucine-rich repeat (LRR) genes make the largest contribution to the high level of genome divergence of the 10 species. LRR genes can be sorted into 3 different age groups. Parallel evolutionary trajectories have independently occurred among LRR genes in the different *Tetrahymena* species. Thousands of young LRR genes contain tandem arrays of exactly 90-bp exons. The introns separating these exons show a unique, extreme phase 2 bias, suggesting a clonal origin and successive expansions of 90-bp–exon LRR genes. Identifying LRR gene age groups allowed us to document a *Tetrahymena* intron length cycle. The youngest 90-bp exon LRR genes in *T*. *thermophila* are concentrated in pericentromeric and subtelomeric regions of the 5 micronuclear chromosomes, suggesting that these regions act as genome innovation centers. Copies of a *Tetrahymena* Long interspersed element (LINE)-like retrotransposon are very frequently found physically adjacent to 90-bp exon/intron repeat units of the youngest LRR genes. We propose that *Tetrahymena* species have used a massive exon-shuffling mechanism, involving unequal crossing over possibly in concert with retrotransposition, to create the unique 90-bp exon array LRR genes.

## Abstract 2

Genomic comparison of ten morphologically very similar species of ciliate from the genus Tetrahymena reveals how parallel microevolutionary processes have shaped their genomes and created unique genes through retrotransposition.

## Introduction

The species is one of the fundamental units of biology. Defining a species has been a controversial issue for more than half a century, and there are more than 20 species concepts [1]. One commonly used method of species definition by taxonomists is the morphospecies concept [1]. The term morphospecies is applied to a taxonomic group containing multiple cryptic biological species that are sexually isolated but morphologically indistinguishable or very similar [2–4]. Morphospecies are quite common and widespread in eukaryotes, from unicellular protists to mammals [2, 5–7], and yet the evolutionary novelty hidden in morphospecies remains poorly investigated. Ciliates are a group of protists in which morphospecies are particularly common; thousands of free-living ciliate morphospecies are estimated to exist [8, 9]. The ciliate genus *Tetrahymena* is composed of more than 40 currently described species [10]. Historically, a large group of morphologically very similar *Tetrahymena* species were referred to as *T*. *pyriformis* [9]. Following the discovery of mating types in *Tetrahymena*, it was recognized that *T*. *pyriformis* is a morphospecies consisting of many reproductively isolated species and some asexual species [11, 12]. These species cannot be distinguished by ultrastructural morphology (such as cortical features revealed using protargol staining or electron microscopy), the number and size of micronuclear chromosomes, or tolerance to metals and high temperature [12]. Comparison of the ribosomal DNA (rDNA) sequences of these species suggested that they have high genetic divergence [9] and that the *Tetrahymena* genus is a few hundred million years old [13].

Like ciliates in general, *Tetrahymena* separates its germline and somatic genetic information by maintaining 2 functionally distinct nuclei: the silent, diploid micronucleus (MIC) or germline nucleus, and the highly expressed, polyploid macronucleus (MAC) or somatic nucleus [8, 14]. The ciliate life cycle includes a sexual stage (conjugation) in which the conjugants undergo meiosis of the diploid MIC and exchange haploid gametic pronuclei, which fuse and generate a diploid fertilization nucleus in each exconjugant [15]. The new diploid MIC and polyploid MAC of the sexual progeny differentiate from mitotic siblings of the diploid fertilization nucleus, and the parental MAC is destroyed [15]. During MAC differentiation, transposable elements (TEs) and other repeated sequences inherited from the MIC are excised as Internal Eliminated Sequences (IESs) [16, 17]. The MAC chromosomes are formed by joining the remaining MAC-destined sequences (MDSs) [16, 17]. All gene expression during the vegetative phase of the life cycle occurs in the MAC [16, 17]. Thus, from a genetic and evolutionary standpoint, ciliates are comparable to multicellular eukaryotes that separate germline and somatic cell lineages [18].

We were intrigued by the nature of the evolutionary novelty that has led to prolific speciation within this genus while retaining morphological similarity over a few hundred million years. The MAC and MIC genomes of the model species *T*. *thermophila* were sequenced, assembled, and annotated previously [19, 20]. We report here the sequencing and annotation of 9 additional *Tetrahymena* MAC genomes widely spanning the phylogeny of the genus. The sequences of the most conserved genes across the genus have allowed us to robustly confirm the ancient origins of this morphospecies. We sought to understand what genes have changed in these species as they diverged from one another, i.e., the source of evolutionary novelty. To this end, we identified and characterized the youngest, species-specific genes of all 10 species. By far the most common domain found among these youngest genes is the leucine-rich repeat (LRR). Our evidence indicates that widespread exon shuffling is responsible for the evolutionary diversification of *Tetrahymena* LRR genes. This shuffling has generated nearly 50,000 exons that are exactly 90 bp in length, more than 96% of which are separated by phase 2 introns. We also provide evidence that ectopic recombination, possibly in concert with retrotransposition mediated by a Long interspersed element (LINE)-like retrotransposon, have been major factors in the extensive shuffling of the exactly 90-bp *Tetrahymena* LRR gene exons.

## Results

### The morphologically uniform genus *Tetrahymena* originated more than 300 million years ago

**Fig 1:**
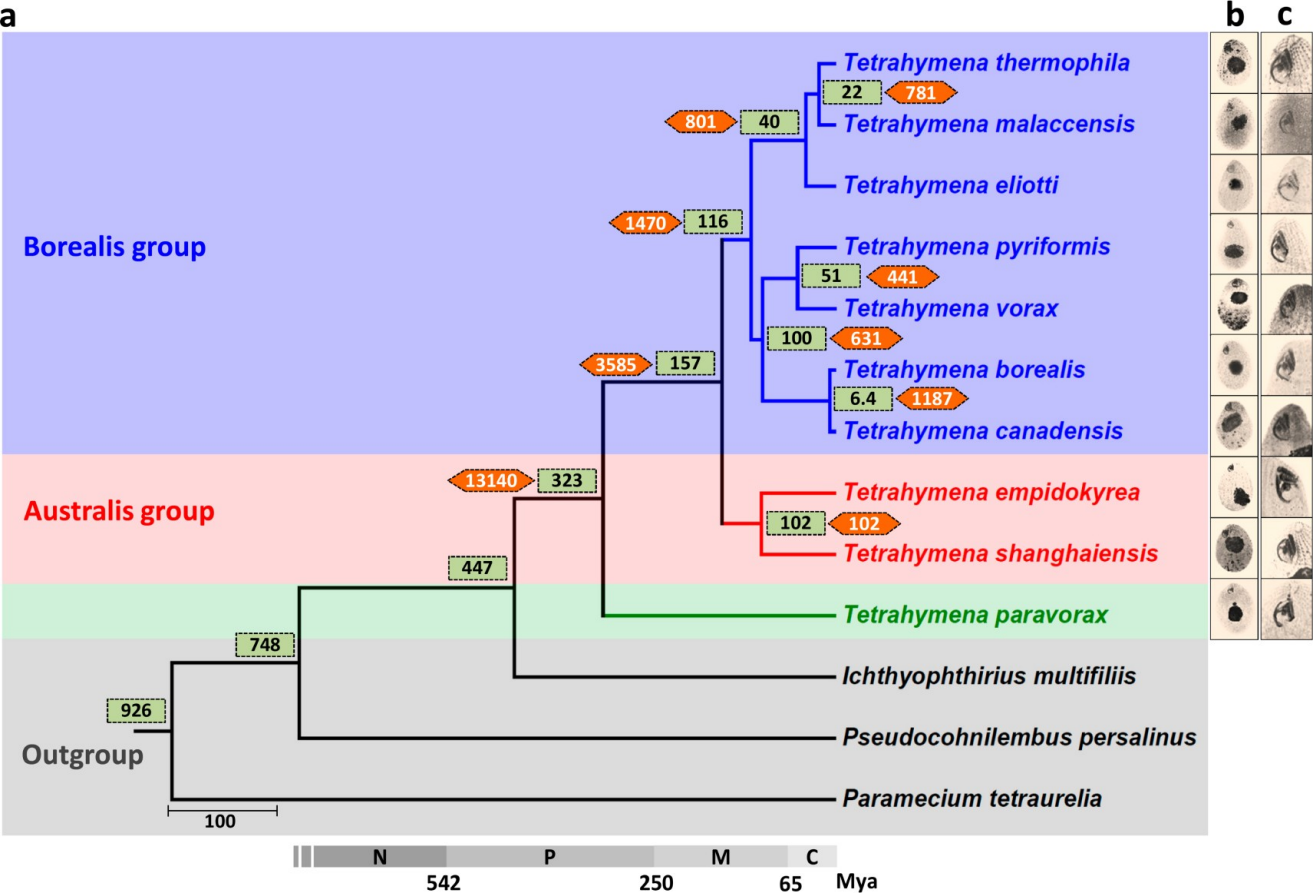
Phylogenetic tree and estimated divergence times of 10 morphologically similar *Tetrahymena* species. (a) Maximum likelihood species tree, using 198 one-to-one orthologs, 104,434 amino acid sites, and 1,000 bootstraps. Green boxes, estimated divergence times for each node; orange hexagons, number of shared ortholog groups for each node; gray bar, geologic timescale. (b) Overall cell morphology and (c) oral apparatus, as revealed by silver-staining. C, Cenozoic; M, Mesozoic; Mya, million years ago; N, Neoproterozoic; P, Paleozoic.

Ten species of the *Tetrahymena* genus were selected for comparative analysis: *T*. *thermophila* (the only species with previously published MAC and MIC genomes), *T*. *malaccensis*, *T*. *elliotti*, *T*. *pyriformis*, *T*. *vorax*, *T*. *borealis*, *T*. *canadensis*, *T*. *empidokyrea* (a mosquito parasite), *T*. *shanghaiensis*, and *T*. *paravorax*. These species are barely distinguishable at the morphological level (Fig 1 and S1 Table) [12]. The MAC genomes of these species were sequenced and assembled (Table 1). Scaffold N50 values were comparable to those previously reported for the *T*. *thermophila* MAC genome [19]. In contrast to their similar morphological characteristics, a high level of genetic divergence was found among the 10 species, with genome sizes varying within a 1.4-fold range (84.9–114.8 Mb; Table 1), Guanine and Cytosine (GC) content varying from 20% to 29% (Table 1), and weak sequence similarity among the 10 genomes (S1 Fig).

**Table 1:** Comparison of statistical data on the genomes of 10 *Tetrahymena* species.

Species	MAC Genome Size (Mb)	N50 (MAC) (Kb)	Number of Scaffolds (MAC)	GC Content (%)	Number of Genes	MIC Genome Size (Mb)	N50 (MIC) (Kb)
*T*. *thermophila*^1^	103.0	637	1,158	22.3	26,996	157.7	487
*T*. *malaccensis*^2^	106.7	496	554	22	24,866	140.3	65
*T*. *elliotti*	90.8	711	331	22.4	22,925	NA	NA
*T*. *pyriformis*	116.1	603	367	28	26,866	NA	NA
*T*. *vorax*	114.8	658	512	28.3	25,238	NA	NA
*T*. *borealis*	93.5	639	325	23.6	20,694	NA	NA
*T*. *canadensis*	103.4	416	1,637	23.9	25,188	NA	NA
*T*. *empidokyrea*	84.9	447	558	25.6	20,847	NA	NA
*T*. *shanghaiensis*	95.6	154	2,660	20.1	21,982	NA	NA
*T*. *paravorax*	108.4	574	448	28.4	25,551	NA	NA

^**1**^MAC and MIC genomes of *T*. *thermophila* are previously published data (TGD Wiki, http://ciliate.org). All other genomes are newly sequenced.

^**2**^Draft MIC genome of *T*. *malaccensis* was sequenced by Pacbio SMRT sequencing technology; the N50 value refers to contig length.

**Abbreviations:** GC, Guanine and Cytosine; MAC, macronucleus; MIC, micronucleus; N50, the sequence length of the shortest contig at 50% of the total genome length; NA, not available; SMRT, single-molecule real-time; TGD, *Tetrahymena* Genome Database.

By integrating de novo and homology-based gene prediction methods as well as RNA sequencing (RNA-Seq) data, protein-coding genes were modeled in all 9 newly sequenced *Tetrahymena* species (see Table 1, which also includes previously available *T*. *thermophila* data). A species tree was inferred using phylogenomic analysis of 198 one-to-one ortholog genes identified in the 10 *Tetrahymena* species and in 5 other ciliate species used as outgroups: *Ichthyophthirius multifiliis*, *Pseudocohnilembus persalinus*, *Paramecium tetraurelia*, *Oxytricha trifallax*, and *Stylonychia lemnae* (Fig 1A). Molecular clock estimates of divergence times suggest that the *Tetrahymena* genus originated more than 300 million years ago (Mya) (Fig 1A), similar to previous estimates based on the rDNA sequences [13, 21]. This origin time is comparable to that of mammals and is far earlier than that of the *Drosophila* genus, approximately 40 Mya [22]. These 9 whole MAC genome assemblies and annotations will be useful for many comparative genomic purposes and have been made available at the *Tetrahymena* Comparative Genomics Database (TCGD; http://ciliate.ihb.ac.cn).

### The distribution of species-specific genes suggests that the pericentromeric and subtelomeric regions of MIC chromosomes are the main centers of genome innovation in *T*. *thermophila*

**Fig 2:**
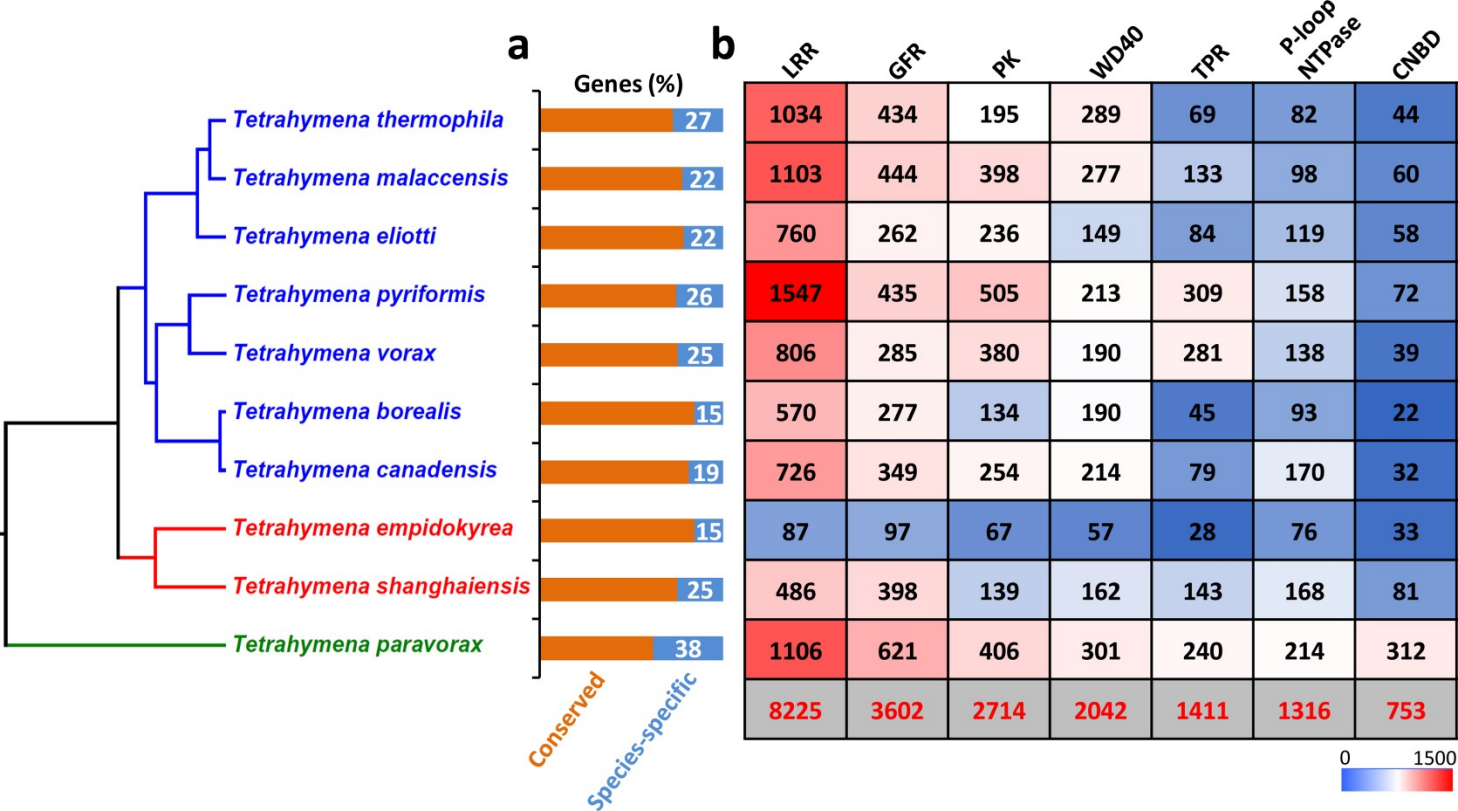
Top gene domains that contribute to the high MAC genome divergence in the 10 species. (a) Percentage of species-specific genes in each species. (b) Heat map of the top 7 categories of domains found in species-specific genes: the bottom row (gray background) shows the total number of genes containing each domain category in all 10 species. CNBD, cyclic nucleotide-binding; GFR, growth factor receptor cysteine-rich; LRR, leucine-rich repeat; MAC, macronucleus; P-loop NTPase, P-loop-containing nucleoside triphosphate hydrolase; PK, protein kinase; TPR, tetratricopeptide repeat; WD40, WD40 repeat.

Whole-genome comparative analysis was performed to cluster genes into ortholog groups using OrthoMCL [23]. A total of 24,486 gene clusters (ortholog groups) were identified and assigned to 10 categories (I–X) based on the number of *Tetrahymena* species (1 to 10) represented in each cluster (S2A Fig and S1 Data). A total of 8,445 ortholog groups were found containing member(s) in all 10 species (category X), including 6,052 one-to-one orthologs. Based on ortholog clustering, all predicted protein-coding genes in each species were divided into 2 groups: conserved genes (represented in at least 2 species) and “species-specific” genes (represented in only 1 species in our current sample). Proportions of species-specific genes range from 15% to 38% among the 10 species (Fig 2A). Different species seem to have independently undergone similar genome innovation processes, because the top protein domains encoded by species-specific genes are very similar for every species, mainly consisting of the following 7 domains: LRR, tetratricopeptide repeat (TPR), WD40 repeat, protein kinase (PK), cyclic nucleotide-binding domain (CNBD), growth factor receptor (GFR) cysteine-rich domain, and P-loop-containing nucleoside triphosphate hydrolase (P-loop NTPase) (Fig 2B and S2B Fig). These 7 protein domains are essentially universal, occurring widely in both eukaryotes and prokaryotes.

**Fig 3:**
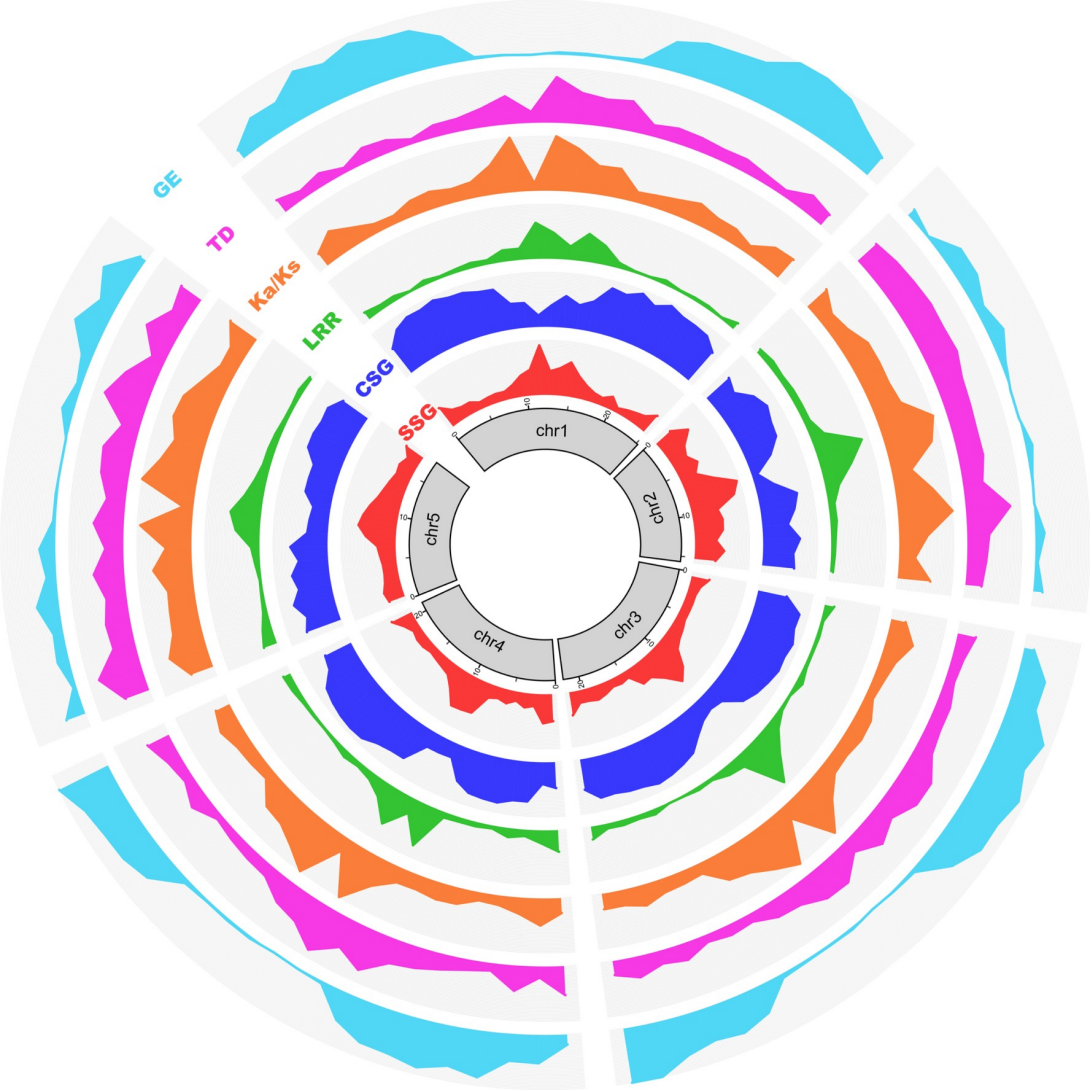
Pericentromeric and subtelomeric regions of MIC chromosomes are gene innovation centers. Circos (http://circos.ca/) diagram mapping the frequency of various properties associated with rapidly evolving genes to the 5 chromosomes of the *T*. *thermophila* MIC genome. Chromosomes (after omitting IESs) were divided into approximately 1 Mb bins. Values were normalized for the total number of genes and plotted for each bin. SSG indicates the density distribution of all species-specific genes; y-axis is the number of genes. CSG indicates the density distribution of the most highly conserved genes (i.e., ortholog category X); y-axis is the number of genes. LRR indicates the density distribution of species-specific LRR genes; y-axis is the number of genes. Ka/Ks indicates the distribution of Ka/Ks ratios, plotted as median value of each bin. TD indicates the distribution of tandem gene duplication frequencies; y-axis is the percentage of tandem duplicated genes in this bin. GE indicates the gene expression level during vegetative growth (SPP medium), plotted as the median FPKM value. Note that the current chromosome-level assembly was generated based on short reads (e.g. Illumina), and the centromeric and some of subtelomeric regions are still incompletely assembled, which is the likely reason for the weak patterns seen at some chromosome termini (for example, both termini of chr2). chr2, Chromosome 2; CSG, conserved genes; FPKM, fragments per kilobase of exon per million reads mapped; GE, gene expression; IES, internal eliminated sequence; Ka/Ks, ratio of nonsynonymous to synonymous substitutions; LRR, leucine-rich repeat; MIC, micronucleus; SPP, Super Proteose Peptone; SSG, species-specific gene.

In *T*. *thermophila*, the germline (MIC) contains 5 pairs of metacentric chromosomes [24, 25]. Recently, chromosome-level assemblies of all 5 chromosomes were generated, and the limits of centromeric regions of each MIC chromosome were rigorously established [20], allowing us to map gene distribution patterns. We aligned *T*. *thermophila’s* most conserved (category X, found in all 10 species) and least conserved (*T*. *thermophila*-specific) genes with the MDS of the 5 MIC chromosome assemblies. In general, species-specific genes are highly enriched in the pericentromeric and subtelomeric regions (approximately 2 Mb near the midpoints of the centromeric regions, and the terminal approximately 1 Mb at both ends of the chromosome assemblies—note that these are arbitrary lengths based on the observed gene distribution patterns). In contrast, species-specific genes are depleted in MIC chromosome arms (Fig 3). Conversely, the most conserved genes are enriched on MIC chromosome arms and depleted in MIC pericentromeric and subtelomeric regions (Fig 3). The same differential localization is observed for species-specific LRR genes, the largest group of species-specific genes (Fig 3). This differential localization supports the hypothesis that, in *T*. *thermophila*, species-specific genes are preferentially generated and/or retained within the pericentromeric and subtelomeric regions of the MIC genome. In a subsequent section, we provide additional support for the preferential generation hypothesis.

### Species-specific genes show relatively rapid evolution, high incidence of tandem duplication, and low expression in axenic culture

The abundance of species-specific genes in *Tetrahymena* raises several questions that are addressed in this section. By what mechanism(s) have they expanded? Have they experienced reduced selective pressure, perhaps suggestive of sub- or neo-functionalization? Are they engaged in essential metabolic functions or rather more specialized functions?

Tandem gene duplication is a major way to generate raw material for genome innovation [26]. The initial analysis of the *T*. *thermophila* MAC genome sequence uncovered a high proportion of tandemly duplicated genes [19]. We have further investigated this phenomenon using comparative genomics. We determined the proportion of strictly tandemly arranged genes (no interspersion of unrelated genes) in each ortholog cluster and compared these frequencies among the 10 categories of ortholog clusters. We found far higher frequencies among the species-specific group (category I; range 8%–51%) compared with the most conserved group (category X; range 7%–14%; S3 Fig). Singletons, i.e., genes that do not fall into any OrthoMCL gene cluster in any of the 10 species (S2 Fig), and which thus show the greatest divergence, are also tandemly arranged (with respect to other singletons) with high frequency (S3 Fig) and show an absence of interspersion with Category I–X genes.

The largest tandemly duplicated gene cluster found in any of the 10 species was an LRR gene cluster in *T*. *pyriformis* consisting of 17 strict tandem inparalogs, extending to 65 LRR genes when the criterion used to identify tandemly duplicated genes was relaxed by allowing 2 inparalogs to be separated by no more than 3 other genes (S4 Fig). Although this tandem LRR gene cluster belongs to an ortholog group with genes present in 4 species (category IV), the extensive expansion is species specific, i.e., restricted to *T*. *pyriformis*. Additional species-specific clusters of tandemly duplicated LRR genes are found in other species (e.g. S5 Fig). As with the distribution of species specific, the proportion of tandemly duplicated genes is higher in the pericentromeric and subtelomeric regions than on chromosome arms (Fig 3).

The Ka/Ks value (the ratio of nonsynonymous to synonymous substitutions) is informative regarding the selective pressures encountered in gene evolution [27]. When paralogs in different *Tetrahymena* species are compared, pairwise Ka/Ks values are significantly higher (Mann Whitney U test, *p* < 0.01) for category I and II genes (i.e., genes in ortholog clusters represented in only 1 or 2 species), with median Ka/Ks values 0.25 and 0.30, respectively, than for categories III–X genes, with median Ka/Ks values between 0.03 and 0.07 (S6A Fig). Ka/Ks values less than 1 indicate that the genes underwent purifying selection. However, the higher pairwise Ka/Ks ratios of species-specific genes suggest relatively relaxed pressures of purifying selection in their evolution. This difference is especially true for LRR genes, for which the median Ka/Ks value of approximately 0.4 for categories I and II genes is 20 times higher than the approximate 0.02 value for category X genes (S6B Fig). Mapping Ka/Ks values of all genes to the *T*. *thermophila* MIC chromosomes shows that genes within pericentromeric and subtelomeric regions have higher Ka/Ks ratios than those within chromosomes arms (Fig 3). This distribution of Ka/Ks ratios resembles the gene distribution pattern of species-specific genes and provides independent evidence for the preferential localization of rapidly evolving genes to pericentromeric and subtelomeric chromosomal regions.

In its natural aquatic environment, bacteria are the principal food source of *Tetrahymena*, but it can also grow in axenic culture in the laboratory [28]. *Tetrahymena* can take up particulate nutrients through phagocytosis and absorb soluble nutrients in growth media through its membrane system [29]. When expression levels of *T*. *thermophila* genes during vegetative growth in axenic culture (Super Proteose Peptone [SPP] medium) are plotted against MIC chromosomal location, these levels are lower in pericentric and subtelomeric regions and higher on MIC chromosome arms (Fig 3). Directly checking the expression of species-specific genes shows that most of them are silenced or have very low expression during vegetative growth in axenic culture, the overall expression level being much lower than the whole-proteome background (S7A Fig). Further analysis of genes in different categories of gene clusters shows that gene expression levels correlate with the degree of gene conservation: conserved genes (categories II–X) are more highly expressed than species-specific genes (S7B Fig). This finding suggests that most species-specific genes do not function in basic structural or metabolic functions or in absorbing the soluble nutrients found in axenic culture. Further experimentation may reveal growth conditions under which these species-specific genes are expressed.

### LRR genes make the largest contribution to the high level of genome divergence and show unique 90-bp exon arrays, phase 2 introns, and diverse gene structures

The number of genes containing the 7 most frequent species-specific protein domains has extensively expanded in every *Tetrahymena* species (S2 Table). This is especially true for LRR genes: all but 2 species (*T*. *empidokyrea* and *T*. *shanghaiensis*) have more than 1,000 LRR genes, and *T*. *pyriformis* has over 2,000 (S2 Table), more than 6% of its total genes. Indeed, LRR genes numerically compose the largest category of species-specific genes in *Tetrahymena* (Fig 2B and S2 Table). Furthermore, in any given *Tetrahymena* species, about 45%–70% of LRR genes are specific to that species (except for *T*. *empidokyrea*, the mosquito parasite). This range is significantly higher (chi-squared test *p* < 1 × 10^−5^) than that for all pooled species-specific genes in each species (15%–38%). Therefore, LRR genes form the largest gene category contributing to the high level of genome divergence between the 10 morphologically similar *Tetrahymena* species investigated here. LRR is a widespread protein structural motif, found in all life forms, that is generally 20 to 29 residues long and often involved in protein–protein interactions. Genes with different numbers of LRRs can form a variety of protein structures [30] and perform many different functions.

**Fig 4:**
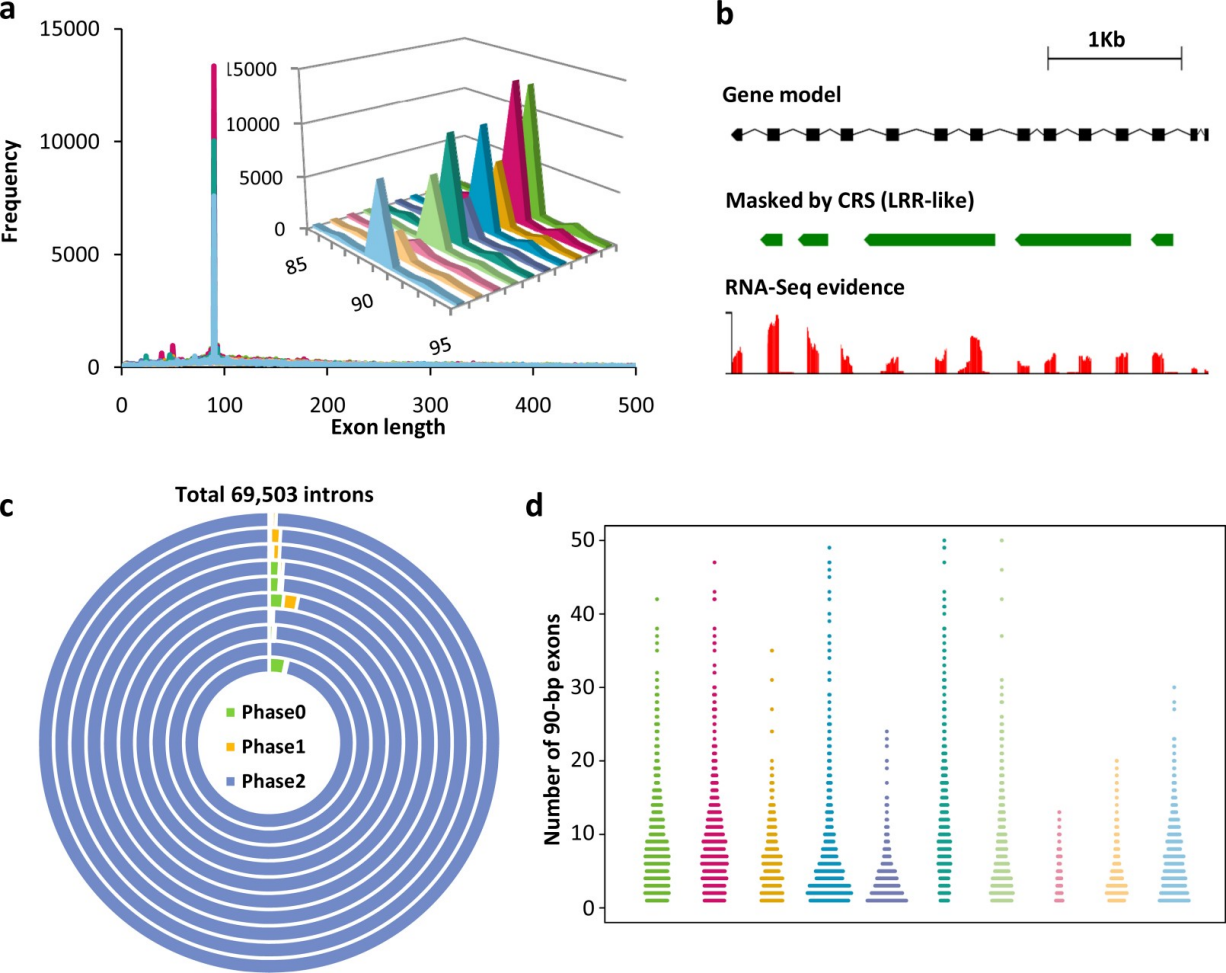
Unique features of LRR genes in *Tetrahymena*. (a) LRR gene exon length distributions for all 10 *Tetrahymena* species. Every species shows an exon peak at 90 bp, representing the exactly 90-bp exon arrays. An inset shows the detailed exon distribution range from 85 to 95 bp in length. From right to left (inset), species are *T*. *thermophila*, *T*. *malaccensis*, *T*. *elliotti*, *T*. *pyriformis*, *T*. *vorax*, *T*. *borealis*, *T*. *canadensis*, *T*. *empidokyrea* (mosquito parasite), *T*. *shanghaiensis*, and *T*. *paravorax*. (b) LRR gene TTHERM_000586765, an example of a 90-bp exon array gene masked by at least 1 of the 8 de novo–identified MAC LRR gene CRSs. (c) Extreme phase 2 bias of introns among 90-bp exon containing LRR genes in 10 *Tetrahymena* species. The 10 concentric circles represent the 10 species, from inside to outside: *T*. *thermophila*, *T*. *malaccensis*, *T*. *elliotti*, *T*. *pyriformis*, *T*. *vorax*, *T*. *borealis*, *T*. *canadensis*, *T*. *empidokyrea* (mosquito parasite), *T*. *shanghaiensis*, and *T*. *paravorax*. (d) Highly variable numbers of 90-bp exons in different LRR genes in all 10 species. The numbers of 90-bp exons in different genes were used to make the dot plot. From left to right, species are *T*. *thermophila*, *T*. *malaccensis*, *T*. *elliotti*, *T*. *pyriformis*, *T*. *vorax*, *T*. *borealis*, *T*. *canadensis*, *T*. *empidokyrea* (mosquito parasite), *T*. *shanghaiensis*, and *T*. *paravorax*. The color scheme is the same as panel A. Numerical data underlying this panel are listed in S2 Data. CRS, consensus repeat sequence; LRR, leucine-rich repeat; MAC, macronucleus; RNA-Seq, RNA sequencing.

A striking feature of the MAC (somatic) genomes of the 10 *Tetrahymena* species is the large number of exons that are exactly 90 bp in length (Fig 4A and 4B). In silico functional annotation of genes containing at least one 90-bp exon showed that 76% of them are LRR genes (S8A Fig). Among LRR genes containing 90-bp exons, 77% of them have at least 3 consecutive 90-bp exons, which we will refer to as 90-bp exon arrays (Fig 4B). Extreme intron phase bias associated with 90-bp exons was found in the LRR genes. The overwhelming majority of introns (>98%) that precede 90-bp exons (tens of thousands of them) are phase 2 introns, meaning that the intron is inserted at position 2 of a codon (Fig 4C). This extreme bias is observed regardless of the number of 90-bp exons in the gene (S3 Table). Comparison controls (introns in LRR genes that lack 90-bp exons and introns preceding 90-bp exons in non-LRR genes) show no phase 2 bias (34%) or a mild phase 2 underrepresentation (24%), respectively (S9 Fig). This extreme intron phase bias of LRR 90-bp exons is observed in all 10 *Tetrahymena* species investigated here (Fig 4C). A consequence of this extreme phase 2 intron bias is that, in an array of consecutive 90-bp exons, the 5′ base pair of any exon functions in concert with the two 3′ base pairs of the previous exon to encode a”junction” amino acid. Thus, every 90-bp exon in the array contributes full or partial sequence information for the translation of 31 amino acids. The entire array of consecutive 90-bp exons thus behaves translationally as an interlocked unit. As far as we know, tandem arrays of exactly 90-bp exons and phase 2 introns have not been described in LRR genes of any other organism.

In *Tetrahymena*, the unique 90-bp exon structure can be used as an indicator to measure the number of LRRs in a gene (which cannot always be identified by common protein sequence similarity algorithms). When we focused on LRR genes containing 90-bp exons, the number of 90-bp exons in LRR genes showed large variation, ranging from 1 to 50 in most genes (Fig 4D), even among LRR genes that are clustered as inparalogs (S10 Fig). Thus 90-bp exon-containing LRR genes encode diverse protein structures with potentially different functions.

### LRR genes in *Tetrahymena* MAC genomes fall into three major age groups

To further investigate the 90-bp exon-containing LRR genes, we first focused on the LRR genes in the model species, *T*. *thermophila*. In this species, 80% (1,500 out of 1,874) of LRR genes contain at least one 90-bp exon (S8B and S8C Fig). This finding raises the possibility of relatively recent evolutionary expansion that might be detected by the presence of repetitive sequences within an LRR gene or among different LRR genes. Therefore, we used RepeatModeler to identify nucleotide consensus repeat sequences (CRSs; the output consensus sequences of RepeatModeler) in the *T*. *thermophila* MAC genome. We de novo–identified 8 CRSs (CRS1–CRS8, see S4 Table and S3 Data) that mask LRR genes (Fig 4B). Taken together, they mask 53% (986 out of 1,874) of *T*. *thermophila* LRR genes (S8D Fig).

**Fig 5:**
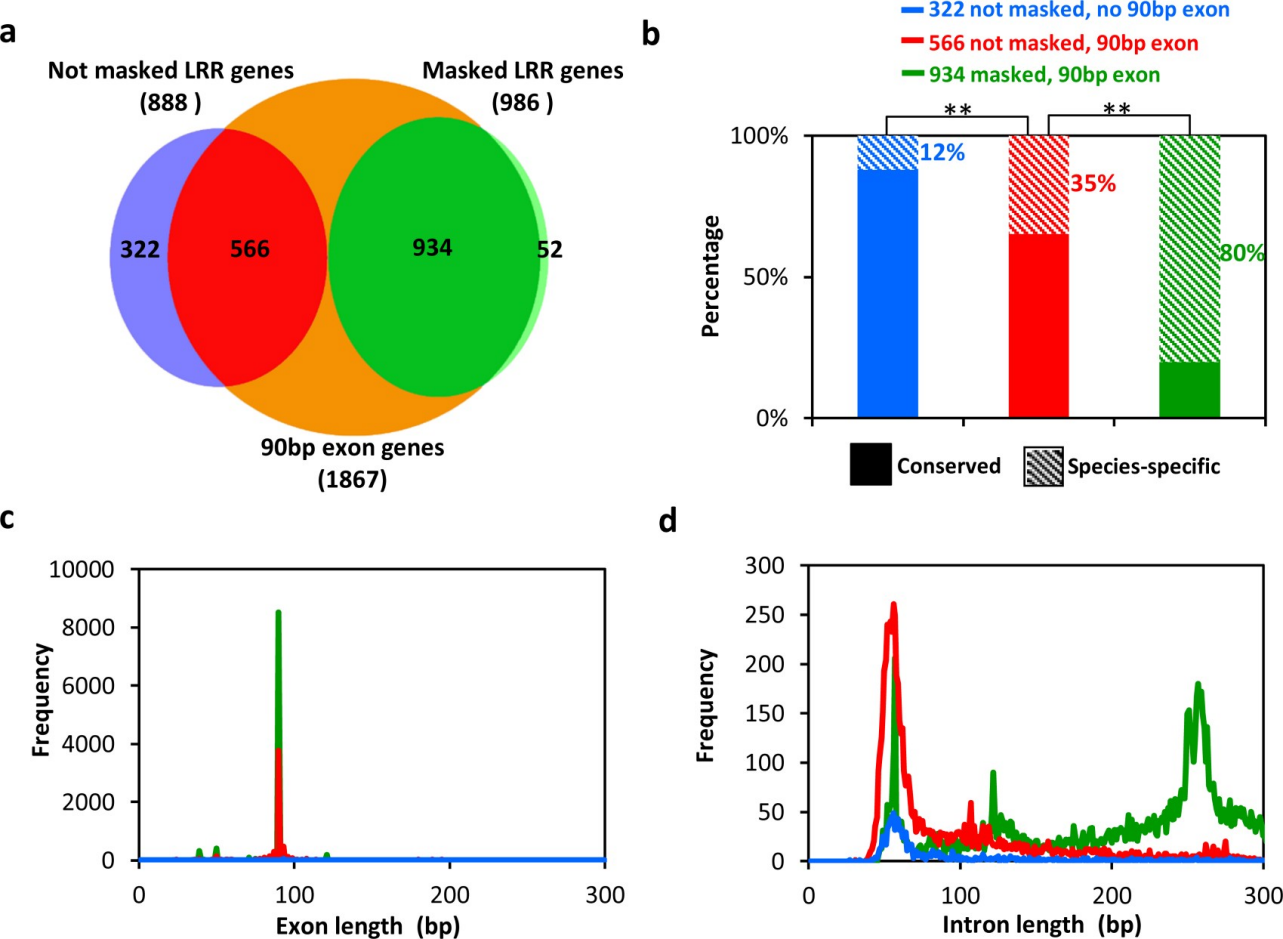
*T*. *thermophila* LRR genes can be sorted into 3 groups with different properties. (a) LRR genes are sorted into 3 groups based on the presence or absence of 90-bp exons and on MAC CRS masking. (b) Ratios of species-specific to conserved genes among the 3 groups of LRR genes. Two asterisks indicate a significant difference between 2 groups (chi-squared test, *p* < 1 × 10^−5^). (c) Exon length distributions of the 3 groups of LRR genes. (d) Intron length distributions for the 3 groups of LRR genes. Colors in (c) and (d) are as in (b). CRS, consensus repeat sequence; LRR, leucine-rich repeat; MAC, macronucleus.

*T*. *thermophila* LRR genes can be sorted into 3 groups depending on whether they contain at least one 90-bp exon and whether they are masked by at least 1 of the 8 CRSs (Fig 5A). Group I consists of 322 genes that lack 90-bp exons and are not masked by any CRS. Group II consists of 566 genes that have at least one 90-bp exon and are not masked by any CRS. Group III consists of 934 genes that have at least one 90-bp exon and are masked by at least 1 CRS. Group II and III LRR genes generally contain arrays with tandem repeats of at least three 90-bp consecutive exons: 80% (456 out of 566) for group II and 87% (815 out of 934) for group III.

The fraction of species-specific LRR genes increases from group I to group III (Fig 5B). Only 12% of group I LRR genes are species specific, while the percentages are 35% in group II and 80% in group III (Fig 5B). This finding suggests that LRR genes in *T*. *thermophila* fall into 3 major age groups and that group III mainly contains the youngest LRR genes. To validate this conclusion, we investigated additional features of the 3 groups.

Group I genes, which lack 90-bp exons (Fig 5C), appear to be the oldest *T*. *thermophila* LRR genes. Most group I LRR genes (88%) are conserved (i.e., not species specific, suggesting earlier origin) (Fig 5B) and generally have fewer than 10 introns (92%) (S11A Fig). These traits are shared by the LRR genes of the more distantly related *P*. *tetraurelia*, which lack a 90-bp exon frequency peak and have few introns (S12 Fig). Introns of group I genes are usually approximately 56 bp long, similar to the genome background intron length (i.e., all predicted genes; Fig 5D and S13 Fig). These results suggest that ancestors of group I LRR genes originated before the *Paramecium-Tetrahymena* divergence. The GC content of *T*. *thermophila* group I genes is also similar to that of the genome background (approximately 27% for CDS; S11B Fig), and their leucine codon usage is similar to that of non-LRR genes (S11C Fig). In contrast, group II and III genes have 90-bp exons, usually in arrays of at least 3 exons, and contain more introns (57% and 49% of genes in group II and III, respectively, have more than 10 introns) (S11A Fig). To the best of our knowledge, arrays of exactly 90-bp exons encoding LRR domains is a feature unique to *Tetrahymena*, which suggests that group II and III genes originated later than group I genes, after the *Paramecium-Tetrahymena* divergence.

Several observations strongly suggest that group III LRR genes are younger than group II genes. The former contain intragenic repeat units detectable at the nucleotide sequence level (masked by at least 1 CRS), which are lacking in group II LRR genes (S14 Fig). Furthermore, group II LRR genes have shorter introns, with a length distribution similar to that of group I genes and to the genome background length peak (approximately 56 bp; Fig 5D). In contrast, group III LRR genes usually have longer introns (main distribution peak at approximately 257 bp; Fig 5D and S14A Fig). Also, nucleotide conservation profiles of 90-bp exons show that group III LRR genes have greater intraspecies sequence conservation than group II LRR genes in all *Tetrahymena* species investigated (S15 Fig, S16 Fig and S17 Fig). All these findings suggest that group III represents the youngest group of LRR genes. There are additional characteristic differences between groups II and III. Group III LRR genes have a higher GC content than group I and II LRR genes, whose GC content is similar to the genome background (S11B Fig). Also, group III genes show different leucine codon preferences than those of group I and II genes (S11C Fig).

### Recurrent waves of clonal expansion have shaped the evolutionary landscape of *Tetrahymena* LRR genes

#### Conservation profiles provide evidence of clonal expansions in *Tetrahymena* LRR genes

Using the same methods and criteria as in *T*. *thermophila*, we also identified LRR CRSs in the other 9 *Tetrahymena* species (S4 Table and S3 Data) and sorted their LRR genes into groups I, II, and III (S18 Fig) with the exception of *T*. *empidokyrea*, the only parasitic *Tetrahymena* examined here, which lacks the youngest group (III) of LRR genes.

The 90-bp exons of both group II and III LRR genes show extreme leucine conservation at 6 positions in all 10 *Tetrahymena* species (S19 Fig and S20 Fig). This observation, together with the exact 90-bp length and the extraordinary intron phase 2 bias, suggests that 90-bp exons originated by clonal expansion, by which we mean an increase in numbers by rounds of successive duplication starting from 1 or a few 90-bp exon(s) already present in the last common ancestor (LCA) of the 10 species, more than 300 Mya.

Amino acid conservation profiles of 90-bp exons of group II genes for 9 of the 10 species are very similar (S20 Fig). In contrast, the sequence profile *of T*. *paravorax* shows relatively higher conservation (S20 Fig and S5 Table). We next compared the sequence profiles between *T*. *paravorax* and the other 9 species using the Two Sample Logo tool based on the binomial statistical test. The amino acid profile of *T*. *paravorax* shows significant overrepresentation (35%–49%, S21A Fig) at some positions (9, 10, 12, 13, 15, 18, 20, 23, 24) compared to the other 9 species. At *T*. *paravorax* positions 3, 10, 11, 14, 17, and 20 (S5 Table), the most conserved amino acids are different from those in the other 9 species. The nucleotide sequence profile also showed significant overrepresentation at many positions (43%–50%, S21B Fig) compared to the other 9 species, especially at position 47, 59, 74, 80, and 82. These results suggest a later clonal expansion of group II exons in *T*. *paravorax*, after its divergence from the other 9 species.

The amino acid conservation profiles of group III exons show that, at several positions, different amino acids predominate in different species (S20 Fig). This is consistent with the hypothesis that group III exons resulted from later clonal expansions of progenitors having some differences from the consensus. For example, in contrast to the conserved leucine at position 16 in other species, isoleucine is the most common amino acid (85%) in *T*. *shanghaiensis* (S20 Fig), suggesting that the progenitor of the clonal expansion that generated the group III exons in *T*. *shanghaiensis* was a minority type with isoleucine at this position.

#### Intron length distributions provide independent evidence for recent independent waves of group III LRR gene repeat generation within different *Tetrahymena* species

A striking general feature of *Tetrahymena* group II and III LRR genes is that their 90-bp exons are usually found in tandem arrays of distinct intragenic repeat units (detectable at the nucleotide level) containing at least 3 consecutive exons (S14 Fig). For example, *T*. *thermophila* shows a peak at approximately 10 exons (S11A Fig). The introns in 90-bp exon arrays show characteristic lengths in each *Tetrahymena* species that has group III LRR genes (S18 Fig, column 4). For example, in *T*. *thermophila*, most group III LRR gene introns are approximately 257 bp in length (S18 Fig, column 4, green line), and therefore repeat units within LRR genes are approximately 347 bp long (90-bp exon + 257-bp intron). In *T*. *malaccensis*, its closest related species, the intron length of group III LRR genes peaks at about 232 bp. Such differences, also seen in other *Tetrahymena* species (S18 Fig, column 4, green lines), suggest that group III LRR genes expanded independently through recent lineage-specific events within individual species.

Although the main intron distribution peak of *T*. *thermophila* group III LRR genes is located at about 257 bp, we also see a sharp secondary peak at about 122 bp for a subset of 69 group III genes, which is exclusively masked by CRS1 (Fig 5D and S22 Fig). This result suggests a recent expansion of a subset of LRR genes originating from a *T*. *thermophila* LRR gene with at least one 122-bp intron. Interestingly, similar secondary intron length distribution peaks of group III LRR genes are seen in other species, including *T*. *pyriformis*, *T*. *vorax*, *T*. *shanghaiensis*, and *T*. *paravorax* (S18 Fig, column 4, green lines). These secondary intron length peaks imply that clonal expansions of subsets of LRR genes continue to independently occur in various species and can in principle account for the species-specific intron length distributions found today.

#### Very recent clonal expansions of 90-bp exons within group III LRR genes are revealed by CRS masking

As reported above, group III genes were assigned to subgroups based on CRS-masking scores. Most *Tetrahymena* CRS-masked exons fall into species-specific clusters or lineage-specific clusters (e.g., *T*. *thermophila* and *T*. *malaccensis*, or *T*. *borealis* and *T*. *canadensis* lineages) (S19 Fig), suggesting that clonal expansions of group III genes have continued to occur in different lineages or species.

Seven CRSs, which we refer to as “exceptional CRSs,” cluster differently from all the others (S19B Fig, green clade). A phylogenetic analysis of exons masked by the various CRSs shows that 90-bp exons masked by exceptional CRSs of several distantly related species cluster together (S19B Fig, green clade), suggesting that the ancestor of these exons arose very early, pre-dating speciation within the *Tetrahymena* genus. However, other observations suggest that exons masked by exceptional CRSs expanded recently, in independent lineages. First, these exons still show intraspecies nucleotide level conservation, as they are masked by the exceptional CRSs. Second, nucleotide conservation profiles of *T*. *thermophila* CRS1-masked exons reveal nucleotide flips or greater conservation of the same nucleotide at particular positions relative to exons masked by the other 7 CRSs (S19C Fig and S23 Fig), indicative of clonal expansion too recent for mutation-induced nucleotide randomization to have occurred. Third, in *T*. *thermophila*, the intron length distributions of CRS1-masked 90-bp exons has a sharp, approximately 122-bp intron peak, absent from the length distribution of introns masked by all the other 7 CRSs (approximately 257-bp intron peak; S22 Fig), also indicative of recent clonal expansion. Thus, the results obtained with exceptional CRSs support our conclusion that recent clonal expansions have independently occurred in various species.

In summary, our analyses of 90-bp exons of *Tetrahymena* LRR genes provide evidence of recurring waves of clonal expansion of 90-bp exons. The first detectable expansion occurred after the generation of the first exactly 90-bp exons and phase 2 introns in *Tetrahymena* LRR genes. This expansion occurred after the *Tetrahymena-Paramecium* divergence but preceded the divergence of the 10 currently sequenced *Tetrahymena* species. More recent expansion waves generated the group III genes that, within species, still retain nucleotide conservation with one another. The most recent expansions have generated multiple clades of lineage-specific or species-specific 90-bp exons, detected by CRS masking and as secondary peaks in the intron length landscape.

### LRR gene evolutionary innovation: Proposed mechanisms at work

#### The strength of preferential localization of *T*. *thermophila* LRR genes to pericentromeric and subtelomeric regions of MIC chromosomes is related to the age of the genes

**Fig 6:**
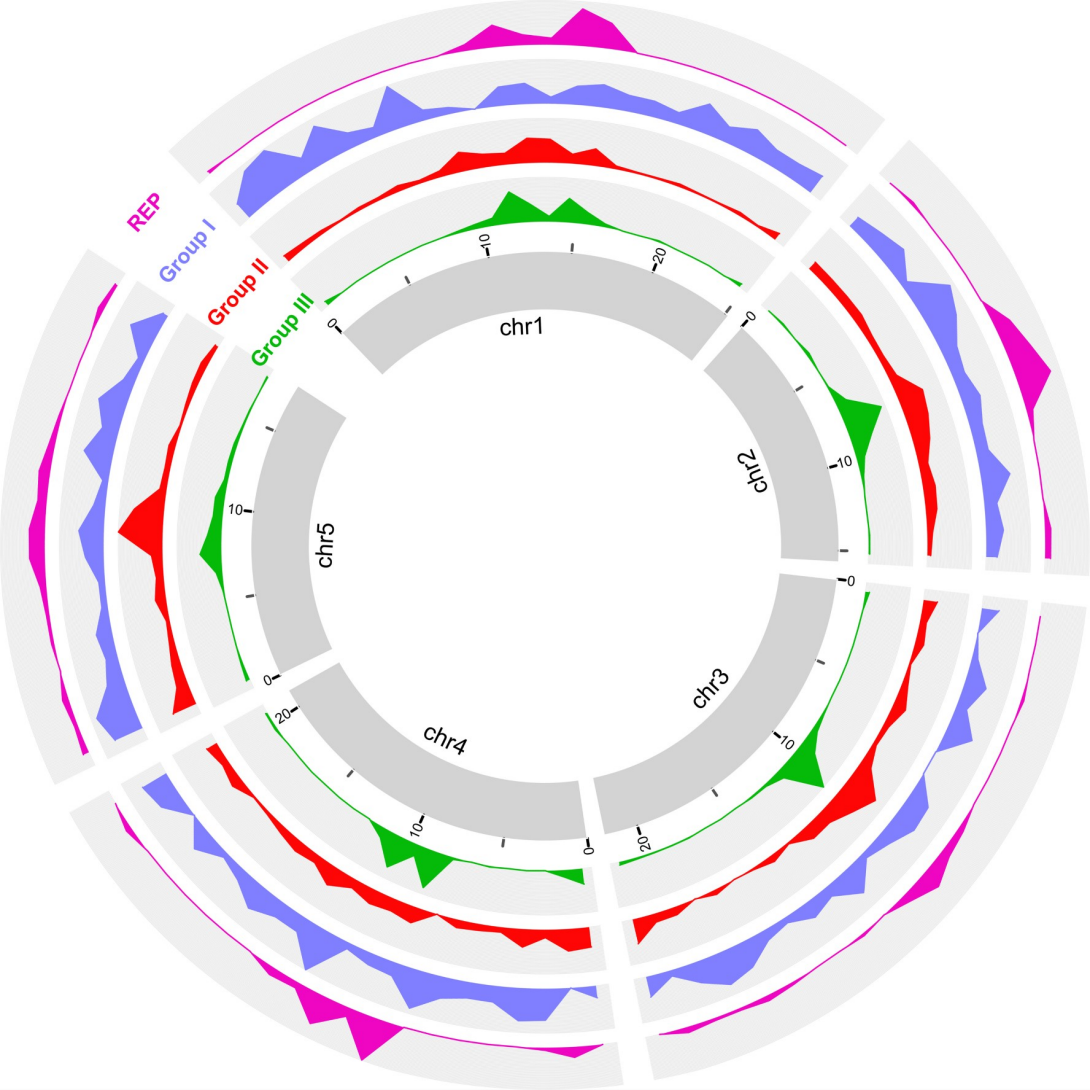
Differential distributions of the 3 groups of LRR genes and the 3′ terminal segment of Tt.*REP*s along the 5 *T*. *thermophila* MIC chromosomes. Central ring: the 5 chromosomes in the *T*. *thermophila* MIC genome; group III: group III LRR genes; y-axis, number of genes. Group III: group II LRR genes; group I: group I LRR genes; *REP*: 54-bp conserved sequences at 3′ end of Tt.*REP*s; y-axis, number of Tt.*REP*s (represented by 54-bp conserved sequences). chr1, Chromosome 1; LRR, leucine-rich repeat; MIC, micronucleus; Tt.*REP*, *T*. *thermophila* REP retrotransposon.

We have reported above that species-specific *T*. *thermophila* genes show preferential localization to pericentromeric and subtelomeric regions of MIC chromosomes. The 3 age groups of LRR genes in *T*. *thermophila* show different frequencies of species-specific gene content (Fig 5B). Therefore, it is reasonable to expect differential localizations of these 3 groups of LRR genes on the MIC chromosomes. By mapping all the *T*. *thermophila* LRR genes in the 3 groups to the MIC chromosomes, we found that the youngest (group III) LRR genes, as expected, are highly enriched in the pericentromeric and subtelomeric regions (Fig 6). Group II LRR genes are somewhat enriched in the pericentromeric regions but are also found in chromosome arms (Fig 6). The oldest group of LRR genes, which have the highest proportion of conserved genes, are mainly distributed on the MIC chromosome arms (Fig 6). Our finding that the younger LRR genes are the higher is their concentration in pericentromeric and subtelomeric regions of MIC chromosomes strengthens the conclusion that these chromosomal regions are the highest source of genomic innovation in *Tetrahymena*.

#### *Tetrahymena* LRR genes show evidence of extensive ectopic recombination

Duplication by ectopic recombination (i.e., unequal crossing over) is generally considered to be the most common way of creating new genes [31, 32]. In *T*. *thermophila*, only about 1.6% of LRR genes appear to be the result of direct tandem duplications of entire genes. Most LRR genes show highly variable numbers of 90-bp exons, suggesting the occurrence of frequent inter- and intra-genic ectopic recombination. For example, the number of 90-bp exons in LRR genes ranges from 1 to 51 in *T*. *thermophila*. This suggests that extensive ectopic recombination has occurred during the evolution of LRR genes—not just gene duplications, which would not change the number of 90-bp exons per gene. To demonstrate the occurrence of ectopic recombination, we focused on group III LRR genes because they still show 90-bp exon sequence conservation at the nucleotide level (see also S17 Fig). We found that inparalogs in a gene cluster (ortholog group) with high sequence similarity to one another show high variability in the number of 90-bp exons (S10 Fig). This result strongly suggests that extensive ectopic recombination between LRR genes has generated inparalogs with diverse gene structures (i.e., different number of repeats).

**Fig 7:**
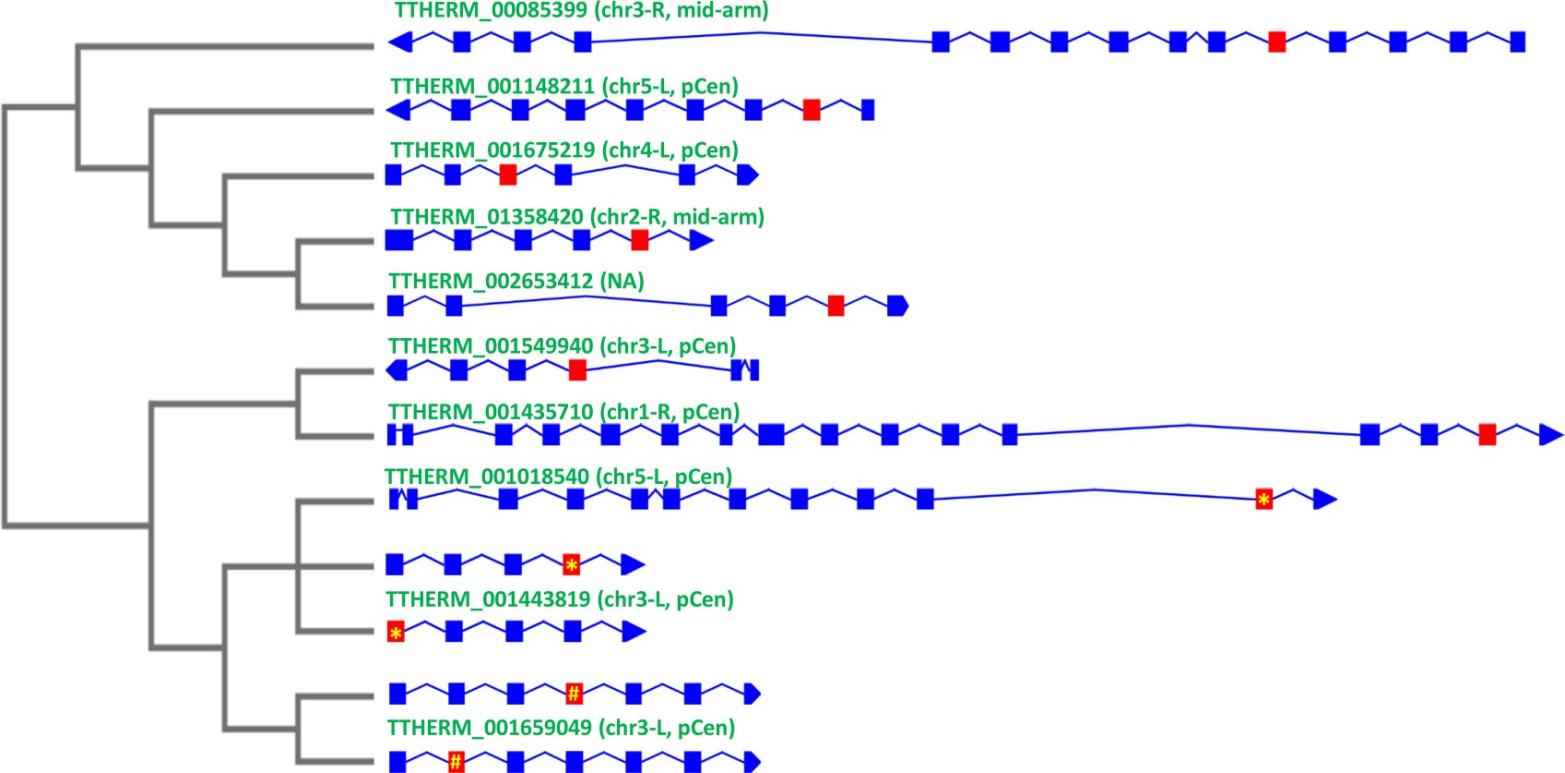
Phylogeny and MIC chromosome distribution of 12 nearly identical 90-bp exons in different *T*. *thermophila* LRR genes give evidence of extensive ectopic recombination. Right: intron/exon diagram of the 10 LRR genes containing the twelve 90-bp exons (shown in red) that share between 88 and 90 identical nucleotides. Listed above each gene: MIC chromosome location. L or R indicates the left or right arm of chromosome. Left: a maximum likelihood phylogenetic tree based exclusively on these twelve 90-bp exons. Note that (a) this is the largest group of nearly identical 90-bp exons and (b) TTHERM_001443819 and TTHERM_00001659049 both have 2 exons that belong to this group. Identical 90-bp exons share the same symbol: yellow asterisk (*) or number sign (#). chr3, Chromosome 3; LRR, leucine-rich repeat; MIC, micronucleus; mid-arm, near the middle of chromosome arms; NA, not available (the gene is located in still unassembled region of MIC genome); pCen, pericentromeric region.

To elucidate the evolutionary relationships among 90-bp exons of inparalogs, we investigated a *T*. *thermophila* LRR gene cluster consisting of 83 inparalogs. This cluster contains a total of 1,932 90-bp exons—an average of approximately 23 exons per gene. A phylogenetic tree of all the 90-bp exons (S24 Fig) clearly shows that extensive ectopic recombination has scrambled the sequence of the exons. Very often, the sequence of an exon is more closely related to that of an exon in a different gene of the cluster than to other exons in the same gene (S24 Fig). To further validate this result, we clustered the nucleotide sequences of all 90 bp-exons with identity levels of 97% or greater (i.e., allowing no more than 2 nucleotides’ difference between 2 exons). The largest cluster contains twelve 90-bp exons, and these exons are found in different LRR genes with diverse gene structures on different MIC chromosomes (Fig 7, S25 Fig and S26 Fig). Ectopic recombination or gene conversion between 2 different exons in the same or different genes are possible mechanisms that can explain these observations. Exons of 90 bp participating in ectopic recombination need not belong to inparalogs; the same events can in principle occur between 90-bp exons of the same gene or of any 2 different LRR genes. Of course, only ectopic recombination that occurs in the MIC can be perpetuated. Recombination between nonhomologous chromosomes could often result in chromosomal rearrangements, which may become apparent when high-quality MIC genome assemblies are available for comparison to that of *T*. *thermophila*.

#### Group III 90-bp exon array LRR genes show preferential physical association with IESs in the *T*. *thermophila* MIC genome

As described in a previous section, the youngest *T*. *thermophila* LRR genes are highly enriched in the pericentromeric and subtelomeric regions of all 5 MIC chromosomes. These regions are also enriched with IESs [20] (S27 Fig), which include active and inactive TEs, other undefined repetitive sequence, as well as undefined unique sequence [20]. In the *T*. *thermophila* MIC, we have found that most (72%) species-specific LRR genes are flanked by IESs (S28 Fig). This percentage is significantly higher than for other species-specific genes (72% versus 56%; chi-squared test *p* < 1 × 10^−5^). These findings raise the possibility that IESs, and perhaps TEs within them, contribute to the generation of new species-specific genes.

To further investigate this possibility, we analyzed previously reported MIC CRSs [20] and found that some mask both MDSs and IESs (S29 Fig). A notable case is the MIC CRS named “Contig[0735]#nonTE/REP-MAC” [20], which masks >3,000 MIC DNA segments. The 5′ segment of this MIC CRS shares significant nucleotide sequence identity to 7 of the 8 LRR gene-related MAC CRSs described in the previous section. Therefore, we will here abbreviate the name of that MIC CRS as tLRR-MIC-CRS. Compared to other MIC CRSs, tLRR-MIC-CRS is exceptional in that 80% of the masked DNA segments are located in MDS (i.e., also in the MAC). We now understand why: tLRR-MIC-CRS masks group III LRR genes containing 90-bp exon genes. Hundreds of tLRR-MIC-CRS–masked MIC DNA segments contain IESs (S29 Fig), as the mask’s 3′ segment represents MIC IES sequence.

#### A Non-long terminal repeat (non-LTR) retrotransposon is found in strong physical association with LRR gene repeat units in the *T*. *thermophila* and *T*. *malaccensis* MIC genomes

**Fig 8:**
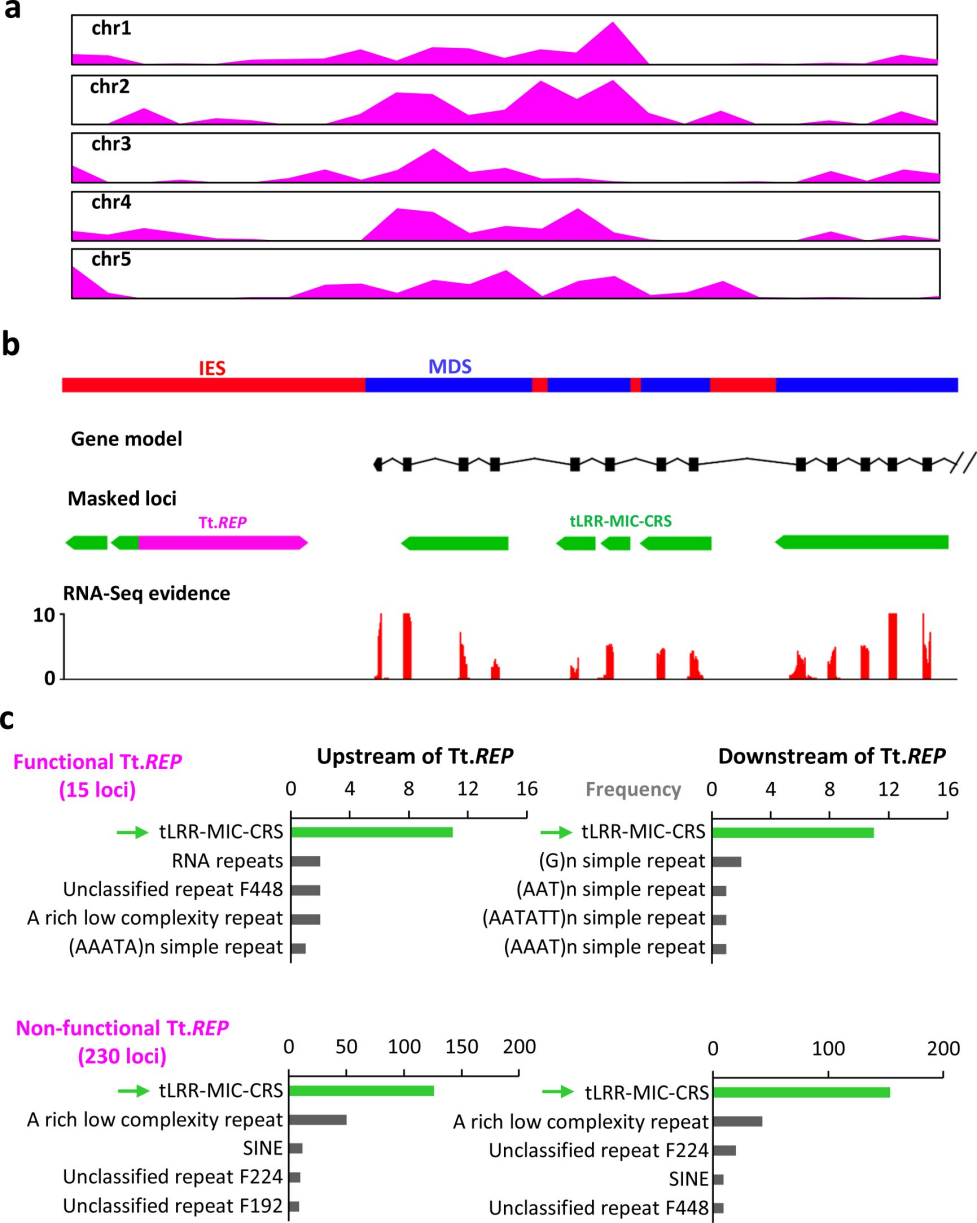
Group III LRR gene repeats are the most common element flanking non-LTR *REP* retrotransposons in the *T*. *thermophila* MIC genome. (a) Tt.*REP* sequence distribution across 5 MIC chromosomes in *T*. *thermophila*, normalized to the same length. (b) Example of a Tt.*REP* (non-LTR *REP* retrotransposon in *T*. *thermophila*) fragment flanking tLRR-MIC-CRS–masked segments of a functional group III LRR gene (TTHERM_01344670), containing a 90-bp exon array and supported by RNA-Seq gene expression data. (c) MIC sequences masked by tLRR-MIC-CRS are most frequently flanked on one or both sides by Tt.*REP*. Green bar and arrow, tLRR-MIC-CRS–masked loci; black bars, loci masked by the next most frequent repeat families or low-complexity sequences. Numerical data underlying this panel are listed in S2 Data. chr1, Chromosome 1; CRS, consensus repeat sequence; IES, internal eliminated sequence; LRR, leucine-rich repeat; MAC, macronucleus; MDS, MAC-destined sequence; MIC, micronucleus; non-LTR, Non-long terminal repeat; RNA-Seq, RNA sequencing; Tt.*REP*, *T*. *thermophila* REP retrotransposon.

A BLAST search of the National Center for Biotechnology Information (NCBI) nonredundant nucleotide sequence Reference Database using the 8 *T*. *thermophila* MAC CRSs shows that CRS6 matches a highly conserved 54-bp sequence at the 3′ end of the previously identified *T*. *thermophila* non-LTR retrotransposon, Tt.*REP* [33] (S30 Fig). Genome-wide analysis showed that 169 copies of the conserved 54-bp “tail” of Tt.*REP* are retained in the MAC genome (in contrast to most of Tt.*REP*’s sequence, which is excluded from the MAC genome by developmental IES excision), and the distribution of those 54-bp sequences is highly correlated with the distribution of group III LRR genes in MIC chromosomes (Fig 6 and S31 Fig). Furthermore, CRS6 masks 3 tandem copies of what was originally called a “complex direct repeat” [33] (size approximately 350 bp) adjacent to Tt.*REP* and that we now recognize as 90-bp exon/intron repeats of group III LRR genes. In addition, by checking the relatively recently expanded copies of Tt.*REP2* (a previously reported functional copy of Tt.*REP* [33]), we found that the tLRR-MIC-CRS repeat is usually located near Tt.*REP* (S32 Fig). As expected from previous findings about the distribution of TE elements in the *T*. *thermophila* MIC [20], Tt.*REP*s are highly enriched in pericentromeric and subtelomeric regions, as are the species-specific LRR genes (Fig 8A).

Analysis of nearby repeat sequences shows that most Tt.*REP* elements (76%) colocalize with LRR repeat units (Fig 8B and 8C). More importantly, LRR repeat units are usually adjacent to both ends of Tt.*REP*, regardless of whether this TE is considered functional (i.e., has the 2 intact open reading frames of Tt.*REP*, S30A Fig) or nonfunctional (i.e., lacks at least 1 intact open reading frame) (Fig 8C). These neighboring LRR repeat units (approximately 62%) belong to functional protein-coding genes, some of which are expressed in the MAC (Fig 8B). These findings indicate an intimate physical relationship between Tt.*REP* and LRR genes.

To further validate the physical association between LRR gene repeat units and non-LTR *REP* retrotransposons in the *Tetrahymena* MIC genome, we obtained a draft MIC genome assembly of *T*. *malaccensis* (the species most closely related to *T*. *thermophila*). We used long-read PacBio single-molecule real-time (SMRT) sequencing technology, which allows much more complete assembly of long repeated elements like *REP*. Most (60%) non-LTR *REP* retrotransposons in *T*. *malaccensis* (Tm.*REP*), and especially the functional ones (86%), were flanked by the *T*. *thermophila* “complex direct repeat” homolog, encoding an LRR 90-bp exon and intron sequence unit (S33 Fig). These combined findings in *T*. *thermophila* and *T*. *malaccensis* strengthen the evidence for a very specific and intimate association between *REP* and LRR genes.

## Discussion

### High genetic diversity is observed among the 10 *Tetrahymena* species

Morphospecies have been widely found in many eukaryotes across the tree of life, including mammals, birds, insects, nematodes [5], fungi, stramenopiles [34], myxosporeans [35], flagellates [36], amoebae [37], foraminiferans [38], and ciliates [8]. Among ciliates, morphospecies appear to be particularly common. Foissner and colleagues reported that there are 4,500 described free-living ciliate morphospecies [9], suggesting that some general evolutionary features may favor their appearance within this particular group of unicellular eukaryotes. Despite their morphological similarity, molecular evidence (such as 18S rDNA sequence) indicates high genetic diversity among ciliate morphospecies, such as in *Paramecium*, *Tetrahymena*, *Chilodonella*, *Carchesium*, and some choreotrich and oligotrich ciliates [39–45]. Recent single-cell omics data also support the high genomic diversity of ciliates [46]. This dichotomy suggests that morphological and molecular evolution may be decoupled in many ciliate species [43].

Nanney noted this phenomenon in *Tetrahymena* long ago [13, 21]. Based on the divergence of rDNA sequence, he estimated this genus has existed for approximately 300 million years although its morphological design has persisted unchanged [13, 21]. Our whole genome studies of *Tetrahymena* more fully demonstrate the features of high genetic diversity and ancient divergence despite high morphology conservation. From a macroevolutionary perspective, we observe high genetic diversity at the MAC genome level when comparing the 10 sequenced *Tetrahymena* species. This high genetic diversity is consistent with our independent estimate that the *Tetrahymena* genus originated approximately 300 Mya (Fig 1), about the origin time of amniotes [47].

Our study further shows that the high genomic diversity is particularly pronounced among genes encoding proteins with certain domains, LRR domain-containing genes being the most numerous (Fig 2). The 10 species share >8,000 orthologs (6,052 one-to-one orthologs) in all 10 species, a number even larger than the total genes in the yeast genome. On the other hand, thousands of genes, averaging nearly a quarter (24%) of their total proteome, are unique to each species. Considering that morphospecies appear to be particularly common among ciliates, our findings in *Tetrahymena* likely are the tip of an iceberg: the biological diversity of other ciliate morphospecies is likely to be very high when investigated at the genome level.

### Pericentromeric and subtelomeric regions of *T*. *thermophila* are genome innovation centers

The human genome sequencing project revealed that pericentromeric and subtelomeric regions are structurally complex [48], characterized by highly repetitive sequences and segmental duplication sequences specific to each chromosome arm [49]. In plants also, repetitive sequences are abundant in the centromeric, pericentromeric, and subtelomeric regions [50, 51]. In *Drosophila*, TEs are abundant in centromeric and pericentromeric regions [52]. In some species, subtelomeric regions have also been shown to be gene rich [53–55]. For example, many variable surface antigen genes are located in the subtelomeric regions of the chromosomes of *Plasmodium falciparum* [54]. Pericentromeric regions are often referred to as genomic junkyards, but they can also be the birthplace of new genes with novel functions [56, 57]. The occurrence of numerous duplications, transpositions, and rearrangements within pericentromeric and subtelomeric regions is associated with their being hotspots for eukaryotic chromosome evolution [58, 59].

In *T*. *thermophila*, the only *Tetrahymena* species with a chromosome-level MIC genome assembly, TEs and other IES sequences are enriched in pericentromeric and subtelomeric regions as well [20]; they likely are also enriched in centromeric regions, but the centromeres are currently poorly assembled precisely because of the abundance of repeated sequence. Our study reveals that many species-specific genes of various types, especially LRR genes, are also enriched in these regions (Fig 2, Fig 3, Fig 8A and S27 Fig). Additional lines of evidence reported here support the idea that pericentromeric and subtelomeric regions are centers of gene innovation in *T*. *thermophila*.

1. Genes within MIC chromosome arms have significantly lower Ka/Ks ratios than those within MIC pericentromeric and subtelomeric regions (Fig 3 and S34 Fig), suggesting a higher concentration of rapidly evolving genes in the latter regions.
2. The proportion of tandemly duplicated genes is higher in pericentric and subtelomeric regions than chromosome arms.
3. Most telling, the youngest LRR genes are concentrated in pericentromeric regions, while the oldest are concentrated in chromosome arms. This finding rules out the alternative possibility that MIC chromosome arms are the centers of innovation and new genes are “swept” over time by some unknown mechanism towards centromeres and telomeres.

**Fig 9:**
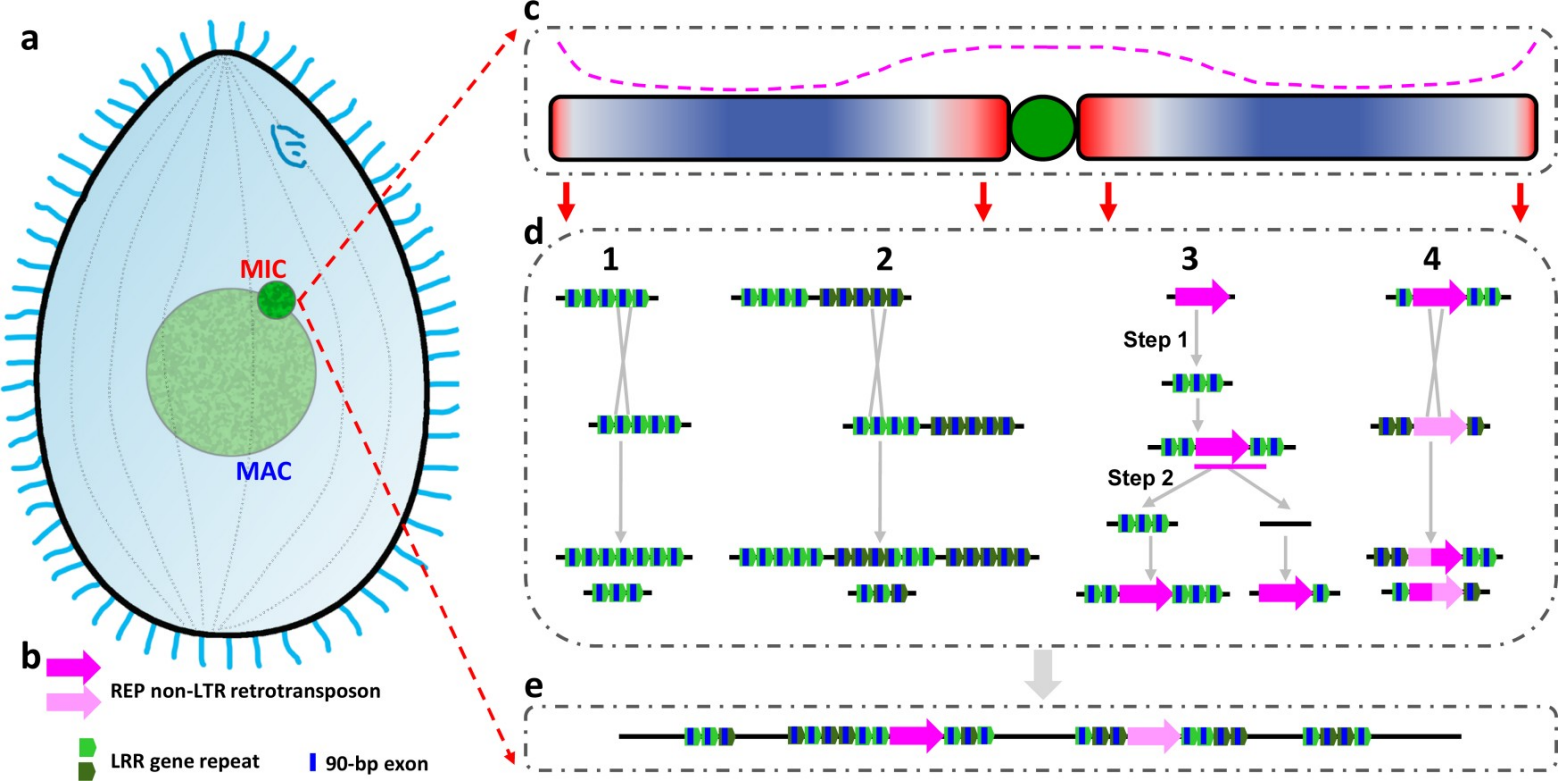
Evolutionary model of the observed innovation in LRR genes with tandem 90-bp exons. (a) Diagram of a *Tetrahymena* cell. (b) Key to LRR gene-related symbols. (c) Typical MIC chromosome showing the biased distribution of key genetic elements. Central green circle: centromere (not yet fully assembled and characterized). Red and blue shading: biased chromosomal distribution of youngest and most conserved genes, respectively. Darkest color: highest concentration. Pink dashed line above the chromosome: biased chromosomal distribution of TEs, *REP* included, and other repeated sequences. (d) Multiple exon-shuffling mechanisms proposed to explain how pericentromeric and subtelomeric regions of the MIC genome function as LRR gene innovation centers. (1) Unequal crossing over between 2 different exons of the same LRR gene leads to alleles with more and fewer tandem repeats. (2) Unequal crossing over between exons in 2 different LRR genes leads to exon duplications and deletions. (3) *REP* retrotransposition into a preexisting LRR gene (step 1), followed by possible (not yet demonstrated) *REP*-mediated retrotransduction of LRR gene repeats into another LRR gene (step 2) would lead to a net increase in number of LRR gene repeats. Pink line: transcript resulting from cotranscription of *REP* and 1 LRR gene repeat. Note that the right branch of represents a co-retrotransposition of REP and an LRR repeat, which lead to dispersal of the LRR repeats and could potentially mediate further ectopic recombinations. (4) *REP* copies, also being repeated sequences, can undergo unequal crossing over, with similar consequences as mechanism 2. (e) Representative product of the above mechanisms: LRR gene with long tandem arrays of 90-bp exons. LRR, leucine-rich repeat; MAC, macronucleus; MIC, micronucleus; non-LTR, Non-long terminal repeat; *REP*, REP-type retrotransposon; TE, transposable element.

For these reasons, we conclude that pericentric and subtelomeric regions of *T*. *thermophila* MIC chromosomes are the main centers of gene innovation (Fig 9). We expect the same to be true for the other *Tetrahymena* species because the evolutionary observations reported in this article seem to be so conserved in all of them.

Because essential and conserved genes are preferentially located on germline chromosome arms, the segregation of genome innovation to pericentromeric and subtelomeric regions may effectively physically insulate the organism’s need for genome stability from the uncertainty of genome innovation.

### The uniqueness of 90-bp exons and phase 2 introns of *Tetrahymena* LRR genes

The primary function of the LRR domain is as a protein recognition motif [30, 60]. LRR genes are widely distributed among eukaryotes [61] and often highly abundant [62–66], especially in plants (e.g., the apple genome has more than 1,000 LRR genes [67]). We report here that most *Tetrahymena* species have >1,000 LRR genes, and some of them have almost 2,000. The great abundance of *Tetrahymena* LRR genes and their continuous, species-specific diversification suggest that they may serve important evolutionary functions.

The exactly 90-bp exon peak (Fig 4) of LRR genes present in all *Tetrahymena* species investigated here is a remarkable feature of this gene family. Equally remarkable is that more than 96% of the approximately 72,000 90-bp exons are flanked by phase 2 introns on both ends (Fig 4C and S9 Fig). Thus, 3 consecutive 90-bp exons must contribute to the translation of one LRR 30–amino acid repeat unit. Tandem repeats have contributed to gene diversity in many eukaryotes, including primates, insects, fungi, and dinoflagellates [68–71]; however, we are unaware of tandem repeats in any other eukaryote with such an exceptional uniformity of exon and intron features. These unique features suggest that the exactly 90-bp–exon LRR genes with phase 2 introns may represent a novel category of genes that have evolved only once: in the *Tetrahymena* lineage.

### The special association between the non-LTR *REP* retrotransposon and LRR gene introns

The preferential association of the *REP* retroelement and LRR gene introns described in the Results section is very striking: 76% of Tt.*REPs* colocalize with LRR intron/exon repeat units (Fig 8). Furthermore, LRR repeat units are the most common element flanking Tt.*REPs*, (Fig 8, S31 Fig). Some of these nearby LRR repeat units belong to functional protein-coding genes (Fig 8B). Indeed, we have reported here cases in which remnants of the *REP* element are inserted in the middle of a functional LRR gene (S31 Fig). In *T*. *malaccensis*, with a more complete assembly of repeated element sequence, 86% of functional Tm.*REPs* are flanked by LRR 90-bp intron/exon units (S33 Fig). These results show, at a minimum, that LRR gene introns are favored *REP* transposition targets, a possibility first glimpsed by Fillingham and colleagues [33]. The above observations also raise the possibility of *REP*-mediated co-retrotransduction of LRR intron/exon repeats. In fact, we can assemble some transcript fragments including adjacent regions of LRR gene repeats and *REP* DNA in developing MACs deficient in the RNA interference (RNAi) silencing pathway (S35 Fig); this result indicates the possibility of co-transcription and co-retrotransposition of LRR gene repeats and adjacent *REP* DNA. In other eukaryotes, such events often come to attention when paralogs are found in the genome that have lost their introns because they are spliced out prior to retrotranscription. In the case of the *Tetrahymena* LRR genes, this would result in fused 90-bp LRR exons. We searched the *T*. *thermophila* MAC genome and found only one possible case of fusion of 2 LRR exons, despite our efficient means of detection. Thus, if co-retrotransposition between LRR genes and *REP* elements really occurred, the introns of LRR genes may fail to be spliced out. However, more evidence is needed to support this hypothesis.

### *Tetrahymena* LRR genes are a remarkable example of “exon shuffling”

Exon shuffling is a phenomenon whereby 2 or more exons from different genes are joined, or the same intron/exon unit is duplicated [31]. Exon shuffling is a major mechanism by which new genes are created [31]. Well-studied examples include the *Jingwei* gene in *Drosophila* [72] and LRR-containing pathogen receptors in plant [73]. The existence of thousands of *Tetrahymena* LRR genes with tens of thousands of exactly 90-bp exons and phase 2 introns is strong evidence for massive exon shuffling.

Our evidence implicates 2 types of MIC mechanisms as potential factors in LRR gene innovation by exon shuffling: ectopic recombination and retrotransposition. Ectopic recombination (i.e., unequal crossing over) is generally considered to be the most common way of creating new genes by tandem duplication [31, 32]. Cases of tandem gene duplication were previously reported in the *T*. *thermophila* MAC genome [19], and we describe here similar observations in the MAC genomes of 9 other *Tetrahymena* species. In the case of *Tetrahymena* LRR genes, MIC ectopic recombination between different repeats in the same LRR gene or between different LRR genes (Fig 7, S25 Fig, S26 Fig, Fig 9D1 and 9D2), either during meiosis or during asexual MIC mitosis, has likely resulted in the creation of LRR genes with more and fewer combinations of repeats (exon shuffling) and genomes with more and fewer LRR genes. Subsequent natural selection and genetic drift would result in the differential survival of genomes with more LRR genes and alleles with more repeats.

Several observations suggest that the *REP* retrotransposon could represent a second mechanism that may have caused ectopic recombination between LRR genes, although the phenomenon remains to be demonstrated. *Tetrahymena REP* non-LTR retrotransposon copies, as other TEs, are restricted to the MIC and are preferentially associated with pericentromeric and subtelomeric regions of MIC chromosomes (Fig 8A), just as the group III species-specific LRR genes. At a finer scale, *Tetrahymena REP*s are preferentially found next to LRR gene repeats, as described in the previous section. Retrotransposition of *REP* to introns of preexisting LRR genes (Fig 9D3, step1)—followed by the possible *REP*-mediated retrotransduction of LRR repeats to some other LRR gene—would lead to a net increase in the number of repeats in the target LRR gene (Fig 9D3, step2, left branch). A similar kind of exon shuffling has been reported for the human non-LTR retrotransposon LINE1, which retrotransduces DNA sequences flanking its 3' end into transcribed genes [74]. In addition, possible *REP-*mediated co-retrotransposition to a non-LRR intron location potentially leads to the dispersion of LRR repeats in the MIC genome (Fig 9D3, step2, right branch). Furthermore, *REP*s are repeated sequences, themselves capable of undergoing ectopic recombination with one another and thus generating novel LRR genes (Fig 9D4).

Finally, we have reported here that some *Tetrahymena* LRR gene repeats containing exactly 90-bp exons are fused with other domains, including PK (S36A Fig). We conclude that exon shuffling has likely also occurred between LRR gene repeats and other protein domains to generate genes with novel functions.

In summary, during *Tetrahymena* evolution, it is very likely that exon shuffling, mediated by the concerted action of unequal crossing over, *REP-*mediated ectopic recombination, and possibly co-retrotransduction, is largely responsible for the origin, dispersion, expansion, and intragenic structure of LRR genes containing unique 90-bp exon arrays (Fig 9).

### An intron length cycle revealed by *Tetrahymena* LRR 90-bp exon genes

We have reported above that cohorts of younger LRR genes have longer than average introns, with lengths inversely related to gene age, while the introns of the oldest LRR genes are approximately 50 bp—the genome background intron length. These observations suggest the existence of a *Tetrahymena* LRR “intron length cycle.”

We propose the following possible scenario. A wave of *REP* retrotranspositions into introns within LRR genes containing 90-bp exon/intron repeat units leads to a cohort of *REP*-containing LRR genes of the same age. Because the *REP* DNA is excised as an IES during MAC development, the insertion initially affects neither the length of the introns in the MAC nor the expression of the host genes in the cohort. With time, random mutations inactivate the *REP* elements of the cohort, but the *REP*-derived DNA is still recognized as an IES and excised during MAC development. This stage is consistent with our finding that many LRR gene introns in the MIC contain IESs (Fig 8B and S31 Fig).

As inactive *REP* copies continue to mutate, the length of *REP* remnants recognized and excised as IES gradually gets smaller, and the intron therefore gets correspondingly longer because DNA previously excised as IES has become additional intron DNA. Because of the randomness of those mutations, the cohort’s peak in the intron length distribution slowly becomes broader and shallower. The long-term tendency of all *Tetrahymena* introns to shorten ultimately drives the cohort mean intron length back down to the approximately 50 bp genome background limit, and the cohort ceases to form a distinct peak. The cycle would operate identically if any of the *REP* transposition events also resulted in co-retrotransduction of LRR intron/exon repeats.

We can document this natural LRR intron length cycle because we can distinguish older from younger *Tetrahymena* LRR genes and detect the role played by the *REP* retrotransposon in their evolution. The waves of events that periodically restart the intron length cycle are likely to occur most frequently in pericentromeric and subtelomeric regions of MIC chromosomes, where active TEs—*REP* included—are concentrated. The intron length cycle may well apply to other genes, where age dependence of intron length may be more difficult to document than in the LRR genes.

### On the function of the unique LRR genes in *Tetrahymena*

*Tetrahymena* species have more LRR genes than other eukaryotes. For example, whereas the human genome has <400 LRR genes [64], most *Tetrahymena* species have >1,000 LRR genes, and some (*T*. *paravorax*, *T*. *pyriformis*, *T*. *malaccensis*, and *T*. *thermophila*) have almost 2,000. These LRR genes have intact open reading frames, and RNA-seq data show that many are expressed genes under standard laboratory culture conditions. Furthermore, 9 of the 10 species have continued to generate LRR genes with tandem arrays of exactly 90-bp exons, including the earliest diverged *T*. *paravorax*. The mosquito parasite, *T*. *empidokyrea*, is the only *Tetrahymena* species in which the continued creation of LRR genes stopped—but only after group II LRR genes had evolved (S18 Fig). Significantly, Ka/Ks values indicate that most 90-bp exon LRR genes have undergone purifying selection, indicative of the continued evolutionarily importance of their function. Thus, the independent retention and continued evolution of 90-bp exon LRR genes in all the *Tetrahymena* species suggest a response to similar types of ever-present evolutionary challenges.

In plants, LRR genes are used for bacterial pathogen recognition—a type of innate immunity ([75] and S36B Fig). Such genes include LRR receptor kinase genes (e.g., *Xa21* [76], signal peptide + LRR domain + coiled-coil + PK domain), LRR genes (e.g. *Xa21D* [77], with only LRR domain), and nucleotide-binding site (NBS)-LRR genes (P-loop NTPase domain + LRR domain) [78]. In all 10 *Tetrahymena* species, domain compositions of species-specific genes show that not only LRR but also PKs and P-loop NTPase domain genes have been extensively expanded (S36A Fig). These expansions suggest that at least some *Tetrahymena* LRR genes could be involved in bacteria recognition-like processes. Indeed, the extensive diversity of *Tetrahymena* LRR genes hints at providing responsiveness to unpredictable challenges of a biological nature and is reminiscent of other well-known combinatorial immunity mechanisms, such as bacterial clustered regularly interspaced short palindromic repeats (CRISPR) systems to respond to viral infection [79] and vertebrate and plant systems of adaptive immunity [75, 76].

In addition, *Tetrahymena* cells experience special environmental challenges because of their free-living, opportunistic grazer lifestyle. They live in fresh water and have diverse food sources—especially bacteria, the oral apparatus of which is specially adapted to ingest—whose relative availability and diversity can be quickly altered by environmental changes. Furthermore, the freshwater environment is one that is subject to more frequent and pronounced variation in physical and chemical conditions than the marine environment in which *Tetrahymena*’s ancestors originated. Therefore, it seems reasonable to propose that *Tetrahymena* has evolved many different genes to specifically adjust to frequent environmental change, including the sensing of different bacterial foods and their digestion products [80–82]. Most *Tetrahymena* LRR genes do not include transmembrane domains, and many LRR proteins may be restricted to the cytoplasmic cell domain. If so, some could also function as a defense mechanism to neutralize noxious digestion byproducts unpredictably generated by opportunistic feeding on different types of bacteria. Additional experimental studies will be needed to test these hypotheses.

## Materials and methods

Additional method details are given in S1 Text.

### Cell strains, growth conditions, and morphological analysis

*T*. *thermophila* MAC and MIC genomes were previously sequenced and are available at the TGD Wiki (http://ciliate.org). Another 9 *Tetrahymena* species (*T*. *malaccensis*, *T*. *elliotti*, *T*. *pyriformis*, *T*. *vorax*, *T*. *borealis*, *T*. *canadensis*, *T*. *empidokyrea*, *T*. *shanghaiensis*, and *T*. *paravorax*) were newly sequenced in this study. These species were obtained from either the American Type Culture Collection (ATCC) or the *Tetrahymena* Stock Center (S6 Table). Cells were grown in SPP medium and harvested at a density of 150,000 to 250,000 cells/ml. The morphology of *Tetrahymena* cells was analyzed using the silver carbonate impregnation method [83].

### Genome sequencing, genome assembly, and gene annotation

MAC genomes of the 9 *Tetrahymena* species were sequenced using the Illumina platform. Three of them (*T*. *malaccensis*, *T*. *elliotti*, and *T*. *borealis*) were sequenced by the Broad Institute in collaboration with the J. Craig Venter Institute, and the rest were sequenced by Institute of Hydrobiology, Chinese Academy of Sciences. In general, paired-end (insert size 180–220 bp) and mate-pair (insert size 2–3 Kb) libraries were constructed and sequenced using standard Illumina protocols. The germline MIC genome of *T*. *malaccensis* was sequenced using PacBio Sequel platform. For RNA-Seq, Poly-A mRNAs were isolated and sequenced using standard Illumina protocols.

MAC genomes of each species were assembled using SOAPdenovo [84] or ALLPATHS-LG [85]. The assembly with the longest N50 length was selected, and scaffolds with length <1 Kb were excluded. The *T*. *malaccensis* MIC genome was assembled using SMRTLINK_V5 (HGAP4, https://www.pacb.com/support/software-downloads/). Repeat consensus sequences were de novo identified and annotated using RepeatModeler (http://www.repeatmasker.org/RepeatModeler/) with default settings, and the output consensus sequence library of RepeatModeler was used to mask the genomes using RepeatMasker (-div 20).

Gene models were predicted using ab initio–based and homology-based methods (S37 Fig). RNA-Seq data for logarithmic growth stage in all 9 newly sequenced species were generated to aid gene prediction. Finally, a set of gene models was created using Evidence Modeler [86] by merging all predicted gene models and RNA-Seq transcripts, followed by manual correction.

### Ortholog group identification

Ortholog groups (clusters) were annotated using OrthoMCL [23], which provides the best overall balance of sensitivity and specificity for multiple species ortholog clustering [87]. The important parameter inflation index of OrthoMCL was set at 1.5 to balance sensitivity and selectivity, as used in OrthoMCL-DB construction [88]. OrthoMCL groups genes in different species into clusters (ortholog groups). These ortholog groups were classified into 10 categories according to their presence in different species (Category I–X, see S2 Fig). Genes were classified as singletons if they could not be assigned to any ortholog group by OrthoMCL. Note that the number of genes in an ortholog group may vary among species because of the presence of inparalogs and co-orthologs; therefore, statistical analyses were performed to determine whether inparalogs had undergone significant expansion (such inparalogs can also be species specific, also see S1 Text). We identified ortholog groups that show significant inparalog expansion in one species because they are usually species-specific genes (S38 Fig). Species-specific genes include singletons, category I, and members of significantly expanded ortholog group genes.

### Phylogenomic analysis and divergence time estimation

A total of 198 one-to-one ortholog groups were identified in all 10 *Tetrahymena* species and other ciliates (*I*. *multifiliis*, *P*. *persalinus*, *P*. *tetraurelia*, *O*. *trifallax*, and *S*. *mytilus*). A maximum-likelihood tree was constructed using RAxML [89]. The divergence time for *Tetrahymena* species was estimated using r8s [90] with the divergence time for *Ichthyophthirius* and *Tetrahymena* as the approximate calibration time point. This divergence time is about 447 Mya [91] based on a phylogenomics study in ciliates incorporating a ciliate fossil record-based estimate reported by Weitschat and colleagues [92].

### Ka/Ks analysis

Pairwise alignments of protein sequences were performed for gene pairs in each OrthoMCL ortholog group using MUSCLE [93], and pairwise nucleotide sequence alignments were generated by transforming protein alignments into codon alignments in RevTrans [94]. Ka/Ks ratios were calculated based on pairwise codon alignments using PAML (runmode = −2) [95].

### Gene expression analysis

For gene expression analysis, RNA-Seq data, generated from cells during the vegetative growth stage, were mapped to assembled genomes using TopHat [96]. Gene expression level was estimated using uniquely mapped reads based on predicted gene models and expressed as fragments per kilobase of exon per million reads mapped (FPKM) values.

### Sequence logos and phylogenetic tree of consensus sequences of 90-bp exons

The vast majority of the 90-bp exons are in the same phase. The sequences of 90-bp exons are naturally aligned, because all of them are exactly 90 bp in length. Because there is extreme phase 2 intron bias in consecutive 90-bp exons of LRR genes, a 90-bp exon can be translated into 29 amino acids after excluding the first base pair at the 5′ end and the last 2 base pairs at the 3′ end. Nucleotide sequence logos were generated using the sequences of 90-bp exons plus, as anchors, the GT-AG intronic splicing signals. Amino acid sequence logos were generated based on the 29 amino acids translated from 90-bp exons. LRR genes containing 90-bp exons were sorted into 2 groups, group II and group III (see Results section). For group III LRR genes, we further divided them into different groups based on the masking of CRSs identified by RepeatModeler. Each LRR gene in group III was exclusively assigned into a CRS group based on the highest masking score. Then, we compared the sequence logo difference between either group II and III or different CRS groups. For each group (group II and group III, or CRS groups), sequences of all 90-bp exons in all LRR genes were extracted, which are naturally aligned because they are identical in length. These alignments were used to generate sequence logos using WebLogo. Sequence logo profile comparisons between *T*. *paravorax* and the other 9 species were performed by the Two Sample Logo tool (http://www.twosamplelogo.org) [97] (binomial test, *p*-value cutoff: 1 × 10^−5^).

To investigate the relationships of CRS groups (note that CRS groups only exist in group III LRR genes) in different species, the sequences of all 90-bp exons in all LRR genes of each CRS group were extracted. Because there are more than 45,000 exons of 90 bp, it is hard to interpret such a large tree. Therefore, we generated a 90-bp consensus sequence for all the 90-bp exons in each CRS group and then constructed a phylogenetic tree based on the consensus sequences (90 bp in length) of all CRS groups.

## Supporting information

S1 Fig *Tetrahymena* species show high genome diversity. Heat map and matrix showing the percentage of homologous regions among all 10 genomes. Pairwise genome alignment was performed using promer in MUMmer 3 software (http://mummer.sourceforge.net/) with default settings, and only one-to-one alignments were used to identify homologous regions. Note that the difference between the percentages above and below the diagonal for the same species pair is due to the different genome sizes of the 2 species used as denominators when calculating the percentages.
(TIF)

S2 Fig Species-specific genes in all *Tetrahymena* species are enriched for the same subset of protein domains. (a) Ortholog group (cluster) distributions for all 10 *Tetrahymena* species. A defined ortholog group contains at least 2 genes in either the same or different species (also see S39 Fig). Genes that could not be assigned to any ortholog group are defined as singletons. Ten categories of ortholog groups (roman numerals I–X) are defined based on the number of different species represented in the ortholog group, e.g., genes appearing in only 1 species are assigned to category I, etc. Red dotted box: singletons and category I genes; blue dashed box: genes in ortholog groups identified in at least 2 species (categories II–X). Numbers in bold in each cell indicate the number of genes; numbers in parenthesis indicate the number of ortholog groups. (b) Distribution of genes encoding the top 7 protein domains, which are the same for every species. Left: singletons or category I genes. Right: genes in significantly expanded ortholog groups in some species. The number of genes encoding each type of protein domain was used to generate the heat maps.
(TIF)

S3 Fig *Tetrahymena* species-specific genes have higher tandem duplication frequencies. For category I–X, the percentage of tandemly duplicated genes was calculated based on tandem inparalogs in each gene cluster (OrthoMCL ortholog group). For singletons, which have no inparalogs, the tandem arrangements of genes were used to calculate the percentage regardless of whether they included inparalogs. Numerical data underlying this figure are listed in S2 Data.
(TIF)

S4 Fig The largest tandem duplicated inparalogs cluster in *Tetrahymena*. The synteny map of homologous MAC scaffolds for 10 species shows the largest cluster of tandem duplicated inparalogs. For each species, blue bars above the horizontal black line represent genes transcribed in the forward direction, and blue bars under the horizontal black line represent genes transcribed in the reverse direction. Orange lines between maps of different species represent the one-to-one ortholog relationships among the 10 species. The red bars under the central black line of the *T*. *pyriformis* map represent the largest tandem duplicated gene cluster among the 10 species, containing 17 strict tandem LRR inparalogs. This cluster is extended to 65 LRR inparalogs if 2 inparalogs are allowed to be separated by up to 3 other unrelated genes. The dashed arrow points to an expanded view of this cluster. The synteny map indicates that this tandem inparalog cluster specifically arose in *T*. *pyriformis*.
(TIF)

S5 Fig Case showing a *T. malaccensis*-specific tandemly duplicated inparalog cluster with 5 LRR genes. The synteny map of a MAC chromosome for 10 species shows a *T*. *malaccensis*-specific tandem duplicated LRR gene cluster. Symbols are as in S4 Fig. The red bars under the *T*. *malaccensis* horizontal black line, and the enlarged diagram at the bottom shows a tandem duplicated gene cluster containing 5 LRR genes. LRR, leucine-rich repeat; MAC, macronucleus.
(TIF)

S6 Fig Rapid evolution of *Tetrahymena* species-specific genes. (a) The Ka/Ks ratio distribution for all 10 categories of ortholog groups. All Ka/Ks ratios for each ortholog group were used to generate the box plot (Numerical data underlying this panel can be accessed at http://ciliate.ihb.ac.cn/tcgd/download.html). (b) The Ka/Ks ratio distribution for all 7 protein domain groups and all 10 ortholog categories. The median Ka/Ks ratio for every ortholog category was used. Numerical data underlying this panel are listed in S2 Data.
(TIF)

S7 Fig Low expression of species-specific genes in SPP medium in *Tetrahymena*. (a) Gene expression level differences between conserved (left bar) and species-specific (right bar) genes in each species. The box plot was generated using the expression values (FPKMs) of all conserved genes (left) and species-specific (right) genes. Key to the colors representing various species is shown in panel b. Two asterisks indicate that there is significant difference (Mann Whitney U test, *p* < 0.01) between the expression of conserved and species-specific genes. (b) Expression levels for genes for all species in all 10 categories of ortholog groups. Note that the median FPKM value for each category is plotted in each panel. Gene expression levels were measured in vegetatively multiplying cells. Numerical data underlying this figure are listed in S2 Data. FPKM, fragments per kilobase of exon per million reads mapped; SPP, super protease peptone.
(TIF)

S8 Fig Characteristic features of *Tetrahymena* LRR genes. (a) Most 90-bp exon-containing genes are LRR genes and vice versa; (b) RNA-Seq evidence supports the presence of 90-bp exon arrays in LRR genes (using gene TTHERM_01349950 as an example). (c) Most LRR genes contain 90-bp exons in *T*. *thermophila*. (d) Nearly half of *T*. *thermophila* LRR genes are masked by 8 MAC CRSs. CRS, consensus repeat sequence; LRR, leucine-rich repeat; MAC, macronucleus; RNA-Seq, RNA sequencing.
(TIF)

S9 Fig Extreme phase 2 bias of introns among group II and III LRR genes in 10 *Tetrahymena* species. The 10 concentric circles represent the 10 species, from inside to outside: *T*. *thermophila*, *T*. *malaccensis*, *T*. *elliotti*, *T*. *pyriformis*, *T*. *vorax*, *T*. *borealis*, *T*. *canadensis*, *T*. *empidokyrea*, *T*. *shanghaiensis*, and *T*. *paravorax*. (a) Phase distribution of introns in group I LRR genes. (b) Phase distribution of introns preceding non-LRR 90-bp exons. (c) Phase distribution of introns preceding 90-bp exons in group II LRR genes. (d) Phase distribution of introns preceding 90-bp exons in group III LRR genes. Note that *T*. *empidokyrea*, a mosquito parasite, lacks group III LRR genes. LRR, leucine-rich repeat.
(TIF)

S10 Fig Variation in the number of 90-bp exons among the 5 inparalog OrthoMCL clusters containing the largest numbers (83, 60, 29, 13, 12 genes) of group III LRR genes in *T. thermophila*. For each inparalog cluster, the box plot shows the variation of the number of 90-bp exons (y-axis) among different inparalog clusters, indicative of the diversity of gene structures (different numbers of 90-bp exons) found even among the most closely related group III LRR genes. Numerical data underlying this figure are listed in S2 Data. LRR, leucine-rich repeat.
(TIF)

S11 Fig Number of introns, GC content, and leucine codon usage of 3 groups of *T. thermophila* LRR genes. (a) Distributions of the number of introns per gene for the 3 groups of LRR genes. (b) GC content distributions for the 3 groups of LRR genes. (c) Leucine codon usage among LRR gene groups (differences are indicated with arrows). Numerical data underlying this figure are listed in S2 Data. LRR, leucine-rich repeat.
(TIF)

S12 Fig *P. tetraurelia* LRR genes are similar to *Tetrahymena* group I LRR genes. (a) *P*. *tetraurelia* LRR genes lack a prominent 90-bp exon peak. (b) No second intron length peak was observed for *P*. *tetraurelia* LRR genes. (c) *P*. *tetraurelia* LRR genes usually have no or few introns. This distribution is similar to that of all *P*. *tetraurelia* genes. LRR, leucine-rich repeat.
(TIF)

S13 Fig Intron length distributions for all 10 *Tetrahymena* species. The dashed circles indicate secondary peaks.
(TIF)

S14 Fig Differences between *Tetrahymena* group II and group III LRR genes. (a) Group II and group III LRR genes show different intron length distributions. Upper panels, 2 representative gene models illustrating intron length differences between group II and group III LRR genes; bottom panel, intron length distributions in all group II and group III LRR genes. (b) Intragenic repeats are more common in *Tetrahymena* group III than group II LRR genes. Dot plot self-alignments to detect nucleotide level intragenic repeats in the 2 representative LRR genes from panel a. Intragenic repeats are detected in the group III (right) but not in the group II (left) LRR gene. (c) Partial nucleotide sequence including 2 intragenic repeat units in the group III LRR gene in panel a. The start position (start site of a block in self-alignment) of a repeat unit is indicated by the numbers 1 and 2. Red characters highlight the 90-bp exon, and black characters represent the upstream and downstream intron sequences. Bold black characters represent the intron splice sites. LRR, leucine-rich repeat.
(TIF)

S15 Fig Comparison of 90-bp exon sequence logos between group II and III LRR genes of all 10 species. “AG” and “GT” at the left and right ends, respectively, are the intron splice sites that flank the 90-bp exons. The conserved leucine codon locations in both groups are centered at exon nucleotide positions 36, 48, 66, 75, 81, and 87. All logos were generated using WebLogo (http://weblogo.berkeley.edu/logo.cgi). LRR, leucine-rich repeat.
(TIF)

S16 Fig Alignment of nucleotide sequence logos of every 90-bp exon of group II LRR genes for each of the 10 species. Plots are as described under S15 Fig. LRR, leucine-rich repeat.
(TIF)

S17 Fig Alignment of nucleotide sequence logos of every 90-bp exon of group III LRR genes for each of 9 species. Plots are as described under S15 Fig. Note that no logo is shown for *T*. *empidokyrea* because it lacks group III LRR genes. LRR, leucine-rich repeat.
(TIF)

S18 Fig Similar features of the 3 groups of LRR genes are shared by all the *Tetrahymena* species. Proportions of conserved and species-specific genes (numerical data are listed in S2 Data), exon length (bp), intron number, intron length (bp), GC content, and leucine codon usage (numerical data are listed in S2 Data) were compared between group I (blue), II (red), and III (green) LRR genes in all 10 *Tetrahymena* species. Note that *T*. *empidokyrea*, the parasitic species, contains no group III LRR genes. LRR, leucine-rich repeat.
(TIF)

S19 Fig Clonal origin and expansions of *Tetrahymena* 90-bp exon LRR genes. (a) comparison of 90-bp exon amino acid sequence logos of group II and III LRR genes of all 10 species. Because flanking introns have phase 2, the first nucleotide and the last two nucleotides of every exon contribute to the terminal codon of its upstream and downstream exons, respectively; therefore, only 29 amino acids are represented for each exon in all the amino acid sequence logos. (b) phylogenetic tree of consensus sequences of 90-bp exons masked by various MAC CRSs in all 10 species. For LRR genes masked by any given CRS, the consensus nucleotide sequence of all their 90-bp exons was used for the phylogenetic analysis. The clade which includes the *T*. *thermophila* CRS1-type 90-bp exons is shown in different color (green) to highlight that it clusters exons from distantly related species that likely underwent recent clonal expansions. (c) Comparison of 90-bp exon nucleotide sequence logos of *T*. *thermophila* LRR genes masked by either CRS1 (top) or all 7 other CRSs (bottom). Note that the nucleotide flips between 90-bp exons masked by CRS1 and all 7 other CRSs at position 34, 35, 37, 43, 51, 52, 68, and 80 (black arrows). CRS, consensus repeat sequence; LRR, leucine-rich repeat; MAC, macronucleus.
(TIF)

S20 Fig Alignment of amino acid sequence logos of every 90-bp exon of group II and III LRR genes for each of the 10 species. (a) Group II LRR genes. (b) Group III LRR genes. Note that *T*. *empidokyrea* is missing in panel b because it lacks group III exons. LRR, leucine-rich repeat.
(TIF)

S21 Fig Sequence logo comparison between *T. paravorax* and the other 9 species. Amino acid (a) and nucleotide (b) sequence logos of 90-bp exons were compared between *T*. *paravorax* and the other 9 species using Two Sample Logos (http://www.twosamplelogo.org) (binomial test, *p*-value cutoff: 1 × 10^−5^). For each comparison, symbols above the central bar represent enriched levels (*T*. *paravorax* versus others), and below the bar represents depleted levels (*T*. *paravorax* versus others). The red arrows indicate highly enriched amino acids or nucleotides in all species.
(TIF)

S22 Fig Intron length distributions of LRR genes masked by 8 *T. thermophila* CRSs. Each panel represents the introns masked by the indicated CRS. When a gene was masked by more than one CRS, it was assigned to only 1 of the 8 subgroups based on the highest masking score. CRS, consensus repeat sequence; LRR, leucine-rich repeat.
(TIF)

S23 Fig Alignment of nucleotide sequence logos of 90-bp exons of group III LRR genes best masked by each of the 8 different *T. thermophila* CRSs. Plots are as described under S15 Fig. When a gene was masked by more than one CRS, it was assigned to only 1 of the 8 subgroups based on the highest masking score. CRS, consensus repeat sequence; LRR, leucine-rich repeat.
(TIF)

S24 Fig Phylogenetic tree illustrating extensive ectopic recombination of 90-bp exons from different inparalogs. Exons of 90 bp from different inparalogs of the largest gene cluster (cluster 1 in S10 Fig) in group III LRR genes were selected for phylogenetic analysis. This gene cluster contains 83 inparalogs and a total of 1,932 exons of 90 bp. The same color was used for all the 90-bp exons from the same inparalog (total 83 different colors). Bottom left: zoom-in view of a representative clade encompassed by the curved, thick black bar. The enlarged clade illustrates more clearly the close clustering of leafs with different colors (different inparalogs), indicative of the extensive recombination of closely related 90-bp exons in different inparalogs. The size of grey dots indicate the support values of phylogenetic tree. LRR, leucine-rich repeat.
(TIF)

S25 Fig Phylogenetic network analysis of 12 nearly identical 90-bp exons. Unlike simple bifurcating trees, phylogenetic networks indicate multiple pathways of descent, e.g., as the result of recombination, which can be recognized by closed rectangles in the graph. The phylogenetic network of 12 nearly identical 90-bp exons is shown as green edges, and bootstrap supporting values are labeled. At the end of each branch are intron/exon diagrams of the 10 LRR genes containing the twelve 90-bp exons (shown in red) that share between 88 and 90 identical nucleotides. Identical 90-bp exons are indicated by yellow asterisk (*) or number sign (#). Listed above each gene: MIC chromosome location. L or R indicates the left or right arm. “pCen” indicates the pericentromeric region; “mid-arm” indicates near the middle of chromosome arms; and “NA” indicates not available because the gene is located in a still unassembled region. Note that (1) This is the largest group of nearly identical 90-bp exons and (2) TTHERM_001443819 and TTHERM_00001659049 both have 2 exons that belong to this group. The above phylogenetic network was constructed using SplitsTree4 (https://ab.inf.uni-tuebingen.de/software/splitstree4) based on the Ucorrected_P-NeighborNet-EqualAngle pipeline with default settings and 1,000 bootstraps. The phylogenetic network defines each nonconstant column in the sequence alignment as a so-called split and generates a phylogenetic graph of the split network by integrating all compatible and incompatible splits, and thus the phylogenetic network gives all the possible relationships of 90-bp exons compared to a simple phylogenetic tree. LRR, leucine-rich repeat; MIC, micronucleus.
(TIF)

S26 Fig Phylogenetic network analysis supports the close relationship of 12 nearly identical 90-bp exons. The phylogenetic network was constructed as in S25 Fig. In this type of representation, every 90-bp exon of the 10 genes containing 12 nearly identical 90-bp exons is individually shown; 90-bp exons from the same gene are shown as branches with the same color. A clade containing the 12 nearly identical 90-bp exons is highlighted with a black circular arc. Rectangles are evidence of recombination, as in S25 Fig.
(TIF)

S27 Fig Distribution of *T. thermophila* IES and repetitive sequence in the 5 MIC chromosomes. The MAC-destined components of the MIC chromosomes were divided into 1 Mb bins, and the percentages (y-axis) of IES sequences (left) and repetitive sequences (right) were plotted. The repetitive sequences, including TEs, were retrieved from the masking results using the MIC consensus sequence library in *T*. *thermophila* reported by Hamilton and colleagues [20]. IES, internal eliminated sequence; MAC, macronucleus; MIC, micronucleus; TE, transposable element.
(TIF)

S28 Fig Top domain categories in IES-flanking or IES-containing genes in the *T. thermophila* MIC. For each IES, domain(s) in the nearest genes on either side, or in the gene containing the IES (within an intron) were counted. Two asterisks indicate significant enrichment of IES-associated genes (chi-squared test, *p* < 0.01). Numerical data underlying this figure are listed in S2 Data. IES, internal eliminated sequence; MIC, micronucleus.
(TIF)

S29 Fig tLRR-MIC-CRS is by far the MIC CRS that masks most MIC MDSs in *T. thermophila*. The number of MIC (red bars) and MAC (blue bars) genome loci masked by members of the MIC CRS library were sorted by the number of masked loci in MDS. Green arrow indicates sequences masked by tLRR-MIC-CRS. Numerical data underlying this figure are listed in S2 Data. CRS, consensus repeat sequence; MAC, macronucleus; MDS, MAC-destined sequence; MIC, micronucleus.
(TIF)

S30 Fig *T. thermophila* MAC CRS6 includes a conserved 54-bp Tt.*REP* element. (a) Structure of a functional *T*. *thermophila* Tt.*REP*. It contains 2 genes named ORF1 and ORF2. ORF1 encodes a potential zinc finger protein, and ORF2 encodes a protein with both reverse transcriptase and endonuclease domains. It also contains a conserved 3′ 54-bp “tail” (red arrow) [33]. (b) Dot plot showing nucleotide sequence matches between CRS6 (x-axis) and, in the y-axis, either an LRR repeat unit (green dots) or Tt.*REP2* (pink dots). (c) Enlargement of the particular intron showing the perfect match of CRS6 to the conserved *REP* 54-bp tail, presumably a remnant of an originally functional *REP* copy inserted within an LRR gene intron. Since CRS6 is a *consensus* sequence, this match implies a clade of LRR introns having copies of the *REP* remnant. Sequence alignments and dot plots were generated using YASS (http://bioinfo.lifl.fr/yass/yass.php). CRS, consensus repeat sequence; LRR, leucine-rich repeat; MAC, macronucleus.
(TIF)

S31 Fig Two examples of *REP* element remnants embedded within introns of functional *T. thermophila* LRR genes. (a) Gene TTHERM_001119553; (b) gene TTHERM_001023110. For each panel: top level: MIC DNA segment: IES (red) and MDS (blue). Second level: gene model. Dark boxes: 90-bp exons. Angle brackets: introns. The longest angle bracket includes intron MDS sequence, and *REP* remnant sequence that will be excised as IES during MAC development. Third level: green: tLRR-MIC-CRS-masked segment; pink: *REP* element remnant, including its conserved 54-bp “tail” (green arrow). Fourth level: RNA-Seq evidence. CRS, consensus repeat sequence; LRR, leucine-rich repeat; MAC, macronucleus; MDS, MAC-destined sequence; MIC, micronucleus; RNA-Seq, RNA sequencing.
(TIF)

S32 Fig tLRR-MIC-CRS-masked sequences co-expanded with Tt.*REP* copies in the *T. thermophila* MIC. (a) Phylogenetic tree of homologs of Tt.*REP2* identified through a BLAST search. Tt.*REP2* is a previously identified, functional copy of the *T*. *thermophila* non-LTR *REP* retrotransposon [33]. MIC genome supercontig coordinates are shown for each copy. Tt.*REP* copies potentially representing the most recent retro-transposition events are colored pink. (b) Examples illustrating in detail the physical relationship between 3 Tt.*REP* copies highlighted in (a) and tLRR-MIC-CRS-masked sequences in the MIC genome. Note that Tt.*REP* copy at supercontig location 2.734:15527..17709 is not included in panel b because is at the end of a small, incomplete scaffold, whose flanking sequence, likely repetitive, remains unassembled. CRS, consensus repeat sequence; LRR, leucine-rich repeat; non-LTR, Non-long terminal repeat; MIC, micronucleus.
(TIF)

S33 Fig *T*. *malaccensis* tLRR-MIC-CRS-masked LRR repeats are most often flanked by non-LTR *REP* retrotransposons (Tm.*REPs*). Green bar, tLRR-MIC-CRS-masked loci; black bar, loci masked by other repeat families or low complexity sequences, listed in order of incidence. A Tm.*REP* copy is considered functional if it contains both intact ORF1 and ORF2 and nonfunctional if it lacks one or both intact ORFs. Numerical data underlying this figure are listed in S2 Data. CRS, consensus repeat sequence; LRR, leucine-rich repeat; MIC, micronucleus.
(TIF)

S34 Fig Ka/Ks ratios of 6,052 one-to-one orthologs in 5 MIC chromosomes of *T. thermophila*. MAC-destined DNA of MIC chromosomes was divided into approximately 1 Mb bins. (a) Density distribution of the 6,052 one-to-one orthologs; y-axis is the number of genes. (d) Distribution of Ka/Ks ratios of 6,052 one-to-one orthologs, plotted as median value of each bin. The Ka/Ks were calculated using codeml (runmode = 0) in PAML (maximum likelihood method) based on the phylogenomic tree in Fig 1. Significant higher Ka/Ks values were found between pericentromeric bins and middle arm bins in chr1, 2, and 4 (Mann Whitney U test, *p* < 0.01). MAC, macronucleus; MIC, micronucleus; PAML, phylogenetic analysis by maximum likelihood.
(TIF)

S35 Fig RNA-Seq evidence for co-transcription of Tt.*REP* and LRR repeats in *T*. *thermophila*. (a) Some de novo–assembled transcript fragments show sequence identity to both Tt.*REP* and tLRR-MIC-CRS-masked sequences (containing LRR repeats) in cells deficient in the RNAi (Δ*DCL1*) or Polycomb (Δ*EZL1*) repression pathways. These pathways are required for the transcriptional silencing—and ultimate excision—of TEs and other IESs during MAC development in *T*. *thermophila*. (b) RNA-Seq evidence for tLRR-MIC-CRS-masked sequence and Tt.*REP* co-transcription in RNAi (Δ*DCL1*) and Polycomb repression (Δ*EZL1*) pathway-deficient conjugating cells. Note that the sequence reads are essentially absent when *REP* is silenced in wild-type conjugating cells. CRS, consensus repeat sequence; IES, internal eliminated sequence; LRR, leucine-rich repeat; MAC, macronucleus; MIC, micronucleus; RNAi, RNA interference; RNA-Seq, RNA sequencing; TE, transposable element.
(TIF)

S36 Fig Similar domain compositions of plant innate immunity related LRR genes and *Tetrahymena* species-specific genes. (a) Protein domain architecture for *Tetrahymena* species-specific genes, presented as a network. Each node represents a specific domain type, and lines represent links between 2 domains within a gene. The width of each line indicates the number, which is also written on the line. LRR, TPR, WD40, PK, CNBD, GFR, and P-loop NTPase domains are shown in different colors, and lines connecting the same color of node represent genes containing only this domain (e.g., an LRR–LRR connection indicates genes that only contain the LRR domain). Coiled-coil (Coil), transmembrane helix (Transmembrane), and signal peptide (Signal_peptide) structures are also included to illustrate that other domains are often associated with the 7 most frequent domains in species-specific genes. (b) Domain architectures of 2 innate immunity LRR genes previously reported in plants. *Xa21* (UniProt ID: Q1MX30) is a receptor kinase-like protein in *Oryza sativa subsp*. *Japonica*. NBS-LRR (represented as H9DWE0) is a class of proteins containing both P-loop NTPase and LRR domains in *O*. *sativa subsp*. *Indica*. CNBD, cyclic nucleotide-binding domain; GFR, growth factor receptor; LRR, leucine-rich repeat; P-loop NTPase, P-loop-containing nucleoside triphosphate hydrolase; PK, protein kinase; TPR, tetratricopeptide repeat.
(TIF)

S37 Fig Gene prediction pipeline. Both ab initio– and homology-based methods were used for gene prediction. Assembled RNA-Seq data were used to generate training gene sets for ab initio gene predictions and were also incorporated as cDNA evidence. EvidenceModeler was used to generate a set of gene models combining evidence from all gene prediction programs. Final predicted gene sets were generated after a few manual corrections. RNA-Seq, RNA sequencing.
(TIF)

S38 Fig Examples of species-specific expansion of inparalogs in OrthoMCL clusters. The number of inparalogs in each species is shown above the species name. (a) Category IX cluster 180 has undergone extensive expansion only in *T*. *paravorax* (29 genes). (b) Category II cluster 75 has undergone specific expansion only in *T*. *pyriformis* (61 genes).
(TIF)

S39 Fig Relationships among genes within an OrthoMCL ortholog group (related to S1 Text). OrthoMCL first identified reciprocal best blast hits between species (“true” orthologs: A1, B1, and C1) and then assigned, to the same ortholog group, genes that gave reciprocal better within-species blast hits to any of those genes (rest of the genes shown in the figure). Although OrthoMCL clusters are usually referred to as ortholog groups, pairs of genes within a cluster may be true orthologs (red arrows), inparalogs (if they are in the same species, blue arrow), or co-orthologs (if they are in different species, dotted black arrows). Orthologs are genes that are conserved between species. Inparalogs are members of a gene expansion within a certain species or lineage; they can be species-specific or very recently duplicated lineage-specific genes.
(TIF)

S1 Table Morphological characters of 10 *Tetrahymena* species. (DOCX)

S2 Table The 7 protein domains that have undergone the most extensive expansions in *Tetrahymena*. (DOCX)

S3 Table Intron phase among LRR genes with only one or two 90-bp exon(s). LRR, leucine-rich repeat.
(DOCX)

S4 Table Number of CRSs masking Group III LRR genes identified in the MAC genome of each species. CRS, consensus repeat sequence; LRR, leucine-rich repeat; MAC, macronucleus.
(DOCX)

S5 Table The most conserved amino acids of 90-bp exons in group II LRR genes in different species. LRR, leucine-rich repeat.
(DOCX)

S6 Table *Tetrahymena* strains used in this study and their source. (DOCX)

S7 Table Domain accessions used for LRR and PK gene identification (related to S1 Text). LRR, leucine-rich repeat; PK, protein kinase.
(DOCX)

S1 Text Supplementary methods. (DOCX)

S1 Data Ortholog groups generated by OrthoMCL. (ZIP)

S2 Data Individual numerical values that underlie the summary data displayed in the following figure panels: Fig 4D, Fig 8C, S3 Fig, S6B Fig, S7A Fig, S7B Fig, S10 Fig, S11A Fig, S11B Fig, S11C Fig, S18 Fig left and right panels, S28 Fig, S29 Fig, S33 Fig. (XLSX)

S3 Data CRS libraries generated by RepeatModeler in all 10 species. CRS, consensus repeat sequence.
(DOCX)

## Abbreviations

ATCC: American Type Culture Collection
CNBD: cyclic nucleotide-binding domain
CRISPR: clustered regularly interspaced short palindromic repeats
CRS: consensus repeat sequence
FPKM: fragments per kilobase of exon per million reads mapped
GC: Guanine and Cytosine
GFR: growth factor receptor
IES: Internal Eliminated Sequence
Ka/Ks: ratio of nonsynonymous to synonymous substitutions
LCA: last common ancestor
LINE: long interspersed element
LRR: leucine-rich repeat
MAC: macronucleus
MDS: MAC-destined sequence
MIC: micronucleus
Mya: million years ago
NBS: nucleotide-binding site
NCBI: National Center for Biotechnology Information
non-LTR: Non-long terminal repeat
P-Loop NTPase: P-loop-containing nucleoside triphosphate hydrolase
PK: protein kinase
rDNA: ribosomal DNA
RNAi: RNA interference
RNA-Seq: RNA sequencing
SMRT: single-molecule real-time
SPP: Super Proteose Peptone
TCGD: Tetrahymena Comparative Genomics Database
TE: transposable element
TGD: *Tetrahymena* Genome Database
Tm.REP: *T*. *malaccensis* REP retrotransposon
TPR: tetratricopeptide repeat
Tt.REP: *T*. *thermophila* REP retrotransposon
