# Supplementary figures and images for: Hidden genomic evolution in a morphospecies—The landscape of rapidly evolving genes in Tetrahymena
Source: PLoS Biol. 2019 Jun 3;17(6):e3000294. doi: 10.1371/journal.pbio.3000294 (PMC6564038; doi:10.1371/journal.pbio.3000294)

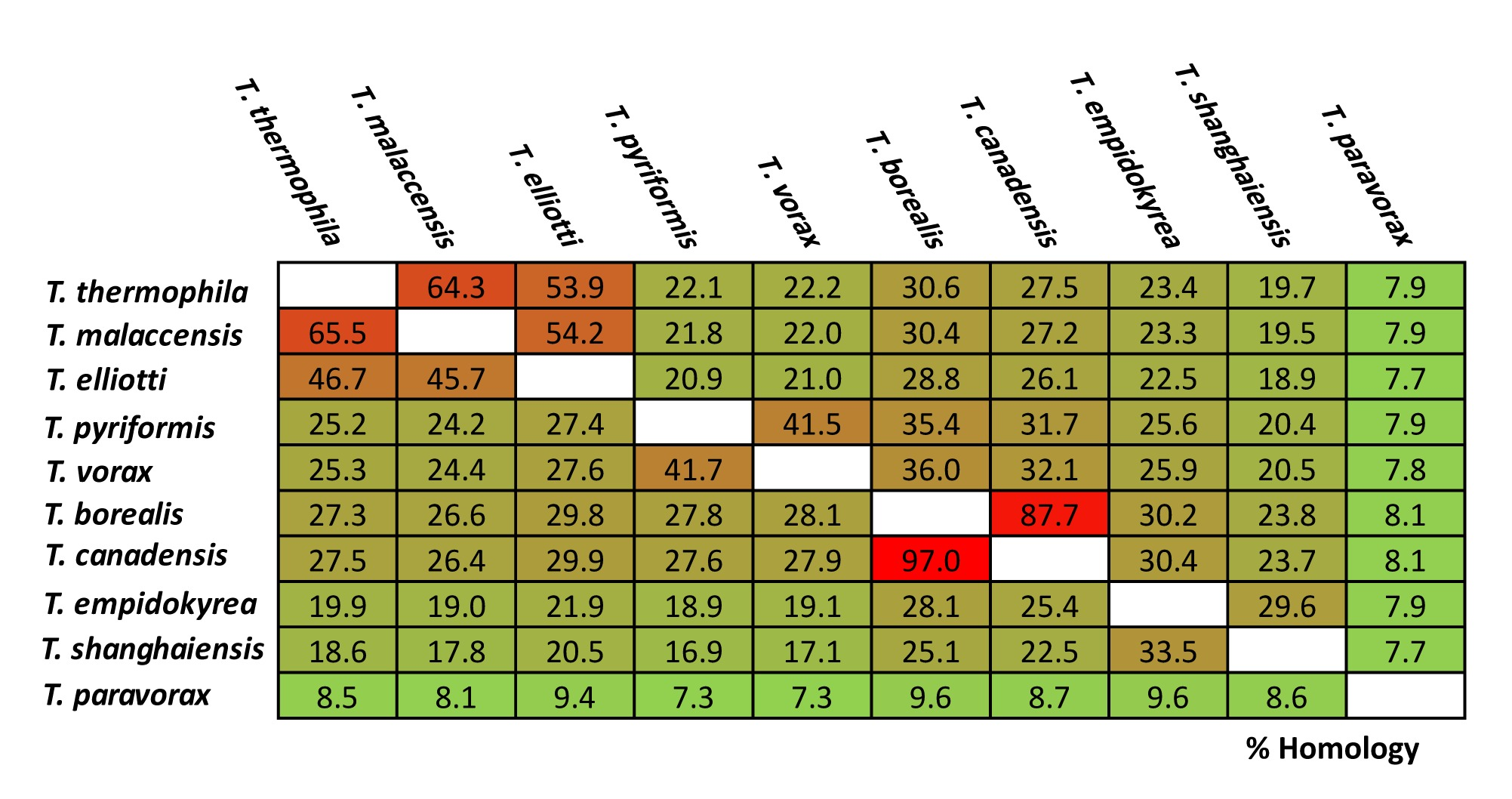

Supplement: S1 Fig — Heat map and matrix showing the percentage of homologous regions among all 10 genomes. Pairwise genome alignment was performed using promer in MUMmer 3 software (http://mummer.sourceforge.net/) with default settings, and only one-to-one alignments were used to identify homologous regions. Note that the difference between the percentages above and below the diagonal for the same species pair is due to the different genome sizes of the 2 species used as denominators when calculating the percentages. (TIF) [file pbio.3000294.s001.tif]

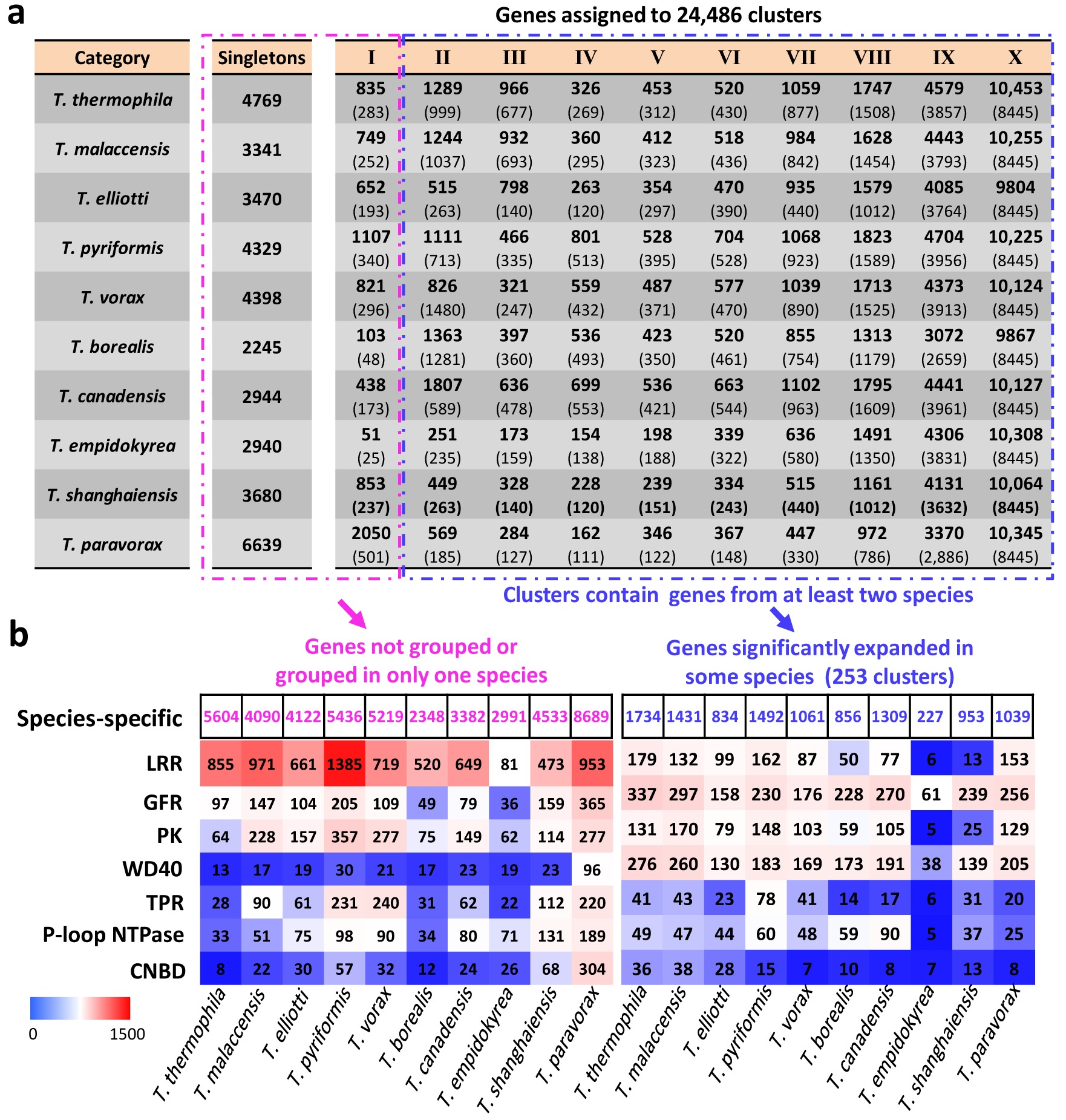

Supplement: S2 Fig — (a) Ortholog group (cluster) distributions for all 10 Tetrahymena species. A defined ortholog group contains at least 2 genes in either the same or different species (also see S39 Fig). Genes that could not be assigned to any ortholog group are defined as singletons. Ten categories of ortholog groups (roman numerals I–X) are defined based on the number of different species represented in the ortholog group, e.g., genes appearing in only 1 species are assigned to category I, etc. Red dotted box: singletons and category I genes; blue dashed box: genes in ortholog groups identified in at least 2 species (categories II–X). Numbers in bold in each cell indicate the number of genes; numbers in parenthesis indicate the number of ortholog groups. (b) Distribution of genes encoding the top 7 protein domains, which are the same for every species. Left: singletons or category I genes. Right: genes in significantly expanded ortholog groups in some species. The number of genes encoding each type of protein domain was used to generate the heat maps. (TIF) [file pbio.3000294.s002.tif]

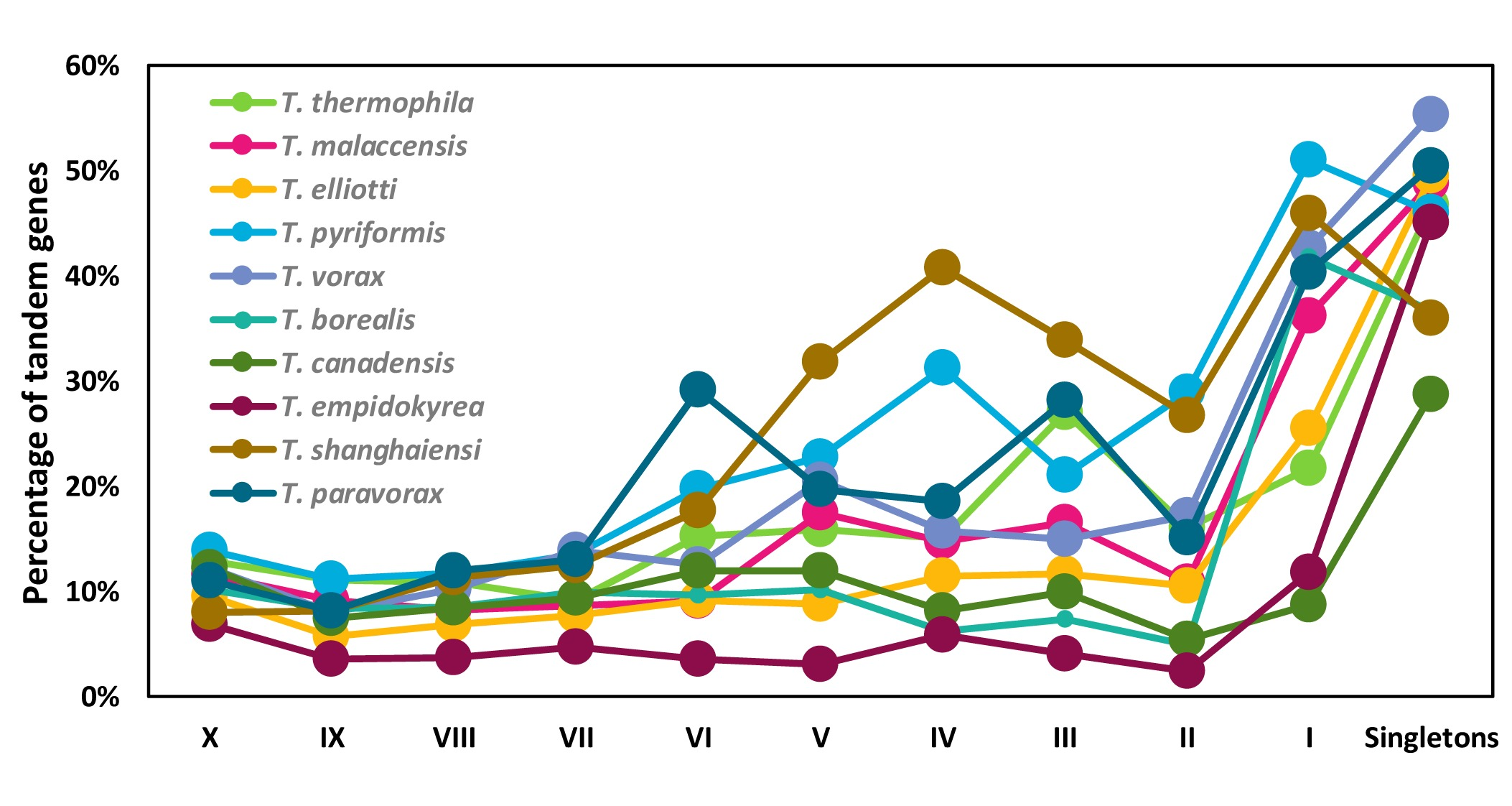

Supplement: S3 Fig — For category I–X, the percentage of tandemly duplicated genes was calculated based on tandem inparalogs in each gene cluster (OrthoMCL ortholog group). For singletons, which have no inparalogs, the tandem arrangements of genes were used to calculate the percentage regardless of whether they included inparalogs. Numerical data underlying this figure are listed in S2 Data. (TIF) [file pbio.3000294.s003.tif]

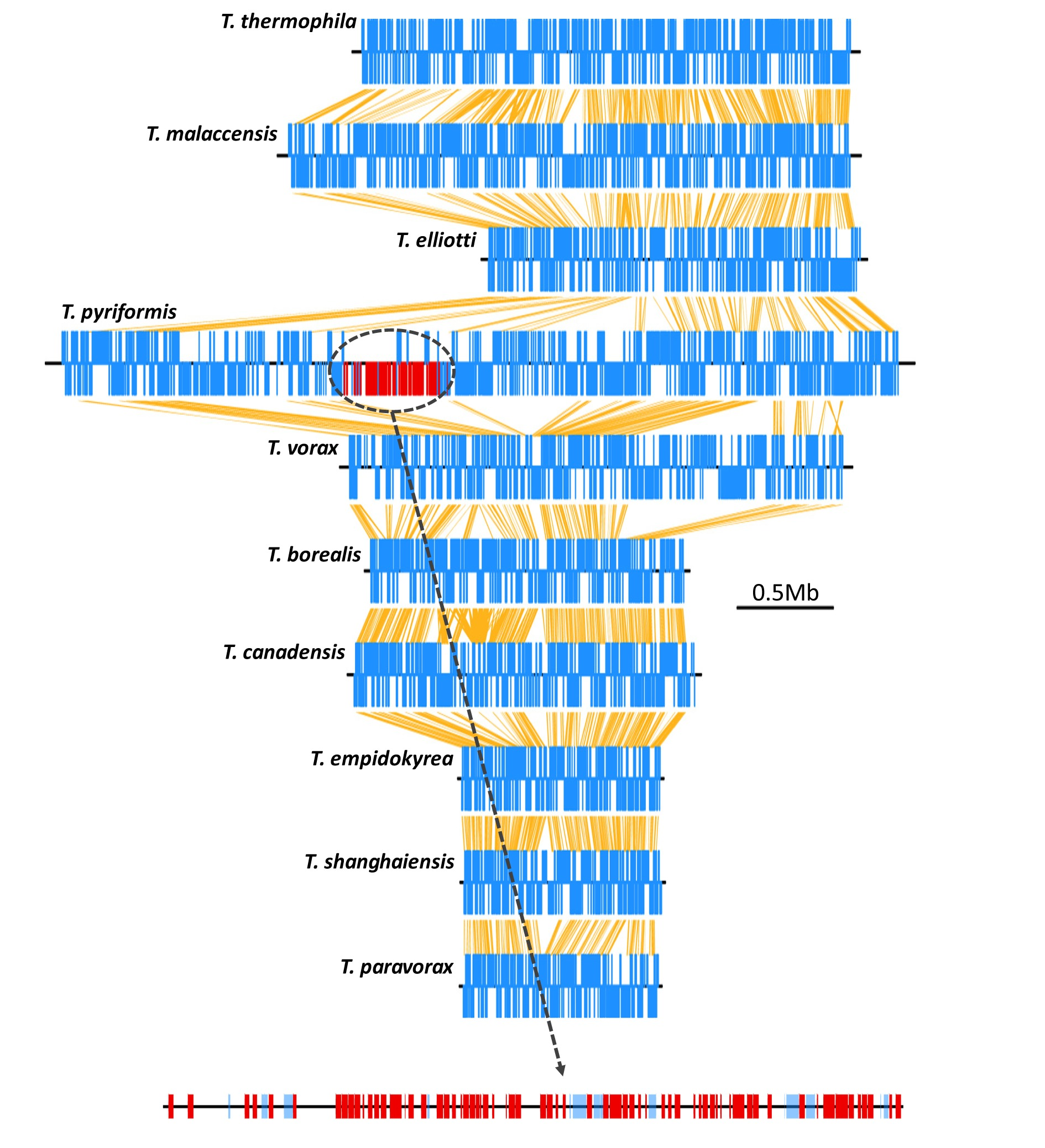

Supplement: S4 Fig — The synteny map of homologous MAC scaffolds for 10 species shows the largest cluster of tandem duplicated inparalogs. For each species, blue bars above the horizontal black line represent genes transcribed in the forward direction, and blue bars under the horizontal black line represent genes transcribed in the reverse direction. Orange lines between maps of different species represent the one-to-one ortholog relationships among the 10 species. The red bars under the central black line of the T. pyriformis map represent the largest tandem duplicated gene cluster among the 10 species, containing 17 strict tandem LRR inparalogs. This cluster is extended to 65 LRR inparalogs if 2 inparalogs are allowed to be separated by up to 3 other unrelated genes. The dashed arrow points to an expanded view of this cluster. The synteny map indicates that this tandem inparalog cluster specifically arose in T. pyriformis. (TIF) [file pbio.3000294.s004.tif]

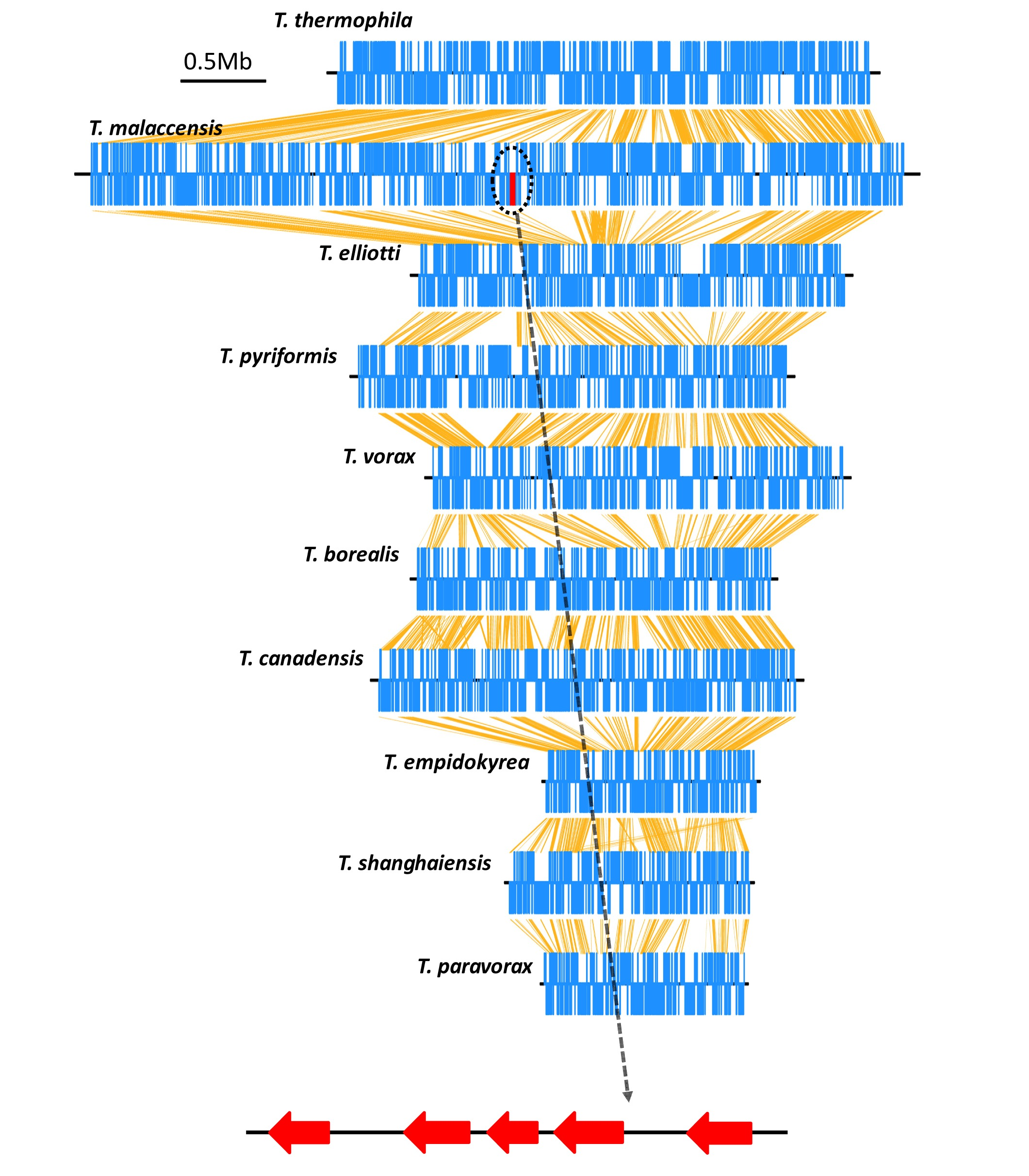

Supplement: S5 Fig — The synteny map of a MAC chromosome for 10 species shows a T. malaccensis-specific tandem duplicated LRR gene cluster. Symbols are as in S4 Fig. The red bars under the T. malaccensis horizontal black line, and the enlarged diagram at the bottom shows a tandem duplicated gene cluster containing 5 LRR genes. LRR, leucine-rich repeat; MAC, macronucleus. (TIF) [file pbio.3000294.s005.tif]

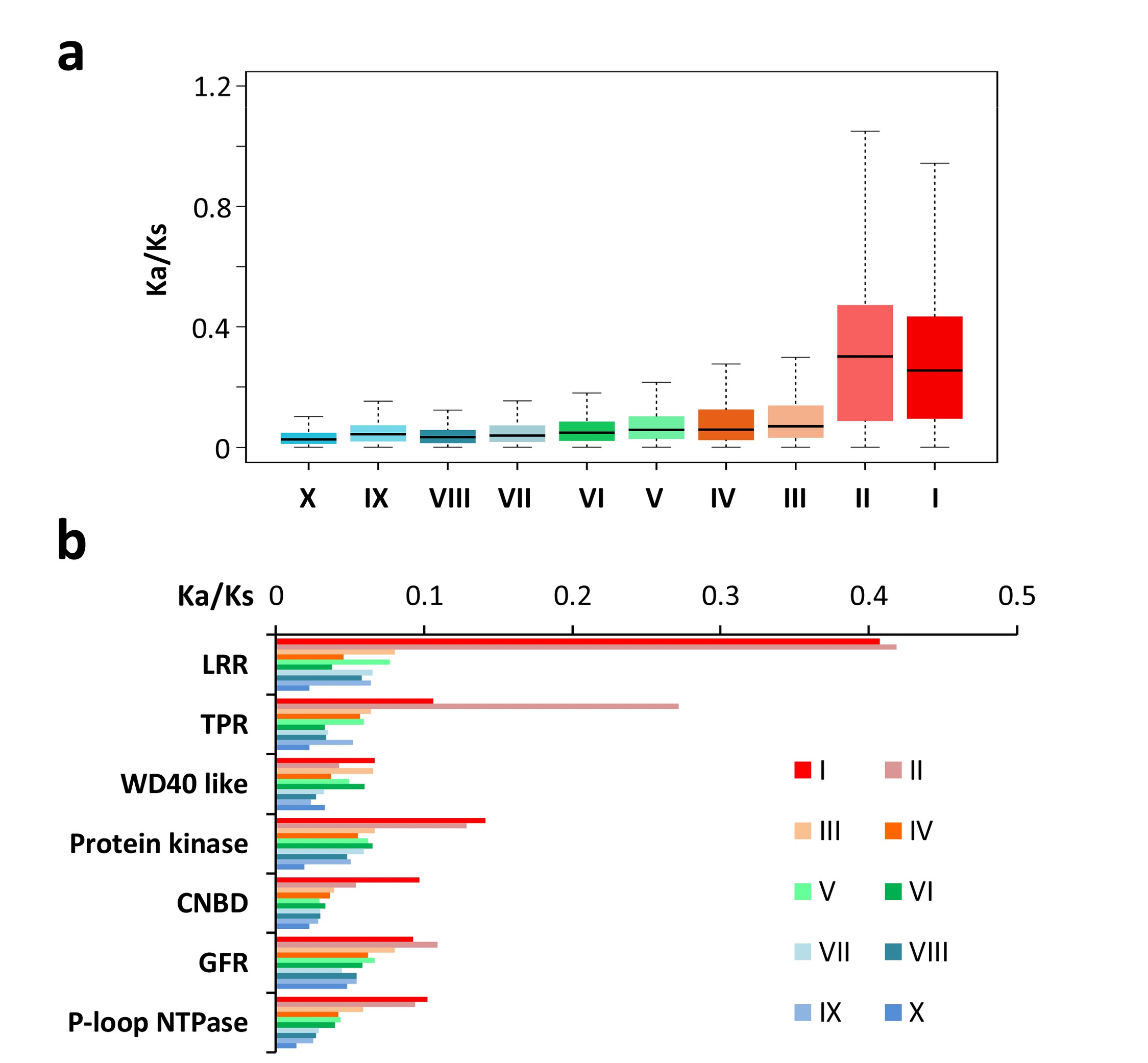

Supplement: S6 Fig — (a) The Ka/Ks ratio distribution for all 10 categories of ortholog groups. All Ka/Ks ratios for each ortholog group were used to generate the box plot (Numerical data underlying this panel can be accessed at http://ciliate.ihb.ac.cn/tcgd/download.html). (b) The Ka/Ks ratio distribution for all 7 protein domain groups and all 10 ortholog categories. The median Ka/Ks ratio for every ortholog category was used. Numerical data underlying this panel are listed in S2 Data. (TIF) [file pbio.3000294.s006.tif]

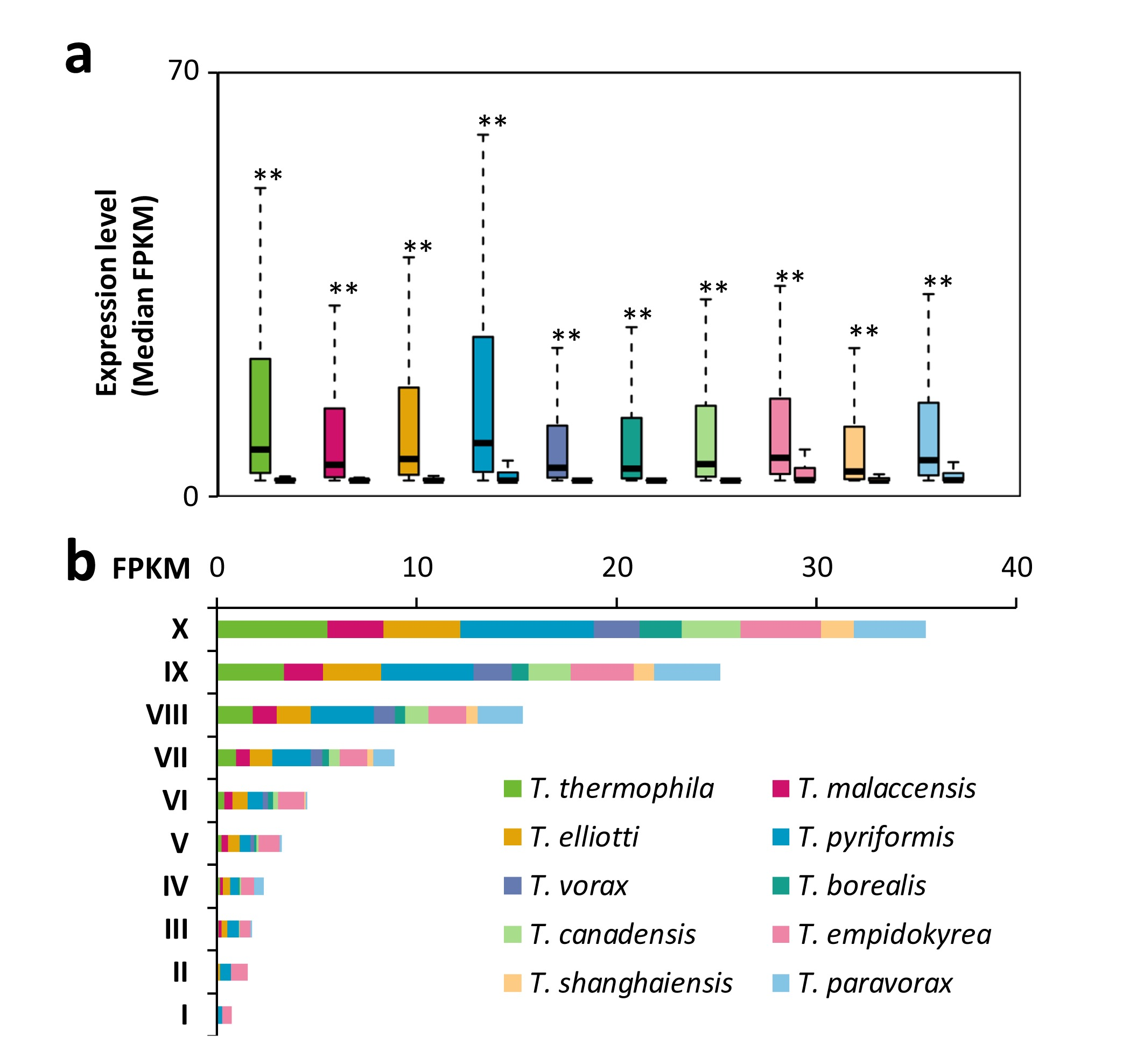

Supplement: S7 Fig — (a) Gene expression level differences between conserved (left bar) and species-specific (right bar) genes in each species. The box plot was generated using the expression values (FPKMs) of all conserved genes (left) and species-specific (right) genes. Key to the colors representing various species is shown in panel b. Two asterisks indicate that there is significant difference (Mann Whitney U test, p < 0.01) between the expression of conserved and species-specific genes. (b) Expression levels for genes for all species in all 10 categories of ortholog groups. Note that the median FPKM value for each category is plotted in each panel. Gene expression levels were measured in vegetatively multiplying cells. Numerical data underlying this figure are listed in S2 Data. FPKM, fragments per kilobase of exon per million reads mapped; SPP, super protease peptone. (TIF) [file pbio.3000294.s007.tif]

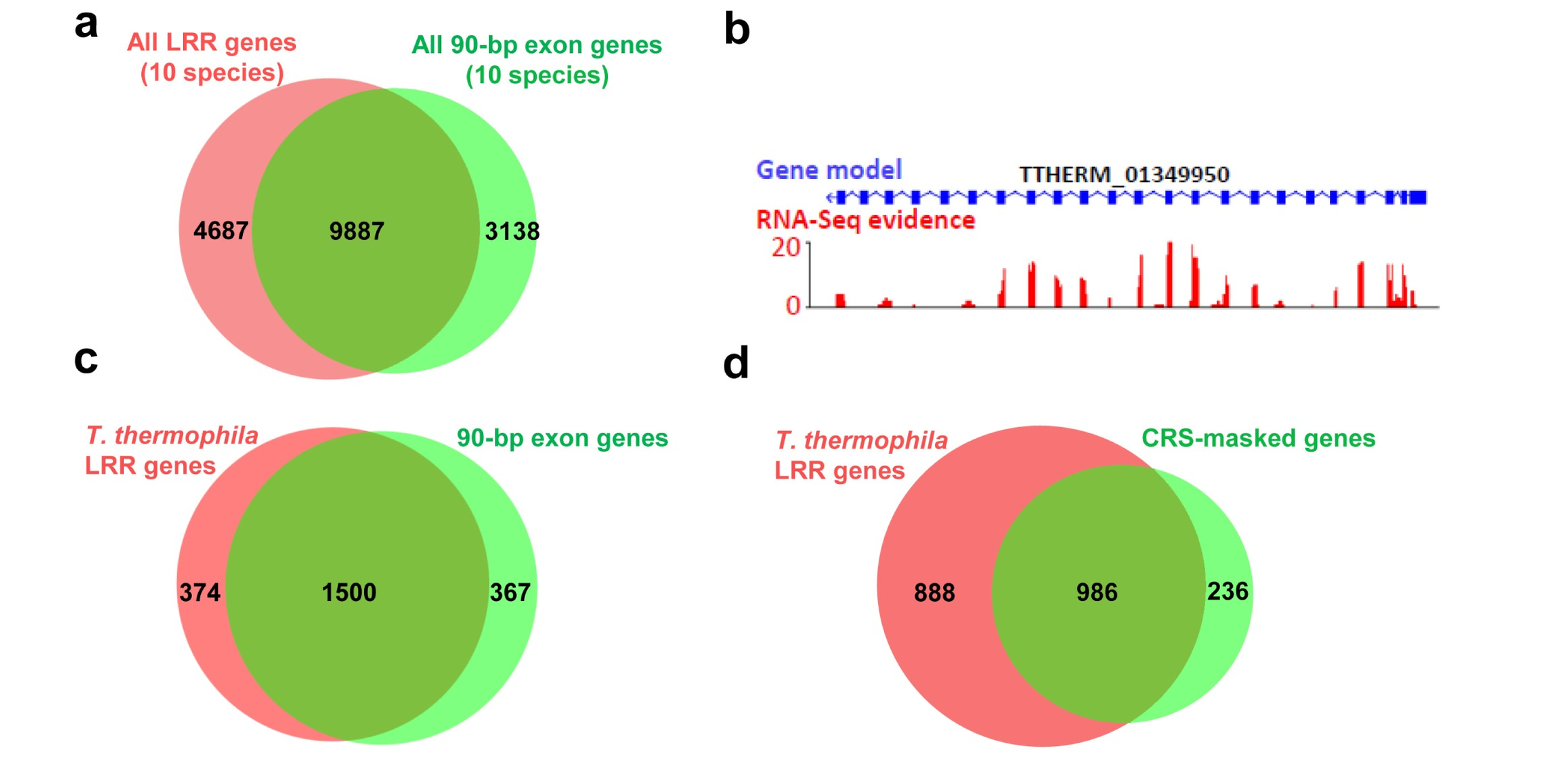

Supplement: S8 Fig — (a) Most 90-bp exon-containing genes are LRR genes and vice versa; (b) RNA-Seq evidence supports the presence of 90-bp exon arrays in LRR genes (using gene TTHERM_01349950 as an example). (c) Most LRR genes contain 90-bp exons in T. thermophila. (d) Nearly half of T. thermophila LRR genes are masked by 8 MAC CRSs. CRS, consensus repeat sequence; LRR, leucine-rich repeat; MAC, macronucleus; RNA-Seq, RNA sequencing. (TIF) [file pbio.3000294.s008.tif]

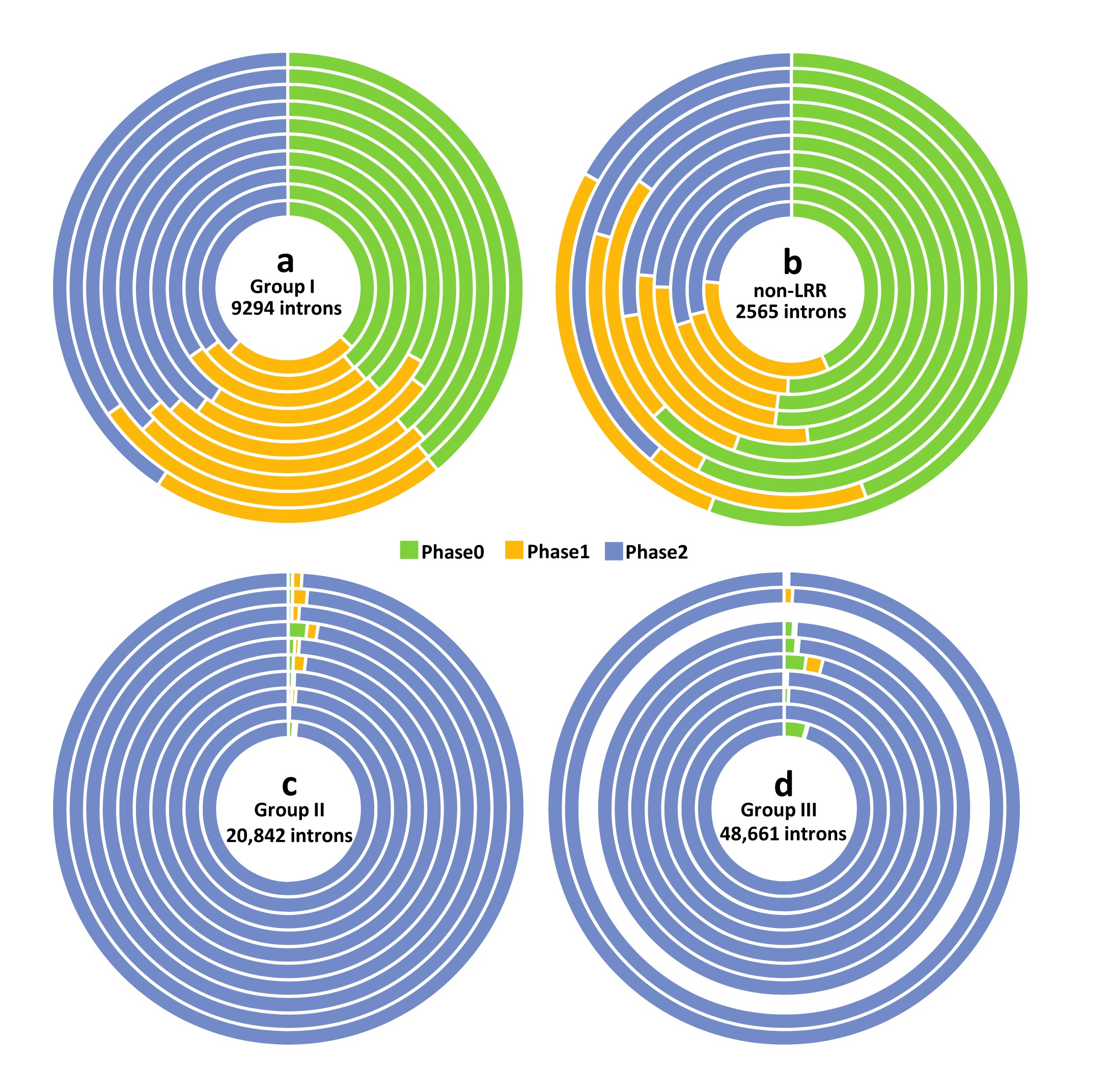

Supplement: S9 Fig — The 10 concentric circles represent the 10 species, from inside to outside: T. thermophila, T. malaccensis, T. elliotti, T. pyriformis, T. vorax, T. borealis, T. canadensis, T. empidokyrea, T. shanghaiensis, and T. paravorax. (a) Phase distribution of introns in group I LRR genes. (b) Phase distribution of introns preceding non-LRR 90-bp exons. (c) Phase distribution of introns preceding 90-bp exons in group II LRR genes. (d) Phase distribution of introns preceding 90-bp exons in group III LRR genes. Note that T. empidokyrea, a mosquito parasite, lacks group III LRR genes. LRR, leucine-rich repeat. (TIF) [file pbio.3000294.s009.tif]

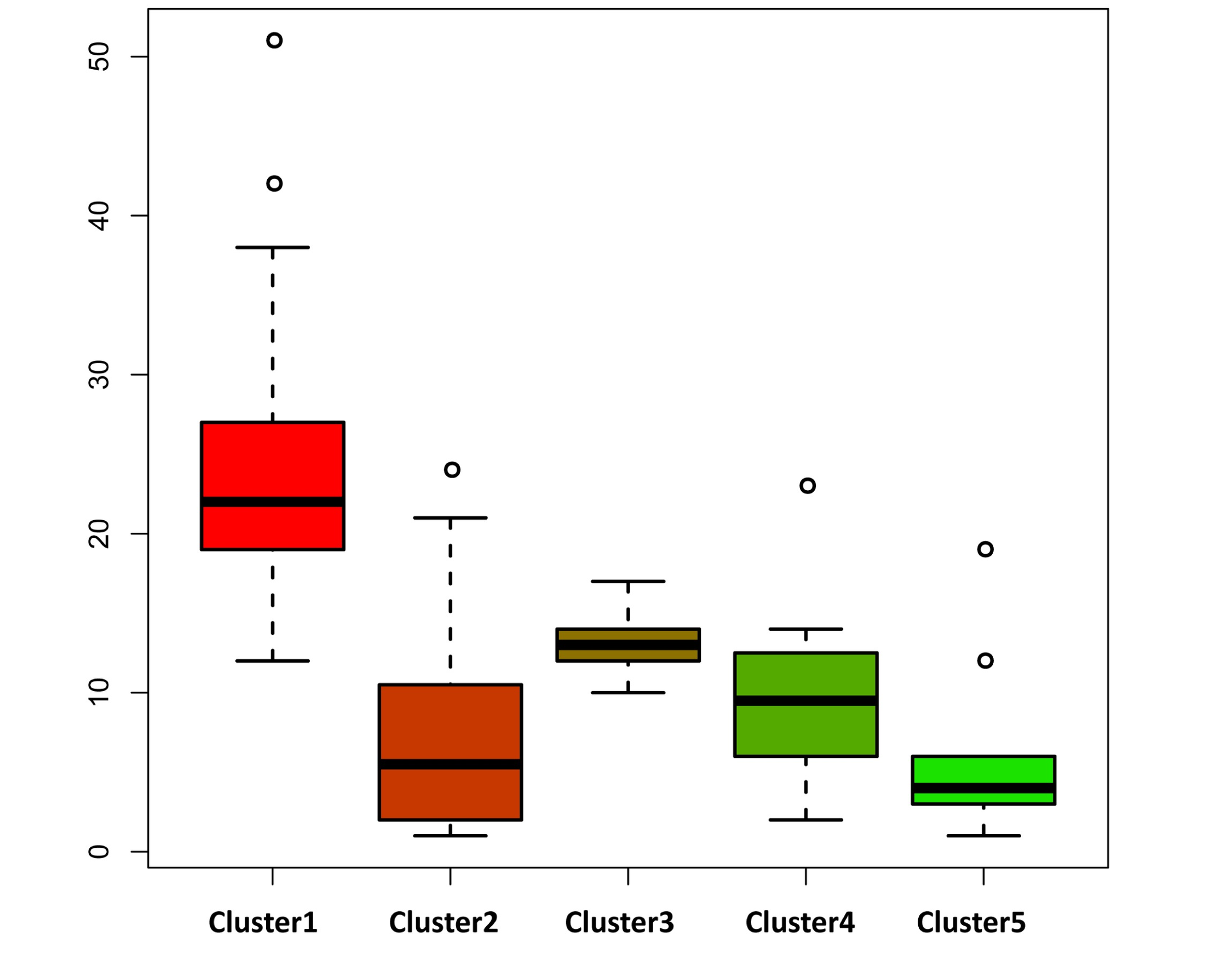

Supplement: S10 Fig — For each inparalog cluster, the box plot shows the variation of the number of 90-bp exons (y-axis) among different inparalog clusters, indicative of the diversity of gene structures (different numbers of 90-bp exons) found even among the most closely related group III LRR genes. Numerical data underlying this figure are listed in S2 Data. LRR, leucine-rich repeat. (TIF) [file pbio.3000294.s010.tif]

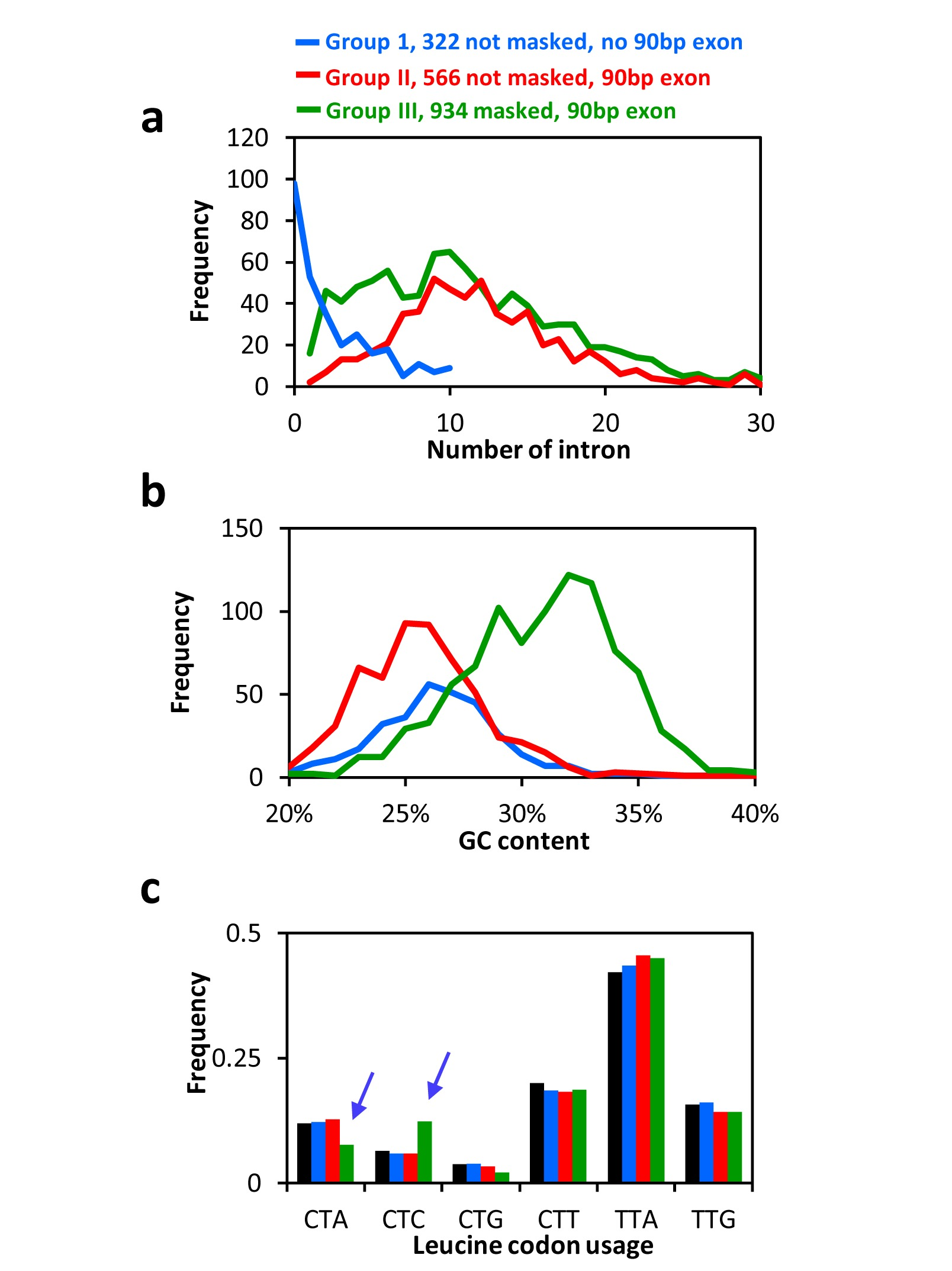

Supplement: S11 Fig — (a) Distributions of the number of introns per gene for the 3 groups of LRR genes. (b) GC content distributions for the 3 groups of LRR genes. (c) Leucine codon usage among LRR gene groups (differences are indicated with arrows). Numerical data underlying this figure are listed in S2 Data. LRR, leucine-rich repeat. (TIF) [file pbio.3000294.s011.tif]

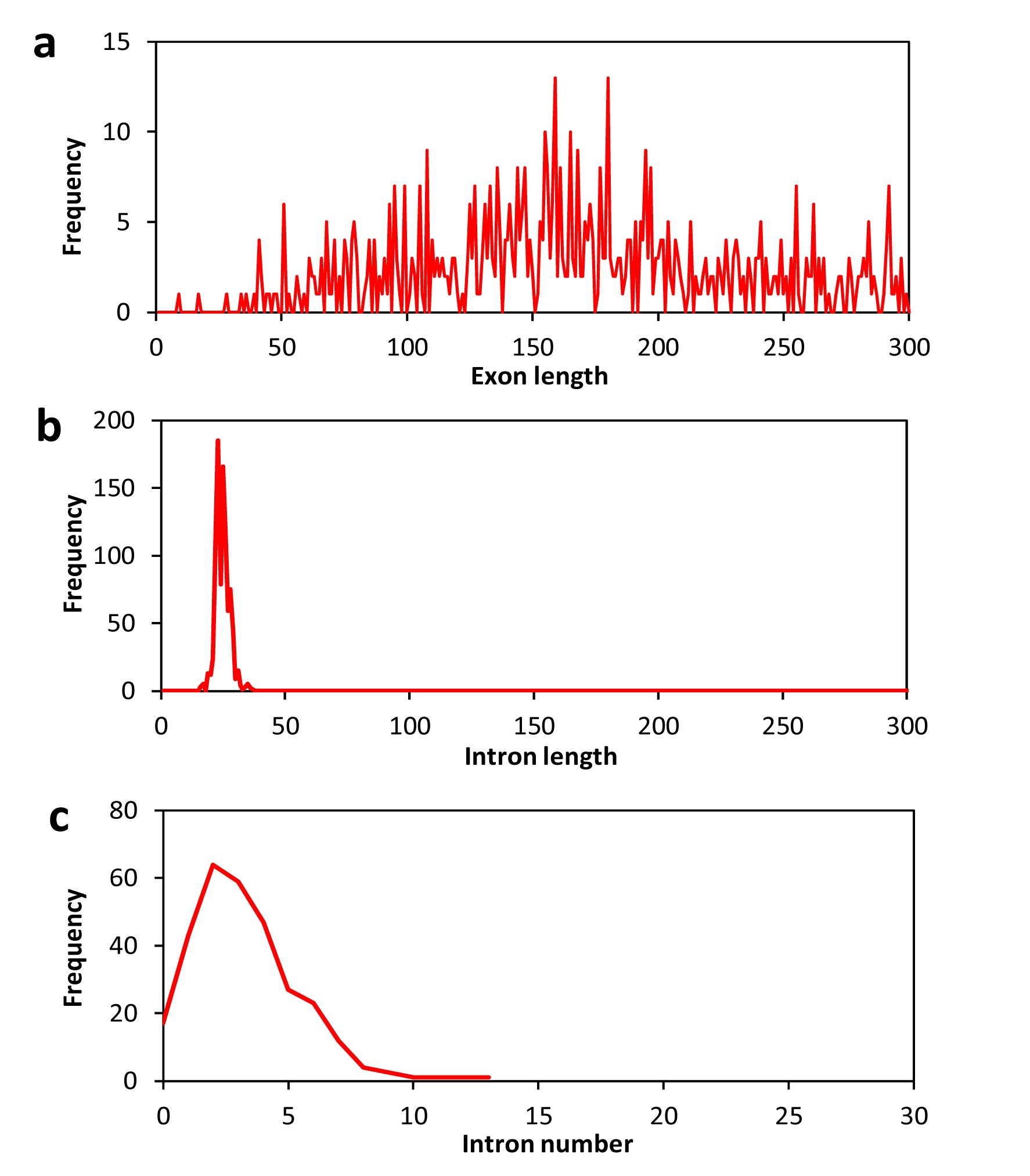

Supplement: S12 Fig — (a) P. tetraurelia LRR genes lack a prominent 90-bp exon peak. (b) No second intron length peak was observed for P. tetraurelia LRR genes. (c) P. tetraurelia LRR genes usually have no or few introns. This distribution is similar to that of all P. tetraurelia genes. LRR, leucine-rich repeat. (TIF) [file pbio.3000294.s012.tif]

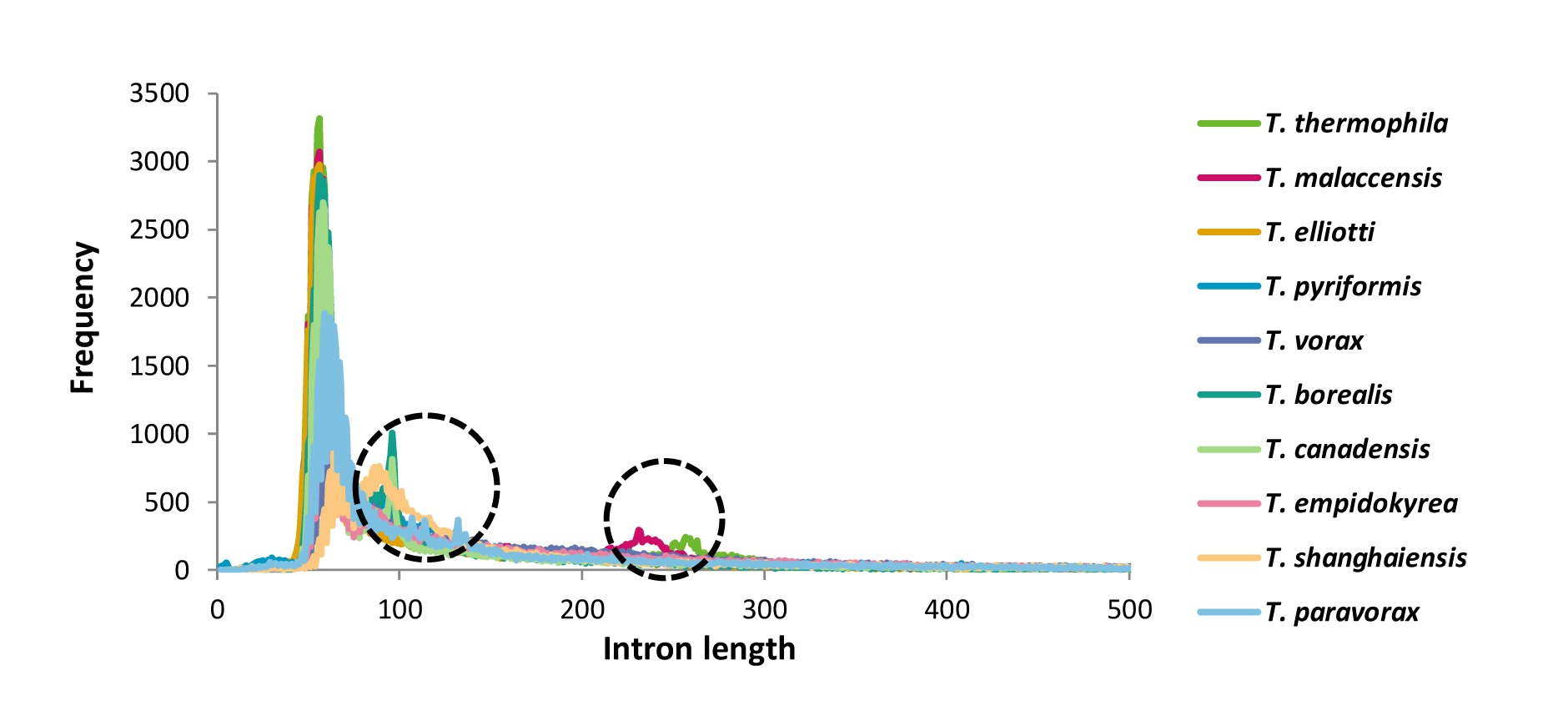

Supplement: S13 Fig — The dashed circles indicate secondary peaks. (TIF) [file pbio.3000294.s013.tif]

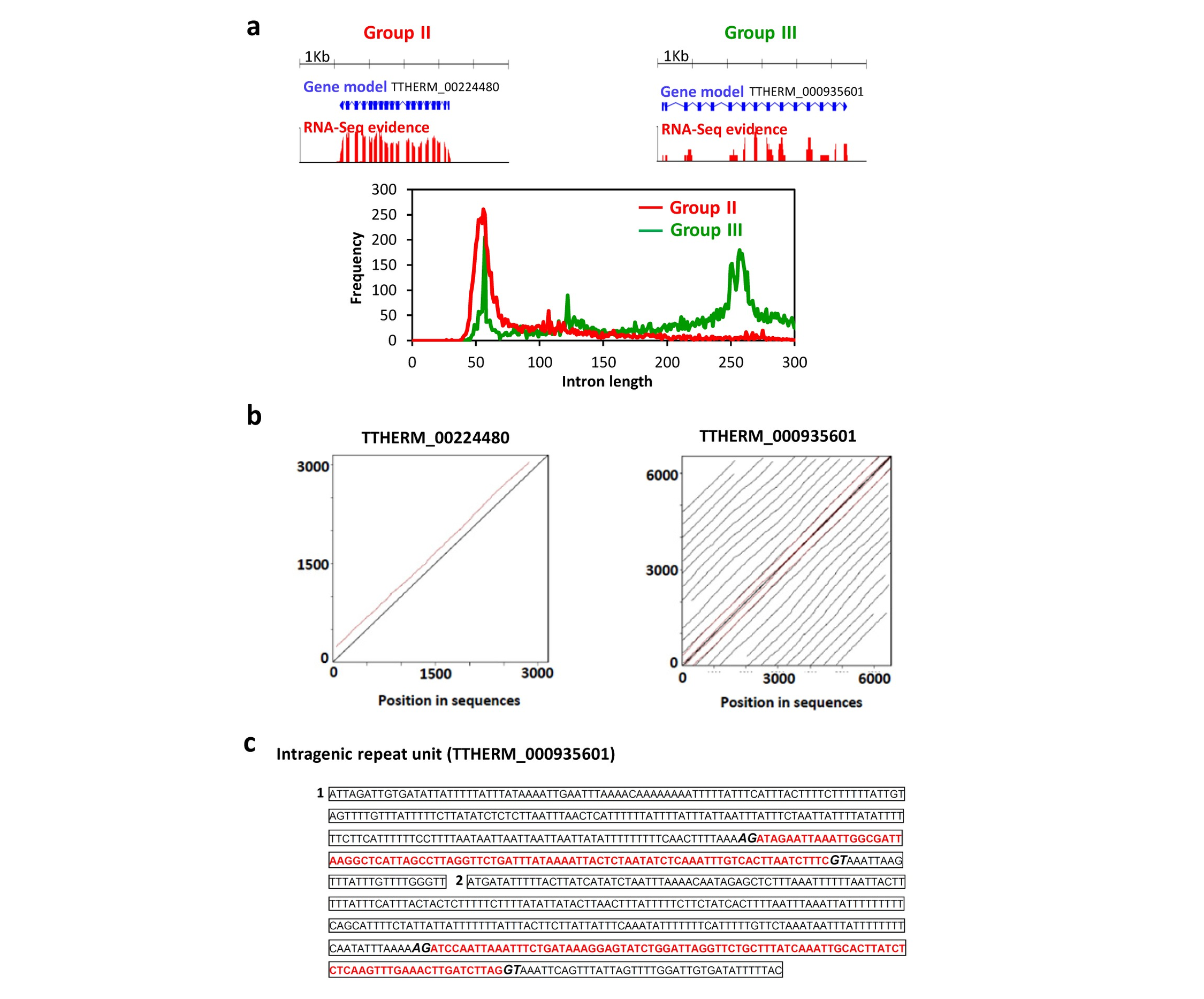

Supplement: S14 Fig — (a) Group II and group III LRR genes show different intron length distributions. Upper panels, 2 representative gene models illustrating intron length differences between group II and group III LRR genes; bottom panel, intron length distributions in all group II and group III LRR genes. (b) Intragenic repeats are more common in Tetrahymena group III than group II LRR genes. Dot plot self-alignments to detect nucleotide level intragenic repeats in the 2 representative LRR genes from panel a. Intragenic repeats are detected in the group III (right) but not in the group II (left) LRR gene. (c) Partial nucleotide sequence including 2 intragenic repeat units in the group III LRR gene in panel a. The start position (start site of a block in self-alignment) of a repeat unit is indicated by the numbers 1 and 2. Red characters highlight the 90-bp exon, and black characters represent the upstream and downstream intron sequences. Bold black characters represent the intron splice sites. LRR, leucine-rich repeat. (TIF) [file pbio.3000294.s014.tif]

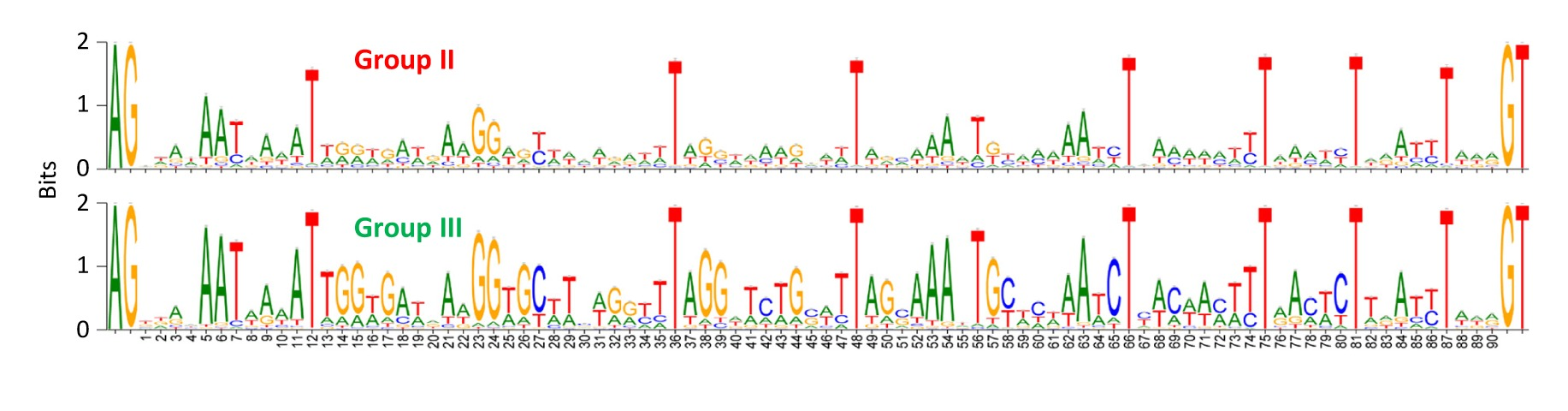

Supplement: S15 Fig — “AG” and “GT” at the left and right ends, respectively, are the intron splice sites that flank the 90-bp exons. The conserved leucine codon locations in both groups are centered at exon nucleotide positions 36, 48, 66, 75, 81, and 87. All logos were generated using WebLogo (http://weblogo.berkeley.edu/logo.cgi). LRR, leucine-rich repeat. (TIF) [file pbio.3000294.s015.tif]

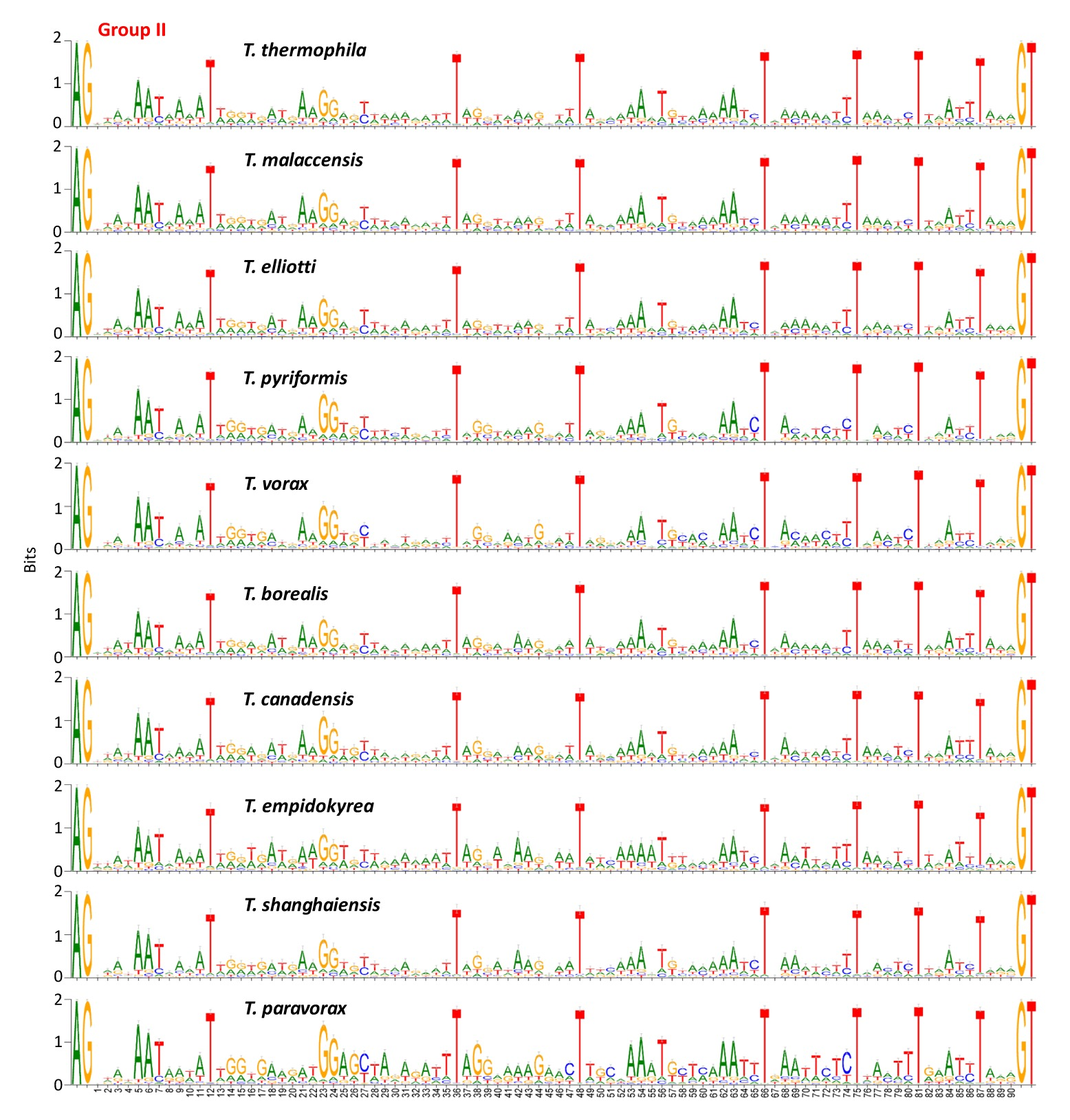

Supplement: S16 Fig — Plots are as described under S15 Fig. LRR, leucine-rich repeat. (TIF) [file pbio.3000294.s016.tif]

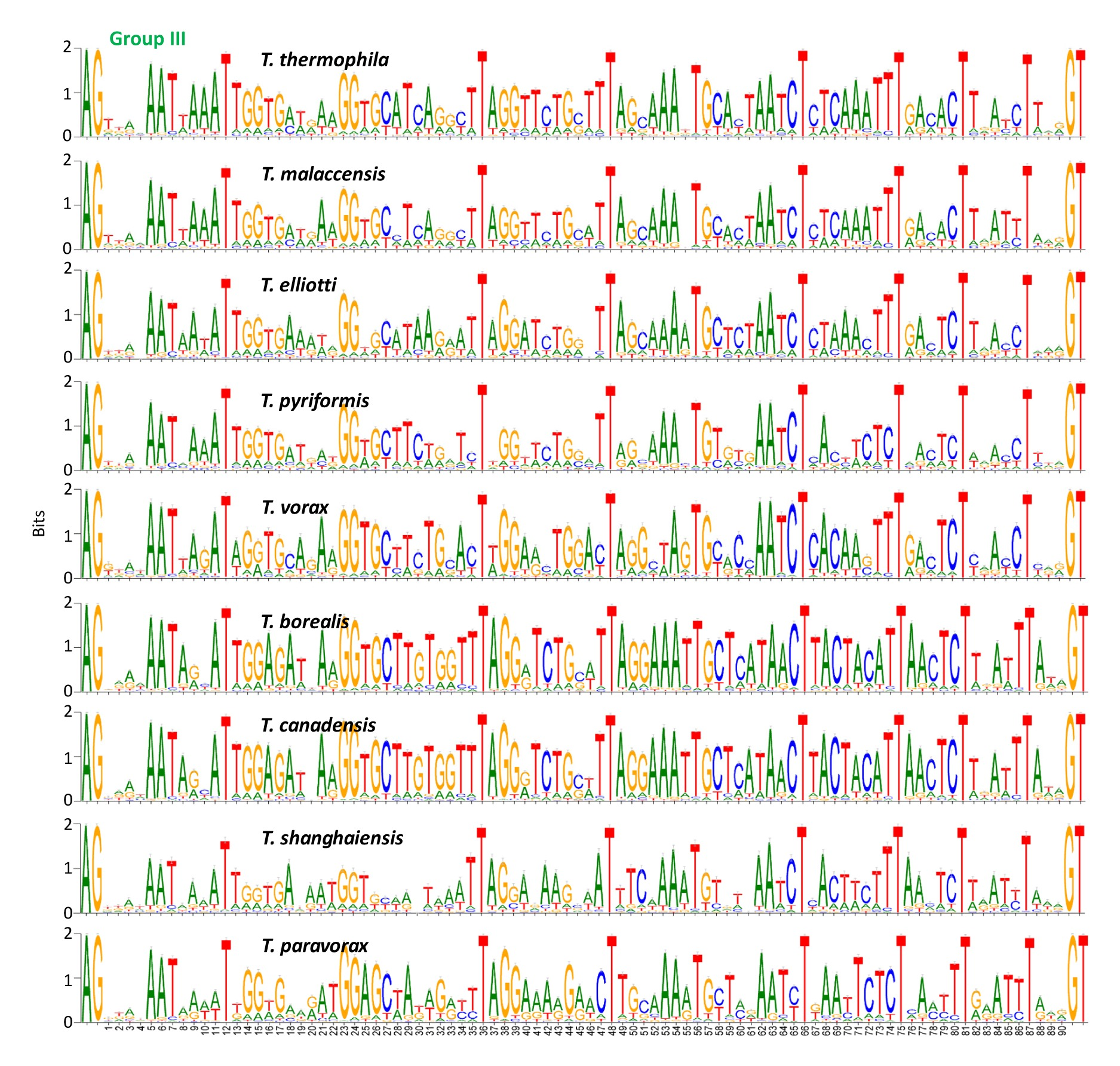

Supplement: S17 Fig — Plots are as described under S15 Fig. Note that no logo is shown for T. empidokyrea because it lacks group III LRR genes. LRR, leucine-rich repeat. (TIF) [file pbio.3000294.s017.tif]

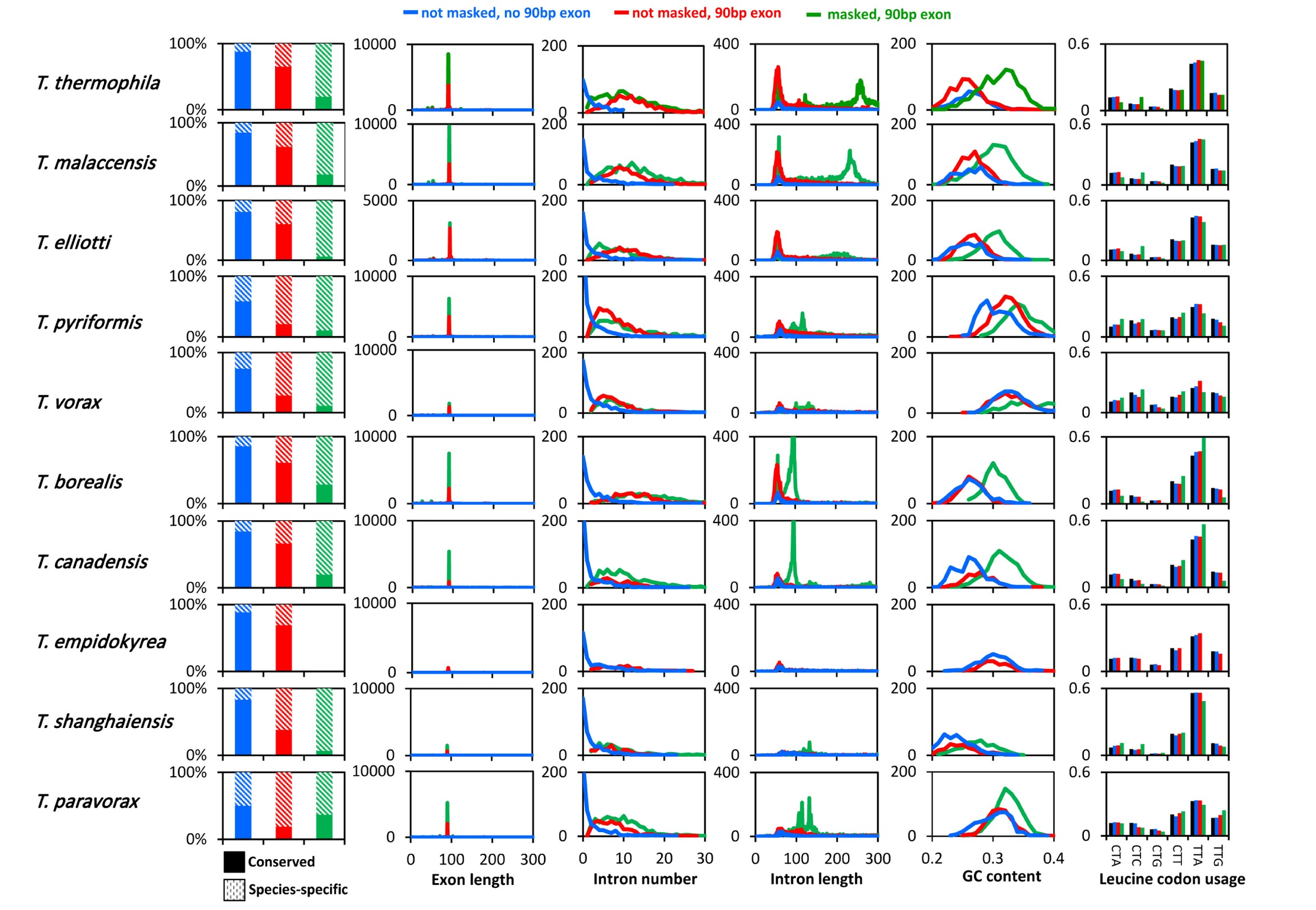

Supplement: S18 Fig — Proportions of conserved and species-specific genes (numerical data are listed in S2 Data), exon length (bp), intron number, intron length (bp), GC content, and leucine codon usage (numerical data are listed in S2 Data) were compared between group I (blue), II (red), and III (green) LRR genes in all 10 Tetrahymena species. Note that T. empidokyrea, the parasitic species, contains no group III LRR genes. LRR, leucine-rich repeat. (TIF) [file pbio.3000294.s018.tif]

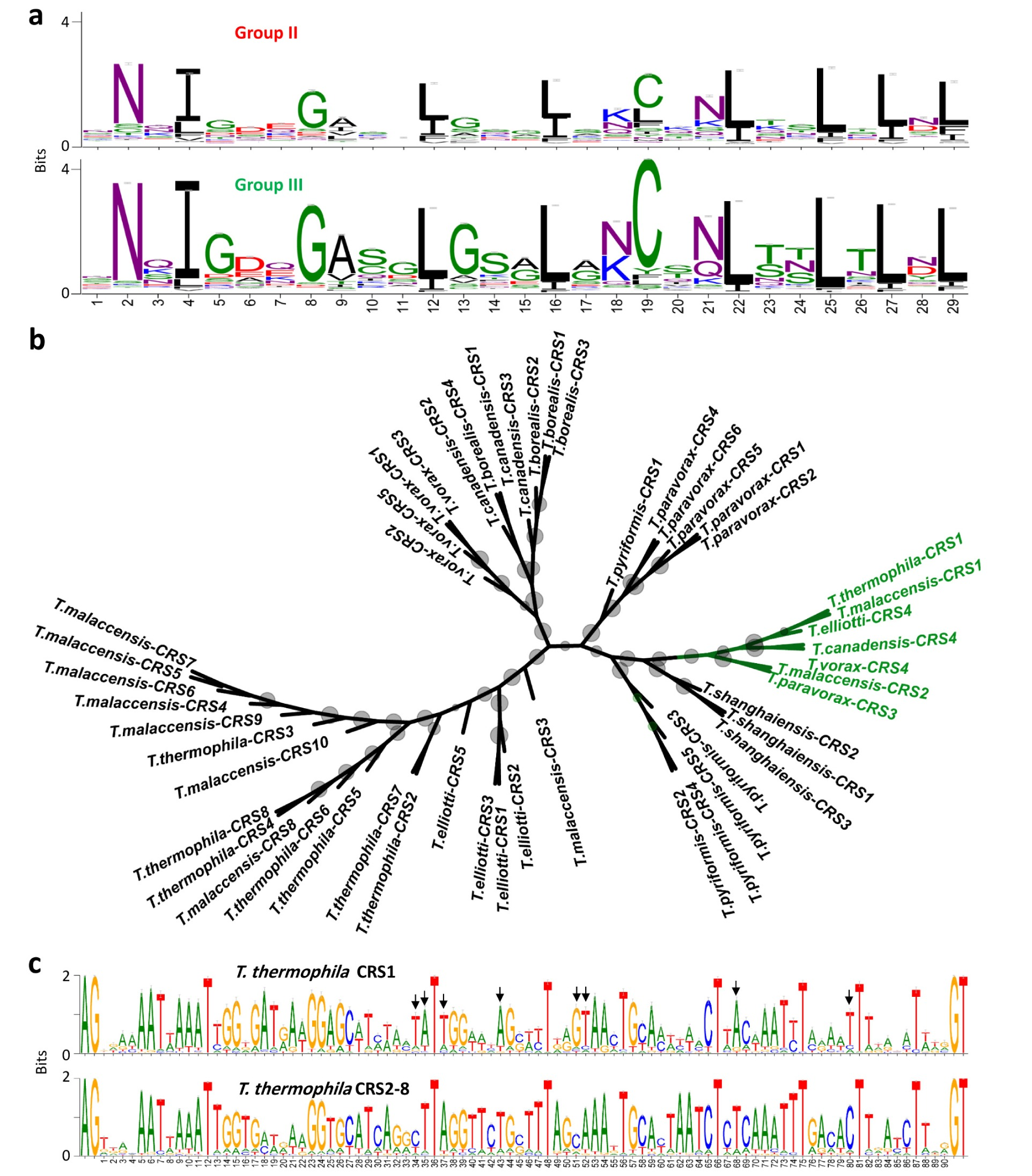

Supplement: S19 Fig — (a) comparison of 90-bp exon amino acid sequence logos of group II and III LRR genes of all 10 species. Because flanking introns have phase 2, the first nucleotide and the last two nucleotides of every exon contribute to the terminal codon of its upstream and downstream exons, respectively; therefore, only 29 amino acids are represented for each exon in all the amino acid sequence logos. (b) phylogenetic tree of consensus sequences of 90-bp exons masked by various MAC CRSs in all 10 species. For LRR genes masked by any given CRS, the consensus nucleotide sequence of all their 90-bp exons was used for the phylogenetic analysis. The clade which includes the T. thermophila CRS1-type 90-bp exons is shown in different color (green) to highlight that it clusters exons from distantly related species that likely underwent recent clonal expansions. (c) Comparison of 90-bp exon nucleotide sequence logos of T. thermophila LRR genes masked by either CRS1 (top) or all 7 other CRSs (bottom). Note that the nucleotide flips between 90-bp exons masked by CRS1 and all 7 other CRSs at position 34, 35, 37, 43, 51, 52, 68, and 80 (black arrows). CRS, consensus repeat sequence; LRR, leucine-rich repeat; MAC, macronucleus. (TIF) [file pbio.3000294.s019.tif]

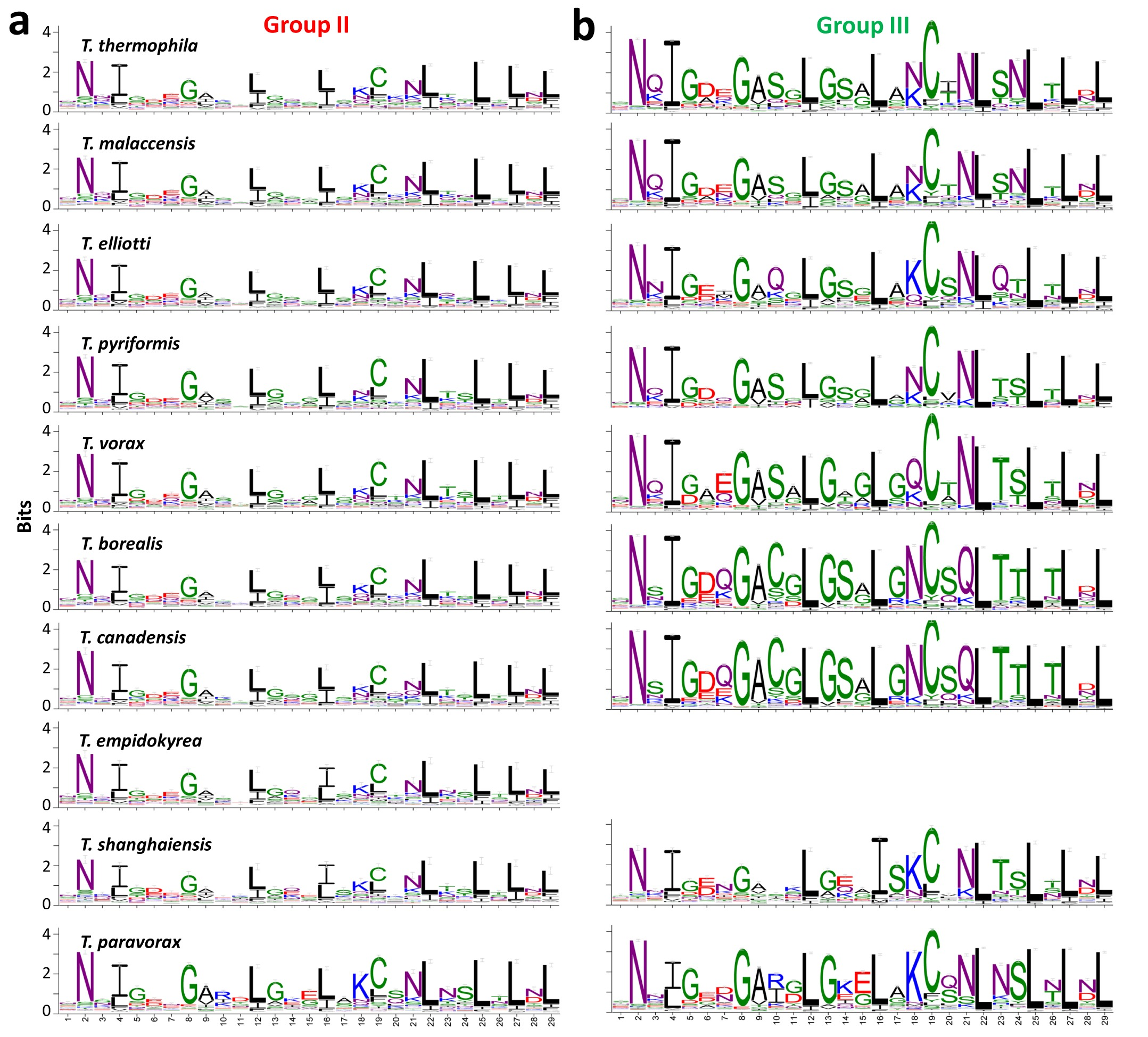

Supplement: S20 Fig — (a) Group II LRR genes. (b) Group III LRR genes. Note that T. empidokyrea is missing in panel b because it lacks group III exons. LRR, leucine-rich repeat. (TIF) [file pbio.3000294.s020.tif]

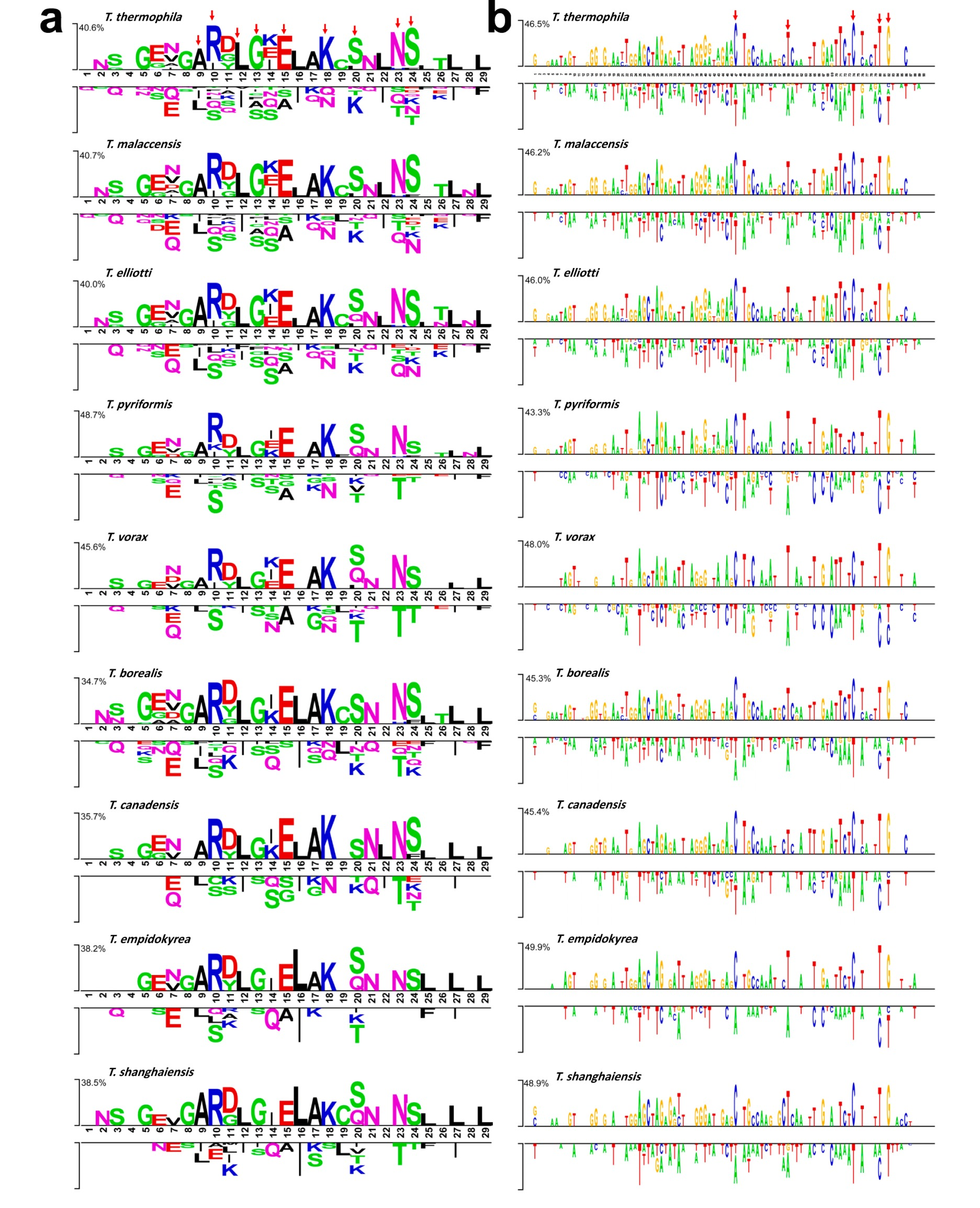

Supplement: S21 Fig — Amino acid (a) and nucleotide (b) sequence logos of 90-bp exons were compared between T. paravorax and the other 9 species using Two Sample Logos (http://www.twosamplelogo.org) (binomial test, p-value cutoff: 1 × 10^−5^). For each comparison, symbols above the central bar represent enriched levels (T. paravorax versus others), and below the bar represents depleted levels (T. paravorax versus others). The red arrows indicate highly enriched amino acids or nucleotides in all species. (TIF) [file pbio.3000294.s021.tif]

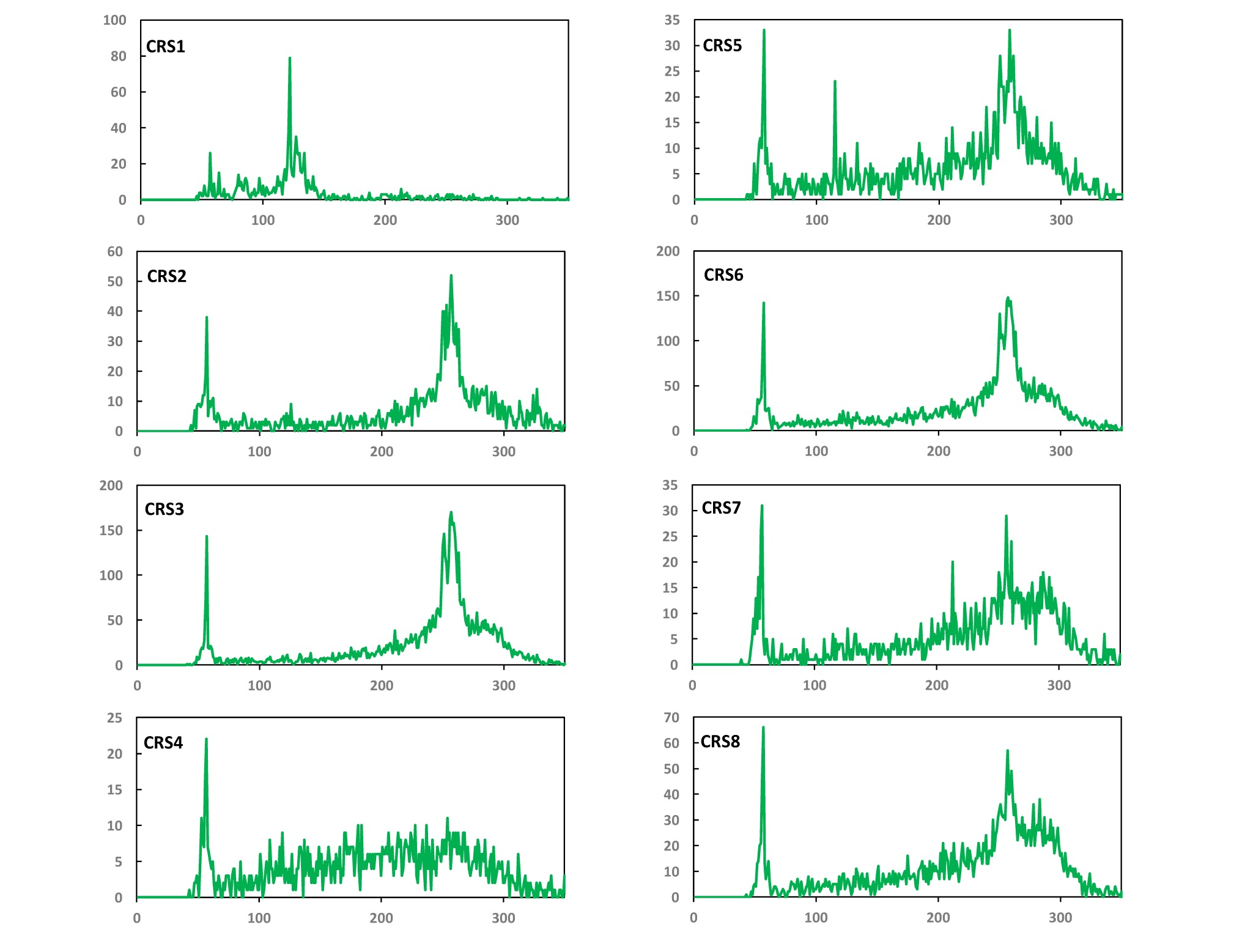

Supplement: S22 Fig — Each panel represents the introns masked by the indicated CRS. When a gene was masked by more than one CRS, it was assigned to only 1 of the 8 subgroups based on the highest masking score. CRS, consensus repeat sequence; LRR, leucine-rich repeat. (TIF) [file pbio.3000294.s022.tif]

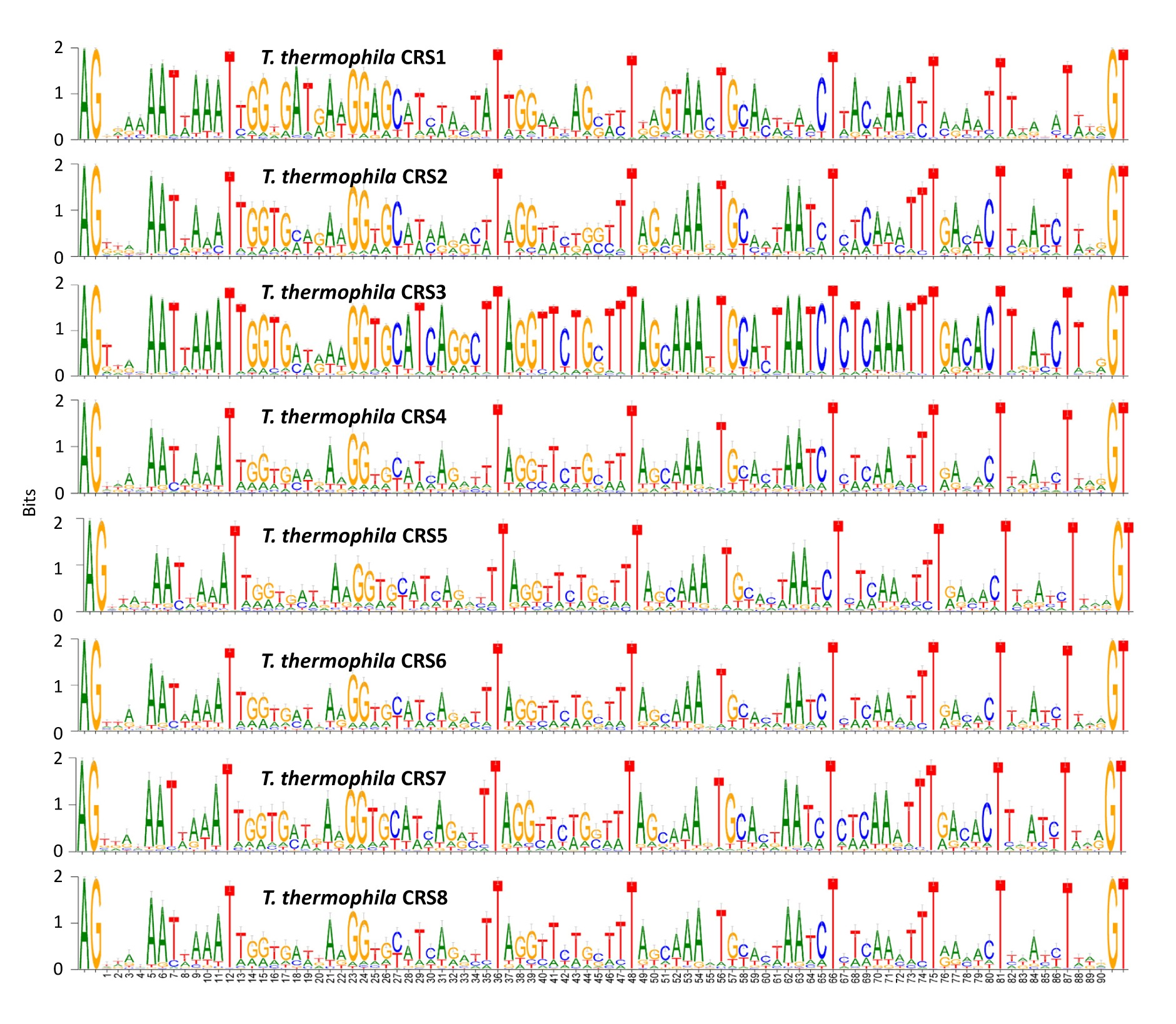

Supplement: S23 Fig — Plots are as described under S15 Fig. When a gene was masked by more than one CRS, it was assigned to only 1 of the 8 subgroups based on the highest masking score. CRS, consensus repeat sequence; LRR, leucine-rich repeat. (TIF) [file pbio.3000294.s023.tif]

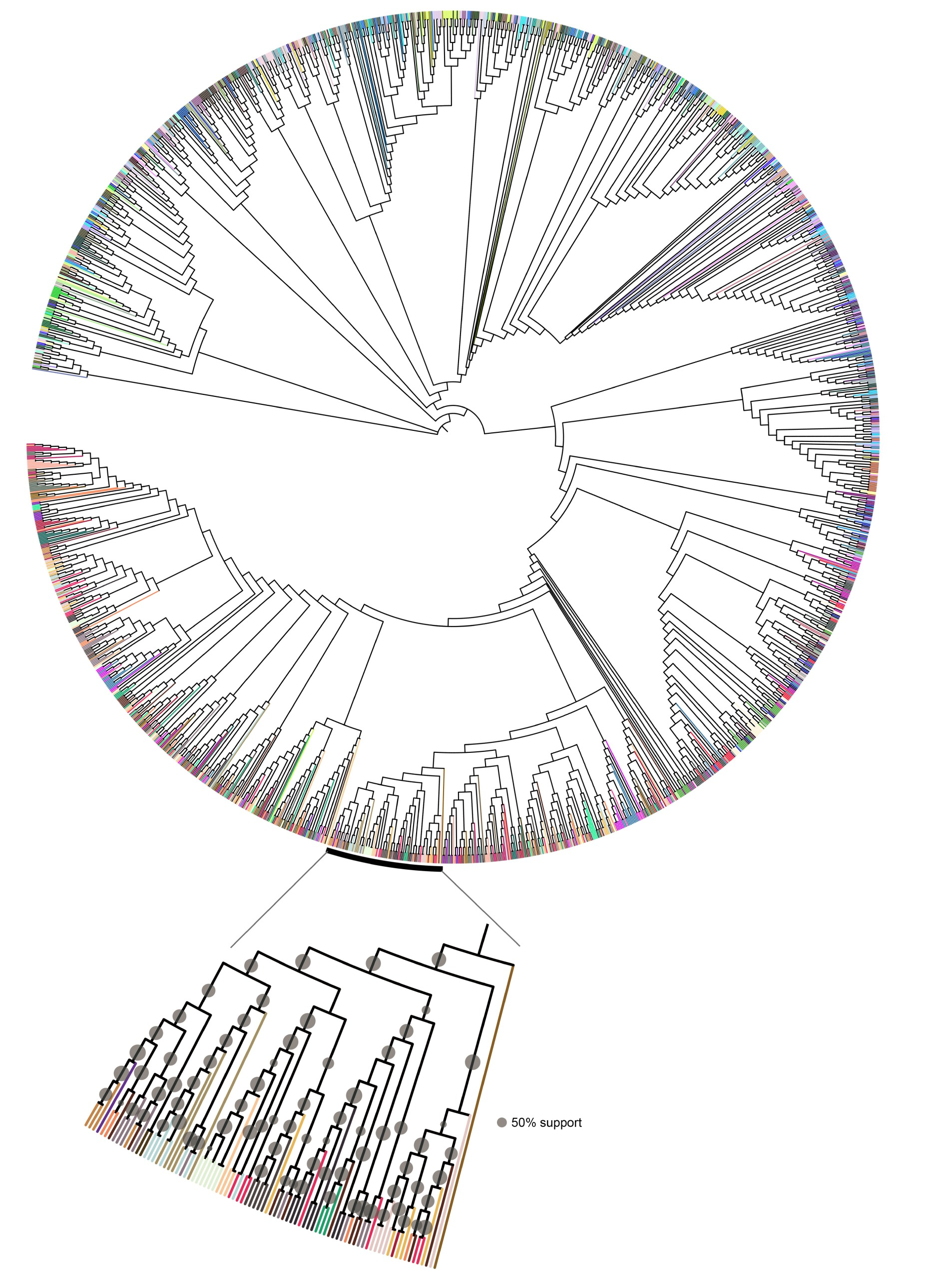

Supplement: S24 Fig — Exons of 90 bp from different inparalogs of the largest gene cluster (cluster 1 in S10 Fig) in group III LRR genes were selected for phylogenetic analysis. This gene cluster contains 83 inparalogs and a total of 1,932 exons of 90 bp. The same color was used for all the 90-bp exons from the same inparalog (total 83 different colors). Bottom left: zoom-in view of a representative clade encompassed by the curved, thick black bar. The enlarged clade illustrates more clearly the close clustering of leafs with different colors (different inparalogs), indicative of the extensive recombination of closely related 90-bp exons in different inparalogs. The size of grey dots indicate the support values of phylogenetic tree. LRR, leucine-rich repeat. (TIF) [file pbio.3000294.s024.tif]

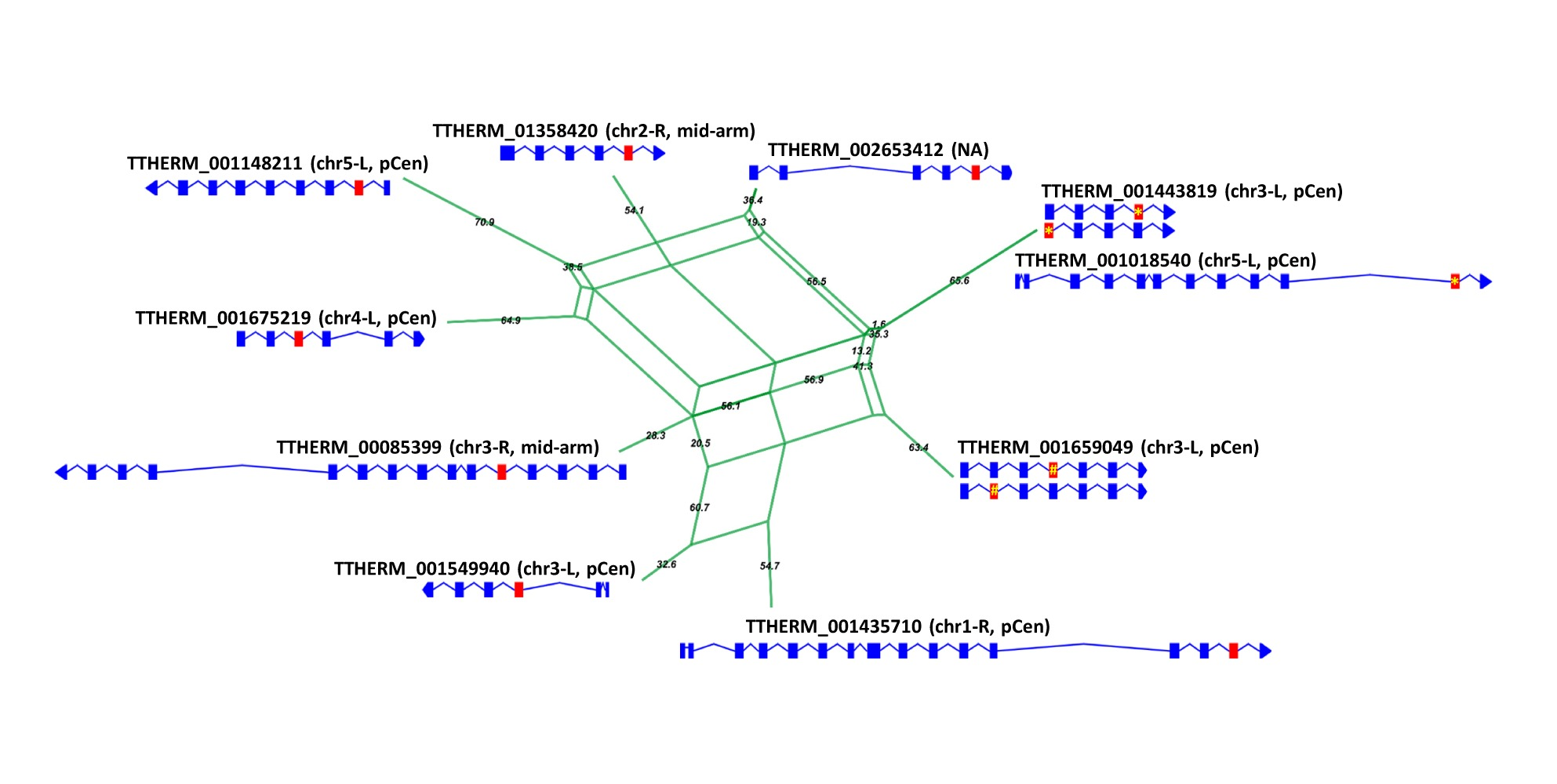

Supplement: S25 Fig — Unlike simple bifurcating trees, phylogenetic networks indicate multiple pathways of descent, e.g., as the result of recombination, which can be recognized by closed rectangles in the graph. The phylogenetic network of 12 nearly identical 90-bp exons is shown as green edges, and bootstrap supporting values are labeled. At the end of each branch are intron/exon diagrams of the 10 LRR genes containing the twelve 90-bp exons (shown in red) that share between 88 and 90 identical nucleotides. Identical 90-bp exons are indicated by yellow asterisk (*) or number sign (#). Listed above each gene: MIC chromosome location. L or R indicates the left or right arm. “pCen” indicates the pericentromeric region; “mid-arm” indicates near the middle of chromosome arms; and “NA” indicates not available because the gene is located in a still unassembled region. Note that (1) This is the largest group of nearly identical 90-bp exons and (2) TTHERM_001443819 and TTHERM_00001659049 both have 2 exons that belong to this group. The above phylogenetic network was constructed using SplitsTree4 (https://ab.inf.uni-tuebingen.de/software/splitstree4) based on the Ucorrected_P-NeighborNet-EqualAngle pipeline with default settings and 1,000 bootstraps. The phylogenetic network defines each nonconstant column in the sequence alignment as a so-called split and generates a phylogenetic graph of the split network by integrating all compatible and incompatible splits, and thus the phylogenetic network gives all the possible relationships of 90-bp exons compared to a simple phylogenetic tree. LRR, leucine-rich repeat; MIC, micronucleus. (TIF) [file pbio.3000294.s025.tif]

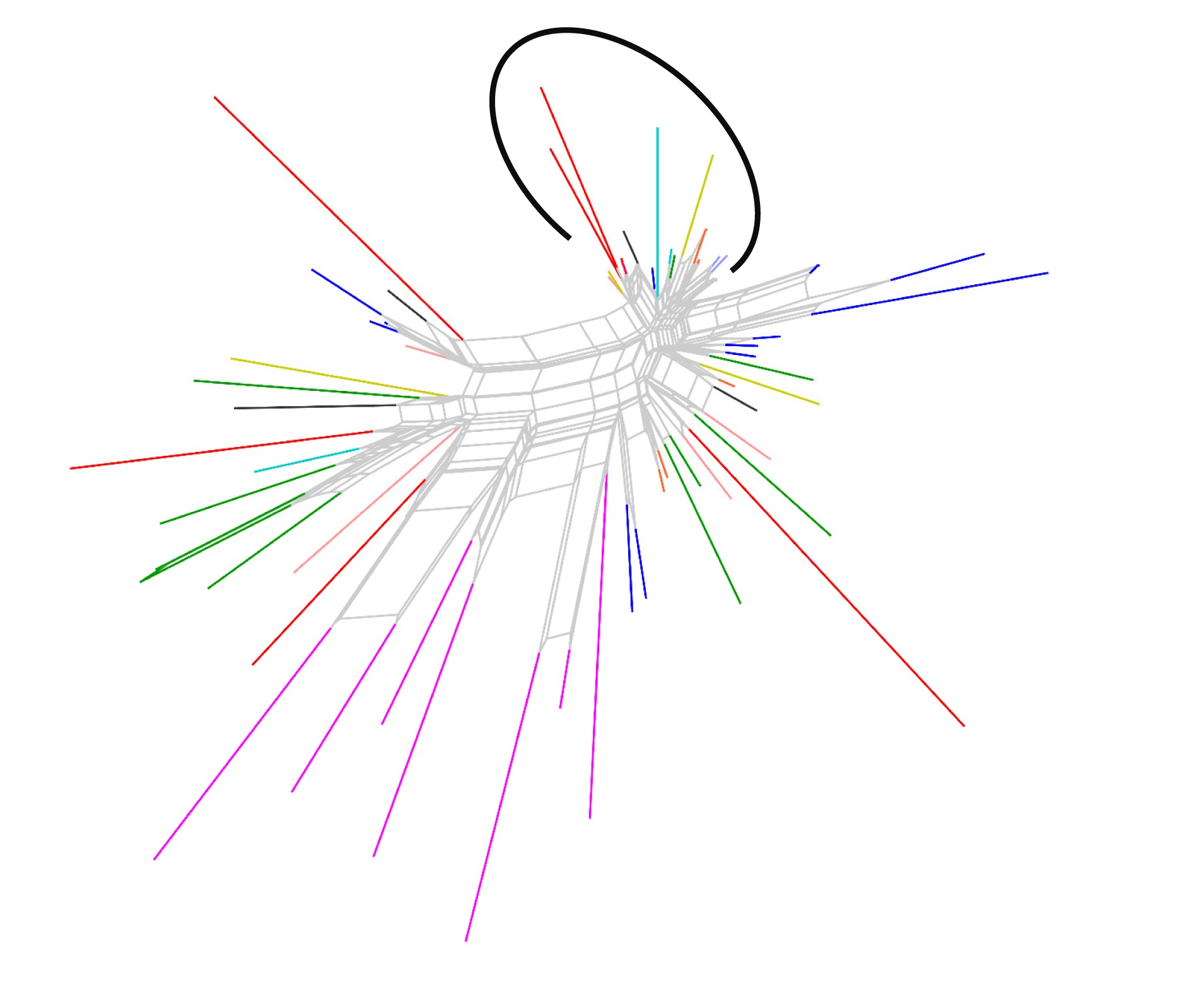

Supplement: S26 Fig — The phylogenetic network was constructed as in S25 Fig. In this type of representation, every 90-bp exon of the 10 genes containing 12 nearly identical 90-bp exons is individually shown; 90-bp exons from the same gene are shown as branches with the same color. A clade containing the 12 nearly identical 90-bp exons is highlighted with a black circular arc. Rectangles are evidence of recombination, as in S25 Fig. (TIF) [file pbio.3000294.s026.tif]

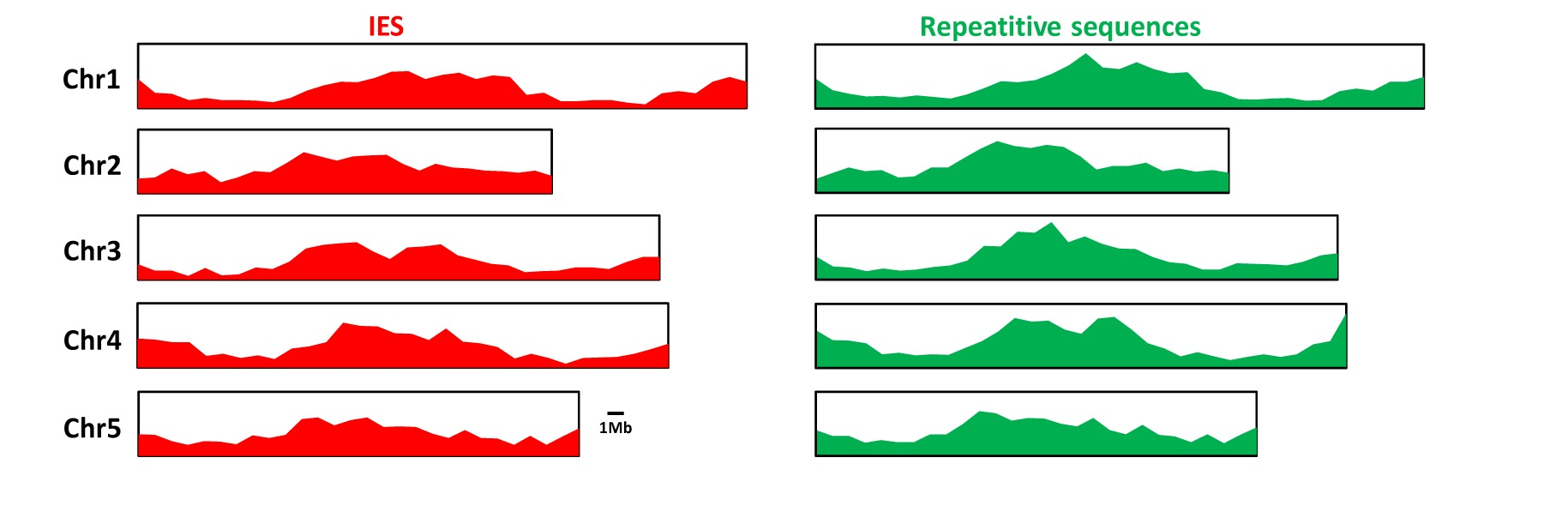

Supplement: S27 Fig — The MAC-destined components of the MIC chromosomes were divided into 1 Mb bins, and the percentages (y-axis) of IES sequences (left) and repetitive sequences (right) were plotted. The repetitive sequences, including TEs, were retrieved from the masking results using the MIC consensus sequence library in T. thermophila reported by Hamilton and colleagues [20]. IES, internal eliminated sequence; MAC, macronucleus; MIC, micronucleus; TE, transposable element. (TIF) [file pbio.3000294.s027.tif]

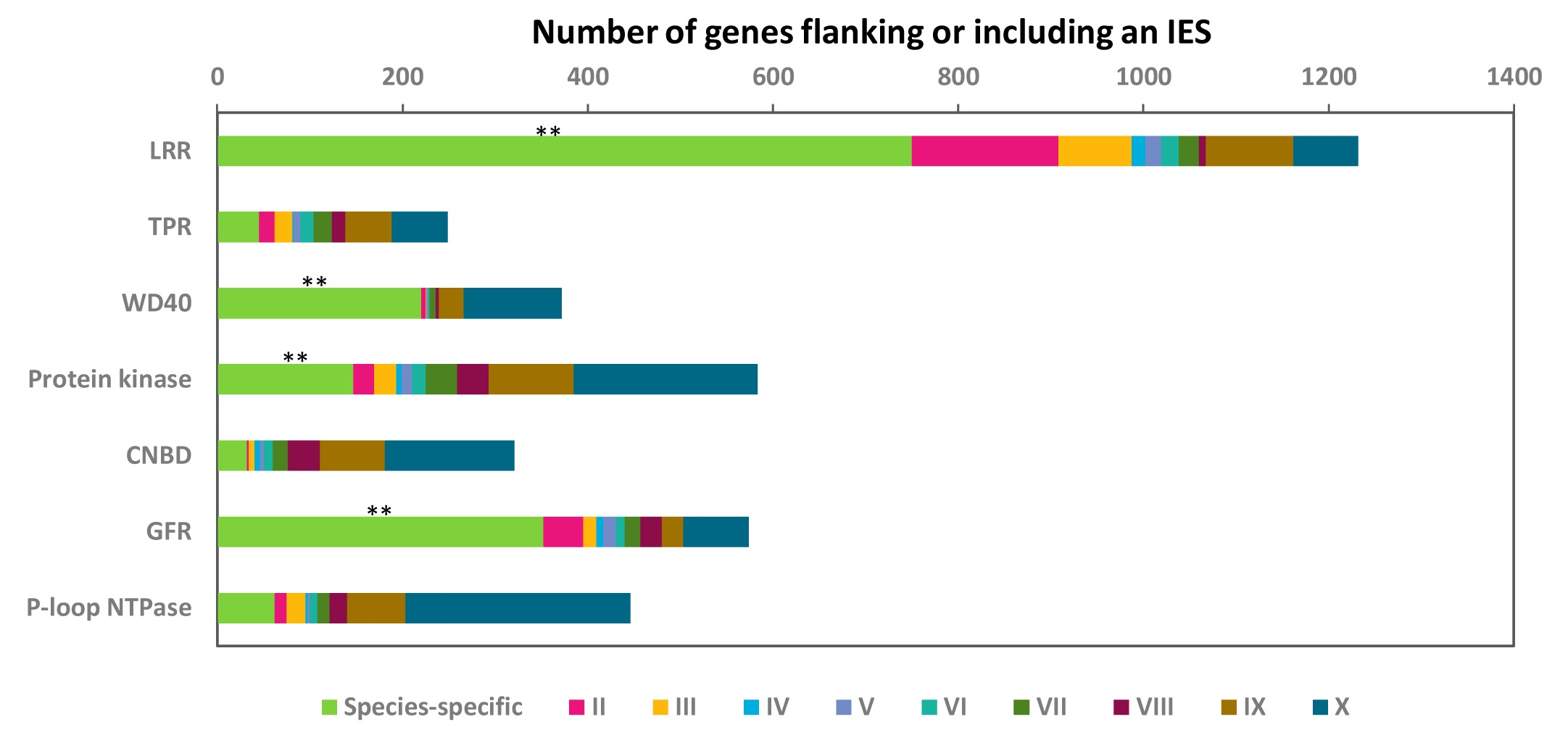

Supplement: S28 Fig — For each IES, domain(s) in the nearest genes on either side, or in the gene containing the IES (within an intron) were counted. Two asterisks indicate significant enrichment of IES-associated genes (chi-squared test, p < 0.01). Numerical data underlying this figure are listed in S2 Data. IES, internal eliminated sequence; MIC, micronucleus. (TIF) [file pbio.3000294.s028.tif]

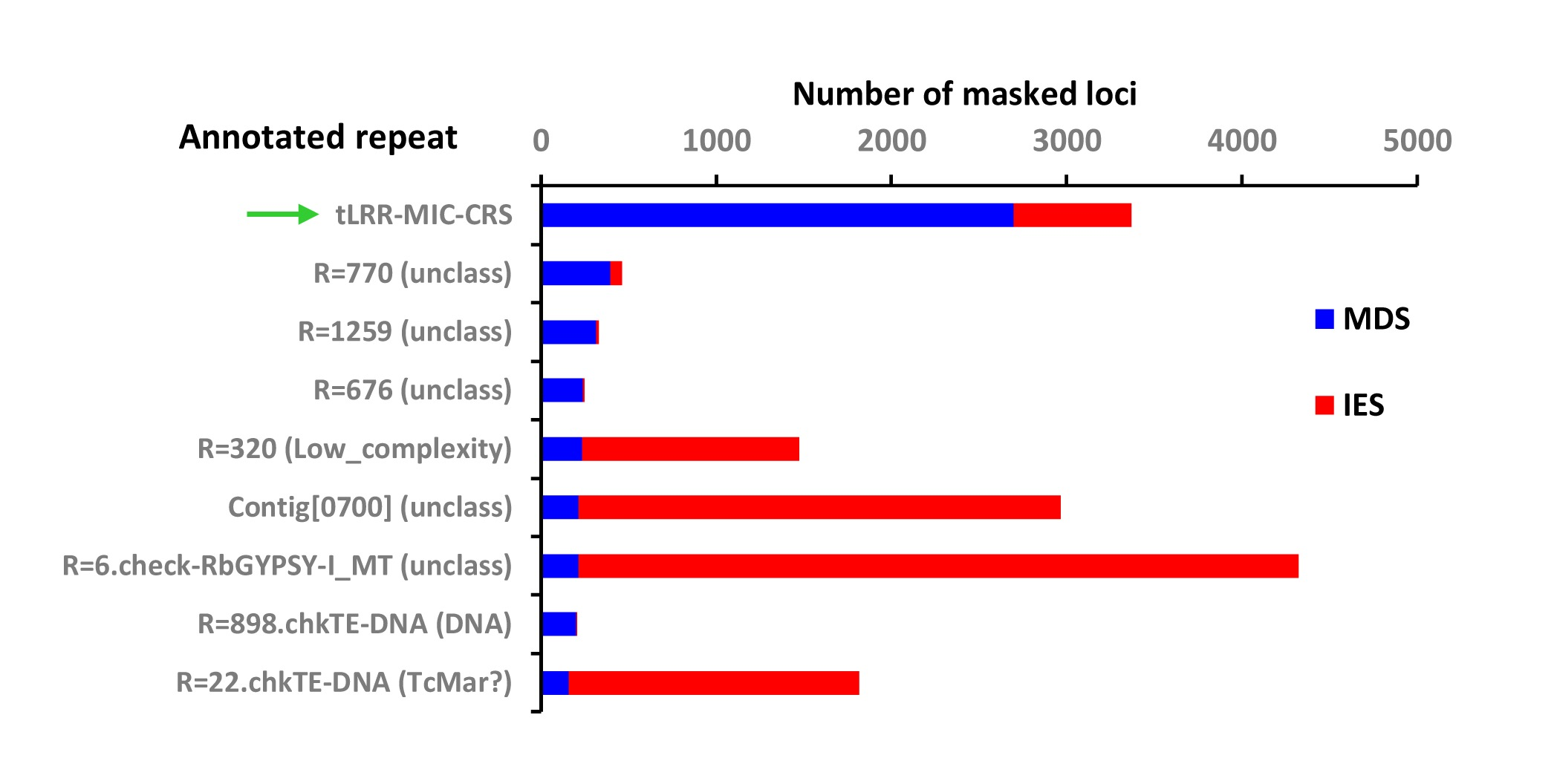

Supplement: S29 Fig — The number of MIC (red bars) and MAC (blue bars) genome loci masked by members of the MIC CRS library were sorted by the number of masked loci in MDS. Green arrow indicates sequences masked by tLRR-MIC-CRS. Numerical data underlying this figure are listed in S2 Data. CRS, consensus repeat sequence; MAC, macronucleus; MDS, MAC-destined sequence; MIC, micronucleus. (TIF) [file pbio.3000294.s029.tif]

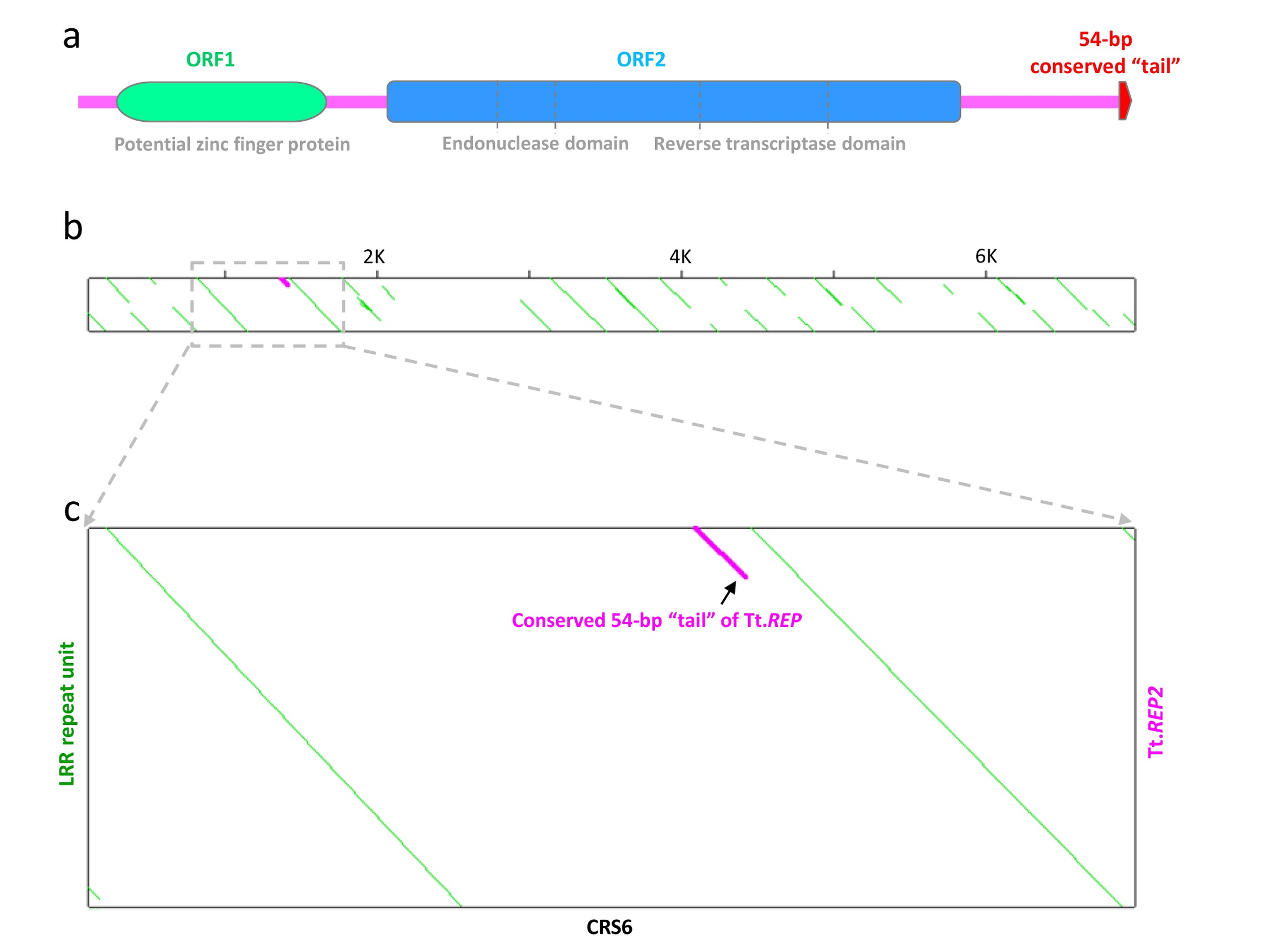

Supplement: S30 Fig — (a) Structure of a functional T. thermophila Tt.REP. It contains 2 genes named ORF1 and ORF2. ORF1 encodes a potential zinc finger protein, and ORF2 encodes a protein with both reverse transcriptase and endonuclease domains. It also contains a conserved 3′ 54-bp “tail” (red arrow) [33]. (b) Dot plot showing nucleotide sequence matches between CRS6 (x-axis) and, in the y-axis, either an LRR repeat unit (green dots) or Tt.REP2 (pink dots). (c) Enlargement of the particular intron showing the perfect match of CRS6 to the conserved REP 54-bp tail, presumably a remnant of an originally functional REP copy inserted within an LRR gene intron. Since CRS6 is a consensus sequence, this match implies a clade of LRR introns having copies of the REP remnant. Sequence alignments and dot plots were generated using YASS (http://bioinfo.lifl.fr/yass/yass.php). CRS, consensus repeat sequence; LRR, leucine-rich repeat; MAC, macronucleus. (TIF) [file pbio.3000294.s030.tif]

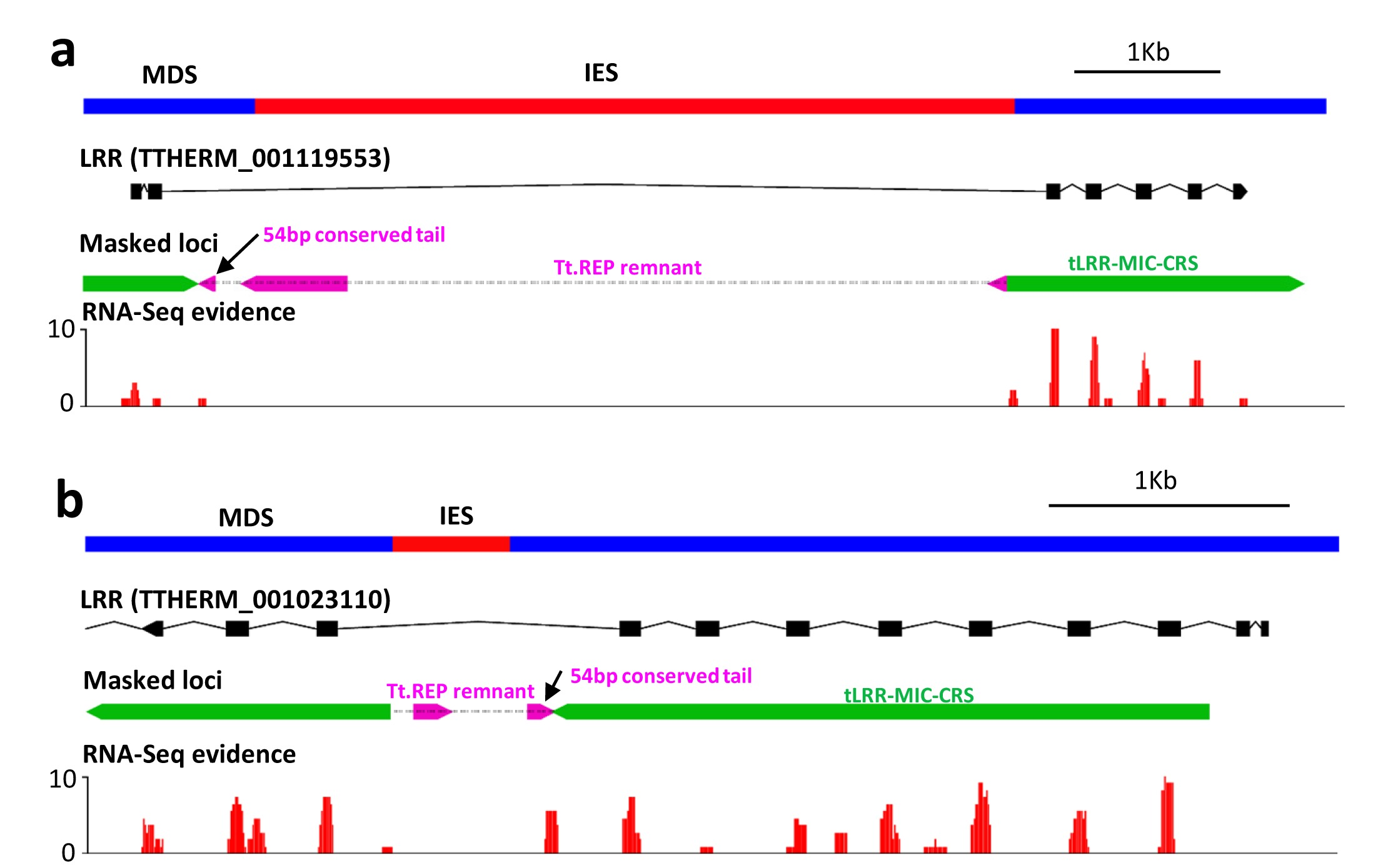

Supplement: S31 Fig — (a) Gene TTHERM_001119553; (b) gene TTHERM_001023110. For each panel: top level: MIC DNA segment: IES (red) and MDS (blue). Second level: gene model. Dark boxes: 90-bp exons. Angle brackets: introns. The longest angle bracket includes intron MDS sequence, and REP remnant sequence that will be excised as IES during MAC development. Third level: green: tLRR-MIC-CRS-masked segment; pink: REP element remnant, including its conserved 54-bp “tail” (green arrow). Fourth level: RNA-Seq evidence. CRS, consensus repeat sequence; LRR, leucine-rich repeat; MAC, macronucleus; MDS, MAC-destined sequence; MIC, micronucleus; RNA-Seq, RNA sequencing. (TIF) [file pbio.3000294.s031.tif]

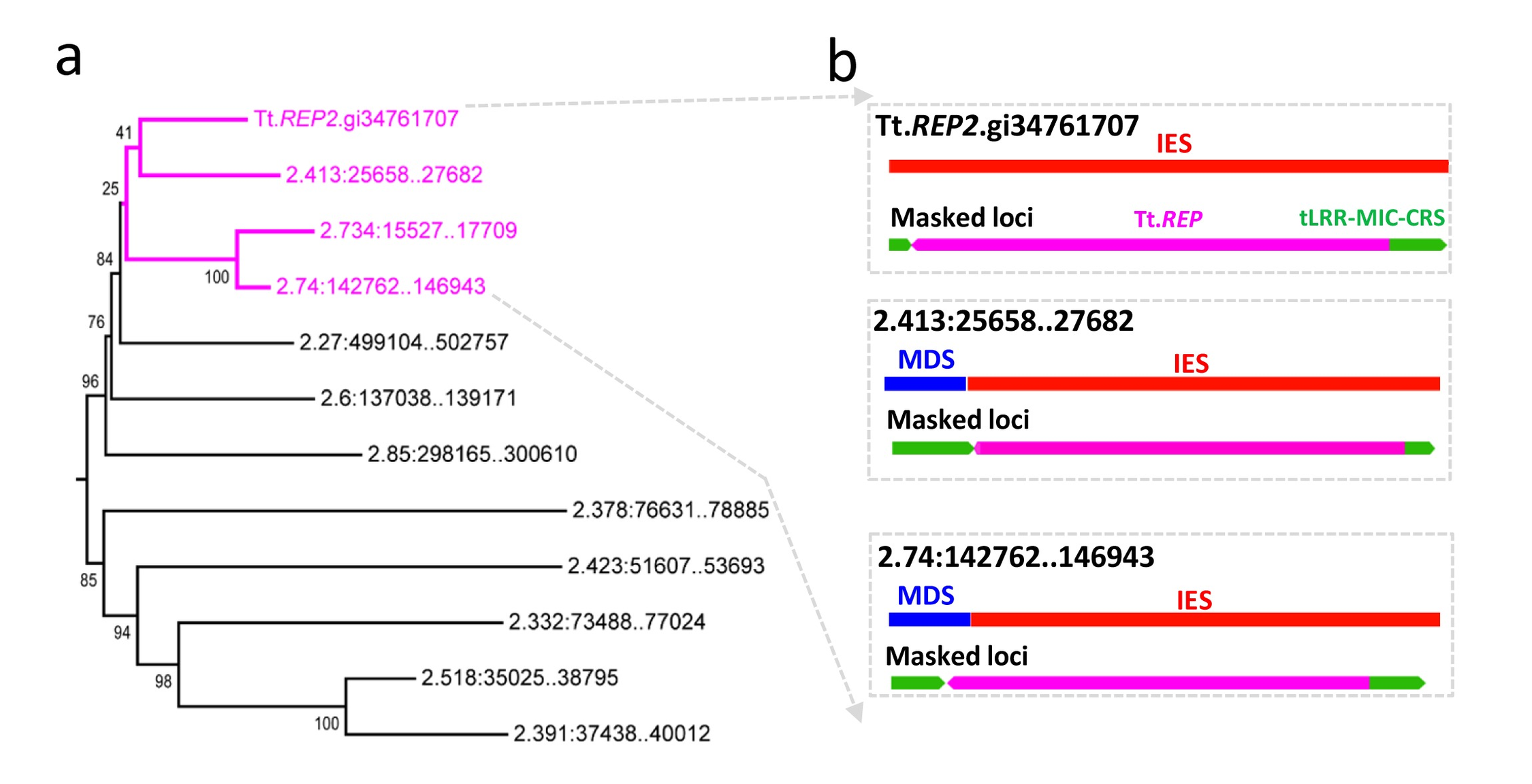

Supplement: S32 Fig — (a) Phylogenetic tree of homologs of Tt.REP2 identified through a BLAST search. Tt.REP2 is a previously identified, functional copy of the T. thermophila non-LTR REP retrotransposon [33]. MIC genome supercontig coordinates are shown for each copy. Tt.REP copies potentially representing the most recent retro-transposition events are colored pink. (b) Examples illustrating in detail the physical relationship between 3 Tt.REP copies highlighted in (a) and tLRR-MIC-CRS-masked sequences in the MIC genome. Note that Tt.REP copy at supercontig location 2.734:15527..17709 is not included in panel b because is at the end of a small, incomplete scaffold, whose flanking sequence, likely repetitive, remains unassembled. CRS, consensus repeat sequence; LRR, leucine-rich repeat; non-LTR, Non-long terminal repeat; MIC, micronucleus. (TIF) [file pbio.3000294.s032.tif]

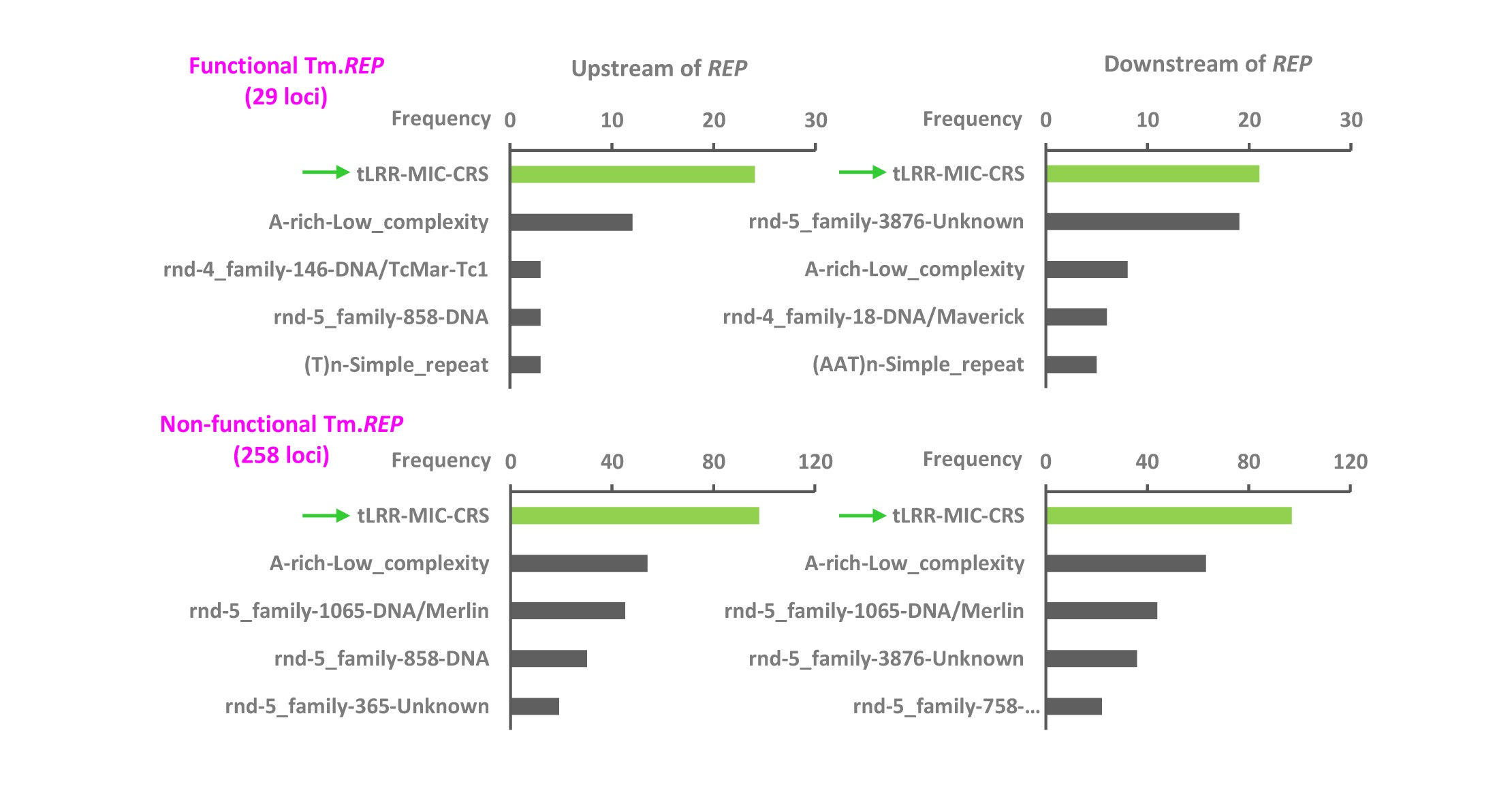

Supplement: S33 Fig — Green bar, tLRR-MIC-CRS-masked loci; black bar, loci masked by other repeat families or low complexity sequences, listed in order of incidence. A Tm.REP copy is considered functional if it contains both intact ORF1 and ORF2 and nonfunctional if it lacks one or both intact ORFs. Numerical data underlying this figure are listed in S2 Data. CRS, consensus repeat sequence; LRR, leucine-rich repeat; MIC, micronucleus. (TIF) [file pbio.3000294.s033.tif]

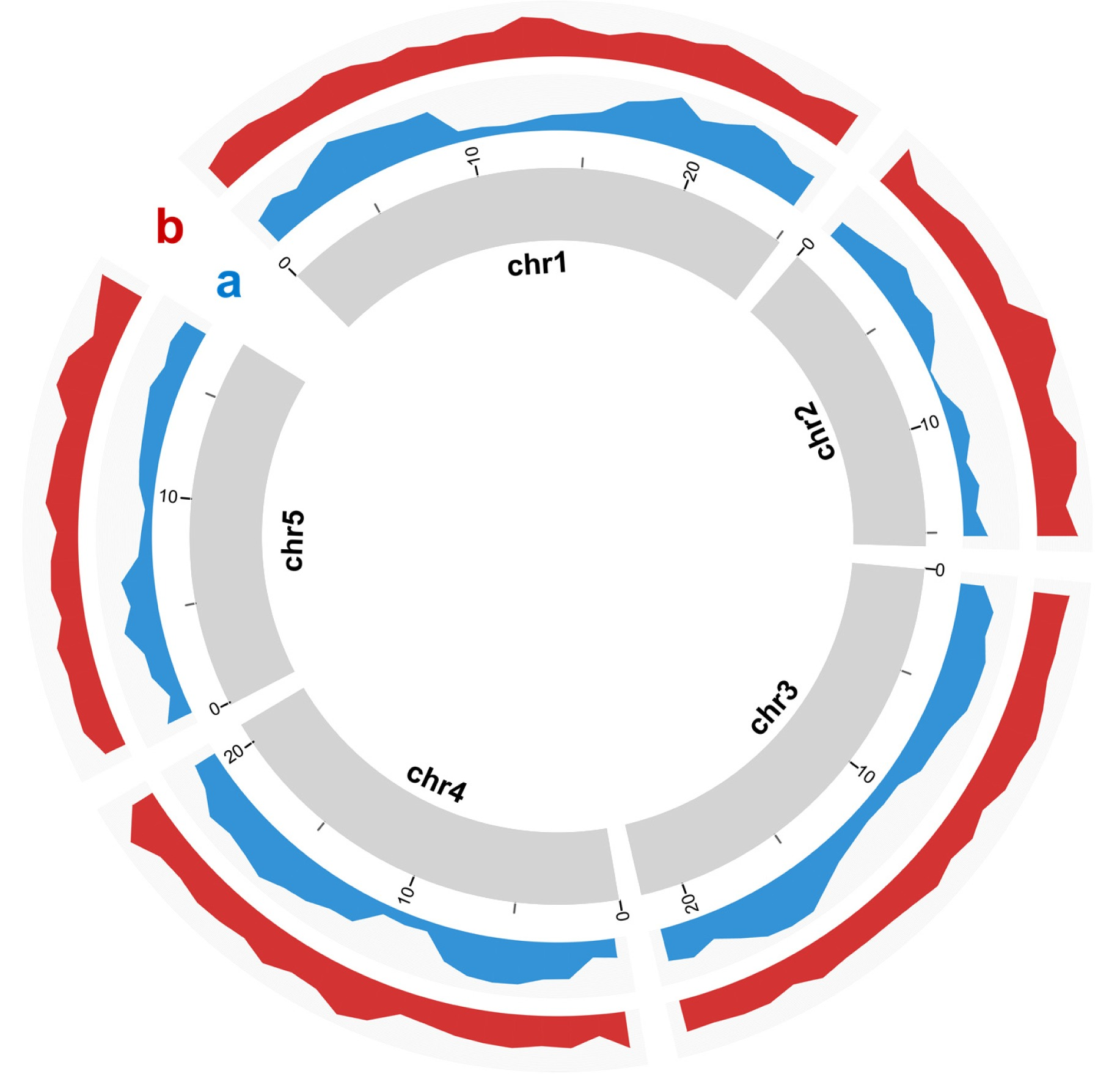

Supplement: S34 Fig — MAC-destined DNA of MIC chromosomes was divided into approximately 1 Mb bins. (a) Density distribution of the 6,052 one-to-one orthologs; y-axis is the number of genes. (d) Distribution of Ka/Ks ratios of 6,052 one-to-one orthologs, plotted as median value of each bin. The Ka/Ks were calculated using codeml (runmode = 0) in PAML (maximum likelihood method) based on the phylogenomic tree in Fig 1. Significant higher Ka/Ks values were found between pericentromeric bins and middle arm bins in chr1, 2, and 4 (Mann Whitney U test, p < 0.01). MAC, macronucleus; MIC, micronucleus; PAML, phylogenetic analysis by maximum likelihood. (TIF) [file pbio.3000294.s034.tif]

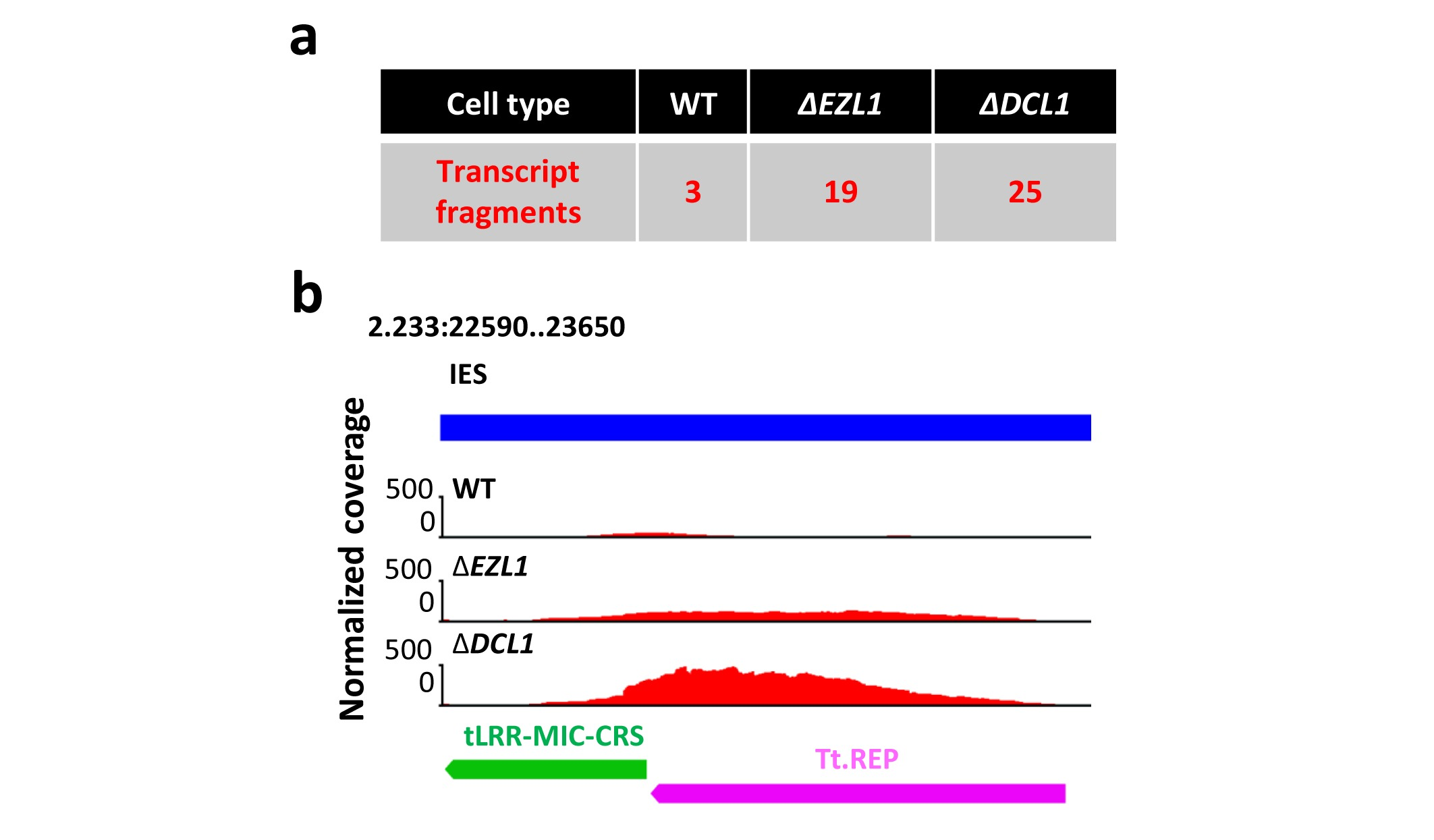

Supplement: S35 Fig — (a) Some de novo–assembled transcript fragments show sequence identity to both Tt.REP and tLRR-MIC-CRS-masked sequences (containing LRR repeats) in cells deficient in the RNAi (ΔDCL1) or Polycomb (ΔEZL1) repression pathways. These pathways are required for the transcriptional silencing—and ultimate excision—of TEs and other IESs during MAC development in T. thermophila. (b) RNA-Seq evidence for tLRR-MIC-CRS-masked sequence and Tt.REP co-transcription in RNAi (ΔDCL1) and Polycomb repression (ΔEZL1) pathway-deficient conjugating cells. Note that the sequence reads are essentially absent when REP is silenced in wild-type conjugating cells. CRS, consensus repeat sequence; IES, internal eliminated sequence; LRR, leucine-rich repeat; MAC, macronucleus; MIC, micronucleus; RNAi, RNA interference; RNA-Seq, RNA sequencing; TE, transposable element. (TIF) [file pbio.3000294.s035.tif]

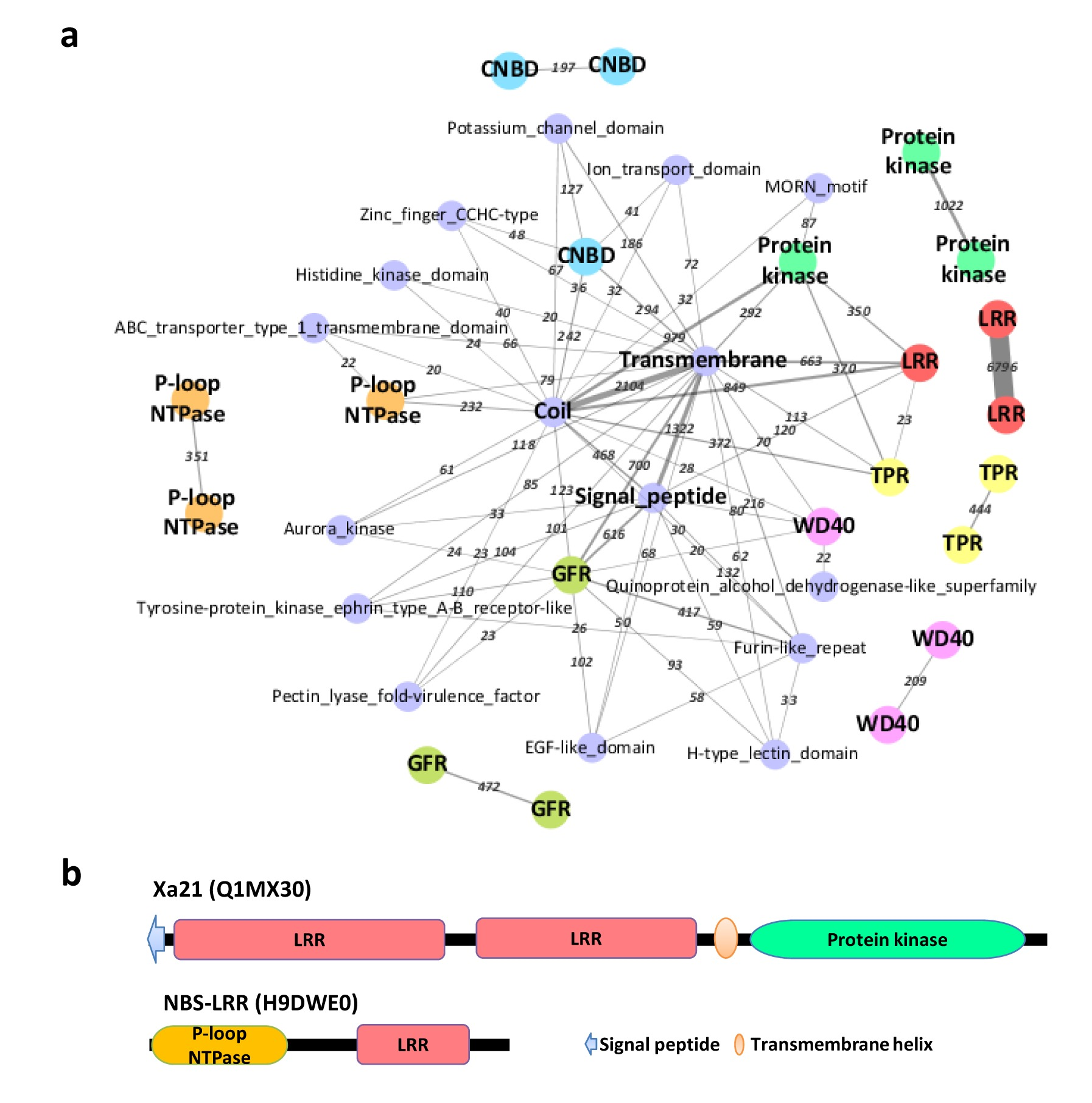

Supplement: S36 Fig — (a) Protein domain architecture for Tetrahymena species-specific genes, presented as a network. Each node represents a specific domain type, and lines represent links between 2 domains within a gene. The width of each line indicates the number, which is also written on the line. LRR, TPR, WD40, PK, CNBD, GFR, and P-loop NTPase domains are shown in different colors, and lines connecting the same color of node represent genes containing only this domain (e.g., an LRR–LRR connection indicates genes that only contain the LRR domain). Coiled-coil (Coil), transmembrane helix (Transmembrane), and signal peptide (Signal_peptide) structures are also included to illustrate that other domains are often associated with the 7 most frequent domains in species-specific genes. (b) Domain architectures of 2 innate immunity LRR genes previously reported in plants. Xa21 (UniProt ID: Q1MX30) is a receptor kinase-like protein in Oryza sativa subsp. Japonica. NBS-LRR (represented as H9DWE0) is a class of proteins containing both P-loop NTPase and LRR domains in O. sativa subsp. Indica. CNBD, cyclic nucleotide-binding domain; GFR, growth factor receptor; LRR, leucine-rich repeat; P-loop NTPase, P-loop-containing nucleoside triphosphate hydrolase; PK, protein kinase; TPR, tetratricopeptide repeat. (TIF) [file pbio.3000294.s036.tif]

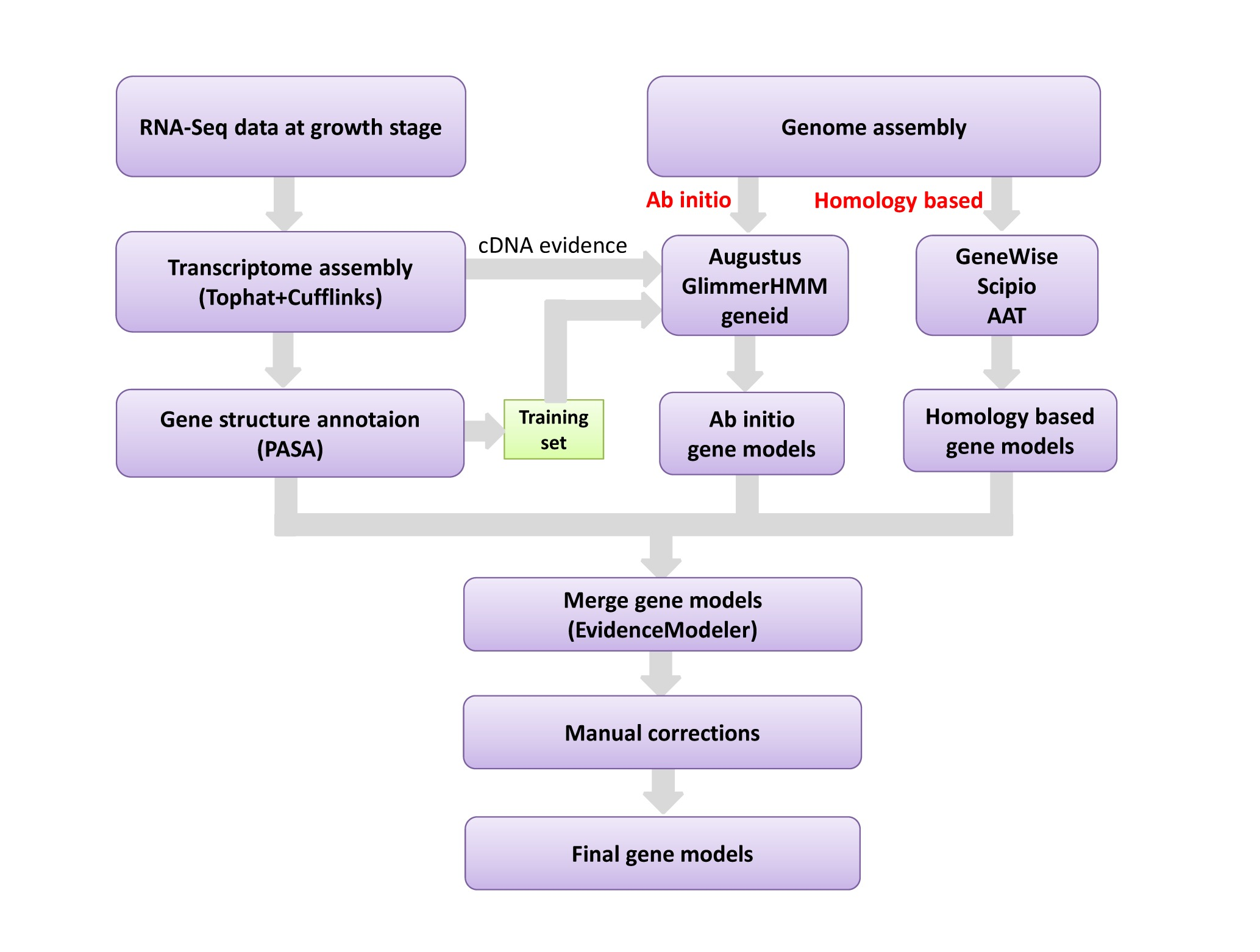

Supplement: S37 Fig — Both ab initio– and homology-based methods were used for gene prediction. Assembled RNA-Seq data were used to generate training gene sets for ab initio gene predictions and were also incorporated as cDNA evidence. EvidenceModeler was used to generate a set of gene models combining evidence from all gene prediction programs. Final predicted gene sets were generated after a few manual corrections. RNA-Seq, RNA sequencing. (TIF) [file pbio.3000294.s037.tif]

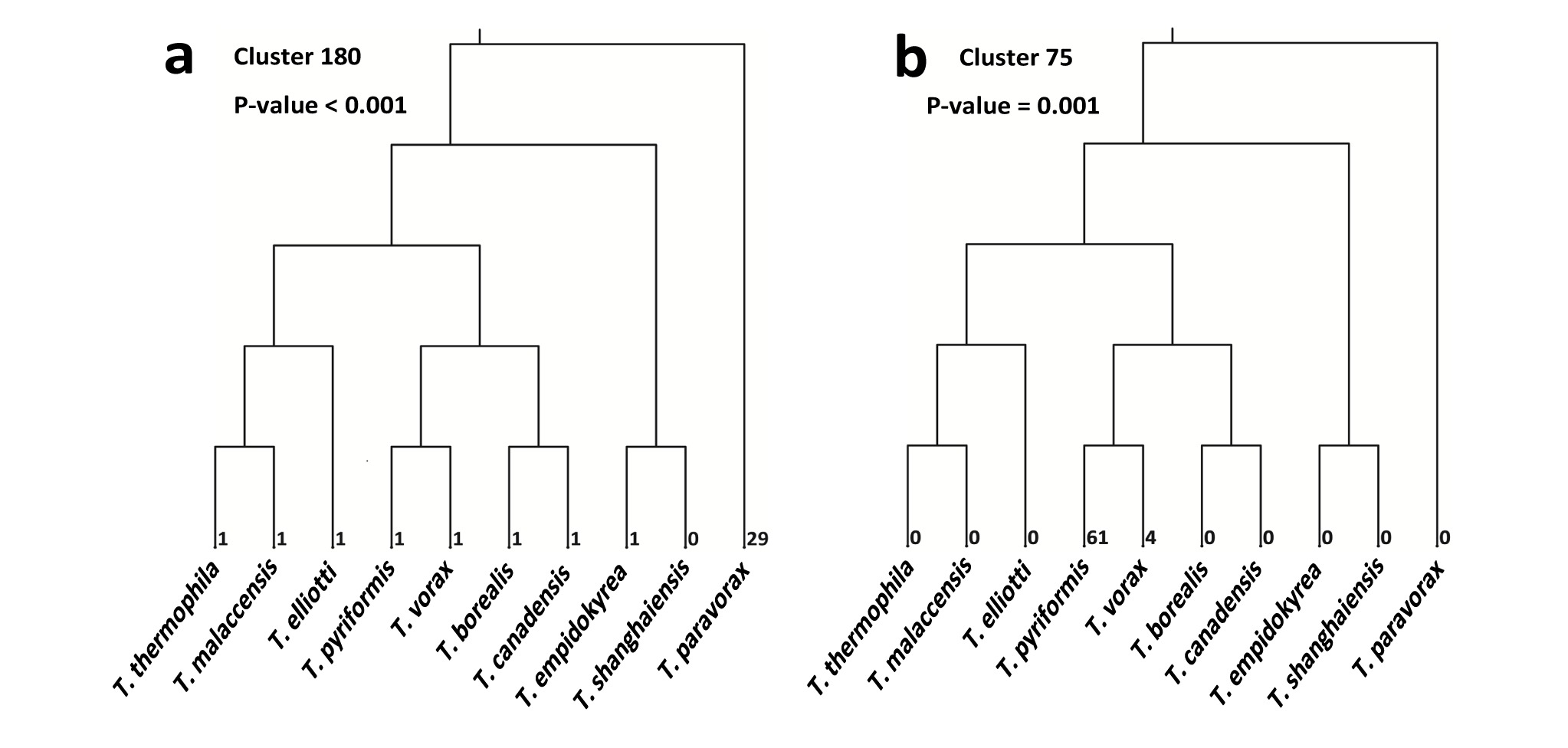

Supplement: S38 Fig — The number of inparalogs in each species is shown above the species name. (a) Category IX cluster 180 has undergone extensive expansion only in T. paravorax (29 genes). (b) Category II cluster 75 has undergone specific expansion only in T. pyriformis (61 genes). (TIF) [file pbio.3000294.s038.tif]

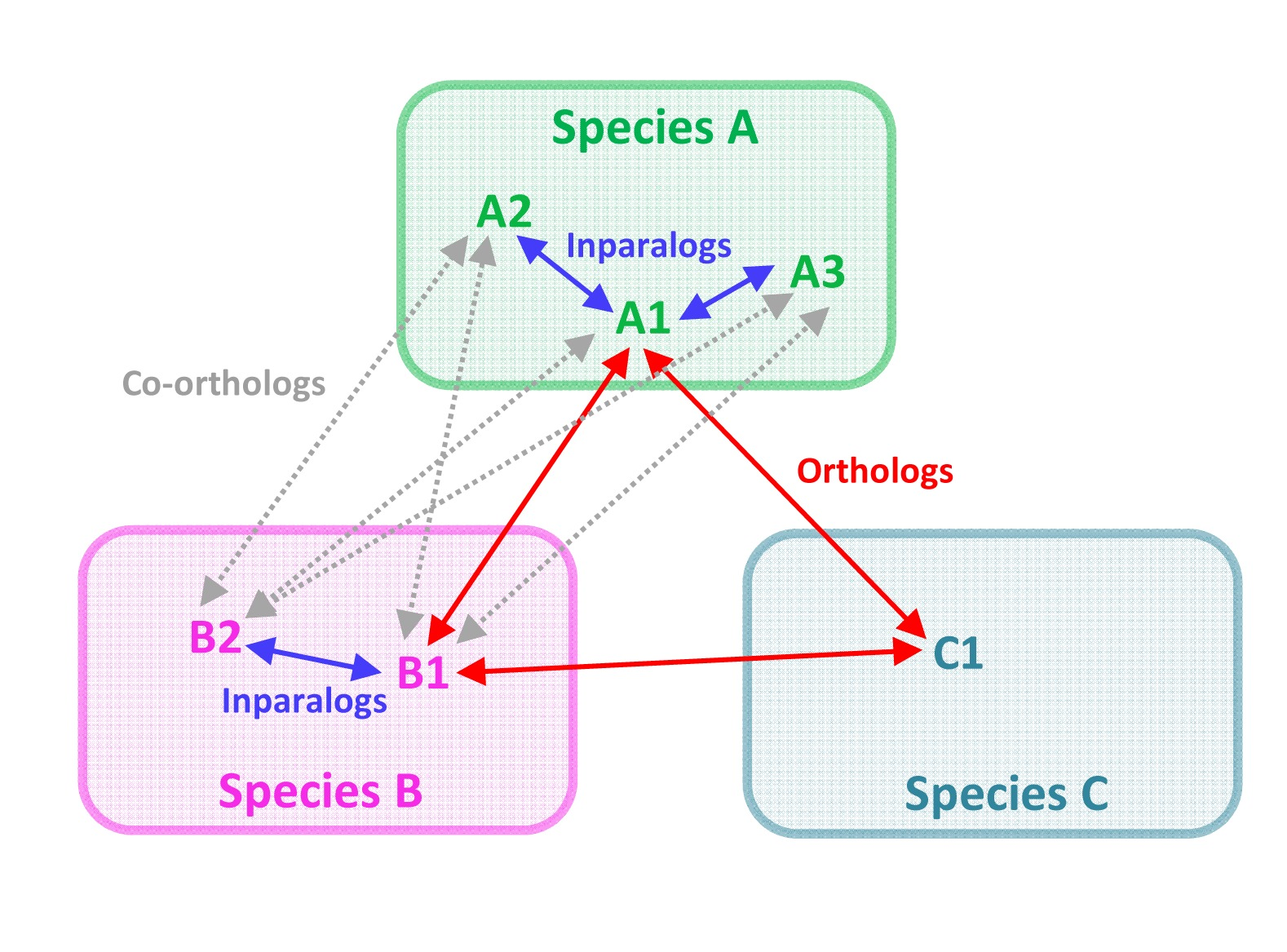

Supplement: S39 Fig — OrthoMCL first identified reciprocal best blast hits between species (“true” orthologs: A1, B1, and C1) and then assigned, to the same ortholog group, genes that gave reciprocal better within-species blast hits to any of those genes (rest of the genes shown in the figure). Although OrthoMCL clusters are usually referred to as ortholog groups, pairs of genes within a cluster may be true orthologs (red arrows), inparalogs (if they are in the same species, blue arrow), or co-orthologs (if they are in different species, dotted black arrows). Orthologs are genes that are conserved between species. Inparalogs are members of a gene expansion within a certain species or lineage; they can be species-specific or very recently duplicated lineage-specific genes. (TIF) [file pbio.3000294.s039.tif]
