# Supplementary material for: Hidden genomic evolution in a morphospecies—The landscape of rapidly evolving genes in Tetrahymena
Source: PLoS Biol. 2019 Jun 3;17(6):e3000294. doi: 10.1371/journal.pbio.3000294 (PMC6564038; doi:10.1371/journal.pbio.3000294)
Supplement: S1 Table — (DOCX) [file pbio.3000294.s040.docx]

**S1 Table. Morphological characters of ten *Tetrahymena* species.**

| **Species** | **Life cycle** | **MIC** | **Other known features** | **CVP number** | **CR number** | **Cell length (um)** | **Cell width (um)** |
| --- | --- | --- | --- | --- | --- | --- | --- |
| *T. thermophila* | F | Y | / | 2-3 | 16-19 | 44-59 | 28-40 |
| *T. malaccensis* | F | Y | / | 1-4 | 16-24 | 45-68 | 26-36 |
| *T. elliotti* | F | Y | / | 2 | 12-17 | 32-52 | 16-36 |
| *T. pyriformis* | F | N | / | 2 | 13-18 | 48-73 | 23-26 |
| *T. vorax* | F | N | Cyst & Oral transf | 2-3 | 18-20 | 48-66 | 27-30 |
| *T. borealis* | F | Y | / | 2 | 15-18 | 45-65 | 26-39 |
| *T. canadensis* | F | Y | / | 2 | 18-20 | 40-48 | 28-34 |
| *T. empidokyrea* | P | Y | / | / | 17-20 | 40-53 | 22-34 |
| *T. shanghaiensis* | F | Y | / | 2 | 16-21 | 48-56 | 24-29 |
| *T. paravorax* | F | Y | Oral trans | 2 | 19-22 | 32-48 | 27-40 |

Life cycle: F, free living; P, facultative parasitic. MIC: Y, yes; N, no. NA, not available. Oral transf, microstome (small oral apparatus) to macrostome (large oral apparatus) transformation. Cyst: encystment and excystment. CVP, contractile vacuole pore. CR, ciliary row.
