# Supplementary material for: Hidden genomic evolution in a morphospecies—The landscape of rapidly evolving genes in Tetrahymena
Source: PLoS Biol. 2019 Jun 3;17(6):e3000294. doi: 10.1371/journal.pbio.3000294 (PMC6564038; doi:10.1371/journal.pbio.3000294)
Supplement: S2 Table — (DOCX) [file pbio.3000294.s041.docx]

**S2 Table. The seven protein domains that have undergone the most extensive expansions in *Tetrahymena.***

| **Species** | **LRR** | **TPR** | **WD40** | **PK** | **CNBD** | **GFR** | **P-loop NTPase** |
| --- | --- | --- | --- | --- | --- | --- | --- |
| *T. thermophila* | 1874 | 488 | 665 | 1142 | 623 | 755 | 920 |
| *T. malaccensis* | 1936 | 595 | 651 | 1396 | 608 | 741 | 941 |
| *T. elliotti* | 1369 | 453 | 494 | 1088 | 536 | 481 | 867 |
| *T. pyriformis* | 2218 | 914 | 591 | 1620 | 596 | 796 | 1037 |
| *T. vorax* | 1341 | 787 | 563 | 1387 | 543 | 604 | 994 |
| *T. borealis* | 1278 | 508 | 546 | 1092 | 539 | 666 | 838 |
| *T. canadensis* | 1439 | 641 | 598 | 1379 | 599 | 799 | 985 |
| *T. empidokyrea* | 479 | 354 | 461 | 976 | 527 | 321 | 823 |
| *T. shanghaiensis* | 927 | 509 | 531 | 1032 | 529 | 731 | 920 |
| *T. paravorax* | 1713 | 607 | 668 | 1209 | 700 | 979 | 957 |
| *Total* | 14,574 | 5856 | 5768 | 12,321 | 5800 | 6873 | 9282 |

The number of genes including at least one copy of each domain is shown.
