# Supplementary material for: Hidden genomic evolution in a morphospecies—The landscape of rapidly evolving genes in Tetrahymena
Source: PLoS Biol. 2019 Jun 3;17(6):e3000294. doi: 10.1371/journal.pbio.3000294 (PMC6564038; doi:10.1371/journal.pbio.3000294)
Supplement: S3 Table — LRR, leucine-rich repeat. (DOCX) [file pbio.3000294.s042.docx]

**S3 Table. Intron phase among LRR genes with only one or two 90-bp exon(s).**

| **Group 2 LRR genes containing only one internal 90bp exon** | | | | | |
| --- | --- | --- | --- | --- | --- |
| **Species** | **Phase0** | **Phase1** | **Phase2** | **Total** | **% Phase2** |
| *T. thermophila* | 2 | 4 | 45 | 51 | 88.2 |
| *T. malaccensis* | 2 | 2 | 49 | 53 | 92.45 |
| *T. elliotti* | 1 | 2 | 42 | 45 | 93.3 |
| *T. pyriformis* | 4 | 2 | 124 | 130 | 95.4 |
| *T. vorax* | 1 | 3 | 129 | 133 | 97.0 |
| *T. borealis* | 2 | 2 | 25 | 29 | 86.2 |
| *T. canadensis* | 1 | 1 | 54 | 56 | 96.4 |
| *T. empidokyrea* | 1 | 2 | 29 | 32 | 90.6 |
| *T. shanghaiensis* | 0 | 2 | 44 | 46 | 95.7 |
| *T. paravorax* | 1 | 4 | 77 | 82 | 93.9 |
| **Total** | 15 | 24 | 618 | 657 | 94.1 |
| **Percent** | 2.3 | 3.7 | 94.1 |  |  |
|  | | | | | |
| **Group 2 LRR genes containing two internal 90bp exons** | | | | | |
| **Species** | **Phase0** | **Phase1** | **Phase2** | **Total** | **% Phase2** |
| *T. thermophila* | 0 | 2 | 104 | 106 | 98.1 |
| *T. malaccensis* | 0 | 0 | 100 | 100 | 100.00 |
| *T. elliotti* | 0 | 0 | 68 | 68 | 100.0 |
| *T. pyriformis* | 1 | 1 | 308 | 310 | 99.4 |
| *T. vorax* | 3 | 3 | 208 | 214 | 97.2 |
| *T. borealis* | 1 | 0 | 73 | 74 | 98.6 |
| *T. canadensis* | 1 | 1 | 96 | 98 | 98.0 |
| *T. empidokyrea* | 0 | 1 | 51 | 52 | 98.1 |
| *T. shanghaiensis* | 0 | 0 | 80 | 80 | 100.0 |
| *T. paravorax* | 0 | 0 | 138 | 138 | 100.0 |
| **Total** | 6 | 8 | 1,226 | 1,240 | 98.9 |
| **Percent** | 0.5 | 0.6 | 98.9 |  |  |
|  | | | | | |
| **Group 3 LRR genes containing only one internal 90bp exon** | | | | | |
| **Species** | **Phase0** | **Phase1** | **Phase2** | **Total** | **% Phase2** |
| *T. thermophila* | 0 | 1 | 45 | 46 | 97.8 |
| *T. malaccensis* | 0 | 0 | 41 | 41 | 100.00 |
| *T. elliotti* | 1 | 1 | 60 | 62 | 96.8 |
| *T. pyriformis* | 0 | 0 | 56 | 56 | 100.0 |
| *T. vorax* | 0 | 2 | 61 | 63 | 96.8 |
| *T. borealis* | 0 | 0 | 8 | 8 | 100.0 |
| *T. canadensis* | 0 | 1 | 36 | 37 | 97.3 |
| *T. empidokyrea* | NA | NA | NA | NA | NA |
| *T. shanghaiensis* | 0 | 0 | 29 | 29 | 100.0 |
| *T. paravorax* | 0 | 0 | 67 | 67 | 100.0 |
| **Total** | 1 | 5 | 403 | 409 | 98.5 |
| **Percent** | 0.2 | 1.2 | 98.5 |  |  |
|  | | | | | |
| **Group 3 LRR genes containing two internal 90bp exons** | | | | | |
| **Species** | **Phase0** | **Phase1** | **Phase2** | **Total** | **% Phase2** |
| *T. thermophila* | 0 | 3 | 97 | 100 | 97.0 |
| *T. malaccensis* | 0 | 0 | 88 | 88 | 100.00 |
| *T. elliotti* | 0 | 0 | 136 | 136 | 100.0 |
| *T. pyriformis* | 1 | 1 | 118 | 120 | 98.3 |
| *T. vorax* | 1 | 4 | 97 | 102 | 95.1 |
| *T. borealis* | 0 | 0 | 24 | 24 | 100.0 |
| *T. canadensis* | 0 | 1 | 107 | 108 | 99.1 |
| *T. empidokyrea* | NA | NA | NA | NA | NA |
| *T. shanghaiensis* | 0 | 1 | 115 | 116 | 99.1 |
| *T. paravorax* | 1 | 1 | 128 | 130 | 98.5 |
| **Total** | 3 | 11 | 910 | 924 | 98.5 |
| **Percent** | 0.3 | 1.2 | 98.5 |  |  |

Note that *T. empidokyrea* lack group III LRR genes.
