# Supplementary material for: Hidden genomic evolution in a morphospecies—The landscape of rapidly evolving genes in Tetrahymena
Source: PLoS Biol. 2019 Jun 3;17(6):e3000294. doi: 10.1371/journal.pbio.3000294 (PMC6564038; doi:10.1371/journal.pbio.3000294)
Supplement: S4 Table — CRS, consensus repeat sequence; LRR, leucine-rich repeat; MAC, macronucleus. (DOCX) [file pbio.3000294.s043.docx]

**S4 Table. Number of CRSs masking Group III LRR genes identified in the MAC genome of each species.**

| **Species** | **Total CRS** | **CRS masking**  **Group III LRR genes** |
| --- | --- | --- |
| *T. thermophila* | 27 | 8 |
| *T. malaccensis* | 35 | 10 |
| *T. elliotti* | 14 | 5 |
| *T. pyriformis* | 51 | 5 |
| *T. vorax* | 43 | 5 |
| *T. borealis* | 13 | 4 |
| *T. canadensis* | 34 | 4 |
| *T. empidokyrea* | 7 | 0* |
| *T. shanghaiensis* | 12 | 3 |
| *T. paravorax* | 58 | 6 |

* *T. empidokyrea* lacks group III LRR genes.
