# Supplementary material for: Hidden genomic evolution in a morphospecies—The landscape of rapidly evolving genes in Tetrahymena
Source: PLoS Biol. 2019 Jun 3;17(6):e3000294. doi: 10.1371/journal.pbio.3000294 (PMC6564038; doi:10.1371/journal.pbio.3000294)
Supplement: S5 Table — LRR, leucine-rich repeat. (DOCX) [file pbio.3000294.s044.docx]

**S5 Table. The most conserved amino acids of 90-exons in Group II LRR genes in different species.**

| **Pos** | ***T.th*** | ***T.ma*** | ***T.el*** | ***T.py*** | ***T.vo*** | ***T.bo*** | ***T.ca*** | ***T.em*** | ***T.sh*** | ***T.pa*** |
| --- | --- | --- | --- | --- | --- | --- | --- | --- | --- | --- |
| **1** | **N:0.15** | **N:0.16** | **N:0.16** | **N:0.16** | **N:0.17** | **N:0.17** | **N:0.17** | **N:0.19** | **N:0.18** | **N:0.15** |
| **2** | **N:0.70** | **N:0.71** | **N:0.71** | **N:0.75** | **N:0.77** | **N:0.69** | **N:0.77** | **N:0.73** | **N:0.67** | **N:0.81** |
| **3** | **N:0.19** | **Q:0.20** | **Q:0.22** | **N:0.17** | **Q:0.17** | **Q:0.20** | **Q:0.15** | **Q:0.18** | **K:0.18** | **S:0.21** |
| **4** | **I:0.67** | **I:0.67** | **I:0.64** | **I:0.66** | **I:0.70** | **I:0.62** | **I:0.63** | **I:0.63** | **I:0.56** | **I:0.66** |
| **5** | **G:0.29** | **G:0.30** | **G:0.30** | **G:0.38** | **G:0.41** | **G:0.28** | **G:0.37** | **G:0.34** | **G:0.32** | **G:0.51** |
| **6** | **D:0.26** | **D:0.29** | **D:0.28** | **D:0.28** | **D:0.21** | **D:0.28** | **D:0.29** | **D:0.26** | **D:0.30** | **E:0.25** |
| **7** | **E:0.27** | **E:0.26** | **E:0.25** | **E:0.28** | **E:0.24** | **E:0.28** | **E:0.25** | **E:0.30** | **E:0.21** | **N:0.17** |
| **8** | **G:0.63** | **G:0.65** | **G:0.63** | **G:0.73** | **G:0.71** | **G:0.64** | **G:0.72** | **G:0.66** | **G:0.60** | **G:0.81** |
| **9** | **A:0.32** | **A:0.30** | **A:0.24** | **A:0.35** | **A:0.39** | **A:0.29** | **A:0.37** | **A:0.30** | **A:0.23** | **A:0.52** |
| **10** | **K:0.19** | **S:0.20** | **S:0.20** | **S:0.31** | **S:0.28** | **S:0.15** | **K:0.18** | **S:0.20** | **E:0.15** | **R:0.35** |
| **11** | **S:0.12** | **S:0.13** | **N:0.13** | **S:0.14** | **G:0.18** | **K:0.14** | **S:0.14** | **E:0.11** | **K:0.13** | **D:0.25** |
| **12** | **L:0.50** | **L:0.53** | **L:0.57** | **L:0.63** | **L:0.63** | **L:0.57** | **L:0.56** | **L:0.58** | **L:0.59** | **L:0.76** |
| **13** | **G:0.28** | **G:0.29** | **G:0.31** | **G:0.41** | **G:0.41** | **G:0.39** | **G:0.42** | **G:0.40** | **G:0.36** | **G:0.60** |
| **14** | **Q:0.16** | **S:0.18** | **S:0.21** | **S:0.15** | **N:0.18** | **Q:0.19** | **S:0.22** | **Q:0.27** | **Q:0.20** | **K:0.24** |
| **15** | **G:0.21** | **G:0.21** | **G:0.19** | **G:0.30** | **G:0.28** | **G:0.30** | **G:0.34** | **G:0.20** | **E:0.18** | **E:0.40** |
| **16** | **L:0.51** | **L:0.59** | **L:0.60** | **L:0.66** | **L:0.61** | **L:0.46** | **L:0.54** | **I:0.50** | **I:0.49** | **L:0.69** |
| **17** | **S:0.22** | **S:0.24** | **S:0.25** | **S:0.18** | **G:0.26** | **S:0.30** | **S:0.21** | **S:0.28** | **S:0.35** | **A:0.33** |
| **18** | **K:0.32** | **K:0.33** | **K:0.33** | **N:0.26** | **K:0.30** | **K:0.35** | **K:0.27** | **K:0.38** | **K:0.42** | **K:0.63** |
| **19** | **C:0.46** | **C:0.44** | **C:0.43** | **C:0.54** | **C:0.50** | **C:0.42** | **C:0.46** | **C:0.45** | **C:0.38** | **C:0.56** |
| **20** | **K:0.25** | **K:0.21** | **K:0.19** | **K:0.17** | **T:0.27** | **K:0.19** | **Q:0.20** | **T:0.20** | **K:0.17** | **S:0.34** |
| **21** | **N:0.47** | **N:0.48** | **N:0.46** | **N:0.49** | **N:0.48** | **N:0.43** | **N:0.35** | **N:0.45** | **N:0.43** | **N:0.61** |
| **22** | **L:0.68** | **L:0.70** | **L:0.73** | **L:0.82** | **L:0.80** | **L:0.79** | **L:0.71** | **L:0.77** | **L:0.76** | **L:0.84** |
| **23** | **T:0.19** | **T:0.19** | **Q:0.19** | **T:0.33** | **T:0.41** | **T:0.25** | **T:0.32** | **N:0.21** | **T:0.29** | **N:0.39** |
| **24** | **S:0.16** | **N:0.19** | **S:0.17** | **S:0.36** | **S:0.27** | **S:0.23** | **S:0.22** | **S:0.32** | **S:0.31** | **S:0.52** |
| **25** | **L:0.82** | **L:0.82** | **L:0.81** | **L:0.85** | **L:0.84** | **L:0.79** | **L:0.79** | **L:0.71** | **L:0.75** | **L:0.87** |
| **26** | **T:0.15** | **T:0.14** | **K:0.14** | **T:0.21** | **T:0.26** | **T:0.18** | **T:0.20** | **T:0.26** | **T:0.19** | **T:0.27** |
| **27** | **L:0.70** | **L:0.68** | **L:0.68** | **L:0.76** | **L:0.77** | **L:0.70** | **L:0.70** | **L:0.68** | **L:0.66** | **L:0.85** |
| **28** | **N:0.28** | **N:0.25** | **N:0.26** | **N:0.26** | **N:0.28** | **N:0.27** | **N:0.27** | **N:0.27** | **N:0.24** | **N:0.33** |
| **29** | **L:0.66** | **L:0.66** | **L:0.64** | **L:0.68** | **L:0.68** | **L:0.67** | **L:0.69** | **L:0.66** | **L:0.63** | **L:0.80** |

T.th=*T. thermophila*; T.ma=*T. malaccensis*; T.el=*T. elliotti*; T.py=*T. pyriformis*; T.vo=*T. vorax*; T.bo=*T. borealis*; T.ca=*T. canadensis*; T.em=*T. empidokyrea*; T.sh=*T. shanghaiensis*; T.pa=*T. paravorax*; For each position, the most conserved amino acid and its fraction were shown. Rows with red font indicate that the most conserved amino acids are different in *T. paravorax* compared to other species.
