# Supplementary material for: Hidden genomic evolution in a morphospecies—The landscape of rapidly evolving genes in Tetrahymena
Source: PLoS Biol. 2019 Jun 3;17(6):e3000294. doi: 10.1371/journal.pbio.3000294 (PMC6564038; doi:10.1371/journal.pbio.3000294)
Supplement: S6 Table — (DOCX) [file pbio.3000294.s045.docx]

**S6 Table. *Tetrahymena* strains used in this study and their source.**

| **Species** | **Strain ID and source** |
| --- | --- |
| *T. malaccensis* | TSC_SD01608 (*Tetrahymena* stock center) |
| *T. elliotti* | TSC_SD01607 (*Tetrahymena* stock center) |
| *T. pryformis* | Used in previous study (Ye *et al.* 2014, Aquatic Toxicology) |
| *T. vorax* | 30421 (ATCC) |
| *T. borealis* | TSC_SD01609 (*Tetrahymena* stock center) |
| *T. canadensis* | 30770 (ATCC) |
| *T. empidokyrea* | 50595 (ATCC) |
| *T. shanghaiensis* | 205039 (ATCC) |
| *T.paravorax* | 205177 (ATCC) |
