# Supplementary material for: Hidden genomic evolution in a morphospecies—The landscape of rapidly evolving genes in Tetrahymena
Source: PLoS Biol. 2019 Jun 3;17(6):e3000294. doi: 10.1371/journal.pbio.3000294 (PMC6564038; doi:10.1371/journal.pbio.3000294)
Supplement: S7 Table — LRR, leucine-rich repeat; PK, protein kinase. (DOCX) [file pbio.3000294.s046.docx]

**S7 Table. Domain accessions used for LRR and protein kinase gene identification.**

| **Database** | **Accession** | **Description** |
| --- | --- | --- |
| Pfam | PF00069 | Protein kinase domain |
| Pfam | PF02816 | Alpha-kinase family |
| Pfam | PF07714 | Protein tyrosine kinase |
| Pfam | PF07568 | Histidine kinase |
| Pfam | PF12282 | Signal transduction histidine kinase |
| Pfam | PF05445 | Poxvirus serine/threonine kinase |
| Pfam | PF06580 | Histidine |
| Pfam | PF07536 | HWE histidine kinase |
| Pfam | PF07730 | Histidine kinase |
| Pfam | PF10494 | Serine-threonine protein kinase |
| Pfam | PF14381 | Ethylene-responsive prtein kinase Le-CTR1 |
| Pfam | PF15785 | Serine/threonine-protein kinase smg-1 |
| Pfam | PF00512 | His Kinase A (phosphoacceptor) domain |
| Pfam | PF01163 | RIO1 family |
| Prosite | PS50109 | Histidine kinase domain |
| Prosite | PS50011 | Protein kinase domain |
| Prosite | PS51158 | Alpha-type protein kinase |
| Prosite | PS00108 | Serine/Threonine protein kinases active-site |
| Prosite | PS00109 | Tyrosine protein kinases specific active-site |
| SMART | SM00090 | RIO-like kinase |
| SMART | SM00133 | Extension to Ser/Thr-type protein kinases |
| SMART | SM00219 | Tyrosine kinase, catalytic domain |
| SMART | SM00220 | Serine/Threonine protein kinases, catalytic domain |
| SMART | SM00221 | Protein kinase; unclassified specificity. |
| SMART | SM00811 | Alpha-kinase family |
| SMART | SM00911 | HWE histidine kinase |
| SMART | SM00388 | His Kinase A (phosphoacceptor) domain |
|  |  |  |
| InterPro | IPR001611 | Leucine-rich repeat |
| InterPro | IPR006553 | Leucine-rich repeat, cysteine-containing subtype |
| InterPro | IPR026906 | Leucine rich repeat 5 |
| InterPro | IPR025875 | Leucine rich repeat 4 |
| InterPro | IPR032675 | Leucine-rich repeat domain, L domain-like |
| InterPro | IPR003591 | Leucine-rich repeat, typical subtype |
