# Supplementary material for: Hidden genomic evolution in a morphospecies—The landscape of rapidly evolving genes in Tetrahymena
Source: PLoS Biol. 2019 Jun 3;17(6):e3000294. doi: 10.1371/journal.pbio.3000294 (PMC6564038; doi:10.1371/journal.pbio.3000294)
Supplement: S1 Text — (DOCX) [file pbio.3000294.s047.docx]

**Supplementary Methods**

**Cell strains, growth conditions, and morphological analysis**

For all species, 18S rDNA sequences obtained from either the genome or transcriptome assembly were used to confirm species identity. *T*. *canadensis* obtained from the ATCC was labeled *T*. *rostrata*, but its 18S rDNA and cloned *COX1* sequences had high homology to *T*. *canadensis* sequences, indicating that the *T. rostrata* species had been misidentified. This species is currently named *T*. *sp* in the ATCC (ID: 30770).

Cells were grown in SPP medium [[1](#_ENREF_1)] and harvested at a density of 150,000–250,000 cells/ml. For morphological analysis, cells of all ten *Tetrahymena* species were harvested at the vegetative growth stage by centrifugation at 360 *g* for 3 min at room temperature (about 25°C). The supernatant was removed, 1 ml 50% formalin was added, tubes were shaken to mix, and the mixture was incubated for 5 min. Cells were then transferred to an embryo dish and stained using the modified silver carbonate impregnation method [[2](#_ENREF_2)]. Photomicrographs of stained specimens were obtained using a Nikon ECLIPSE 80i (800–2000× magnification).

**DNA and RNA isolation and high-throughput sequencing**

The protocol reported by Gorovsky *et al.* [[1](#_ENREF_1)] was followed to isolate *Tetrahymena* nuclei. MACs were purified away from MICs by differential sedimentation, except for amicronucleate species, as this step was unnecessary. MAC:MIC ratios were determined to check MAC purity using DAPI staining. MAC purity was >90% for all samples (most had >95% purity). Since the average DNA copy number of the MAC in an exponentially growing culture is >15-fold greater than that of the MIC, MAC DNA purity in purified samples is even higher. DNA was extracted from purified MACs [[1](#_ENREF_1)] and total RNA was prepared using an RNeasy Protect Cell Mini Kit (Qiagen, Valencia, CA, USA) according to the protocol provided in TetraFGD [[3](#_ENREF_3),[4](#_ENREF_4)].

MAC genomes were sequenced using the Illumina platform. Paired-end (insert size 180–220 bp) and mate-pair (insert size 2–3 kb) libraries were constructed and sequenced using the standard Illumina protocol (https://icom.illumina.com/). Briefly, genomic DNA was fragmented, and appropriately sized fragments were selected. For mate-pair library construction, fragment ends were biotinylated and circularized, and fragments were enriched based on binding to streptavidin beads. Fragment ends were repaired, A-tailed, and ligated to sequencing adaptors. Adaptor-ligated fragments were PCR-amplified using Phusion polymerase, denatured with sodium hydroxide, and diluted in hybridization buffer. Prepared libraries were loaded into flow cells and sequenced.

*T*. *malaccensis* MIC nuclei were isolated by differential centrifugation following established protocols [[5](#_ENREF_5)]. MIC nuclei purity was about ~99.3%. 10kb SMRTbell^TM^ libraries were constructed and sequenced using a PacBio Sequel sequencer. A total of ~15 Gb of long reads were generated. The *T*. *malaccensis* MIC genome was assembled using HGAP4, MECAT, WTDBG, and Miniasm. The assembly with highest base accuracy was selected as the final assembly.

For transcriptome sequencing, Poly-A+ mRNAs were isolated using Dynal magnetic beads (Invitrogen) and fragmented by heating to 94°C. First-strand cDNAs were synthesized using reverse transcriptase and random hexamer primers, and second strands were synthesized with DNA polymerase and random hexamer primers. Double-stranded cDNAs were end-repaired and a single adenosine moiety was added. Illumina adapters were ligated, and gel electrophoresis was used to select DNA fragments sized at about 200 bp. Libraries were PCR-amplified using Phusion polymerase. Cluster formation, primer hybridization, and paired-end sequencing were performed using proprietary reagents according to manufacturer-recommended protocols.

**Genome assembly and repeat masking**

MAC genomes of *Tetrahymena* species other than *thermophila* were sequenced by combining paired-end (short insert size) and mate-paired (long insert size) libraries; this simple strategy has been shown to perform well for another ciliate [[6](#_ENREF_6)]. To construct genome assemblies, paired-end reads and mate-paired reads, obtained by Illumina sequencing for each species, were assembled using SOAPdenovo[[7](#_ENREF_7)] or ALLPATHS-LG [[8](#_ENREF_8)]. Since SOAPdenovo does not use a fixed K-mer, a series of K-mer values (from 33 to 79) were used for assembly. Finally, the assembly with the longest N50 length was selected by removing scaffolds shorter than 1Kb.

Repeat sequences were identified *de novo* using RepeatModeler and the generated consensus sequences library was used to mask the genomes using Repeatmasker (http://www.repeatmasker.org/).

**Gene prediction**

Gene models were predicted using *ab initio* and homology-based methods (S37 Fig). RNA-Seq data for the logarithmic growth stage of *Tetrahymena* were generated to aid gene prediction. For this, RNA-Seq reads were mapped to the assembled reference MAC genome, and transcripts underwent reference-guided assembly using the TopHat and Cufflinks pipeline [[9](#_ENREF_9)]. In the *ab initio* approach, assembled transcripts were validated by aligning putative transcripts onto the assembled genome using PASA [[10](#_ENREF_10)]. The so-called “complete” category of open reading frames (e.g. full length CDS sequences) were extracted as described in the PASA manual (http://pasapipeline.github.io/#A_ComprehensiveTranscriptome), and those “complete” transcripts with good hits in the GenBank non-redundant protein database were used as a high-quality data set for training *ab initio* gene predictors, including Augustus [[11](#_ENREF_11)], GlimmerHMM [[12](#_ENREF_12)], and geneid [[13](#_ENREF_13)]. As Augustus software could accept cDNA or protein evidence, assembled transcripts were also used as cDNA evidence in this program. For the homology-based method, all protein sequences of genome-sequenced ciliates were used as a reference database, and assembled *Tetrahymena* genomes were aligned to this database for gene prediction using GeneWise [[14](#_ENREF_14)], ATT [[15](#_ENREF_15)], and Scipio [[16](#_ENREF_16)]. Finally, an integrated set of gene models was created using Evidence Modeler [[17](#_ENREF_17)] by merging all predicted gene models and the RNA-Seq transcripts, followed by minor manual corrections.

**Gene annotation**

Homologs of predicted *Tetrahymena* genes were BLAST-searched against the NCBI non-redundant protein database, and protein domains and potential functional classifications were annotated using InterProScan (which integrates a series of search applications and databases) [[18](#_ENREF_18)]. LRR and protein kinase domain genes were identified by domain annotation in InterProScan, which includes information from Pfam, Prosite, SMART, and Gene3D databases (S7 Table). Protein kinase subcategories were based on the *T*. *thermophila* kinome classification (http://kinase.com/web/current/kinbase/browser/SpeciesID/10092). In detail, all predicted protein sequences of *Tetrahymena* were searched against the profiles of all protein kinase families using HMMER [[19](#_ENREF_19)] with E-value cutoff 0.01, and protein kinases were classified according to the top profile hit.

**Ortholog group identification**

Comparative genomic approaches are increasingly used for both evolutionary and functional analyses, and the concepts of orthology and paralogy (which originated in the field of molecular systematics [[20](#_ENREF_20)]) are being applied to functional characterization and classification for whole-genome comparisons [[21-23](#_ENREF_21)]. Ortholog groups (clusters) were annotated using OrthoMCL [[24](#_ENREF_24)], which provides the best overall balance of sensitivity and specificity for multiple species ortholog clustering [[25](#_ENREF_25)]. The important OrthoMCL parameter, inflation index, was set at 1.5 to balance sensitivity and selectivity as used in OrthoMCL-DB construction [[26](#_ENREF_26)]. OrthoMCL groups proteins into ortholog groups (clusters) containing orthologs, inparalogs, and co-orthologs (S39 Fig). Orthologs are defined by reciprocal best BLAST hits between different species (between-species hits) [[24](#_ENREF_24)] and represent conserved genes. Inparalogs are defined as reciprocal better BLAST hits [[24](#_ENREF_24)] within-species compared with between-species hits and represent recently duplicated genes, i.e. species-specific gene expansion of genes. To investigate the significantly expanded ortholog groups in different species or lineages, gene number changes among different species within an ortholog group were analyzed using CAFE 3.0 [[27](#_ENREF_27)] based on the phylogenomic tree.

**Comparative genomics analysis**

The MAC genome of the *T. thermophila* model species (as sequenced by the Sanger method) was used as the reference genome for analyzing gene synteny with other *Tetrahymena* species. The assembled scaffolds for each genome were aligned to the reference genome using LASTZ [[28](#_ENREF_28)]; if multiple homologous scaffolds were found for a reference scaffold, then homologous scaffolds were identified and arranged as aligned coordinates (and vice versa). Since some scaffolds have low sequence similarity to reference gene sequences, especially those of species more distantly related to *T*. *thermophila*, ortholog information based on protein sequences was used to verify homologous scaffolds, with manual correction. Based on the homologous scaffolds identified for all ten species, gene synteny and collinearity were scanned and detected using MCScanx [[29](#_ENREF_29)], and combined with the OrthoMCL results. Finally, gene synteny maps were generated using genoPlotR [[30](#_ENREF_30)].

**Phylogenomic analysis and divergence time estimation**

A total of 198 one-to-one ortholog groups were identified in the ten *Tetrahymena* species and other ciliates (including *Ichthyophthirius multifiliis*, *Pseudocohnilembus persalinus*, *Paramecium tetraurelia*, *Oxytricha trifallax* and *Stylonychia mytilus*). To construct the phylogenomic tree for the ten *Tetrahymena* species, one-to-one orthologs were aligned using MUSCLE [[31](#_ENREF_31)], and a maximum-likelihood tree was constructed using RAxML[[32](#_ENREF_32)] with the PROTGAMMALGF model and 1000 bootstraps.

Based on the species tree, the divergence time of *Tetrahymena* species was estimated using r8s [[33](#_ENREF_33)] with the approximate calibration time point taken as the divergence time for *Ichthyophthirius* and *Tetrahymena* (447 Mya) (another study in review).

**References**

1. Gorovsky MA, Yao MC, Keevert JB, Pleger GL (1975) Isolation of micro- and macronuclei of Tetrahymena pyriformis. Methods Cell Biol 9: 311-327.

2. Ma HW, Choi JK, Song WB (2003) An improved silver carbonate impregnation for marine ciliated protozoa. Acta Protozoologica 42: 161-164.

3. Xiong J, Lu XY, Zhou ZM, Chang Y, Yuan DX, et al. (2012) Transcriptome Analysis of the Model Protozoan, Tetrahymena thermophila, Using Deep RNA Sequencing. Plos One 7.

4. Xiong J, Lu YM, Feng JM, Yuan DX, Tian M, et al. (2013) Tetrahymena Functional Genomics Database (TetraFGD): an integrated resource for Tetrahymena functional genomics. Database-the Journal of Biological Databases and Curation.

5. Sweet MT, Allis CD (2006) Isolation and purification of tetrahymena nuclei. CSH Protoc 2006.

6. Xiong J, Wang GY, Cheng J, Tian M, Pan XM, et al. (2015) Genome of the facultative scuticociliatosis pathogen Pseudocohnilembus persalinus provides insight into its virulence through horizontal gene transfer. Scientific Reports 5.

7. Li RQ, Zhu HM, Ruan J, Qian WB, Fang XD, et al. (2010) De novo assembly of human genomes with massively parallel short read sequencing. Genome Research 20: 265-272.

8. Butler J, MacCallum I, Kleber M, Shlyakhter IA, Belmonte MK, et al. (2008) ALLPATHS: De novo assembly of whole-genome shotgun microreads. Genome Research 18: 810-820.

9. Trapnell C, Roberts A, Goff L, Pertea G, Kim D, et al. (2012) Differential gene and transcript expression analysis of RNA-seq experiments with TopHat and Cufflinks. Nature Protocols 7: 562-578.

10. Haas BJ, Delcher AL, Mount SM, Wortman JR, Smith RK, et al. (2003) Improving the Arabidopsis genome annotation using maximal transcript alignment assemblies. Nucleic Acids Research 31: 5654-5666.

11. Stanke M, Diekhans M, Baertsch R, Haussler D (2008) Using native and syntenically mapped cDNA alignments to improve de novo gene finding. Bioinformatics 24: 637-644.

12. Majoros WH, Pertea M, Salzberg SL (2004) TigrScan and GlimmerHMM: two open source ab initio eukaryotic gene-finders. Bioinformatics 20: 2878-2879.

13. Parra G, Blanco E, Guigo R (2000) GeneID in Drosophila. Genome Res 10: 511-515.

14. Birney E, Durbin R (2000) Using GeneWise in the Drosophila annotation experiment. Genome Research 10: 547-548.

15. Huang XQ, Adams MD, Zhou H, Kerlavage AR (1997) A tool for analyzing and annotating genomic sequences. Genomics 46: 37-45.

16. Keller O, Odronitz F, Stanke M, Kollmar M, Waack S (2008) Scipio: Using protein sequences to determine the precise exon/intron structures of genes and their orthologs in closely related species. Bmc Bioinformatics 9.

17. Haas BJ, Salzberg SL, Zhu W, Pertea M, Allen JE, et al. (2008) Automated eukaryotic gene structure annotation using EVidenceModeler and the program to assemble spliced alignments. Genome Biology 9.

18. Jones P, Binns D, Chang HY, Fraser M, Li WZ, et al. (2014) InterProScan 5: genome-scale protein function classification. Bioinformatics 30: 1236-1240.

19. Mistry J, Finn RD, Eddy SR, Bateman A, Punta M (2013) Challenges in homology search: HMMER3 and convergent evolution of coiled-coil regions. Nucleic Acids Research 41.

20. Fitch WM (1970) Distinguishing Homologous from Analogous Proteins. Systematic Zoology 19: 99-&.

21. Tatusov RL, Koonin EV, Lipman DJ (1997) A genomic perspective on protein families. Science 278: 631-637.

22. Chervitz SA, Aravind L, Sherlock G, Ball CA, Koonin EV, et al. (1998) Comparison of the complete protein sets of worm and yeast: Orthology and divergence. Science 282: 2022-2028.

23. Rubin GM, Yandell MD, Wortman JR, Miklos GLG, Nelson CR, et al. (2000) Comparative genomics of the eukaryotes. Science 287: 2204-2215.

24. Li L, Stoeckert CJ, Roos DS (2003) OrthoMCL: Identification of ortholog groups for eukaryotic genomes. Genome Research 13: 2178-2189.

25. Chen F, Mackey AJ, Vermunt JK, Roos DS (2007) Assessing Performance of Orthology Detection Strategies Applied to Eukaryotic Genomes. Plos One 2.

26. Chen F, Mackey AJ, Stoeckert CJ, Jr., Roos DS (2006) OrthoMCL-DB: querying a comprehensive multi-species collection of ortholog groups. Nucleic Acids Res 34: D363-368.

27. De Bie T, Cristianini N, Demuth JP, Hahn MW (2006) CAFE: a computational tool for the study of gene family evolution. Bioinformatics 22: 1269-1271.

28. Harris RS (2007) Improved pairwise alignment of genomic DNA: ProQuest.

29. Wang YP, Tang HB, DeBarry JD, Tan X, Li JP, et al. (2012) MCScanX: a toolkit for detection and evolutionary analysis of gene synteny and collinearity. Nucleic Acids Research 40.

30. Guy L, Roat Kultima J, Andersson SGE (2010) genoPlotR: comparative gene and genome visualization in R. Bioinformatics 26: 2334-2335.

31. Edgar RC (2004) MUSCLE: multiple sequence alignment with high accuracy and high throughput. Nucleic Acids Research 32: 1792-1797.

32. Stamatakis A (2006) RAxML-VI-HPC: Maximum likelihood-based phylogenetic analyses with thousands of taxa and mixed models. Bioinformatics 22: 2688-2690.

33. Sanderson MJ (2003) r8s: inferring absolute rates of molecular evolution and divergence times in the absence of a molecular clock. Bioinformatics 19: 301-302.
