# Supplementary material for: Hidden genomic evolution in a morphospecies—The landscape of rapidly evolving genes in Tetrahymena
Source: PLoS Biol. 2019 Jun 3;17(6):e3000294. doi: 10.1371/journal.pbio.3000294 (PMC6564038; doi:10.1371/journal.pbio.3000294)
Supplement: S3 Data — CRS, consensus repeat sequence. (DOCX) [file pbio.3000294.s050.docx]

**Supplementary file 2. CRS sequences identified in all *Tetrahymena* species.**

>T.thermophila_rnd-5_family-155#LRR_CRS1

CAATTTAATTATTACATTTATAAATGAAAATCATATTTAATTAAAAATTTTGACCTTTTATAAATATTATTTTTGAATACTTAATTTTAAATAAATAGCATTCATGAAAAAAAAAAAAAAAAAAAAAAAAAAAAAAAAAAAAAAAAAAAAAAAAAAAAAAAAAAAAAAAAAAAAAAAAAAAAAAAAAAAAAAAAAAAAAAAAAAAAAAAAAAAAAAAAAAAAAAAAAAAAAAAAAAAAAAAAAAAAAAAAAAAAAAAAAAAAAAAAACTATTCAAATTTCCTACGAAATTTAAATCAAAATAGATAATTTAATTAAAAACTTTTATTTTGTCTATAAATCAATAAAGAATCAATGTTACCAAATCTAAAAGTTGAGATTAGTTAGGAATTTGCAGTTACCAAGTGCTCTACCTATACTAGAAGCTCCATTATCCCCAATTTAATTTTTACTTTTATTAATGAAAATCATGATTAATTAAAAATTATAGACAGTTTATAAATATTATTTTTATTAACTAAAAAAAAAAAAAAAAAAAAAAAAAAAAAAAAAAAAAAAAAAAAAAAAAAAAAAAAAAAAAAAAAAAAAAAAAAAAAAAAAAAAAAAAAAAAAAAAAAAAAAAAAAAAAAAAAAAAAAAAAAAAAAAAAAAAAAAAAAAAAAAAAAAAAAAAAAAAAAAAAAAAAAAAATCTAAATTAATAAAAACGTCAAATAAAGTAAATTAATTCATTAACTATTAAATTTGTTTTGAAATTTAAATCAAAATATATAAAATAATTCAAAATTTTTAAATTATTAATAAATAAATATAAAATTAATGTTACCAAATGTAAAANTTGAGATTAGTAAGGAATTTGCAGTTACCAAGTGCTGTACCAATAGTAGAAGCTCCATCATCCCCAATTTAATTTTAGCTTTTATAAATGAAAATCANGTTTAATTAAAAATTTAAGAGTTTTATAAATTTAAATTTTTTTAATTNAGTTTAAATAAAAAGCTTTGACATAATTTTTTTAAAAAATTATAAATGAAATAAATTTACCNTATNCCAAATTCTAAATTTGTAAGTTGAGTGCANTTACTCAAACCTAATCCAATATTTTATGCTCCTTTATCACCGATTTTATTTCCCCTTTTCAAACAAAATAAATTAATTTATTAACTATTTAAATTTGTTTTGAAATTTAAATCAAAATANANAAAATAATTCAAAATTTTTAAGTTATCAATAAATAAATAAAAAATTAATNTTACCAAATNTAAAATTTGAGATTAGTAAGGAATTTGCAGTTACCAAGTGCTGTACCAATAGTAGAAGCTCCATCATCCCCAATTTAATTTTAGCTTTTATAAATGAAAATCANGTTTAATTAAAAATTNAAGACATTTTATAAATTAAGTTTATTTAATTAAGTTTGAATAAAAAATTTGACATAATTTTTTCAAAANTATATAAATGAAATAAATTTACNNTATNCCAAANTCTAAATTTGTAAGTTGAGTGCAGTTACTCAAACCTAATCCAATATTTTATGCTCCTTTATCACCGATTTTATTTTCCCTTTTCAAACAAAATAAATCAATTTATTAACTATTTAAATTTGTTTTAAATTTAATCTCAAAATATAAAATAATTAAATTTTTTAAATTATCAAAATTATAAAATTAANNTTACCNAATGCAAAATTTNAGATTAGTAAGNAACTTGCAGTTACCNAGTGCTGTNCCAATAGNATAAGCTCCNTCATCCCCAATTTAATTNTTACTTTTATAAATGAAAATAATATGCTTTAAATAAATAAAATTATCATCAAATTTAAATTTGATTAATTTAAAACTTTTTAAATAAAAANATAAAAATATTAAAATTTTTTTAAATTTTAAAAAATGAAAATTAAANTTACCATAGCNCAAATTTTAAATTCNTAAGTAGTTAACAGCTACCTAGTNCTATACAAATAGCTGATGCTCCTTCNTCTCCAATTTNATTGTTACTAATAAAACATAAAATAATATATATATACAACTTAAAAANTGTTTAAGGATAAATTGATTAAAAATTTATTAAACTGTACAATTAATTTAAATTTATCCTTAAAAAAAANTAAAATTAATTACGAGAGGTTAATATCTAGATGAGTCAACATTTTACAGACTACTTAACGCCGTACTNATAGAAAATGCACCTTCTTTTCCAAATAAATTTGCACTTTTTTAAGTTTTTATTTACAATTAAATATAAAATTATATTGAAAAATAATTTTTTAATTTATAAACAATAATCAATAAAATTTACACTGCATTTAAAGTTAAAGTCGTGAGTTTAGCACAACTACTAATACCTTCTGCAATACTCGATATCCCTCTGTCACCTATTTTATTTTTCCTTTCCAAAAAAAGATNTTTAATAAATAATTTATTTATGTTTTAAATAATACAATAAATATAACTGAATATGCTTAATATAATTGAAATAAAATTACCAAAGCTGCAGATTTAAGTTATTAAGTGTTTTGCATCTACGTAATACTTTGCAAATCCAAACTGCACCTCCATCTCCTAATTAATTTCCACTTTTATTATAAAATATTATTGAAAATAATTTAGTTTAGATAATGAAAANTTTTAATGAGTTTTGAAAGATGAACTTAATTATATTAAAAATAAATAAATTAAAAAAGNATACTCTAAATTTAGCTATAAACTGTTGAGATTAATTAGATTGCTCATAGTTTCTTCTAATAGAATATTTCCTTAAGCTTCAATATCATTTAATCTTTCAAATTAAAAATAAATCAATTAAATACCAATAGTTAGTTAGTTCTATTTTACCTAAGTTCTTGACAATCATGATTAATCTAAAAATATTTAGGAAAAACAAAATTTGGGCGTATATTTAATCATTGCTATTTAAATAAAATAAAATTATAACTATTTTAATTNATTTAAATTATTAAATTAACATATAAATTAATATTTTATTTAACATAATTTAAGATTCAAACTTTTAGNTTTTTATAAATGGTAGAAATAACTAGGGTAANAAGATAATGTTATAATTTATTAAACAATTTGTTGGNTATATTAAGATTTATTAAAATAAAAATATATTTATTTACCTCACATTATTACCAGTTTCAACAAAATCCTTTACGNAGGGATATTTAGCAAATAATTAATTTATCTAATCATCTGAAATATTTTCCTTACTAAATATTAGATTGATAGAAAAAAGTAATTATGTAATTTAAGATAATAAAGTTAAATTATTACTTAAGATTTAATACTAAAGATTCTAATTTTTAACATTATACTAAAGAATTGTTAAGTTAAGCAACTTATTCGTTACTAAGAGTACAATTTCTATTTATTTTATAAATAATAACAAAAGAATTTAATTTATGAATAAATTAATGAATGAATAAATTTTATTTATTCTTAATTTTGTAATAGAAAAGANAAAAAATTTAAAAATATTTATTTTTATTAAAAAAAATATTGCTTGTTTTGAATAAATTTATGATTAAATATGAATGAATAAATATTTATATATATATTTCTTACTTTAACTGTAAGTCAATATGAGTTAGAGCTTTCAAGTCAGATTTCATTAACTGTTTGTTTAAAAAAAAGATTTTATAATAATAAATAAAATTAATAATGATGGATACATCTTATAGAATATTTAAAGAGTTATTCTATTACTCCNTTTTTTTTTATTNATTTCCCATTTATATTTCTTTGATTATTAATTAATTAATTATGATCAGAAATTAATAATTTTGATTAATTAAAAAAATTATTGATTTTCTTTTTTAAATATTCTTATTTAATTTGTACCATTCTAAAAAATATCAGTAAATCAGTCTCCTTATTATTNCTCCTTTATAAAGATATATATCTTAAATACTNTAATTAAACTATCATAAAATTACAATTTTCTATATTTTAGAGGNTTAATTAATTTTTATTATTAAAATTTNTTNATATTTAAAAATATATGAATATATGGATATAAAAGGTAGATATAATGAAANTAAATTTCAAAATNTTATTAAAATAAATTTANTATGATATTACTGATATAAAAAATTAATTATGATANTTATATAGTTATTTTTTTGCAAATGTAGNATAGTANATAAAAAAGAAATATTTTATTCNATAAAGAATATAAATNATTTTNATTATTTGTTTTAATGATAATTAAATTATGATTTTCAATAATTGTTTTTAANAAAATCGAAATTAAACGATAAAAAAAAATTATTTTACAATTTTTATATTCAATTTTTTTTGTATATATACGTACTATTATATTAATAAATAATATTTAATTATTCATTTTAGTAAATTCAATCTATATTTAATTTNACTAATCTTTTTATTTTTTAAGCTAATCTTTTGATTTCTGTTTTTCCTTNTTCAGGCAATTTTCTTTTATAAATATAAATTTATTTTTTAAATAAATAAAAAATTTTACTTAAATTATCTATTTTAAACAATAAAAAGTAATTACTTTAATAACTTTATATTTTTACAGTTGATAATTTCTCTGGATTTTAGAAACTTCTCATTATTACTTATAAAATAAAGATATTTTAAATAACATCTTAAAAACTTTAACATNATTTTTTTCAAAAGTAATAAATGAAAATTTACTCTGATCTAAAAACTAAATTTGTAAGTTGAGTGCAGTTACTCAAACCTAATCCAATATTTTATGCTCCTTTATCACCGATTTTATTGCCCCTTTTCAAATAAAATAAATCAATTTATTAACTATTTAAATTTGTTTTGAAATTTAAATCAAAATATATAAAATAATTCAAGCTTTTTTAGTTATCAATAAATAAATAAANAATTAATTTTACCAAATGTAAAATTTGAGATTAGTTAGGAACTTGCAGTTACCAAGTGCTGTACCAATAGTAGAAGCTCCATCATCCCCAATTTAATTTTAGCTTTTATAAATGCAAATCACGTTTAATTAAAATTTAAGACATTTTATAAATTAAAGTTTTTTTTAATTCAGTTTGAATAAATAAAACTGACGTAATTTTTTTCAAAAGTAATAATAAATNAAATAAATTTACCNTATGCCAAATTCTAAATTTGTAAGTTGAGTGCAGTTACTCAAACCTAATCCAATATTTTATGCTCCTTCATCACCGATTTTATTTTCCCTTTTCAAACAAAATAAATTAATTTATTAACTATTCAAATTTNNTTCGAAATTTAAATCAAAATAGATAAAATAATTNAAAACTTTTAAATTGTCTATAAATCAATAAAGAATTAATGTTACCAAATNTAAAANTTGAGATTAGTTAGGAATTTGCAGTTACCAAGTGCTCTACCNATAGTAGAAGCTCCATCATCCCCAATTTAATTTTNGCTTTTATAAATGAAAATCATGTTTAATTAAAAATTAAAGACATTTTATAAATANTTTTTTGTAACTTGATTTAAATAAAAAGGNTTNACGTAATTTTTTCAAAAATTATAAATGAAATAAATTTACNNTATGNCAAATTCTAAATTAAATAAATAAAATAATATTAAAATTAATNTTAAAACGAAATGTAAAAGTTACGAGATTAGTTAGGAATTTACAGTTACCAAGTGCTNTACCAATAGCTAGAAGCTCCATCATCCCCAATTTAATTGTTACTTTTATAANTNAAAATNACGTTTAATTAAAAATTAAGACATCTTACAAATGTTATTTTTGTATACTTNGTTTTAAATAAACAA

>T.thermophila_rnd-3_family-28#LRR_CRS2

AAAAAAAAAAAAAAAAAAAAAAAAAAAAAAAAAAAAAAAAAAAAAAAAAAAAAAAAAAAAAAAAAAAAAAAAAAAAAAAAAAAAAAAAAAAAAAAAAAAAAAAAAAAAAAAAAAAAAAAAAAAAAAAAAAAAAAAAAAAAAAAAAACATTTTTTCCTTCTAATTAACTATTTTATGATATATTTTTTCTTCAATATTTAAAAAGTTGGAATTAAATTGGTGACGAAGGTGCATCAGGCTTAGGTTCTGGTTTAGCAAATTGCATTAATCTCTCAAATTTGACACTTNACCTTNGGTAAAAACAGTTTATTTGTTTTGGATTGTGATATTTTGAATTATAAAAACTAATTAAAAACAATAAAGCTCTTTTTTCTTTTTCTTTTTATTGTGCTTGCTTTTTTTTGTTCTTTCTTTGTTAATTTATTTAGCTCATCATTTTATTTTCAAATCTTTTGCTTTTATTATTNATTAATTAATCTTATTATTTCATATTTTTTCTTCATTTTTTCCTTCTAATTAACTATTTNATGATATATTTTTTCTTCAATATTTAAAAAGTGGNAATTAAATTGGTGACGAAGGTGCATCAGGCTTAGGTTCTGCTTTAGCAAATTGCATTAATCTCTCAAATTTGACACTTAACCTTGGGTAAAAACAGTTTATTTGTTTTGGATTGTGATATTTTNAATTATAAAAACTAATTAAAAACAATAAAGCTCTTTTTTCTTTTTCTTTTTATTGTGCTTGCTTTTTTTTGTTCTTTCTTTGTTAATTTAGCTCATCATTTTATTTTCAACTTTTGCTTTTATTATTTATTAATTAATCTTATTATTTCATATTTTTTCTTCATTTTTTCCTTCTAATTAACTATTTAATGATATATTTTTTCTTCAATATTTAAAAAGTTANAATTAAATTGGTGACGAAGGTGCATCAGGCTTAGGTTCTGCTTTAGCAAATTGCATTAATCTCTCAAATTTGACACTTGACCTTGGGTAAAAACAGTTTATTTGTTTTGGATTGTGATATTTTNAATTATAAAAACTAATTAAAAACAATAAAGCTCTTTGAATTTATTTGATAGCTTTTATTTATTTTATTTTTTTATCTTATCCACTAATATTATTTTATTTATTAATTTAAAGAATAATTTTTAATTTAAAAGTTTATTCTATTTTGATATTTATTTTAAAATTTACACAAATTTATTATAAAATTTTTAGCTCAATGAATGAATCATAAAAATTCAAAGTAATAAGCAAGTGTCTTAAATCAAAAAGATTAGTTGTCTTTAAAAATTTCTAATGATAAACAANTGGTGAAAAAAGGATGAATAAATATATAAAATGTTTGAATAATATTATATATTTTATTATATAATATTTTGAAAAAGTGTTTTTAACCTAAAATATAAATAGATAGATAGATAGATTTATTATTGATTTTATATTTATAAATATCATTCATATTTTTGTAAGTAAATAAATAAATTATTTATAAAAAATAAAAAAATTAAATTTACTTAAATAAATTAAATCCTCTTAAGCAAACCCTCTGGTTTCTCCAAAAACGATCAAATAATTTGAAACTTTTAGTGTTATATTACATCTTATTAAATTATATAAATGCCAAACTTTTATATGCCAGTTATATTTTAATCTTAAATATTATCCATCAATCATATTTCTATCCTTTTTTTATTTATCATGATTAATTAGATGCCATGATTTAAATAAAACCTCAGGGAGAAATCAAGTTTTACTTTTTATTTTAACTTTTGTGGTTATAATGTTTATTTTTTNTTTTTTTCTCAAATTTTNAATTTATCTTATGATCATTTTTTTTTCATTTTATATTTTTTAAAATCATAAAATAATTGTTTTTTAAATTATCATTNATTCATTCAGACAATACAAATTTGATTTTTTTATTTAATAATATTTAGAGTAAATTAATGAATTTAAAAGCCGTTAATGTTGAATTTGGCTTTTTATAAGCTTATTTCATATCAAGAATTTATGTAATTTTTTTTTAATGTGGTATTATAAATTTTAAAATGCTTATCTTATTTTTTATTATAAAATGTTCTCTAAATATTCTANAAAAATGCAAATAAATTTTTAATNAATTTAATTTAAATAAAAAATAATTTTTATAATTTTGATATATANGTATTTATGNATTATAATAATAGTTTCATTTATTATTTATATATCAGTTTTATAAGATTNATTACATTTAAAACATAAAATTTATATATAAAAAATAATTATTAATAATACTTAAAAATTTATTGATTAATATTTATTTGCAATAAANATATTTTTATATATTTATTTTAATTNTATTTTCTTTTCAATTNTTAATTTTAATTTTGAATTTNTGTTCAAATTATNAAATCTATAAAATAATAAATATTTTTAATGAAANTAAAATANAAAAATAATTAATAAATNAGTTAATAATGAATNATTTTGCTAAACATATTAAAAAGTGCTTATTTATTTATTTTGATCAATTAATTGGAATAATTGTTTCAATTCGTAAACAAATAAATTTAGATGCACAAAAAAAATTAAACAAAAAAATCAAAAAACAATTTATGAGCTGTGAATCAATCAATCAATTAATTCTCCAAACCCATTTGTTTTAAGTTTCAATTTCACAAATGTATTAATTAAAACAAACTAAATAAATTGATAGTTAGTTAAATTACCNNAGATCGAGATTCAAAGATGTTATATTTTNGCACTTCTCCAAGCTCATTCCAATGTTCTTAGCTCCATCTGCACCAAGGTNATTNCCNCTATGAAAATAATAAATAATGAATGTGTTGAAAATAATGAAAATAAAATAGAAAAGTAATTAATAAATGGGTTAATAATGAATGATTTTGCTAAACATATTAAAAAGCGCTTATTTATTTATTTTGATGCAATTAATTGGAATAATTGTTTCAATTCGTAAACAAATAAATTTAGATGCACAAAAAAAATTAAACAAAAAAATAAAAAANAATTTATGAGCTGTGAATCAATCAATCTAATTCTCCAAACCCATTTGTTTTAAGTTTAAATTTCACAAATGTATTAATTAAAACAAACTAAATAAATTGATAGTTAGTTAAATTACCCGAGATTGAGATTCAAAGATGTTATATTTTGGCACTTCTCCAAGCTCATTCCAATGTTCTTAGCTCCATCTGCACCAAGGTTATTATACTATGAAAATAATAAATAATGAATGTGTTGAAAATAATGAAAATAAAATAGAAAAGTAATTAATAAATGGGTTAATAATGAATGATTTTGCTAAACATATTAAAAAGTGCTTATTTATTTATTTTGATGCAATTAATTGGGATAATTGTTTCAATTCGTAAACAAATAAATTTAGATGCACAAAAAAATTAAACAAAAAAATCAAAAAACAATTTATGAGCTGTGAATCAATCAATTAATTCTCCAAACCCATTTGTTTTAAGTTTNAATTTCACAAATNTATTAATTAAAACAAACTAAATAAATTGAAAGTTAGTTAAATTACCNTAGATTNAGATTCAAAGATGTTATATTTTGGCACTTTTCTAAGCTCATTCCAATGTTCTTNGCTCCNTCTGCACCAAGNTNATTATNNCTATGAAAATAATAAATAATGAGTGTGTTGAAAATAATGAAAATAAAATAGAAAAGTAATTAAT

>T.thermophila_rnd-3_family-37#LRR_CRS3

ACAATTTTTATAAATTTTATAAAACTTTAAATGTTTAATTTTANGAAACATTTTTCCATCCTGAGTNTATAAANACNTGTATTTATATNCGAGNANATTAATTATTATTATGCTTTCTAAAAATTAGCCTTATTTAAATATTGAAGAAAAAATATATCATTAAATAGTTAATTAGAAGGAAAAAATGAAGAAAAAATATGAAATAATAAGATTAATTAATAAATAANAAANAAAGAATGAAAAAATAAGCTAAATCATAANAAAAAGAAAAGGAAAACAAATCAAATAAATTAAAAGAACTTTAATGTTTTAAATTAACTTTTATAATTAAAAATATTACAATNTAAAACAAATAAATAGCTTTTATTAAGTGTAAGTTTTAATAGCAAAAAATGAAGAAAATATATCAAATAATAAAAAATGAATAATAAATAAATAATTAAAAATTTAAATGAAAATTAAGTTAAAATAGCTAAAAAATTTTAGTTTTAACAAAAATCAATAAATAATGATGATAATTTNTTTTATAAAAATTATTTATTTAAATAAATTGAAAATTAAATATANAAAAAATATTAATAATAATAAAAATAATATTTTTTAATTAAATATACCTCAGATTAATACGGAGAACCTAATGAGATAAAAGTGAAGAGCTTTTAAACTTTTTTTATTAATTTATATAAATTTAAAGGATATTAATAAAAAAATTAATAATTACTTCTTCNAAATTATAATATTTTTTAATCTATTATATCATTTATTTTTTTTTTCAAAAATATTGAATAGCAATTTATGAAATTTGATTAATTAATTAATTAATTATAAATGGATAAAAAGAACAAATAAATATAANAATTAATTAATTATCAAATATTAATTTATATATTTGGTTTTATGAAGAGATATCACAGAAAATTAATTTAACATTCACTATTTAAACTTAATATTTGTATTTAAAACATATACTTAAAAATAGTATAGTAGGAATTAGAGACGGCCAGTTATTTATTTAGTCATATTTGAAAAAAACCTAGTAGTTAATGTTCTTAAATATNATAAAAATAACGTTTATAAGTAGATTTTTCTGTGTTTTAGCTCTGTAATTTTAGGAATATTTATAATATCTCTAATTATAGCAAGATGNNATACAAAAATTTCATGGTTTGCCTGCCACTTATTATGAAATATATTTTTTATTTGTATATTTATTTANTAAAAAATCTGATTTATNTATTTATTTTATAANAGAATTATAATTAATTAATAATTTAATCACTTCCAGATTAAAATNCTTNTGGTAGTTTACCGCAAAAATATTATAATTTTGGAAGTAAGTAATTTAATTAATTTTTGAAAAATTATTAAAATAAATATATAAATTTATNATTATAAATANTTTAAAAAATTATAAANTAATTTACTAAATATTTNTACGTAAAAATATCTTTATAAATATAAATTAATATAAAATTTTTAATTAAAANTTGTACTTATTTGTTNTTTATATAATTGATATATAATTTATTAAAAATGTAATTAANTTTAATTNCTATTATATTTTTAAGTACATGTTTTAAATANATATTAAAATTAAATAATTAATGCTAAATTATTCCTGTAATATTTTTTNATAAAAACCAAATTTNTTAATTATTAATATTTCAANAATTAATTATTATATTTATTTGATTCTTTTTATTCATTTAAATTAANTAGATTTTGAATTNTTAAGTAAATTTTTGAAAAAATAACTTTCTTATAAATGATATAATATTAAAATTTCGAAATTTTAAGAAGTAATTATTTATTTTTATCAATATCNTTTAGTTNATNTAAGAATAAAAAAAAGTTTTTAGCTATTCGACTTNTTTCTCATTGAATTCTAAATTATTTGTTATGTACATTTCATCAATTTATCTATTTAATATATATTAATAATTTTTATATATATAACTTTCAATTTATAGAAGTATAATTAAAAAATGCCTTTTNTATAGAAATATTTCATTATTATTAATTTTAATTTAAATAAAGNTTTTTTAGCTATTTCAANTTGATTTTCGTATTTCAGATCTTTAATTATTTAATTATTTTTTATTAATATATTTTTTCGCTATTCTAATTGGTAAAATTAATTTNAAACATTAAATTTCTTTTAANTNATTTNATTTATTTCATTTTTCTTTTTATCTTCATTGCTTTTTTCCNCCTATTGTTAACNNAANTGANNTNNCTATTTNATATTATATCTTTCTTCATTTTTTCCTTCTAATTNACNATTTAATGATATATTTTTTCTTCAATATTTAAAAAGGTGGCTAATNAAATTGGTGAAATGAAGGTGCATCAGGCTTAGGTTCTGCTTTAGCAAATTGCATTAATCTCTCAAATCTGACACTTGACCTTCGGTAAAAATCAGTTTATTTGTTTTGGATTGTGGTATTTTTAANTATAAAAACTAATTNAAAACAATAAAGCCTNTTTTTTCTTTTTCTTTTTATTNTGCTTGCATTGATTTNTTGTTCCTTCTTTGTTAAATTTAGCTNATCATTTTATTTTCAACTTTTGCTTTTATTANTTATNTCTTATTATTTCATNTTTTTTCTTCATTTTTTCCTTCTAATTNACTATTTANTGATATATTTTNTCTTCAATATTTAAAAAGNNGCAATNAAATTGGTGNNGAGGGTGCATCAGGCTTAGGTTCTGCTTTAGCAAATTGCATTAATCTNTCANATTTGACACTTGAGCTTNGGTAAAAACAGTTTATTTGTTTTGGATTGTGATATTTTTAANTATAAAAACTAATTAAAAACAATAAAGCTCNTTTTTNTCTTTCTTTTTATTGTGCTTGCTTTTTTTTGTTCCTTCTTTGTTAATTTAGCTCATCATTTTATTTTNAACTTTTGCCTTTATTATTTTATTTCAATTTTNTCTTATTATTAATAAACTNTTATTATTTCGTATTTTTCTTCAATATTTAAAAAGTAACAATTAAATTGGTGATGAAGGTGCATCAGGCTTAGGTTTTGCTTTAGCAAATTGCATTAATCTCTCAAATTTAACACTTNACCTTTNGTAAAAATAGTTTATTTGTTTTGGATTGTGATATTTTTTAAANCNATACAACTAACTANTTTAATTTAGCATTTATNAANTTCCATTTGATTTNTTTTTACTTTTNANCGTGCTTGGTCTTTTTTGTTCTTTCTTTGTTAATTAAACTCATTATTTTATTTTCAANTTTTGCTTTTATTATTAATTAATTTATTATTTCATATTTTTTCTTCATTTTTTCCTTCTANNTAACTATTTAATATAATTTTTTCTTCAATATTTAAAAAGTGACAATNAAATTGGCGATGAAGGTGCATCAGGCTTAGGTTCTGCTTTAGCAAATTGCATTAATCTNTCAAATTTGACACTTGNACTTGAGTAAAAACAGTTTATTTGTTTTGGATTGCGATATTTTTAATTATAAAAACGAATTTAAAACANTAAAGCTCTTTTTTCTTTTTCTTTTTATTGTGCTTGCTTTTTTTTTGTTCTTTCTTTNTTAATTTAGCTCATCATTTCGCTTTTATTATTAATTAAATCTTATTATTTCATATTTTTTCTTCATTTTTTCCTTCTAATTAACTATTTAATGATATATTTTTTCTTCAATATTTAAAAAGTNANAATNAAATTGGTGATGAAGGTGCATCAGGCTTAGGTTCTGCTTTAGCAAATTGCATTAATCTCTCAAATTTGACACTTNACCTTNNGTAAAAACAGTTTATTTGTTTTGGATTGTGATATTTTTAATTATAAAAACTAATTNAAAACAATAAAGCTCTTTTTTCTTTTTCTTTTTATTGTGCTTGCTTTTTTTTGTTCTTTCTTTGTTAATTTAGCTCATCATTTTATTTTCAACTTTTGCTTTTATTATTAATTAATCTTATTATTTCATATTTTTTCTTCATTTTTTCCTTCTAATTAACTATTTAATGATATATTTTTTCTTCAATATTTAAAAAGTNNCAATTAAATTGGTGATGAAGGTGCATCAGGCTTAGGTTCTGCTTTAGCAAATTGCATTAATCTCTCAAATTTGACACTTGGCCTTAGGTAAAAACAGTTTATTTGTTTTGGATTGTGATATTTTTAATTATAAAAACTAATTNAAAACAATAAAGCTCTTTTTTCTTTTTCTTTTTTTATTGTGCTTGCTTTTTTTTGTTCTTTCTTTGTTAATTTAGCTCATCATTTTATTTTCAACTTTTGCTTTTATTATTAATTAATCTTATTATTTCATATTTTTTCTTCATTTTTTCCTTCTAATTAACTATTTAATGATATATTTTTTCTTCAATATTTAAAAAGTTACAATTAAATTGGTGCGATGAAGGTGCATCAGGCTTAGGTTCTGCTTTAGCAAATTGCATTAATCTCTCAAATTTGACACTTNACCTTNNGTAAAAACAGTTTATTTGTTTTGGATTGTGATATTTTTAATTATAAAAACTAATTTAAAACAATAAAGCTCTTTTTTCTTTTTCTTTTTATTGTGCTTGCTTTTTTTGTTCTTTCTTTGTTAATTTAGCTCATTATTTTATTTTCAAATTTTGCTTTTATTATTTAATTAATCTTATTATTTCATATTTTTTCTTCATTTTTTCTTTCTAATTAACTAAATAATTATATAAAAANANGAATCAAATTGGTGATGAAGGTGCNTCAGGCTTAGGTTCTNCTTTAGCAAATTGCATNAATCTCTCTAATTTANCTCTTAATCTTGGGTAAAATATTTAAACAATAAGCTCTTTTTTACACTTTNTTTTTAGTGTTCTTGCAATATNTTTTTTGTTAATTTAACTCATCGTTTTATTTTNAACTTTTACTTTTATNATTAATTAATCNTNTTATTTCATATTATTTTTTAATTTTTCCTTCTAANNAACATAATTAATNATATATTTNCTCTTGAANACTTAAAAAGTCNCAATNAAATTGGTGNTGAATTGGTGCATCAGGCTTAGGTTCTGCTTTAGCAANTTGCACGAATCTCNCAAATTTGACACTTNACCTTGGGTAAAAAGAGTTTATTNGTTTCGGATTGTGATATTTTNAATTATAAAAACTAATTNAAAACAATAAAGNTCTTTTAATTTATTTGATATATTTTCCTTTTTATTGTGCTTGCATTTTTTNTNTTCNTTTTTTGCTAATTTAACTCATCATTTTATTTTCANCTTTTGNTTTTATTATTAATNAATTAATCTTATTANTTNATATNTNTTCTNCATTTTTTCCTTCTAATTAACTATTTAATATAAATTTTTNTTCAAAATTTAAAAAGCGAAAATTAAATTGGTGATNAAGGTTCATCAGNTTTAGGTTCTGCTTTAGCAAATTGCATTAATCTCTCAAATTTGACACTAGANCTAGNGTAAAAACAGTTTANTTGTTTTNGATTGNGATATTTTTAACTATAAAAGCTAACTTAAAACAANAAAGNNCTTTNAATTTATTTGATTTATTTTTCTTTATCATTTTCATTCCGNTAATGTTATTTTATTTANTAATTTAAGAANTCAAATTTTTATTATANAANTTTATTTTATTTAATACAATTTATTCAAANTAATTATAGCAAGTGTTTAACCAAATAAATGAATCANAATAATTCAAAATAAAAAGCAAGTGTCTTAAATCAAAAAGATTAGTATTCATTTGAAATATGAATAATATTCTNATGATATAAACAAGTGGCGAAAAAANGATGGATANATANTAAATTTATGTTTGAATANTNGAATATNATATGTTTTATTATATATATATAGTACCCTAGGGTGCTTGCAGCCTTTTTCAAGNCTNCATACACGAATNNTAACAAGATTTATATTANAATAATCGTAAAAATACATTTTATATTTTATCATTTTATATAATTNCCCTCCTTTTTTAAAATNTTTAAAAATATCCGNTTGTGCCCTCATTTTNNAGATTNGATTTNANTCATAAAATACATATAAATAACATAATACAAAATAACACTTGCTTTTAAAAACAATGGCTATGTGCTGTTTANTAGCAATAGCCATTTTTTTAAAATAGCAAAGTGCTNTAT

>T.thermophila_rnd-4_family-197#LRR_CRS4

AAAAAAAAAAAAAAAAAAAAAAAAAAAAAAAAAAAAAAAAAAAAAAAAAAAAAAAAAAAAAAAAAAAAAAAAAAAAAAAAAAAAAAAAAAAAAAAAAAAAAAAAAAAAAAAAAAAAAAAAAAAAAAAAAAAAAAAAAAAAAAAAAAAAAAAAAAAAAAAAAAAAAAAAAAAAAAAAAAAAAAAAAAAAAAAAAAAAAAAAAAAAAAAAAAAAAAAAAAAAAAAAAAAAAAAAAAAAAAAAAAAAAAAAAAAAAAAAAAAAAAAAAAAAAAAAAAAAAAAAAAAAAAAAAAAAAAAAAAAAAAAAAAAAAAAAAAAAAAAAAAAAAAAAAAAAAAAAAAAAAAAAAAAAAAAAAAAAAAAAAAAAAAAAAAAAAAAAAAAAAAAAAAAAAAAAAAAAAAAAAAAAAAAAAAAAAAAAAAAAAAAAAAAAAAAAAAAAAAAAAAAAAAAAAAAAAAAAAAAAAAAAAAAAAAAAAAAAAAAAAAAAAAAAAAAAAAAAAAAAAAAAAAAAAAAAAAAAAAAAAAAAAAAAAAAAAAAAAAAAAAAAAAAAAAAAAAAAAAAAAAAAAAAAAAAAAAAAAAAAAAAAAAAAAAAAAAAAAAAAAAAAAAAAAAAAAAAAAAAAAAAAAAAAAAAAAAAAAAAAAAAAAAAAAAAAAAAAAAAAAAAAAAAAAAAAAAAAAAAAAAAAAAAAAAAAAAAAAAAAAAAAAAAAAAAAAAAAAAAAAAAAAAAAAAAAAAAAAAAAAAAAAAAAAAAAAAAAAAAAAAAAAAAAAAAAAAAAAAAAAAAAAAAAAAAAAAAAAAAAAAAAAAAAAAAAAAAAAAAAAAAAAAAAAAAAAAAAAAAAAAAAAAAAAAAAAAAAAAAAAAAAAAAAAAAAAAAAAAAAAAAAAAAAAAAAAAAAAAAAAAAAAAAAAAAAAAAAAAAAAAAAAAAAAAAAAAAAAAAAAAAAAAAAAAAAAAAAAAAAAAAAAAAAAAAAAAAAAAAAAAAAAAAAAAAAAAAAAAAAAAAAAAAAAAAAAAAAAAAAAAAAAAAAAAAAAAAAAAAAAAAAAAAAAAAAAAAAAAAAAAAAAAAAAAAAAAAAAAAAAAAAAAAAAAAAAAAAAAAAAAAAAAAAAAAAAAAAAAAAAAAAAAAAAAAAAAAAAAAAAAAAAAAAAAAAAAAAAAAAAAAAAAAAAAAAAAAAAAAAAAAAAAAAAAAAAAAAAAAAAAAAAAAAAAAAAAAAAAAAAAAAAAAAAAAAAAAAAAAAAAAAAAAAAAAAAAAAAAAAAAAAAAAAAAAAAAAAAAAAAAAAAAAAAAAAAAAAAAAAAAAAAAAAAAAAAAAAAAAAAAAAAAAAAAAAAAAAAAAAAAAAAAAAAAAAAAAAAAAAAAAAAAAAAAAAAAAAAAAAAAAAAAAAAAAAAAAAAAAAAAAAAAAAAAAAAAAAAAAAAAAAAAAAAAAAAAAAAAAAAAAAAAAAAAAAAAAAAAAAAAAAAAAAAAAAAAAAAAAAAAAAAAAAAAAAAAAAAAAAAAAAAAAAAAAAAAAAAAAAAAAAAAAAAAAAAAAAAAAAAAAAAAAAAAAAAAAAAAAAAAAAAAAAAAAAAAAAAAAAAAAAAAAAAAAAAAAAAAAAAAAAAAAAAAAAAAAAAAAAAAAAAAAAAAAAAAAAAAAAAAAAAAAAAAAAAAAAAAAAAAAAAAAAAAAAAAAAAAAAAAAAAAAAAAAAAAAAAAAAAAAAAAAAAAAAAAAAAAAAAAAAAAAAAAAAAAAAAAGTTTNTAATATTTTTAATTTAAAAATTAATTTAAAACATTAAAGCTCTTTTAATTNATTTGATTTTTATTTTTTTTTTTGCTTAATTTCTCTTTAATTTATTNAGCTATCTTTGATCAACTTGANATTNATTTTTATAATTATTAAAATTTATTATTTAACATATTTTTNCCATTTTTTCCTTCTATTAACTANTCAATTACATATTATTTCTTCAATATTTAAAAAGTNTCAATAAAATTGGTGATGAAGGTGCATCGGNNTTAGGTTCTGCTTTAGCAAATTGCATTAATCTCTCAAATTTGACACTTAGACTTTAGTAAAAACAGTTTATTTTTCTTTGATTATGATATTTTAATNANAAAAACTANTTAAAAACAATAAAGCTCTTTTTTCTTTTTCTTTTTATTGTGCTTGGTTTTTTTTGTNNTTNNTTTGTTAATTTATTTAGCTCATCNTTTNATTTTGAATTTTTACTTTTATTATTAATTAATATCATTATTTCATATTTTTTCTTCATNTTTTTCTTCTCATTAACTATTTAATTATATATTTTTTNTTNAATATTTAAAAAGTAAGAATTAAATTGGTTCAATAGGTGCATCAGGCTTAGGTTCTGCTTTAGCAAATTGCANTAATCTCTCAAATTTGANACTTNAACTTTAGTAAAAATAGTTTATTTGTTTTTAGTTGTGATTTTTTTANTTATAAAAACAAATTTAAAAACAATAAAGTTAATTTTTCTNTTTCAATTTTTATTGTACTNGCTTTTTATTTGTTTATTTATTTATTAATTTAACTCATAATTTNATTTTNAATTTTTGCTTTTATTATAAATTAATTAATTTTATTATTTNATATTTTTTNTCATATTTTCTTTCTCGTTAACTCGTTAGTAATATATTTTTTGTTNAATATTTAAAAAGTGGNAATNAAATTGGTGCATGAAGGTGCATCAGGCTTAGGTTCTGNTTTAGCAAANTGCACTAATCTCTCAAATTTGANACTTGATCTTCCGTAAAAAGAGTTTATTTTTTTTGGATTGTGATATTTTTATCNTTAAAACTGATTTAAAACAATAAATTTATTTGATAGCTTTTATTTGATTTATTTTTTTATCTTTATTATNCCACTAATGCTATTTTATTTATTAATTTAAGAAATATTTTTTAATTTAAAANTTTATTCTATTTTGATGTTTATTTAAAATTAACAAATTTATTATTTNANATTTTTTTTCTATATTTTCCTTCTCATTAACTAATTAATTATATTTTTTCTTTAATATTTAAAAAGCTACAATTAAATTGGTGATGATGGTGCATCAGGNNNNNNNNNNNNNNNNNNNNNNNNNNNNNNNNNNNNNNNNNNNNNNNNNNNNNNNNNNNNNNNNNNNNNNNNNNNNNNNNNNNNNNNNNNNNNNNNNAAATTTACTATTAATTATCATTTTTACATTTCATATTTTTTCTCAATTTTTCTTCTNAATAATTATTAATNATNTATTTTTTATTNAATATAAAAGCNNCAATTAAATCGGNGATGAGGGTGTATCAGGTTTAGNTTCTNCTTTAGCAAANTGCACTAANCTCTCAAATTTGACACTCNANCTTNAGTAAATAATAGNTTATTTTTATATTGTTTTTTTTATTTAAAGTAAAATAAATTTAATTATACAATAAATTTCATTTTTATTTTTTTTTTTATTNTTTGTATTTTTTTNNTCCCATTTTTTAATTTAANTNATAANTTTATTTTAAAATTTTGCTCTTATCGTAAATTAATCAATTTTATTNTTTNATAATTTTTCTTCATANTTTCCTTCTCAATAAGTCATTAATTATATATTTTNTCTTNAATATTTAANAAGTANAAACGAAATTGGCGATGAAGGTGCATCGNNCTTAGGTTCTGCTTTAGCAAANTGCATTAATCTCTCAAATTTGANACTAGACCTTNCGTAAAAATAGTTTATTATTTTTTNATTGCGATATTTTTATTTTTGGCAATTTAAAACAATAAAGTTAATATTTCTTTTNNTTTTTATTGTGCCTGGTTTNTTTTCTTCTTCCNTTTTTAATTTAGCTCATAATTTTATTTTGAGCTTTTGCTTTTATTATTAATTAATCAATTTTGTCATTTCANANNCTTTTTCTANNTTTTCCTTCTCATTAANTAATNAATTAAAATATTTTTNCTTNAATATTTAAAAAGNTACAATTAAATTGGTGACGAAGGNGCATCAGGCTTAGGTTCTGCTTTAGCAAACTGCATTAATCTCTCAAATTTGAGACTCGATCTTNGGTAAAAACAGNTTATTTNTTTTGGATTGTGATATTTTTAATTAAAAAACTAATTAAAAATAATAAAGTTCTTTTTTCTTTTTCTTTTTATTGTGCTTGCTTTTTTTTCTTCTTTTTAATTTATCGNTCATTATTTTATTTTGANTTTTTGCTTTTATTATTATTAACGATTTTTTATTATTTCATATTTTTTCTTNATTTCTTAACTCATTATTATATATTTTTTATTNAATATATAAAAAGTGGNAATNAAATTGGTGATGAAGGTGCATCGAGCTTAGNTTCTGCTTTAGCAAANTGCATTAATCTCTCAAATTTGANACTTNATCTTTAGTAAAAACAGTTTATTTTTTTTTANATTNTGATTTTTTTATTTATAAATAAATAAACTTAAAAAATAAAGCTCTTTTTTCTATTTCTTTTTATCGTGCTTGGTT

>T.thermophila_rnd-4_family-46#LRR_CRS5

AAAAAAAAAAAAAAAAAAAAAAAAAAAAAAAAAAAAAAAAAAAAAAAAAAAAAAAAAAAAAAAAAAAAAAAAAAAAAAAAAAAAAAAAAAAAAAAAAAAAAAAAAAAAAAAAAAAAAAAAAAAAAAAAAAAAAAAAAAAAAAAAAAAAAAAAAAAAAAAAAAAAAAAAAAAAAAAAAAAAAAAAAAAAAAAAAAAAAAAAAAAAAAAAAAAAAAAAAAAAAAAAAAAAAAAAAAAAAAAAAAAAAAAAAAAAAAAAAAAAAAAAAAAAAAAAAAAAAAAAAAAAAAAAAAAAAAAAAAAAAAAAAAAAAAAAAAAAAAAAAAAAAAAAAAAAAAAAAAAAAAAAAAAAAAAAAAAAAAAAAAAAAAAAAAAAAAAAAAAAAAAAAAAAAAAAAAAAAAAAAAAAAAAAAAAAAAAAAAAAAAAAAAAAAAAAAAAAAAAAAAAAAAAAAAAAAAAAAAAAAAAAAAAAAAAAAAAAAAAAAAAAAAAAAAAAAAAAAAAAAAAAAAAAAAAAAAAAAAAAAAAAAAAAAAAAAAAAAAAAAAAAAAAAAAAAAAAAAAAAAAAAAAAAAAAAAAAAAAAAAAAAAAAAAAAAAAAAAAAAAAAAAAAAAAAAAAAAAAAAAAAAAAAAAAAAAAAAAAAAAAAAAAAAAAAAAAAAAAAAAAAAAAAAAAAAAAAAAAAAAAAAAAAAAAAAAAAAAAAAAAAAAAAAAAAAAAAAAAAAAAAAAAAAAAAAAAAAAAAAAAAAAAAAAAAAAAAAAAAAAAAAAAAAAAAAAAAAAAAAAAAAAAAAAAAAAAAAAAAAAAAAAAAAAAAAAAAAAAAAAAAAAAAAAAAAAAAAAAAAAAAAAAAAAAAAAAAAAAAAAAAAAAAAAAAAAAAAAAAAAAAAAAAAAAAAAAAAAAAAAAAAAAAAAAAAAAAAAAAAAAAAAAAAAAAAAAAAAAAATAATAAAAAAAGGCATCGAAAGAGGTTTTGGACCTGAANTAGCNAAANAAAATAAACTTTTAAATTGAAAAATATTTTTTGTTAAATNAATNAATAAAATAGCGTTANTAGTTGNATGAAAAAGANAAAAAAATNAATCAAATAAAAGCTATCAAACAAATTCAAAGAGCTTTNTTGTTTNNNATTCGTTTTTATAGTAAAAAATATCACAACCCAAAACAAATAAANTGTTTTTACCGAAGGTGGANTGTCANATTTNAGAGNTTNTCGCANTTNGGTAACCTNAAGCAGAGCCTAAGCCTGATCACTCATTTNTTAATCAATTTNATTNTAACTTTTTATAAATTTAAGAGAAAATANATTGTTAAATAGAAGTAAANATTGAAAGTGAAAAATATGAANTAAATTAAGANNGATNGATAGATAAATCCAAAAGTTTAAAATAGAATGANGAATTAACACNAGCAAATGCAATAAAGAAGAAAAAAAATGCAAGTCACAATAAAAAGCTAAAAAAGAAANTTAAAGGGCTATATTGTTTTAAATAATTTTTTATANTTAAAAATATAAAATCGAAACAAATAAACTGNTTTTACCTNAGATAAAGTGTCAAATTTGAGAGNTTAGNGCAATTTGCTAAAGCAGAACCTAAGTTTTATGCNCCTTAGTCACCAATTTTATTNTCGCTTTTGTATTAAAAAATATATTATTAATTATTTAATTAGAAAAATAATAATTAATTAATAATAAAATAAAAGTAAAATAAAATAATNAGTTAAATTAACAATTAAGATAGAAAAAAAACAAACACAAGTTAAAAAGAAAGAAAAAAGATAAATAGAAAAAANGGCTTTATTGTTTTTCAAATTAATTTTTTATAANTAAAAATATCAATCTAAAATAAATAAATTATTTTTACCTGAGATCAAGCGCCAAATTTGANAGATTNACGCATTTTGCTAAAGCAAAACCTAAGCCTGATGCACCNNNATCNCCAATTTCGTTNGANCTTTTTAAATATTGAANAANAAATATNTAATTAAATAGTTAATTAGAAGGAAAAAATGAAGAAAAAATATGAAATAATAAGATNAATTAATAATAAAAGTAAAANTTGAAAATAANATGATGAGTTAAATTAAGAAAGATTCAAGAAGAANAAACTNAAGTAAAATGNANAANAAATAGAAAAGAAATAGAAAAAAGGTTCTATTTATAAATTAATTTATATATATATATATAGTACCCTAGGGTACTGTCTTTTTCAAGGCTACATACAAATAGAATACATAANATAGTTTTATTTTTCTTTTTCTGGGATTTTTGATTTTAAAAAATTTTTATTTTTANTTAGTTTTTATANTTAAAAATATCACAATCCAAAACAAATAAACTGTTTTTACCTAAGGTTAAGTGTCAAATTTGAGAGATTAATGCAATTTGCTAAAGCAGAGCCTAAGCCTGATGCACCTTCGCGCCAATTTAATTNTAACTTTTTAAATATTGAAGAAAAAATATGTCATTAAATAGTTAATTAGAAGGAAAAAATGAAGAAAAAATATGAAATAATAAGATTAATTAATAATAAAAGCAAAAAATTAAAATAAAATGATGAGCTAAATATAACAAAAAAGAAAAAAGAAAGCAAGCACAATAAAAAGAAAAANAAAAAAGAGCTTTATTGTTTTAAATTAGTTTTTATAATTAAAAATATCACAATCCAAAACAAATAAACTGTTTTTACCTNAGATCAAGTGTCAAATTTGAGAGATTANTGCAATTTGCTAAACCAGAACCTAAGCCTGATGCACCTTNGTCACCAATTTAATTTTCACTTTTTAAATATTGAAGAAAAAATATATTATTAAATAGTTAATTAGAAGGAAAAAATGAAGAAAAAATATGAAATAATAAGATTAAATTAATAATAAAAGTAAAAATTGAAAATAAAANGATGAGCTAAATTAACAAAGAAAGAAGAANAAAAAAAAGCAAGCACAATAAAAAGAAAAAGAAAAAANAGCTNTATTGTTTTAAATTAGTTTTTATAATTAAAAATATCACAATCCAAAACAAATAAACTATTTTTACCNAAGGTCAAGTGTCAAATTTGAGAGATTAGTGCAATTTGCTAAAGCAGAACCTAAGCCTGATGCACCTTTATCACCAATTTNATTGTCACTTTNTAAATATTGAATAAAAAATATATAATTAAATAGTTAATTAGAAGGAAAAAATGAAGAAAAAATATGAAATAATAAAATTAATAATAAAANTAAAAATTGAAAATAAAATGATGAGCTAAATTAANAAATNAACNAAGAAAGAANAAANNCAAGCANAATAAAAAGAAANAGAAAAAAGAGCTTTATTGTTTNTAATTAGTTTTTATAATTTAAAAATATCACAATCCAAAACAAATAAACTGTTTTTACCTAAGGTAAAGTGTCAAATTTGANAGATTAATGCAATTTGCTAAAGCAGAANCTAAGCCTNATGNACCTTNATCACCAATTTAATTNTCACTTTTTAAATATTGTAGAAAAAATATNTTATTAAATAGGTAAAATAGGTANAAATGAAAAATAATTATAAATTAAATATAAATTTTTAAATTTTTATTAAAATTACNAATAAGTGAAATATTTATAAGCATANTAAAAATAATTTTAAATATTCTACGNATTTATTTTGCGATCCTATCGATTTCGCAAATTTATTTGATTTTTAAATATTGAAGAAAAAATATATCGTTNAATAGTTAATTCGAAGGAAAAAATGAAGAAAAAATATAAAATAATAAGATTAATTAATAAATTAACAAATAA

>T.thermophila_rnd-5_family-10#LRR_CRS6

AGAAAAAATATGAAAATAATAAAATNAATTAAAAATAAAAATNAAATNAAAATAAAAATAAATAAATTAAATAAAAATAATTAAAAAATTAAATTAANTTTTATAAGTAAAAATATCANAATCCAAAACAAATAAACTGTTTTTACCNAAGATNAAGTGTCAAATTTGAGAGATTAGTGCAATTTGCTAAAGCAGAACCTAAGCCTGATGCACCAATTGCACCAATTTAATTTTANCTTTTTAAATATTGAAGAAAAAATATANAATTAAATNGTTATTAGAAAAAATNAAGTAAAAATATGAAATAATAAGATTAAAAAAAATAAAAGCAAAATTAAAATAAAAAAATAAGTTAAACTAATAAAAAAACTAAAATATTTAGATNATTTTTTAAGTAAAAATATCACAATCCAAAACAAATAAACTATTTTTACNAAAGATNAAGTGTCAAATTTGAGAGATTAGTGCAATTTGCTAAAGCAGAACCTAAGCCTGATGCACCAATTGCGCCAATTTAATTGNATCTTTTTAAATATTGAAGAAAAATTAAATTGTTATTGAAGGAAAAAATATGAAATAATAAGTTAGTTAAAATAGAGCAAAAGTCGAAAATAAAAAATGAGTTAAATTAATAAAAATNAATAAAATAAAAAGCAAACAAATNAAAAAAAAGAGCTTTATTGTTTTAAATTNATTTTTNTAAGTAAAAATATCACAATCCAAAACAAATAAACTATTTTTACNAAAGATNAAGTGTCAAATTTGAGAGATTAGTGCANTTTGCTAAAGCAGAACCTAAGCCTGATGCACCNANTGCACCAATTTNATTNTAACTTTTTAAATATTGAAGAAAAAAAAATAATTAATTATTTATAGAAGGAAAAATGAAGAAAAAATATGAAATNATAAGATTAATTAATAATAAAAGCAAACATTGAAAATAAAANAATGAGTAAATAATAAAAAAAGAATATAAAAAATTTAAATATAATAAACAAATAAAAAGAACAATTTTTATTTTAAATTAATTTTTATAATAAAAATATCANAATNCAAAACAAATAAACTATTTTTACNNAAGATTAAGTGTCAAATTTGAGAGATTAGTGCAATTTGCTAAAGCCGAACCTAAGCCTGATGCACCTTTATCGCCAATTTTAATCTTTCTAAATATTGAAGAAANAATATGTTTTGTTTCATANNNNNNNNNNNNNNNNNNNNNNNNNNNNNNNNNNNNNNNNNNNNNNNNNNNNNNNNTTGCATCTAATGCATCNATTGTTTCAAGTTTTTAGTTTAATAAAAATAATAATTCAAAATATCCAATAAAATAAATAAATGTATTTACCATAANATTAAATATTTAATTTAAGATGTATTCTGAGTGCATTTNTATGTGCGAGTATTTCTATTCCATTCATCGCCAATTTTATCGCTACTTTTTAAATATTGAAAAAATAATANATAATTAATTAGTTAATTAGAAGGAAAAAATGAAGAAAAAATATGAAATAATAGATTAATTAATTATAATAAAAGCAAAAGTTGAAAATAAAAAAATGAGTTAAATTAACAAAGAAAGAATAAAAAAAGCTATCAAGCACAATAAAAAGGAAAAGAAAANAAAGCTTTATTGTTTTAAATTAGTTTTTATAATTAATAAATATCACAATCCAAAACAAATAAACNGTTTTTACTAAAGNTTGAGTGTCAAATTTGAGAGATTAATGCANTTTGCTAAAGCAGAACCTAAGTCTGATGCACCTTTATCACCAATTTNATTATCACTTTTTANATATTAAGAAAAAATATATAATTAATTAGTTAATTATAAAGAAAAAATGAAGAAAAAANATGAAATAATAAGATNAATTAATAAATAATTAATNAAATTAACAAAGAACAAAAAAAAAGAAATCACAATAAAAAGAAAAAGGTAAACAAATAAATTAAAAGAGCTTTAATGTTTTAAATTAACTTTTATAATTAAAAATATTAAATCCAAAACAAATAAATNTTTTTATTAAGTGTAAAATTAATAGCAAAAAATGAAGAAAAAATATCAAATAATAAAAATGAATAATAAATTAATTAAAAATTGAAATGAAAATTAAGTTAAAATAGAGCTAAATTTTTGATTAATAAAAATTAATGAATTGAGTAATTGATGATAATTTTTATATAAAAATTATTTTTTAAATAAATTGAAAGTTAAATATATAAAAAATATTAATAATATAAATATATTNTAAATTAAATATACTTGAGATCAATATGTAGAACCTAATGAGATTAAAGTGAAGANTTTAAAAACTTTTTTTATTAATAAAAATAAATTTAAAGGATATTAATAAAAAAATAAATAATTACTTCTTCTAATTTTTAATATTTTTTAATCTATTATATCATTTATTTTTTTTNAAAAATATTGAATAGCAATTTATGAAATTTAATTAATTATATTTAAAATTATAAATGNTAAAAAAATATAATAATNAAAAATAATATAAATAATTTAATTTATTTATTTATAAGNAAANTTAATTTAAATTTATTATTTAAACTTAATATTTATATTTANAATAAATTTTAAAAATATATATAATAAAAATTAATAATAAAACTATNTATCTATTCTATCTAATTTTTAGGTTAATTTAAAAAATTTATCAAAATATTATATAATAAAACATNTAATATTTTCGATTATTCAAATATTTAATATTTATTCGCTTTTTTCGCCATTTNTTTATCATCAGAAATATATTTTAAAGACAACTAATCTTTTTGATTTAAGACACTTGCTTTTCTACGAATTTTTTATTNATTNATTCATTAACTCTTTTATTATAAATTTGTGTAAATNTTNAATAAANATTAAATAGAATAAANTTNTAAATTAAAAATTATATTTTAAATTAATAAAAAACATTAGTNGAATAAAANTAAAAGAAAAATAAATCAAATAAAAAGCTAATAAACAAAATTAAAAGANTTAGCTATATTGTTTTAAATTNATTTATTATTATTAAAAATATCACAATCCAAAATAAATAAACTGTTTTTACTTAAGANAAAGTGTCAAATTTGAGAGATTAGTGCAATTTGCTAAAGCAGAACCTAAGCNTGATGCACCTTATCGACTAATTTAATTGNTACTTTTTAAATATTGAAGAAAAAATAAATAGTTAATTAATTAATTATTTAGAAGGAAAAAATGAAGAAAAAATATGAAATAATAAGAAATTAATTAATAATAAAAGCAAAAGTTAAAAATTAAAAATGAGTTAAATTAACAATAGAAAAAAAATAAAGCAAATAAAAAAGAAAGCTAAATAAAAATAAAAAGTTAAAAAATAAAAGAGACTTTATTGTTTTAAATTCGTTTTTATAATTAAAAATATCACAATCCAAAACAAATAAACTGTTTTTACCTAAGNTCAAGTGTCAAATTTGAGAGATTAATGCAATTTTGCTAAAGCAGAACCTAAGCCTGATGCACCATCGTCACCAATTTAATTNTCNCTTTTTAAATATTGAAGAAAAATATATTCGTAATTAGTTANTTAAAAGGAAAAAATGAAGAAAAATATGAAATAATAAAAATAATAAGCAAAAGTTAAAAATAAAAAAATGAGTTAAATAAATTAACAAAGAAATAGAAGAAAAAAAAGAAACAAGTCACAATAAAAAGCTAATAAAGAAATAAAGAGCTTTATTGTTTTAAATTTGTTTTTATAATTNAAAATATCACAATCCAAAACAAATAAACTGTTTTTACTNAAGNTCAAGTGCCAAATTTGAGAGATTAATGCANTTTGCTAAACCAGAACCTAAGCCTGATGCACCTTNATCACCAATTTAATTGCNCCTTTTTAAATATTGAAGAAAAAAAATATATNATTTAATTAGTTATTTAGAAGGAAAAAATGAAGAAAAAATACGAAATATAAGAGTTAATTAATTNATAATAAACAAATTAAAATTAAAACATGAGTTAAATTATCAAATAGACAANGAAAGAANAAAAAAAAAGNAANCNNAATAAAAATAAAAAGTTAATAAANGAATTGAAAAATCTAGCTTTATTGTTTTGAATTATTGGGGTATTTTTAATGTTCGAAAATACCNCAGATTACCAAAACAANTNGACANTTTTTACCTTAGTTNAAGTGTNAAATTTTTCAAACAATTTAGTGCAGTTTCCTAAACCATGACCAGAATCTGATGCACCTTAATCACCAATTATTTTATTCCACCTTTTTAAATATTGAAGAAAATATATTTAGTTAATTANNNNNNNNNNNNNNNNNNNNNNNNNNNNNNNNNNNNNNNNNNNNNNNNNNNNNNNNNNNNNNNNNNNNNNNNNNNNNNNNNNNNNNNNNNNNNNNNNNNNGNNNNNNNNNNNNNNNNNNNNNNNNNNNNNNTTTTCTTGTTTTAAATTCGTTTTTAATTATTAAAAAATATTAAATAAAAATAACTATTTTTACNNAAGATCGAGTGTTAAATTTGAGAGATTAGTGCAATTTTCTAAANCAGAACNTAAATCTGATGCACCTTTTGNACCAATTTNATTNNCACTTTTTAAATATTNAATAAAAAATATAGATTAATTAGCTATTTAGTAGAAAAATAAATAAAGAATACGAATTNATATAAAAANNAAAATAAATAAAAAATAATTAAATTAAAAANTAAATAAAAATTAAATGAGCAGAATTTATTAAATAAAATTAATAAAANTAAAAATTTANTNAAAANTTTAAAATATTAATAAACAAATAAATAGTTTTTACGNAAGATCAAGNTTCAAATTTGAGAGATTAGNGCAATTTGCTAAAGCAGAACCTAAATTTGATACACCTTANTCGCCAATTTCGTTCTCCTTTTTAAATATAGAAAAAAAAAAATATNATTAATTAGCTTTAAAAGGGAAAAAATGAATAAAAATATGAAATAATAAGAAATAAATTAATTTATAATATTAAAGCAAAAATTGAAAACAAAAATATGAGTTAAATTAACAATAAGGAAGATAAAAAAAACAAAAATAAAAAATAAAATGACGAATAAATAAATCAANTAAAGAAAGAACNAANAAAAGCAAGCACAATNAAANNGGNAAGAAAAGAGAGCTTTATCGTTTTAAATTAATTTTTATANTTAAAAATATCACAATCAAAAACAAATAAACTGTTTTTACTCAAGCTCAAGTNTNAAATTTGAGAGATTAACGCANTTTACTAAACCAGAACCTAAGTCTGATGCNCCTTTATCACCAATTTAATTGGNACTTTTTAAATATTGAAGAAAAAATATAATTAATAGTTAATTAGTAGAAAAAAATGAANAAATAATATGAAATAATAAAAAATAATTAATTTATAANTAAAANAAAAATTGAAAATTAAANAATGAGTTAAATTAATCAAANAAAAAGCTCATAAATATAATTAAAAGAGNTAGTTAATTANTTAAAATTATTTTCGGGTAAATTTTTAGTNNGAAATNTAATTANTAAAATAATTTAAATATTTTTTATTAAATAAAATGATATTTATAAGAATATATGGTANGTANATATTATTTCAAATTTTACATTTTGTGAATTTAAAGCTTTCNAAAAACTCAAAAATAAAGCTGTTTTAAGCTAATTTAGCTTTATTATCANGTTATTAAGATATATTACATTTCGCCTTTTATAAATTAAAAAGGCATTTCACTCAGTTAATTAGNAAGAAAAAATGAANAAAAAAATGAAATAATAAGATTAATTAATAATTTAGTNAGATTTCTTCATTAAAAATTAAATATTTGATTAAAATTTTTTTATTTTTGTTTGATCAGGTTCGAAGCCTATTTTGATACCTTTTTTTATTATTCTGCTAAATATTTATTTCTTAATGAATGAAATAATATTAGATAATAATAANAAGAATATTTTTTATTAATTTTTAAATAAAAATAATATTAAAATAAATAAAAATTNATTTTTACTNAAGATTAAGTTCCAAANTTGAGAGATTAGTGCACTTTGCTAAAGCNAAACCTAANNCTNATGCNCCTTTATCGCCAATTTAATTATTNCTTTTNATATATTTAAAGTAATATAAATTAGTTAATTAAAATAGAAAAAACGAAGAAATAAATAATTAAAATAAAAAGAATCTAAATTAATTAANAATAAAAGCAAAAGTTGACAATGAAAAAATGAGTTAAATTAGCAAATAAAGTAAGGAAAAAAAAACACAAACAGAATTAAAAAAATAAATCAAATAAAAATCTCGTAAATGAATTATAAAGAGCTTGTTATTTATATTATTTTAAATTCATTTTTATATTCAAAGATATCTACAATCGAAAAAAAATAAATANTGTTTTACTAAAGATAGAGTTTTAAATTTNAGAGATTAGNACANTTTNCTAAACCAGAACCTAAACCTGATGCACCTNCATTACCTATTTCGTTTNCGCTTTTTAAANATTNGAGAAATTATAAAGANTTAATTAGTTATNTTAGAAGGAAAAAAAGTAATTTTAAATAAATATNAAANAATAAGAAATTCATTAATAATATAAACAAAAGCTGATAATTAAAAAATGAGTTAAATTAATAAAGAAAGAAAGGAAAAAAAAAACACAAGAAGAATAATATAAAAAAATAAATTAAATAAAAAACTAATAAATAAGGTAAAATAGTTGAATATTATGTTTTAAATTTATTTTTATATCAAAAATATCACAATCCAAAACAAATAAACTATTTTTACCTTAGACAGAGTGTCAAATTTGATAGATTAGGGCTTTTTTTTTTTTTTTTTTTTTTTTTTTTTTTTTTTTTTTTTTTTTTTTTTTTTTTTTTTTTTTTTTTTTTTTTTTTTTTTTTTTTTTTTTTTTTTTTTTTTTTTTTTTAAAGTATCCCAATCCAAAACAAATAATCAGTTTTTACAAAAGATGAAGAGTCAAATTTGAGAGAT

>T.thermophila_rnd-2_family-6#LRR_CRS7

AAAAAGTNANAATNAAATTGGTGCANAGGGTGCATCAGGCTTAGGTTCNGCTTTAGCAAANTGCATNAATCTCTCAAATTTGACACTTNATCTTGAGTAAAAACAGTTTATTTATTTTGGATTGTGATATTTTTAATTATAAAAATTAATTAAAAACAATAAAGCTCTTTATTATTTTTTTCTTTTTCTTTTTATTGTGCTTGCTTTTTTATCTTTTTTCTTTCTTTGTTAATTTAGCTCATCATTTTATTTTCAACTTTTGCTTTTATTATTAATTAATCTTATTATTTCATATTTTTTCTTCATTTTTTCCTTCTAATTAACTATTTAATGATATATTTTTTCTTCAATATTTAAAAAGTNANAATNAAATTGGTGATGAAGGTGCATCAGGCTTAGGTTCTGGTTTAGCAAAGTGCATTAATCTCTCAAATTTGACACTTTACCTTNGGTAAAAACAGTTTATTTGTTTTGGATTGTGATATTTTTAATTATAAAAACTAATTAAAAACAATAAAGCTCTTTTTTCTTTTTCTTTTTATTGTGCTTGCTTTTTTTTGTTCTTTCTTTCTTTGTTAATTTAGCTCATCATTTTATTTTCAACTTTTGCTTTTATTATTAATTAATCTTATTATTTCATATTTTTTCTTCATTTTTTCCTTCTAATTAACTATTTAATGATATATTTTTTCTTCAATATTTAAAAAGTNACAATAAAATTGGTGATGAAGGTGCATCAGGCTTAGGTTCTGCTTTAGCAAAGTGCATTAATCTCTCAAATTTGACACTTNACTTGAGTAAAAACAGTTTATTTGTTTTGGATTGTGATATTTTTAATTATAAAAACTAATTAAAAACAATAAAGCTCTTTTTTCTTTTTCTTTTTATTGTGCTTGCTTTTTTTTGTTTTCTTTCTTTCTTTGTTAATTTAGCTCATCATTTTATTTTCAACTTTTGCTTTTATTGTTAATTAATCTTATTATTTCATATTTTTTCTTCATTTTTTCCTTCTAATTAACTATTTAATGATATATTTTTTCTTCAATATTTAAAAAGTNACAATTAAATTGGTGATGAAGGTGCATCAGGCTTAGGTTCTGCTTTAGCAAANTGCATTAATCTCTCAAATTTGACACTTNATCTTGGGTAAAAACAGTTTATTTGTTTTGGATTGTGATATTTTTAACTATAAAAATNAATNTAAAACAATAAAGCTCTTTGAATTTATTTGTTNCTTTTATTTGATTTATTTTTTTATCTTTTTTATTNTACTAATATTATTTTATTTATTT

>T.thermophila_rnd-5_family-523#LRR_CRS8

AATTTGAGAGATTAATGCGCTTTGCTAAAGCAGAACCTAAGACTGATGCACCTTCATCGCCAATTTTATTTGACCTTTTTAAATATTGAAGAAAAAATATATNATTAANTAGNTNNTTAGAAGGAAAAAATGAAGNAANAANATGAATAAATTATAGGATNAATNAACAANAAAAGCAAANNTTGAAAATAAAANAATGAGCTAAATTAAGAAAGAAANAAAGAACAAGGANAAACGCAAGCACAATAAAAAAGAAAAAGAAAAATAAATAAATAAAAAACTAGTAAAAAAATTAANATTTAATTTTNTTTTTTATAAGTAAAAATATAACAATCCAAAACAAATAAACTATTTTTACTNAAGGTNGAGCGNTAAATTTGAGAGATTAACGCANTTTGATAAAGCAGAACCTAAGTCTGATGCACCTATATTCCAATTTTATTTTTTCTTTTATAAATATTNATTTAGAAGAAAAAATAAAAAAATAATAAGAAATAATTAATAAATAAATNAATAATAAAAGCAAAAGTTGAAAATAAAATGATGAGCTAAATTAACAAAGAAANAAAGAACAAATAAAANCGCAAAAAAAAGAAACAAAATAAAAAAAATAAAAAGAGCTTTATTGTTTTAATTNGTTTTTATAATTAAAAATATNACAATCCAAAACAAATAAACTGTTTTTACTAAAGGTTAAGTNTCAAATTTGAGAGATTANAGCACTTTGCTAAAGCAGAACCTAAGCCTGATGNACCTTCATCACCAATTTAATTNTAACTTTTTAAATATTGAAGAAAAAATATATCATTAAATAGTTAATTAGAAGGAAAAAATGAAGAAAAAATATGAAATAAAGATTAATAATAATAATAAAAGCAAAATTGAAAATAAAATGATGAGNTAAATTAACAAANAAAGAAAGAACAAAAAAAGCAAGCACAATAAAAAGAAAAATAAATAAANAAATAATTATTGTTTTAAATTAATTTTTATAANTAAAAATATCGCAAAAAAAAAAAATAAANTATTTTTACNCAAGGTAAAGTGTCAAATTTGAGAGATTAGTGCANTTTGCTAAAGCAGAANCTAAATCTGATGCACCTTNATCGCCAATTTAATTGTCGCTTTTAAATATTGAAGAAAAAATATATNATTAAATAGTTAATTAGAAGGAAAAAATGAAGAAAAAATATGAAATAANAAGATTAATTAACAATAAAAGCAAAAGTTGAAAATAAAATGATGAGCTAAATTAACAAAGAAAGAAAGAANAAATAAAAAAGCAAGCAAATAAAAAGAAAAANAAAAAAGAGCTTTATTGTTTTAAATTAGTTTTTATAATTAAAAATATCACAATCCAAAACAAATAAACTGTTTTTACNAAAGNTANAGTGTCAAATTTGAGAGATTAATGCAATTTGCTAAAGCAGAACCTAAGCCTGATGCNCCTTNACACGAATTTAATTGTNACTTTTTAAATATTGAAGAAAAAATATCATTAATTAGTTAATTAGTTGTAANAAATGAAGAAATAAATATGAAATAATAATTAATTAAAAATAAATAAATTGAATAATAAAATAATGGTTAAAATAAAANAACAAANNAAANCAAGCATAATAAAAATAGAAAAAGAAAAAAGATATATAAATACAATAAAAAAAAGAAAATTAAATTAAATAAATAAATAAAAAGCTTGTAAAAAAGTTAAAAGATTTATATTGTTTTAAATTCATTTTATAACTAAAAATAAAAAAAAAAAAAAAAAAAAAAAAAAAAAAAAAAAAAAAAAAAAAAAAAAAAAAAAAAAAAAAAAAAAAAAAAAAAAAAAAAAAAAAAAAAAAAAAAAAAAAAAAAAAAAAAAAAAAAAAAAAAAAAAAAAAAAAAAAAAAAAAAAAAAAAAAAAAAAAAAAAAAAAAAAAAAAAAAAAAAAAAAAAAAAAAAAAAAAAAAAAAAAAAAAAAAAAAAAAAAAAAAAAAGAAGGAAAAAATGAAGAAAAAATATGAAATAAAAAGATTAATTAACAACAAAAGCAAAAGTTGAAAATAAAATGATGAGCTAAATTAACAAAGAAAGAAAGAAAGAACAAAAAAAAGCAAGCACAATAAAAGNAAAAANAAAAGAGAGAATTATAGTTATTTTTACGTTTTAAAAAGATCANAATGNAAAATAAATAAGCTTTTACGCAAAATNAAGTTTTAAATTTGAGAGACTAGTGCATTTTGCTAAAGCAGAGCCTAAGCCTGATGCNCCTTNATCGCCAATTTNATTTCTACTTTTAATATTGAAGAAAAAATATAAAAATAATTAGTTAGTATTTAAAGAAAAAATGAAGAAAAAATATNAAATAATAAGAGATAATTAANAATAAAAAGAAAATCAAAATTTGAAAATAAAANAATGAGCTAAATTANGAAAGATATAAGAAGAATTTTAGNACAAATACAATAAAAAAGAAAAAGTAAAAATAAAGTTTAAAATAAAAAGCTTTTAAATATTCTTAACTAATTTATGAATTAAAAATTTAAATTAATTTTTATAACTAAAGAATNNTAATTCAAAAGAAATAAATTTTACCNAAGATCGAGTGTCAAATTTGAAAGATTAATGCATTTTGATAAAGCAGAACCTAAGCTTGACGCACCTTATGNACCAATTTAATTANAACTTTTTAAATTTGAAAAAAAAAAATATTATGAAATAATAATTAGAAGTAAATTAATAAATAAAGAAAATAATATGANAATAAAATAATNAGTTAAANTAAAAANTTAAAATNAAAATGAGTAAANTTAAATAAAAAAANAAANAAAAATAAATTAAATTCAATAAAAATTAATATACAAATATAAGATTTATATTGTTTTAAATTATTTTTATAATTAAAAATAATNAATCAAAACAAATAAACTATTTTTACNTAAGATCGAGTGTCAAATTTGAGAGNTTAGTGCATTTTGCTAAAGCAGAACCTAAGTCTGATGCACCTTNTNCGCCAATTTAATTCTANCTTTTTAAATATTNAAGAAAAAATATTATAATTAAATTGTTATTTAGAAGGAAAAAGTGAAGAAAAAATATGAAATAAGAGATGAATTAATAAATAAATATGAAAAGCAAATNTTGAAAATAAAANAATGAGTTAAATTAAGTAAAGATAGAACAAGAANAAAGCACAGCTACAATAAAAAAGAAAAGAAAAATAAATAAATAAAAAGCTAATAAACTAATTAAGATTTAAATTCGTTTTTATAACTAAAAATATCGCAATTCCAAAAAAATAAACTGTTTTTACAAAAGNTCGAAGGGTCAAATTTGAAAGATTAATGCAATTTGATAAAGCAGAACCTAAGCCTGATGCACCTTNAACCAATTTAATTCCAACTTTTTAAAATTGAAGAAGAAAANATATGANTTAATAAGAAATGAATAAATAAATATGTAANAANTACTAATTCGGAAAATAAAATAATGAGTTAAATTAACGAAAGATAAGGAAGAACAAGACAAATACAATAAAAAGAAAAAGAAAANTAAATTAAATTAAGNAAAAATCTAATATTTACAAATTAAGATNTACATCANNTTAATATGGAGAACCTAGTGAGATTAAAGTGAAGATTTTAAAAACTTTTAATATTGAGAGCCGCCTAGTNAGATTAAATTGAAGAATTTAAAGNNTTTTGATATTAGTAAANATAAATTTAAAGTATTTTGATAAAAAAAATAANTAATTACTTCTTCTAATTCNCNGTAGTATTTANTCTATTATATCATTTATTTTTTCGNTNAAAAATATAATGAATAGCAATTNACGGAAATTTAATTAATTATAAGTNGATAAAAAGAACAAATAAATTATATGAATTTATAAATTTGCTTTTCATAAAAGAAATCACAAAAAANTTAGGATAACTAACTTCATTATTAAATCTTAATATTTCTTTTTAAAATGCGTACTTAAAAATATAGTNGGAACCAGAGATTTGGATATAAAGGATAGTNAGAATGCGAACTCATTAGAATAAAATAAATTTTTGACNTATTTAAGTGATTTCGAATTTATTTTTTGAATTTTTGGAAATNATTTTTAAATCTATTAAATAANTCAAATTTTCTTATAAATAAATAATTAAAAATCATAAAAATAACGTAAAAAATANAAAATTTAATAATNGNATTTNTTATNTAATAAAGAAATATTTTTATAATAATTGTAATCCATATTTATCTATNATAAANNTTAAAAGGAGATTAATATATATTTAGAATTATAAATATATATATAGTAACTTAGGGACTGCCTTTTTCAAGGCTTCACACTAAAATTTTAAATTTAANGTAATTATAAAAANCAGTTTTTTATGTTAAATCAGAGTTAGCAAATATATTAAATNTGTGGTCTTAAGTAGTAAATTAATTTANAATATATGGNTATAATTAAAAGCGANNAAAAATA

>T.malaccensis_rnd-3_family-8#LRR_CRS1

AAATTAAGTAATATATCTATGAAATTTTTAAAAATAAAAATAATTTTATGAGGCTTTTCTGAAAATATACACTGTTTATTGTTACTTGTTAATTACAAATTAGATTTTATTTAAATTAAATTAAATAATGCTTATTTTACTAATTTCTATCATACTGATTAAATATATTAATTTCTGNTCGTAATTAATTAAGTAAAGATATTACTTTCTAATCATTAATTAATTAAAAAAATTTTTTGATTCTAATTAACAATNACTAAATTAATCATAAAGGTAAAGAAGAAAGAAAAAATTAAAAAAAAATGGAGTAATAGATTAACTCTTTTAATAGTCTGAAGGATGTAACAGNTATTTTTTATTTAATTTATTGTTATTATATCTTTTTTTTTCAATAAAAGTTTAAAAAATCAAACTNNAAAGCTCTGACTAGTATTGACTTACAGTTGAAGTAATATTATCTTTTAATTAAATTTTTTTTTTCAATNNTTGAATTTTATACTAATAAAATTTNATTCTATTAATATTAAATATATTTTAAAATAAATAGAAATTTTGATTTCGATAGTGAATAGTTAGATGCGCTTAATACTTCTCTAAAGAAATGCTGCAAATTAGAATNTTTANCTTTGAATCTTTAGTAATTTTATAATCTAAAATATTATTATTTATTTATGTTTGTCATTTTAAAAAATAGATAAGAATGCATTTAAGATGACTAGCTAAATCGATTCTTTATTGATTATTAATGCTTAATAGATTTAATCCCAAATTTCAAAAATATGAGGTAAATNATAATAGTTTTTTTATAAAATACTTTAAATAGACNGTCGCCAGCCTAATCATTTTTAATATTCTTACTTCATAACNCTAATTTCTTTTANACTTAATAAAATATTAATTATTTTAATTTTACTTTTTAAAGTATCTTAAATATACTGATATCAATATCTATAATTCANTAAATATGANTTAAACACAGCAAAACTAAATTTTTCATCATATTATTTTAATAAANAGCAATGANCAAAGATATACCCATATGTAGTTTTTACTAAAGAACTATGGGTAATAAAGAATTACAAATAGAACCTAAGTATAGTTTATCCTTTGATATTAATTTTACTTATTGCTTAATTTAGAAAAAGTGAACAAATAAAAGTTTTAGGAGATTTCTTGTAAGATGATACTATTAGCAATTTAGTCAATCTTAAGAATTTAAAGCTAAATTTAAGGTACATTTTTATTTAAGTTCATATTTCAAANCTTATTTACATTTTTAGGAAATAATTAATATATACCTTCGTTTTTTATAATTTTAAAGATATAAATTCAAATAATAAAGAATATTTAGAGCTTTAAATATCTTAAAAAATTTTTAAGNATNAGAAAAAATTTTTAAATAAATAGAGGTATATTTTAACTAAGTTCATTTAAAAGAGCTTTTTGTAAAATAGCGCATTAANATANTTTACTTTATTAATATTAATTTTAACTTTTTTTATCGTTAAAAGTGGAAATTCAATNAACGATAAAGNTGCATTTACAATTTTTACAGTAATAGGCAGGTTTAAAATGCTCACTAACCTGAATTTACAGCTTTGGTAATTTAAGTTGCANATAGTTAAATATTTCGTAAACAAAATTAATTTGAGTAGGAATAATNAAATCAGTGATGTAGGGATATCAAATATAGGAGTTGGAATTAGTTGCTGTCTTAATCTTACAGGTTTAACTCTAAACATAGCGTAGATTCTATTTTTATTAAATTTAAAACACTTTATATTTAAATTATTTCTTTATTCACTCTTAAAATCTTAATAAAAAAAGATTTAAAAAATTTTAATTATAGAAAAAATTAGTTTGGTAGTGAAGGAGCATTTTATATTGGTAGANTATTGAGTGCTTGCAAATAGCTAATTTATTTAGATCTTAATATTTCGTAGATAATTTTGATAATATTTTTTATTTATTTAAAAANCTTTAGATTTATAATTTCATATATTTACTCATTTTTTCAATAATAAAGCCAAAATTAAATCGGAGATGGTGGAGCATCAGCTATTAGTACAGGATTAAGTAGCTGCTAATTACTTACTTATTTAAATCTTTAACTCTGGTAATTTTAATTTCGTTTTTTAATATATTTAAATTCAATTTAATTAAAAATTAATTTATTNATTTAATTTGTCAAATATAAAAGGGAAAATAAATTAACTGATAAAGGAGTATAAGATATTGTATCAGGATTAAGAAATTGCCTTCAACTAAAGAATTTAGAATTTAGTATTATGTAAATTAAATTTTTCTTTTTTAAATACTGTTTATTAAAAGTTTTTATTTTTTTTTTAGCAATTTATTTAAAATATTTTCANATAAAAGTAAGAATAACATTGGAANTGAAGGAGCATTTTCTNTTGGTGAAGCANTAGATAGATGTAAATNGCTAAATAATCTAAAAATTGATTTAAGGTATCTTTATTTTTTAAATAATTTAAAGCTTTCTTTACATTTTATTATTTATTTAATTAACTAAAAAACAATTTTTAAAATTTAATTTATTTTTAAGCATAAATTAAATGGTGATAAAGNTGCATTATAAATTTTATAAGATTTGAGTAATTGCAATCAANTTAANGATTTANAATTGTTTGAAAAGGGGNAACTAAATCGGTGATGCAGGNGCATCATAAATTGGATAAGGCTTAAGTAACTGCACTCATCTTACAAATTTAAAATTTNACATGAGGTAAATTTTATTTGTATTTTAACAATAATTTTTATTTCTAAATTATTTATTATTAAAATTGATTATATTAAAATNTTTTTATTTATATTTATTTAACATGAAATTTAATAATAAAAGCAAGAATTAAATTGGGAACGAAGGAGCATCTGCTATTGGTAGAGCACTAAGCAATTATAAGCTGCTAACTAATCTANAATTCTATCTTTAGTATATTTAATTTTATTTTTTTAATAAATAAAATTAAATAATTTTAATAATTTATAATTTTAATAAATNAATTAATTAACTTTAATTGTTTGAAAAGGGAAAACTAAATCGGTGATGCAGGAGCNTCATAAATTGGATAAGGTTTAAGTAGCTGCACTCATCTTACAAATTTAGAATTTNNCATGAGGTAAATTTTATTTGCTATTTTTACAANAATTTTTCTAATATTTTTAAATTATTTATATCAAAAAGTTGTTATTTATATTTATTTAACATGAAATTTAATAATAAAAGTAANAATTAAATTGGGAACGAAGGAGCATNTGCTATTGGTAGAGCACTAAGCAGTTATAAGCTNCTAACTAATCTANAATTCTATCTTTAGTAAATTTAATTTTATTTTTTTAATAAATAAATTTAAAATGCTTAAATAATTTATTNTATTTAATTTTAAGTTTTATTTAAATAATTTTAATAAATAAATTAATTAACTTTAATTATTTGAAAAGCGAAAATTAAATCGGTGATGCAGGAGCATCATAAATTGGATAAGGTTTAAGTAACTGCACTCATCTTACAAATTTAGAATTTNNCNTGAGGTAAATTTTATTTGCTATTTTTACAATAATTTTTCTAATATTTTTAATATTATTTATATTTAAAGTTGTNATTTATATTTATTTAACATGAAATTTAATAATAAAAGCGAAAATTAAATTGGGAACGAAGGAGCATCTGCTATTGGTAGAGCATTAAGTAATTGTAAACTGCTAACTAATCTACGATTTTATCTTTAGTAANTTTAATTTTATTTTTTTAATAAATAAATTTAAACTCTTAAATAATTTACNTTATTCAATTTTAAATTTATTTTNAAACAATTTTAATAAATAAATTAATTAACTTAAATTATTTAAAAGCGAAAATNAAATCGGTGATNCAGGAGCTTCGTAAATTAGNGAAAATTTAANNAGCTGCATAAACTTANAAATTTANAACTTTNCNTAGAGTAAATTTTATTTACTATTTTTAC

>T.malaccensis_rnd-4_family-427#LRR_CRS2

ATTTTATTGTTTTNCGAATTTGAGTCTTGGTTTATTAAGTATGATGTTCATTTAAATCTNTTTAATACTCTNTCTCTCANTCTTTCNCTCTTATTTATTTACTTATTTCTTTNTATTTATTTATTATTTAAATATTTTATTAGTNATAATTCGATTGGTGTCGAAGGAGCTAAGAGCATTGCTAATGCCATAGAGAAGTATCAAAATGTTACTNAATTNAATCTTAATTTAAGGTAATTTTTTAATTAATTTANTTTTATTTGTTTTCTTATTGNAAATTATTAATTAANTNTTTAATTTTTTTAATTGATTATTTATTTTAAAATTAACTCGTTACTTATTTTCTTTTTATTTCAATTTTCTTTACTTTCTCTTATTTATTAATTTATTTTATTTTTATTTATTTATTATTTAAATATTTTATTAGTTGTAATTCNATTGGTGNTGAAGGAGCTAAGAGCATTGCCAATGCNATAGAAAANTATCAAAATNTTACTGAATTGAATCTTNATTTTGGGTAATTTTCTTAAATTTAATTTTTATTTGTTTTTTTGCAAATTTTTTAATTGTTATTTTTATTTGTTTGTNTGTTTGTTTATTGATTAATTTTTTATCGATTTTCATTTACTTTCTTACTCTCTTATTTATTGAAATTTTTTACTTTNTTATATTTATTTATTATTTAAATATTTTATTAGNNATAATTCTNTTGGCGAAAATGGAGCTAAGAGCATTGCCAATGCAATANAAAATTATCAAAATCTCACTATAATTGANTCTTAATTTAAGGTAATTTTCTTAAATTTNATATTTNTTTGTTTTTTTTTGCAAATTTTATTNATTTTTTGTTTATTTGTTTGATTTGATTAGTTTATAATAAACTTTCACTTATTCTTTTACTCTTTCTCTCTCTTTCTGTCTTATTTATTGAAATTTTGTTTAAAATTGATTGCTAATATTTATATGTTATTTAATATTTTATTAGTGATAATTCTATTGGAGAAAATGGAGCTATGAGCATAGCCAATGGAATAAAAAANTNTCAAAATNGTTCANTCATACTGAANCTTNAATTCTTTTNAGGTAATTTTNTTAAAATTATTTAATTATTTATTTACTCTTATTTATTAAATTTTATTTGACAAATAAAATTANTTNTTCGTTAACTGANTGATNGGTTTATTTTAACATTTAATATTTTCTTCCCTCTCTTTCTCTCTTATTTATTTACTTATATTCTTTTTATTTATTTATTATTTAAATATTTTATTAGTAACAATAATATTGGCGATGATGGAGCNAAGAATATTGCTAATGCCTTAGAGAAATGTTAAAATATTACGAATTTGAGTCTTGATTTANNGTAATTTATTTAAATCTAATTTAATATTTTCTTTGATCAATTTCTCTATTATTTACTTACTTATTTTTANTTCTTTNTATTTATCTTTTATTTATTCAATATTTCGTTAAAAAACATTGNTAATATTTATATATAATAAAATAAGAAAAAAAAAAAAAAAAAAAAAAAAAAAAAAAAAAAAAAAAAAAAAAAAAAAAAAAAATTAAAAATAGCTAAATAAAAAATGTAAAACATATATATAAAAAAAAAAAAAAAAATCTTGTTTAATATTTATTTATTATNTATTATTTTATTAGTTCTAATTCTATTGGCGAAAATGGAGCTAAGAGTATTGCCAGTGCAATANAAAATTATCAAAATCTCACTAAATTNAAACTTTGTTTAAGGTAATTTTCTTAAATTTAATTTTTTTGTTTTANTTTGATTAATCGTTTTTGTTAATTTTCGCTTTATTTTTATCTCTCTCATTTCTACTCTTTTATTTCCCATTTATTTATNGAAATATTGTTTATTATATAATTATTAATTTTTNTTAAANATTTTATTAGTNATAATTNTATTGGAGAAAATGGAGCTTATAGCATTGCNANTGCGATTTAAAATTATNAAAATATTACTNAATTAAATCTTAATCTAAAGTAATTTTTTTAANTTAAATTTTATCGTTTATTTTTAAAATTTAATTTTATTTTTTTGATAAATATATTATTTATTTACTTTTACTTATTTCNTTACTCTATTCTCTTTATTATNAAAATTATTTTAATATTATCTATTATTTATTTATTATTTAATATTTTATTAGANANAATTTTATTGGNGANAATGGAGCTAAGAGCATTGCCAGCGCTATAAAAAAATGTCAAAATNTTACTNAATTGACTCTTGATTTANNGTAATTTCTTAAATAATTTTTATTTNCTTTTTAAATTTTCGTTGCTTTCGCTTTTTTATTATTTATTCCTCTATTTTCCTTATCTNTTATATATTGAATTTGTTTAACATTTTAATATTTATTTTTAATATTTTATTAGTTCTAATTCTATCGGCGAAAATGGAGCTAAGAGCATTGCCAGTGCAATANAAAATTATCAAAATCTCACTNAATTGNCTCTTTATTTAAGGTAATTTTTATTTGTTTTATTTTTNCAANCTTTATTGATTTTAATACTTTTTATATTATATTTAATATATTTNATATTTAATAAATATAATTACATAGAGATAATTAATTAAATGAAAACAGTAAGAGTAATTAATTTGCAATTGAAAAAATATTTCGAACACTTAGTTTAGACGGTAAAATTAATATTAAATTTTTTTAAAAAAGTATATAATTATAAAATTTATTTAAAAACAGGAATNTATTTATGAAATACGCAAATAAGAAAATACAAGTTTTGATAATATTATTTTTAATATAAAGTAAATAATAAATAAATAAAATATGATATTATTTAANTACATTTTAAAACATANATTTCATTTANAGAATATAGATTTNAATATTATAGTGTCTTTTCANNTCGANTGACACATATTTTNAGAAAAAAAATTTAANTGAGTANTTGATNNTAAAATAATGAATGGATNNAATAGTTANAGACAATTTAACAAATATGTCATAATAATNAANGTTCATTNCTTTTTANTTANATNATTTTATGGATTTTACTACCAAACTGNTAGGANAATTTAGAAACTAAAAGGTNTNATNACTTTANTAGAGCTGATGAAATNATCTATAAAACTTANAGAGCTAACAGTNTGTATTGTATTNACGAAAGATTTATGTTAGCATGAGAATGTTAAATAANGCTATATGNTTTACTANTTTTGTTAATTTAAGAATCTTAAAATTAAAAAGTATAATCNTTTCAATAGAGCTAATGAATTTAGATTTTGCTNNAATTNATANATACTAATTNGTGTTATTAATCCTCGANACGTTCATAGGAAATNAANAGAAANATNTTTNTTTATTATATTNATTGTTCTTGAGATATTCAAAATAAATCTGATTAGGATATTTNGTTAATTNTATTGAAAATGAACATAGAAGTAATGATTAAATT

>T.malaccensis_rnd-5_family-396#LRR_CRS3

AAAAAAAAAAAAAAAAAAAAAAAAAAAAAAAAAAAAAAAAAAAAAAAAAAAAAAAAAAAAAAAAAAAAAAAAAAAAAAAAAAAAAAAAAAAAAAAAAAAAAAAAAAAAAAAAAAAAAAAAAAAAAAAAAAAAAAAAAAAAAAAAAAAAAAAAAAAAAAAAAAAAAAAAAAAAAAAAAAAAAAAAAAAAAAAAAAAAAAAAAAAAAAAAAAAAAAAAAAAAAAAAAAAAAAAAAAAAAAAAAAAAAAAAAAAAAAAAAAAAAAAAAAAAAAAAAAAAAAAAAAACTAATTATNAGATAAAATTTTTATATGAAATATAATTTAATTANCTTTATNATATAAANAGAAAATGCAAATCCACTCTTAATGCATTATTAATANTGCGGATTGNAATGATAAACTAAAGAATCAATGCTATTCGATTANAAAAATATATNTAGAAGTGTTTTTTGGTNAGATGANAACTATGAAAATGAGAAAATATTAATAATAAACTCTTAAGTATTTTTATAAAAAATACANCGAGCCTTGATTTAAATTGTTCGAATNTTTNGCGGTAGNAAANAGCNTCTGAAAAGAGATGTTGGNATATTACACAATTGANTNAGATTATTTTGNTTTGGAGTTCCATTTTGATCTGAANNANAAATTTGNAACCATGTTTATTAAAAAGTTTCTGAATTAATCACAAANTGGCCAAAAAGNTTTCAAATTTTAATCAGNAATTNTGTTTCTGCAAAGTAACACACACACAAAAAAAAAANAATGCAGATCCACTCTTAATATATCATTAATATTGCAAATTTAAAGGAAAAATTAGAGAATGAATGCTANNCGATATATAAANATATATTTAGAAGTATTNTTTNATAAGATTGATNAATCAAAATGAGAAAAACTTNNTAATACAAAAGATAAATTCTGTATTTTGTTAAAACAAAAATTTTTTAAAATATTGATTGATTGGTTNAAATTGTTCGANTNCTTTGNGGTAGGAAAAAACATCTGNAAAAGATATTAGTATATTACACATTNTAANTANTTCAGACTTTTTGCTTTAGAGTTCTATTTATCTGAATAAAAAATTTGAAATTNATTAAAGANTTCCTCAATTAATCACAANTNAGTAAGCTTTCAAATTTTAGCCAGAAATTTTGTTTCTNCAAAGTAACACACANAAAAATAAAGANCATAAAATAAGACTAGAATCTAGCTANGCATATAATTAGCTAATAAACTTCTATAAAATNTATTTTACCTAGTCTAAGGCAACAGCGAGCGTATNTATATTAGCTTAAAGCGATNTATNNATTATNGCTNCAAGATCTTTATGAATATCTTTTNGGTTTCNTCGAGACATTTATTTTATTTAAAATTATATTAAATTCTAAATAAATTTNATTTCTACTAATGTTTTAAACTAANNTATATATTTATATTATTATAAGGCTATTNAAGTAATTTNAANTTCATTTATNTTTATTTNANATTTATGAGTGANTATAAAAATGGATAAATGCTATCATTAAANTTTACNAACTATNTTTATANTAGGAATANAGTTATTTATATANTNAATAAAGATATTTATTATATTTTAATCGATTTCATTCATAAAATATTTTGTACATATTTTTTATNAAACTANTTATTAAAGNNAATATTATTGCTATTATNGATAATCTANTATATTAANTTTTAACNATNAAGTTGAAAAGTTGTTNGTATATTTCAAGANTAACCAAAGCTTCCTCTTGATGCGNCTATTGTAGTCAGCTAAATTANTCTAAAAAAAAAAAAAAAAAAAAAAAAAAAAAAAAAAAAAAAAAAAAAAAAAAAAAAAAAAAAAAAAAAAAAAAAAAAAAAAAAAAAAAAAAAAAAAAAAAAAAAAAAAAAAAAAAAAAAAAAAAAAAAAAAAAAAAAAAAAAAAAAAAAAAAAAAAAAAAAAAAAAAAAAAAAAAAAAAAAAAAAAAAAAAAAAAAAAAAAAAAAAAAAAAAACAAAANAAGTCTCNAAAGAATGCCAGATCCAGNACGNCTNGCTNGAGAGATCAATNATCNAATTGATGCNNAGCTAGAAAAATTATTNCTAATATAATGGATTCGAGTATTTANGCAGCGGNGCNTNTGCCNTNGTNTTAAGGGCCNANAATCAAGACNAAGTAAAATCGCGNAGTCGCCCTTAAGNTCTTGANCNTGTTCTNACNAGGAGGATGAGAANGGCANCGAGTCNCTGAAAAAAGAGTATGAGNTGCTNCAGNAGTTTAGCCANTCTGATTNTCTAGTCAGTGTCTATGATTGCTTCTTCCTATCTAATGGAAGAANATGTAAGAANNGGATGAAGATGGTAATNAAAANACCAAATTTGTAAACAGGAGAGAATGTNCTNTTTTGTNATGGAAATGGAGCTTTGTGAACGTAAACAANNTTAAAAAAAAAAAAAAAAAAAAAAAAAAAAAAAAAAAAAAAAAAAAAAAAAAAAAAAAAAAAAAAAAAAAAAAAAAAAAAAAAAAAAAAAAAAAAAAAAAAAAAAAAAAAAAAAAAAAAAAAAAAAAAAAAAAAAAAAAAAAAAAAAAAAAAAAAAAAAAAAAAAAAAAAAAAAAAAAAAAAAAAAAAAAAAAAAAAAAAAAAAAAAAAAAAAAAAAAAAAAAAAAAAAAAAAAAAAAAAAAAAAAAAAAAAATGCTTTTTNATTGATATTGATTGNATTTTNGANNGTTTCTTAATTAAGTCTTCCAGTACTGNAATTTTAATCAGCACTTTATNATTATATTTCNGTAAAATAGTAATAAACTTAANTTTTATTTAAAANNAATTAATTTTAAATATATTAAAGTAAATTTAATTATTNTTTAAATAAAGTTTATTAAATTNTAATAAATATTTTTTTGAAAAGACATTTTTAATTAATTAATTAAGATAAANTATAAATAATTCACTTTAAGACTANTGAAAATCACTATTAGATTTTTTGCAANATACCATTTAGACTTAATATTACTTTTGANTTACNATAAAAAATAATTTAATTTTNNTTTATGTAAAAAGAAAATCTTAAGCAATTGTTCGATTTCTTNAGAAANGGNTAACCACCNTCTAANGAAANCAAGGAAATAATNGCTATTCAAATGTTAGAAGGATTGAATAACCTTCACGTCAAAAACATCATGCACAGGGACATCAAGCCTCANAATTATCTNGTTTGCCCTTCTGANACTTATGGATTNACCATCAAGNTNTGTAAAGAANTCTNATTTTAACTAACTTTATTANATTAAAANACTGTTGTTAATTTATTAAGGTGATTTGGGTTTCGCCTCTGCAGTAACAAAATCNAAAAGCTNCCTATCTANGGTAAATANNAAGTATTAATTTNANATANCTTTAAATNTCTTTTATTTATAGAAAGGNACAGATGACTATTTCGCTCCTGAAGTTGAAAAAGGANAATCGAGAATTTAGGTTTTTAAATNNAATTNTTTTTATTTNAAATTCANTTAAATTTTTTAAAAAGTCNGACCTGTATTCNCTGGGACTAGTTTTGCTAGAACTAGATAACTTAAATGAGCTGACAGAAAATTGGATTGATTCAAGTACAAAAGTAGAAATTTAGTAAGGAAGAGGAATNGCTTCNAAANATTAGATAGATAGGAAATCTAATATTTACAAAATAGCATAGATNTGCTTGAAANAAGATTATTAATAAAGAACGNCTGCAGGAGANCTACTTTCTGAACTTATCGAGCTTCATGGAAAACCACTAAAATTTGCACTCACATCTATGATATTGGAAGTAATAAATACATTTTAAAAAATTTCTTCTTACTAATNTAGTTTATAACTTAAATCAAAATATTCAACTTTANATAGATAAATTCCTTCAAATTTGCTTAGNAATTAAAATTAAAGAATCTATAATTATTAAATTAATTAAATTTNGATAATCGAATTGAATCATTTATTACTATTTTTNAATAATAGGAGTCAAGATACCTTAACAAGCTTAACAGATATTCGATAAATTAATGAGCNGCAAAAANTTATGGTGAAGATTTTATGAAATGANCTTCTATTTTATTAAATTATTTTATTTAAGCAAAATTAACCTTAATTTGAGGANTATGCAAAACANTTACTTGATAACACAGATTCNAAAGTGAAGGATAAAGATGGTACTAAACAATTTACANAGGTGGAGGTATTATCAAAGCTACTAAAAAGCCTATNTGAGGANAAGAAGTATGCAAACAACTTCCAAATTCTCAGTTTTGGAAGCTNTGGAATGGTATTAGCNACTAAAAAAGTTAATTCCAACAACAAGGAAATAGTACTTAAAATTCAAAAGATTGANGATGATTAAGCAATTGAAAATGAAATCAACTATTATGAAATAATTGAGGGAACCACTNGTAGTGTAGCTATACGATAGCTATGTCATAGAGAAAGCCACGGCACCTGAAAGATATGCAGTNTTTGAATTAGAAAAGTGCTCATGTAAANTTTTTGACTAAATTCAAAAATATCACAAANTTGTCTTCTTTAATTTAATTCGGNCTAAAANTAAATTTAATTTATGGCCANTAAAAACTTTAATANCNTATAANAAAATCTAGCTGCAAAAATATTTAATCTTGACATNATGAGATGTGTNATAATNTTANTTTGTTGTTTATTGTAAAAAAGGTTCGCTACAAGATTATATTGAAAGGTAATCAAAAGATGGTGAATTTAGTGATGAAGANAAACTAATTATTGCTAAACAAGTAATAGACTGTGTTAATTATATTCATTGGTTTAATATAATCCATAGAGATATTAAACCTGANAATTTTTTAGTTTGTNTAGATGGCAATCAACCTGAAATCAAGTTATGTAATAAATATCTTTATTAATTTGGAATAAATTTAATNTTTATAATTAAAAGGCGATTTCGGATTATCAGCTTAACTTGNATAATTCAGAATCTATCGAAATTTTAGATGTAAATTTTAAAAANTNATAAAAAAANTAATTGAATTATGATTTGATAAAAATTTAGAGAATAGGTAACTATTCATATACGGCACCAGAAATTTTAACTAAAAAAGAAAATGAAAATAAGATTTATTCTAAAAAAGTAATTATTTTATTCTTTTTAAAAATTAAGTAAATATTTTTTATTTTAGTCCGATTCTTATTCTGTTGGTTTAATACTAAGTTTGCTTGATAATTATCTAGAGTTCGAACGAAAAGACTTATCATTTACGAATTTAACGTTTAATAAATTTGATTAGCCTTTCAATTAAAATATAAATGTTAAGGATAACACAGAAATATTTAAATTTATAAAACTCTTANTTGTTTGGGATNAATTAAANAGAGCTACTCTTTCTGATATTGTAGAATCTAATCCTAAAAAATTCGTGTCGAATTCGTAATAGATGAAANAAATTAAATCTATAATTTCCCAAAAGTCAATTGAGGATATCTANAAAATAAAAATAACAANTATGGATGATTTGCATAAAATTAAAGAGTTTCAAATCGTTGAAATAGATTTAGGATAAAGTNTATTTAAGGTAAATTNATTTAGAATGATCACTTAATATTCTNTATTTTATAAAACATNATAATTTAAGATCCCCTCTTAATTTANTATTATTTATTTTTTTCTATAAATTGTCTAAAAATATNANATACTTTATTCGNTTGGTAAATATTGTTATTTTNNACAANATNTTNGACCAAAATTAGCAAATAGATCTAAAAGATTTTTNTTAATTCGTTNTANATCTATGAAATTANTTTTTCANTTAATTGAGGAATTTANTNTNATTATTAATCAAATTTCATNGNTATTTTATTTAAAATAAATCTAATATTTTAATTNTNAATGTAATGACGANTTGANGATTTGNATAAATNTTAAAAATTAAAACTTAAATCTTGTCGATTGANTTAGTTATTAGCTAATTAAGCAGTTTGTCATTTACTAACTANTTGCTTATTTACTTACTAACCAAATAAAAAATAACGAATTTCTTTACTCACTCATCACAACACAACTAAAATACTAACGAAAGCCATCTTTTCTCCCCTTTCTCCTCCATATTTATTTAATTCATTATTTATTCTTATATTTAAAGNTCGAATCAAATTGGTGATGTGGGTGCGAAAGATTTAGGCACAGGAATAGCCTAGTGCAAGAATATCACAACTTTGACTCTTTANTTACGGTAAAATACTTAATTTATTGATTNATAAACTAATAATTGAATTTATTATTTTGTTGATTGAATTAAGCTTTTGACTTAGTTACTAGCTAATTAAGACAGNTTGTCATTTACTAAATAACTTGCTTATTTACTTATTNACTTACTAACCAAATAAAAAATAACGAATTTCTTTACTTACTCATCACAACACAACTAAAATACTAACGNAAGCCAAATNTATTTTTACCATTCTTTCTCCTCCATATTTANTTAATTCATTATTTATTTTTACATTTAAAGTTNNAATTAAATTGGTGACGAAGGTGCTAAACATTTGGGCNCAGGAATAGCCNAGTGCANGAATATCNCAAGCGTTAGACNNTTNAGTTGNGGTAAAANNTTAATTNATNGATTGATAAAAACTAGTCTATTCAAGNTGTTTGNTTACTNTGTTGANTGAANTANGCTNTTGAATAAAGAACTTNGTNANTAGCAGAAGTTACAATAAAATAATTTCCTGATTAAGCCAGCTTATTGATAGCAAATTTTAAAATTCATTNTTTACTNACTTATTTTATAAGNTCNAATNAANTNGGTGANGAGGGNGCAANANGCTTAGGCACGGGANTNGGCTAAGTGTCAAGAATATGACAATTTTGAGCCTTGGNCTNNGGTAAAAACTCAATTTATTGATTGATAATCTAATAATTGAGAATTGATTATTTTATTGATTGAATTAAGCTTTTTACTTAATTTTTAGATAAGTAAGCCAGCTATCATTTACTAACTTGTTTATTTATTTACTTATCAAAAAAAAAAAAAAAATCTTCTTTACTTATTCAACACAACTTAAATACTAATTAAAGCCATCTATTTTTATCATCTTTTCTCCTTCATATCTATTTAATTTATTATTTATTCTTAAATTTAAAGTTCAAATCAAATTGGTGATGCAGGTGCAAAAGATTTAGGCACAGGAATAGCCTAGAACAAGAATATCACAACATTTATGCTTGACTTAGGGTAAAAACTTAATTTATTGATTGATAAACTAAAAATCGAGAAATAATTATTTTGTTGATTGTATTAAGATAATAAATAATAACAAGTCTCATACTTACTCATCACAACAAAACTAAAATACTAATGAAAGCCATCTTTTTTTTACCATCTTATCTCCTCTAAATTTATTTAATTCGTTATTTATTCTTATAATTAAAGTTCCAATAAAATGGGTTTATAGGGTGCAAAAGGCTTAGGCACAGGAATAGCTTAGTGCAAGAATATCACAACTCTGACGCTTGACTTACAGTAAAAACTTAATTTATAAATTGATAAACTAATAACTGAGAATTNACTANTTTGNTNATNGAATTAAGCTTTCGACTTAGTTATTAGCTNNCTAAGCCAGCTTGTTTGTCATTTACTAACTTGCTTATTTGCTTACTTAGTTACCTTACAATACAACTAAAATACTAACGAAAGCCATTTCTTTACACATCCTTTNTCCATCTCATACTTATTNAATNAATTNTCTATNCAATTTATATTAATAAAGCTCCAATCAAATTAGCGATGCGGGTACAAAAGATTTAGGCACAGGAATAGCATAGTGCAAGAATATTATAACTTTGAGTCTTGACTTAGGGTAAAAACTTACTTAATCATTTGATAAACTAATATTTGAGAATTATATTTTGTTGATCGNACTAAGCTTTTGGCTTAGTTATTAGNTAACTAAGCCAGCTTGCGATTTACTCCTCAAAAGACAACTAAAATACTAAACTTTTTTTTTACCATTATTTCTCCTCCATATTTATTTAACGCCATATTTATTCTTATATTTAAAGTTCCAATTAAATTAGTGATGCGGGTATAAAAGATTTAAGCACGAAAATAGCATAGTTCAAGAATATCTCTATTTTGGTGCTTCAGTTATAGTAAAAACCTAATTTATTGATTAATAAACTAATAACTGAGGCTTATTATTTTGTTGATTGAATTAAGCTTTTGGCTTAGTTATTAGCTAATAAAGCCAGATTAGCTTGCTTACTTACATATTTACTTACTCATTATAACACAATTAAAATGCTAAAGAAAGCCCTCT

>T.malaccensis_rnd-2_family-9#LRR_CRS4

TTTAAATAATTCAANANANAAATTTATTATTTTTTTAAATATTCTTNGTANTCCNGGATAAAAANGGCGGTAATTCAGGCATGNAAAATTATTTAACTNTATTTAATATTTTCAAATCCTAACATGTTTATTGCTAATCAAATTTGATATAAANTTGCTNTTNAANACTACTGNAAAGAAAAANGATGTAGGTATAGANNNAATNTTNCNNNAATNTAAAAGATGTAACNANTNATNTTTTAATNCGCTNNTCCTNAANNATTATTCTTNNCANTTAAAAAGTTTTTAAGCTCTTCGCCTTAANCTCATACTGATCTCCATATTNANCTCNAGTAAATTNAATTTATTATGATNAANCNCAATTATTTTTCATTTTAATTATATAATTAATTAAAATTAGTNTTTTATAATATTTNTTAATTNTTTATTTAATTAAATTTATTTTTTNTCAGCGCTTGATTATTAAATTTATTAATTTTTTATCTTGATATATTTTCTTAATTTATTCTACAAATAAACTAATTATTTTTTTAAAATTTAAAAGTNATAANTNAATAGGTCTGAAGTGCATAAGACTTAGGTTCTGCTTTATCAAAATGCACTAATCTCTNAAGTTTAANACTCTATCTTACGTAAAAATAGTTTTTTTTTTTTATTATATTTTTTAATATAAAAATATAAAANAATTTTTATTTTTAAGTTTTTATTTAATGCTCTATTTTTCTAATTTATTTTTAATTATTTTTTTAAAATTTTTATTAATTTANAANNATTCCTTATATTTTCCAGTTTTTGCTCTTACGTTTTCTTCAATTTTAAATAGTAGCAATTAAATTGGCGCGAAAGGTGTATCAAGCTTAGNTTCTGGTTTAGCAAANTGTATTAATCTCACAAATTTGANACTTNATCTTNGGTAAAAATATTTGATTAGATTTTTTAANATTGATATTTTTTAAANAAAAAAAATTNTTTTATTTGTTATCTAAATTTATCATTTNCTCTAAACTCATACAANATNTAAAAAAGTGGAAATGAAATTAGTGATACAAGTGCATCTAATTTAGGATGTGCTTTAGCAAAACTAACTAATCTCTCAAATTTAACAATCTTTATGAGGTATAAACTGATTATTTGTTTAATACTATAATATTTTTGATTATTAAATTATTTGAACAAACACCATAATTATATTTATTGGATTGAAGCTTTTAAATTAATATATTATGATAATCTTTTTTTTATTAAATTTCAATTTTTGCATTGTTTAATGACTCCCTATTTTTTCTTAATTTTTACCTACTGTAAACAATAGTACTATGTTTATTATTTAAACTNTAAATAAGATAGAAACTTTTCATGTCATTTTTNAATTATGATAGNNTCTCCTTNTAAGCATATAAATANNTATGATAAATTAATTATANTTNCTTGAANATTTAAAGATTTTTGGCAATNAAATNGGCGNAGATGGTGCCTCGGGCNNAGGNTNTGNNNTNGNAAAGTGCACCAACCTNTCAAATTCGACNCTTGNTCTAAGGTAAAAGCGNCTTATTTCNTTTNGATTGTGATATATCTTAGCATCAAAATAGACTANACAANAAAATATTATTTTTATTCATTAGCTTTTGTTTGATTTATTCCCTTTATTAATTCAGATTATNTTTTTTTAATNTCTANTTTAAAAATTAGAATAAANTAANNAATTATATTCTCTTCAACAATCAAAAGNNACAATAAGATTGANGAAGAAGGTGCCTCATGCTTGGGTTCTNNTCTAGCANGNTGCNCNAATCTCTAAAAGAGTTTGCTACTCGATCTNAGGTAAAATAAATTACATACATATTTTATTTATTTTCTACTTAAATATAAAATGGATTTTAAATTTAACCTTTTATTTATTAGTTACTTTTATCTAAGTTTTGATTTTGGCTAAAAATAATATTTTTATTTGTTAATTTTTAAAAATCTAATATTTTTTTTTATAGTTTCATGCATATAATCTAAGTAATTAGTAATATTTTTTAATATTTAATATTTAAAAAGGGGTAATAAAATTACTGAACAAGGTACATCAGGCTTAAGCTCTGCATTAGGAAATTGTATCAGTCTCTCAAATTTAAACCTTTATCTTTGGTAATAATAGTTTAGTTATTTGGGTTGTAACATCATGTAGATATAAATGATTTTATAAGATAAAATTCATTTTTATTTATTAGCAATTAGTTTTTTATTTTAAATACATTTTTTCTTAATTTTATTCACTTTTTAGTTTGAACACTACTAAGTAATTTAGTTTTTATATGATATATATCCTTGGTTTTTCAATTGAAATAACTAGCTAATTGTATTTTATTCAATATTTAAAAAGTGAGAATAAAATTGGTGCATTGGGTGCTTTAGGATTAGGTTAAGGATTATAAAATTGCTCAAGTCTCTCAAATTTGACTCTGAATCTTTTGTAAAAGCAGTTTCTTTCTTATTTTCTATAAAAATTCTTTTAAAACAAAAATTTATTTTTTATTATTAAATTTTTAGTTTTTGGTTCTGGTTATGATTTTTGTTAATTGATCTCATTTTTTAATTTCTAAGCTTTTAACATATTTTCTTCATAATTTCCTTCTATTTAACTAACTGATAATAATTTTTATATTTAANNAGNAATNNTTTAAAATTAGGTGATNNAGGCGTTTCNGGNTTANGTTCTNCTTTAGCAAANTGTGCTGAATCTCTCAGATTTGACCCTTAATCTCTGGTATACATTCTATATTTATTTTTATTGATTGAATTGTATTTATATAATGTTATAATAATTATTTAGTTTATTATTTTGCTCTTGATTTGTTTTTTTTAATTTAAAAAGTGGTAATGAAATTGGGAATATTGGTGCTTTAGGATTAGGTTCAGCTTTAGCAAACTGCAATAAAATATCTAATTTGGAACTAAATCTTAGGTAAATATATTTATTTGGTTAGATTATTTCAAAAGGTATTTAAACAATTAAAATATTATAATTGTTTTATTTTTTTTATTATTATTGTGCTTTTTGTTATTTTTTAACTCATATTAATTTATTTACTATTACATATTTTTTTCTTTTCCTACTTAATTTATTTATTACATATTTAAAAGTGACAATAAAGTTGGTGAAGAAGGTGTTTTAAGCTTATGTTTTGCTTTAGTAAAGTGCAAAAATCTCTCAAATTTTATCCTTCATCTTAGGTAAATATTATTTATTTCATTTTGATTGTTTGATTTTTTTGTAGAAATCAACAAAGTTTATTCATTTATTTATTGCTTTATTTTTTGTTTCTTTTTGTATTTTTATTTAATTTAATTTCTTTTTCATTTTTAAATCTGACTTTGATTGTTATTAATTTTTTTTATTTTTTCCTATTTTTACTCATTTTTTCCTTCTTACATCTTACGATTTCATAAATATTAAAAGTTCTAATTAAATTGGTTGTAAGGATATTTCAGGCATAGGGTGTACTTTAAAAAACTCATCAAGTCTCTCAAGTTTAACACTTAATCTATTGTAAAAAAGCTAGTTAAATTATCTTTGATGGATTTTTAATATAAACATTTTTTTGGATTAATTATTTCTTTTTGATCCTTTTTTTGCTTCTTTTATATGATTTCGTTTGAGTATTTTGAAATTTTGGCTTGATTATTTTTGATTATCTTATTTTTTTCTTCTTTTCTAATTATTAAAAACTAATTAATTTTCTTCAATATTTAAAGTAATTGTTAAATTGGGGATTAAGGTGCATCAAGCTTAGGCTTAGGTTTAGCAAATTGTTCTAATCTGCATAGTTTGAACCTTTATCTAAAGTAAAATATTTTTTATTGTGTAAATTTTTTATAACATAAATAACAACAAACAAACAATAAAATTCTCTTATTTATTTACTTTTTATTTTTAAATTTGGTTGAGTTTTTGTTATAAATTTAACTGATTTATAAAACTAAATGTTTTTCTTTGATAAAAAAATAAGATTGNTTNTTCACTATTTACGNTTAANTTTTCCTTTTAAATGATCTATTAAGCTAATTATATGNTCTTCGATATATTTATCAAAAAGNGAGAATTAAATTGGTCACGAAGGTGCATCANGNNTAGGCTCANCTTTAGCGAATTGCNNNAACCTCTCAAANTTGACACTTGATCTTGGGTATTGTTTATTCTAGTTTATAATATTNTTCAANGTANNTGAGACTGAGTTAATAAAACAATATTTATTTAAATTCATATTCATTATATAAGCNTTTCNTGCTTATTAGCCTTANTTCAAATAAAAAATAATGAAGATTTCAAAGNANATTGAATATAATTTAATAACTTAAAATCTANTTAATCTNTGTTGNTTTGAGGTTAAGTTATCACAATCATATGAAATATGATTTCAATNAATTTTAAAATTAAAAGTGATTTTNTTAAATCATAAATGCTTAGTGTCAAATAAATGATTTCATGTATAAAGTNAAGAAAGTTGAATAATCTCATTAAATATTCGTAAAATATAAAATAGACTTTGAGTTTTTTTATACAATATTTGAAAAATTGGGANACATGATAAAATATNAATTTTTGTATTATTAGCTATATTATTCTNTTNATACTAAGTAATTTTTTAATTAAAATTTAATTAAATTTAGATAATATTCTNTTAATTATCATAATNTAATAAATAAATAAATTTAGAATATAATTAATTTACTTTTGTTTCTTTTTATTAATNTAATTAAATATTTGTAAAACTAATATAAAANAGATTCAAATTTTAAATTTAATGAATTTTNATTTNATTTTTTGAAAATTTATAGCGGAAGTAATTATNAAAATAAAATTTAAATATTTTTAAATANNCTATTTNTAAATTTAATATTTTATTTAATTTNNTTTAATAAAAATAACCTAAATNAAATATTNTACGTTTAATTATTCGTTTTAAAAATATAAAATANATAAATAAATATATTTTTTTATATTCAAAAATTTTTTTNATATNATCCATTCTATCTANCTTTATCGCTTATTTTTTATCANAAATTTATTTTTGATAACTAATCTTTTAGCTTTAAGGTACTTGCTCAATATTTTTAATTTGTGTGATCCATTAATTTNATTNNANCTATCAATACATATTATAGATAATTAAAAAATAATTAGAATAAATATTTAAATTATTTTTAATGAATAAAAAAAATTAAAAAATAACAAAATTAAATTAAAATAAATTTAAAAATAAATAAAAAAGTAAATAAATCTAATAAATAAAGANTATTTTTATTTTAAATCATTTTGATATTAAAAAATATCACAATCNAAAANAAATAAGCTGTTTTTACCTTAAATCAAGTGTCAAATTTGAGAGATTAGTGCACTTTGCTAANGCAGAACCTAAGCCTGAGGCACCATTTGCACCAATTTNATTNTANCTTTTTAAATATTNAAGAAAATATAATTAGTTANTGAAGAAAAAATATGAAATAAGAATAAATTAGTAAATAATTTAAGCAAAAATTNNAAATGAAAAATGGCTTAAATTATAAAAAAACGTAATCGAATCTAAAAAGTATAAATAAATAAAAAGAGTATTTTTGTTTTAANCCATTTTGATATTAAAAAATATCACAATCCAAAANAAATAAGCTNTTTTTACCTTAAATNAAGTGTCAAATTTGAGAGATTAGTGCACTTTGCTAATGCAGAACCTAAGTCTGAGGCACCNTCATGCACCAATTTAATTTNCGCTTTTTAAATATTGAAGAAAATATTATTATAGTTAGAAGGAAAAATGAANAAAAAATATNAAATAAATTAATGAATAATTAAAGCAAAAATTGAAAATAAATAATTATTAAAATCAAAAAAAACATAATCAAAATNAAATAAAAATANTATTTTNTTTTAATCAATTTTGATATTAAAAAATATCAACAATCAAAANAAATAAGCTGTTTTTACTNTAAATNAAGTGTCAAATTTGAGAGATTAGCGCACTTTGCTAANGCAGAAGCTAAGCCTGAGGCACCTTTTTCACCAATTTNATTTTCGCTTTTTAAATATTGAAGAAAATAATTAGTTAGTTAGAAAAATGAANAAAAAATATGAAATAAGAATAAATTAGTAAATAATTAAAGCAAATTGAAAATTAAAAATGACTTAAATTAAAAAACATAATCAGNATCAAAAAGTAAATAAATCAAATAAAAAGAGTATTTTTATTTTAANTCATTTTGATATTAAAAAATATCACAATCCAAAATAAATAAGTTGTTTTTACCTTAAATNAAGTGTCAAATTTGAGAGATTAGTGCACTTTGCTAATGCAGAACCTAAGCCTGANGCACCATTTGCACCAATTTAATTNTCGCTTTTTAAATATTNAAGAAATATTAATTAGTTAGTTAGAAGGAAAAATGAATAAAAAGTATNAATTATAAATTAATTAAAAATTTAAGNAAAAATTNAAAAAAAATAATCAAAATAAAAAATTTAACAAATCAAATAAAAATATCACAATCCAAAANAAATAAGCTGTTTTTACCTTAGATNAAGTGTCAAATTTGAGAGATTAGCGCANTTTGCTAATGCAGAACCTAANCNTGAGGCACCATTCGTCGCCAATTTAATTATCACTTTTAAATATTGAAGAAAATATTAGTTAGTTAGAAGGAAAAATGAAGAAAAAATATGAAATAAGAATAAAATAAAATAAATTAAAAAAATATCAAAATCAAAAAGTAAATAAATAAAAAGAGTATTTTATTTTAATCTATTTTGATATTAAAAAATATCATAATCCAAAANAAATAAGCTGTTTTTACCTTAAATNNAGTGTCAAATTTGAGAGATTAGTGCACTTTGCTAATNCAGAACCTAAGTCTGAGGCACCNTTTNCACCAATTTAATTNCCACTTTTTAAATATTNAAGAAAATATTAGTTAGTTAGAAGGAAAAATGAATAAAAAATATAAATAAAAACAAATTAGTATAGCAAAATTAAAATAAAAATAATTAAATTAAAAAAAAAACATAATCAAAATAAAAAGTAAATAAATAAATAAAAAAGGATTTTTNTTTTAATCCATTTTGATATTAAAAAATATCACAATNNAAAATNAATAAGNTATTTTTACNNTAAATAAAGTGTCAAATTTGAGAGATTAGTGCANTTTGCTAATGCAGAACCTAANCNTGAGGCACCNTTATCACCAATTTAATTATCACTTTTTAAATATTNAAGAAAATATNAGTTAGTTAGAAGAAAAATNAAGAAATAATTAAAATATTGAAAATGAAAAAAATGAATTAAATAAANAAAAATTAGAATAAANAAAAATAATTCAAAATATTTTAAAAAAAAATTTTNCTTAATNATAATTANAAAATAAATAAGATTAATAATAAATTTAATTTACTCGAGATCAATTATGAAGATAGTATGAGATTAAAGAGAAGANTTTAAAAACTTTTTTAATAATAAAATTNAAATTAATTNTATATTTTAAATATAAATGACTACGTCTAATCCAGCTTATAGAGATATTTATTCTATTATATCGTTTATTTTTTAGATATTNAATAAGTAAATTTTATNTAGTTAATTTANTATTATTTTTTATTAGATNAATTAGANTACTAAGAATAAATTAATTTGAAATCGAATATAATATTCATNATAAATAAGAGATAAAAGTAAATATAATATTTAGTNAATTTTTTAATAATTTTCATATCGAANAAATCCTTTATATATTTTAGAAANTAAGAATCACAAACTAATTAAATTAGTANATTTCTTTATTAATTCAAAAAAGTATTTATCAAAATTTAAATATTATTAAATTTAAATATTATTAAAAAAATATAATTTTTNTATTTTTTAATTAAAAAATTTGATATAAAAAATATTATTTTAATAAAAGCANATTGATAANATTTATTTAATTTAGCTAAATTATNAAAAAATTANTATAAATTAAATATTTTAAAATTTGAATATTTAGTTTTTGAGTTCATACTTGATTCGATTTATTTATTATATCTTACCAAATTAATTCTTATTAAAAAATAAATAATTAATTAACATTTTAAATTCATTAAATTTCGATAAAATTTTTTCTAACTAAANTAAAACAAAATAAATGATAAAATAGAATAAATATTTTAATAANCTAGAAGATGTAAGTATTTATTTTATAATTAATTACTCTTTAATTTTATTTTTATCATTAAAAAAGTTTTTAAACTCTTATTTTTAAACTCATACTCATCTCCATATTAATCTCAAGTAAATTTAATTATATAAAAATTANTCTTATTTATTTTATCATTACTATCAAATTAAAATTTTTTTCGANAAATATATTCATANATTTATTTATTATTTTTATTAAAATTTTTATTTTTCTATCTGATTTTTNATTTTCAANCCTTACTTAAATTAATTAATAGTTTATACTTATTCAATTTCTTATTCATTTTTCTTTCTAACTAACTAATATTTTCTTCAATATTTAAAAAGTGGANATNAAATTGGTGCGAATGGTGCCTCAGGCTTAGGTTCTGCATTAGCAAACTGCACTAATCTCTCAAATTTGACACTTNATTTANGGTAAAAACAGCTTATTTGTTTTGGATTGATATTAATATAAAAANGAGCTGAAACNAAAACAATCTTTTTACTTGATTNANTTACTTTTTGATTNTNACTTTGNTTTTTTTAATTTAAGCCACTTTTCATTTTNAACNTTTGCTNATTTATTAGATTATTTACTAGTTTCTTTATTCTTNATTCATATTTTTNATTCATTTTTCCTTCTAACTAACTAATATTTNCTTCAATATTNANAAAGGTNNAATNAGATTGGTGCNGANGGTGCCTCAGGCTTAGGTTNTGGATTAGCAAGCTGCACTAATCTCTCAAATTTGACACTTNNTTTANNGTAAAAACAGTTTATTTGTTTTAGATTGTGATATTTTTTAATATCAAAATGAATTGAAACAAAAACAATCTTTTTACTTGATTTAATTACTTTTTGATTCTCATTATGTTTTTTTAATTTAATTCATTATATTATTTTGAACCTTTGCTTATTAGATTATTTACTAGTTTCTTTATTCTTAATTCATATTTTTATTCACTTTTCCTTCTAACTCACTAATATTTTCTTCAATA

>T.malaccensis_rnd-4_family-176#LRR_CRS5

TATTATGATATTAATTTTATATTTTTANGATAAAGATCGAAAAAATAAAGAAATAAAATATGATTCATCAAAAATTTAATTNAAATATTTTTTATTTTTATTTTATTCATTAAATAATTAAAACATTTATTNTAATTATTTTTTAATTATCTCTAATATCTATTATAGTGGCAATTAAATTGATGAATCAGACANANAAAAAATATTGAGCAAGTACCTTAAAGCTAAAAGATTAGTTGTTAAAACTATAAATTTATGATAAACATAAAATGAAGCATAGAAGAAGTATCTTTTGTAAGCCATAAACTTANAGGTNATATTTTTATGTGAAATATATATNAATTACTAAAAGAACTTATATATAAATTGTGAAGCTAAAATTTATATTTTATATTTGTGAAAGAAACAAACAATCAATATAGATTTATGATAAAAAATAAGTAATAAAGATAGATAGAATTGATAAAATAAANAAATAATTTTTTAAATATAAAATAAATTTATTTATCTTTAATTTATATAATTAAANNTCATAAATAAAATTGNATTTACATAATAAATATCTTTTATATATTTAACTTTTTTTNAAAATAAAAGTAAATTTAAATATAAAATTTTAAATTTAATTTTTAATTAAAAATTATTAAAAATAAATGAAATAAGNTAAATATTTATTCTATTATTTTAATCGTTTTTAAGTTAATNCTTCAATTTNNTTATTNGAATATTTGTAAATTTGATTATTTTGACTAAATTTTTAAGTTTGAATTNAAATATTATAACTAAAAATTACATTTCGTGAATTTTCAAGTATTTTTGTAANTAAATATTTATTAAAACTATNTTAATATAGAATTTGCTTTCTATTTTTTAAANTTNCGAATTGTATTATTTATAAATAATTATTTATATTTTTAAACGATTAAGATTTANTAACCANATTTTAAAAGTTAAAATAATTTAAACTATTTAAATTTCTTANATAATTTCAATAATNTTTATTATTTTATATATTAAAATTTAAACGTATTGAACTATTATTAATCAATTAATCTATAAAATTAGTATTGTATTATTAAAATAGATTTAATATACTGCCAAATAAGATATAAATACTCTACTAATATTTAGTNAACANATTATTTTATTACTTAAAGTAATTTAAAATTTTNTTCGAAATAGCATATTTANTAANTAAAAAATAAATAAAGCAAAATCTTTTAAATATTTATGATAAAAAAAAAAAAAAAAAAAAAAAAAAAAAAAAAAAAAAAAAAAAAAAAAAAAAAAAAAAAAAAAAAAAAAAAAAAAAAAAAAAAAAAAAAAAAAAAAAAAAAAAAAAAAAAAAAAAAAAAAAAAAAAAAAAAAAAAAAAAAAAAAAAAAAAAAAAAAAAAAAAAAAAAAAAAAAAAAAAAAAAAAAAAAAAAAAAAAAAAAAAAAAAAAAAAAAAAAAAAAAAAAAAAAAAAAAAAAAAAAAAAAAAAAAAAAAAAAAAAAAAAAAAAAAAAAAAAAAAAAAAAAAAAAAAAAAAAAAAAAAAAAAAAAAAAAAAAAAAAAAAAAAAAAAAAAAAAAAAAAAAAAAAAAAAAAAAAAAAAAAAAAAAAAAAAAAAAAAAAAAAAAAAAAAAAAAAAAAAAAAAAAAAAAAAAAAAAAAAAAAAAAAAAAAAAAAAAAAAAAAAAAAAAAAAAAAAAAAAAAAAAAAAAAAAAAAAAAAAAAAAAAAAAAAAAAAAAAAAAAAAAAAAAAAAAAAAAAAAAAAAAAAAAAAAAAAAAAAAAAAAAAAAAAAAAAAAAAAAAAAAAAAAAAAAAAAAAAAAAAAAAAAAAAAAAAAAAAAAAAAAAAAAAAAAAAAAAAACAAATTATACTTATTAATATTAATTAATAAAGACATTCTAAAAAACCTCTTTTTCTANGTAAACATAAATANTACAAAATTAATAGCATAACTCATAAATAANAAATGGATTAAGTAGCTTNAAATAGCATTCATACTCTTCATAATTGTACCGCNCATCCNCANAACAAAGTTGCTTTTGTNCAGATTAATGGAATNTCTAACCAGNNTAACTCTAGCGTATTCTAATGCNTTGGTTGCTTCGAATAAGACTTATNATTTAGAGGAGCCAACCATATGCTCATCTAATCAATTGTTGAAGCTCAANAATCTGANTTCNATTACAAATGGCCACCNCTNAATGATCAGCAGATAATTAAAAAAATTCATTTGAAAGCTAATNCTCTNAATTCNACTTAGNCCATCCTCGATTAAATCGATTCATACTTCGGTNAGCTAAAAGACGAAATTANCTAAAAGATNGATANCGCTTNGAAAAAGGCTGTCAATCAAGCNCTAGAAATGCCATTTGGNAAAGANTAGATTTTGTAAAGGTACCAGGAGATANCTTAAATNAAAAGCTTNAGATCNNNACTTGTTCNNGACTAAAACAAGTCTTTCGATCTNTATCAGTAATCNTGTAAAGACTTCATTTAGCAAATNGAATNGCAAAAAGNCAGAAACACTCAGCTACTTTAAGAATTGCTCAATNAATGCGATAAAGTTCAAGGGCTGGCNAACTTCGACNAATTAAACAAAAGNAAATAANTCATNTTAGATTTTTTAGANACGCTNAACGTGTTTGAAGNTAAAACGGAAANTTAAGTTAATTCTGATGGTTAANTTNCTAGCNTTNAATCCCAACAATAAGAAAATAGCAANTTGGATNAGCTANTGTAATTGATTTCTAATAAGACNAATTACTGTAGCTAAGANTTTTTGAANGAAATNAGATAANAANTGTAAAAGCTAAGCTCGTTCTTTGAATAAATTTCATTTGANAAAATGNGCCAAGAAAATAAAAAAGCGGTCAGGTTTTCAGAGTTAAGTGATGAAAAAATNAACAATNTGAACGAATATGTTGAACATCTGATAAATATGTAAAATAATGNTTAATACGCTCAATNAGTTAAGAACTCACATCAAATATAANAGTTGCAAAGCGTCNTGGATAACAAATTTAATTTTATNGACNANGACTNTNAAGACAANNTGATGGAGTNTTTAATNGAAACATATCCTTTTTNAAAAAATCTNAANAGTGGTCAAATACACTNTGAAGAATAAAGTTTTAACATTTTAAGGNGNTTAGAAACAAANTATGTTAANAGTTTCGTTCAACTTAGTAAAAAAGAAATAGAATCTATGATGAAAACCAACAAAATTTTGGAAATAGTTAAACCTAAAAAGTTNNACAGGAGATAATTCANTNATTTGATTNTGAGTAAAGTGAGTTAGAAAGTATNTTAAAAATGTATCCTATATTTGATNTGATAGAGCTTAAAGAACCNTAGGTTAGTATCTTGGATGAGCTACAGCTCTAGAAAAGCAAATATTTAAATGATGGAAACTAACGTNTAGAAGTAGTTAAAAATACGAATAACCAANTTGAAATNTNNGTAGATGAAACTGACTACTAAGGGGGAAATAGAAATAAAGNATTTGCTACTAACTGCATTTCNTAGATATTGAGNAGAGACAAAAAATACATATTTAGGATTTAANTTAAAAAGGGAAANGGCGANNATTTTTTGATAGGATTAATGCAAAATAAGAATTTCGATAGCAAATNTGGGTATNNNGATAANATGAGCTATTGTTTTAAANTAGAAAACAATNAGATGAAATACTTATGGAGGCTANGGNATTGATAAATTTATAAAAGGAGATAATAATTAAGAGTTAGACGAAAATAGTACTTTAGAGTTGAGAGTTTGGTTANAAGGATAGTAACTTGAGGTTTTAGATTACCCAAATTATAACTATCAAGTTTAGATCANAGATGGATGTAAATAAAATCTACTTTAAAATGATCTATGTTTATACTTCTCTTTGGNATGCAATGTAACTNAGATAAGTATATATTANTNGAAGCANTGACAGCTGAGTAATTTTGAANATAACTGAATAGNTANAAAGTGCGAATTAATAATTATAAATAAATGNACCATTTAAATTTAATAAAACTTAGTNAATAAGTAGAAGNATTTTATAATTTAACGAGTATTTATATATGAAATATTTCAATATTTATTTTTTCAAATATTTTTTACAAAATTTATNAGAGATTGATTTATTATAATANAGCNATTATTTTATTTGTTGAAACANAAAATAATTTAGNNTATNTAGTAAANATTTTNTATTTTAAAAACAAAAATTTATATCAGAGTAATCATNTTTANNNTNTCANGCTGCTATCAAAAATAAATCTTGAGCATTTTTAAAATTTTAGNANANAAGTAAANAAATATGTAGATTACAACGTTAANTGTATGAATGAAAACNTATTTCGAACCTNTGCAAAAGTTATATCTAAAANAATAGCNAGCATCGTTGAATATAAAAATCATTTCTTCCACAAATTTAAAATANTTGCTCTTAGAAATCCAAATCGGAAAAATATGTTTATCATTTTACTTATTTTTTACTAAAAATTTCAAATTTTTCTTTGCATTGTATTTAATTAAAAGATATTTAATTAATTAAGATTAGAGGATTTTAACAACATAACAAATCAAAAGAAACCAACAAATAAAATGGATTAAGCTGTCTCAAACACNATTTCGGATTTATAAAATTGTATTTTTCACCCTAAAAAAAAGATTTCATTTGTCTAAGTTAATGAAATATCTACTCAACCTAACTCTAGCATATTCTAATGTTCTGATTGCTTCAAATAAGATCTATAGTTTAAAGGCGCTAATTACATGCTCATCTAAAATCTTATTGAAGATTCTGATTCTGAATTTAATTACAAATGGCCTCCCCTTAATGATCAGCAGATAGTTCAGAAGATTCATTTGAAAGCCAAGAATCTCAACTCTACNGAAACCGTCATCAATTAAATNAATTCTTATTACTCTCAGCTTAAAGATGAAATTATCAAAAAAATAGATATTTTTTAGAAAAAAGCAATCAATTAAGCTCTCGAGATGCCGTTTGGCTAGGACCAGATTTTGAAAAAGTACCAAGAAATATCTTAAATTAAGAGCTTAAAATCTCTGATTCTTTAAGATTAGAACAAATCTTTTGATCTCTATTAAAAATCATGTAAAGATTTCATTTAGTAAATATAATTACAAAAAGACAAAAACACTCAACTGCTTAATGATTTACTTAATCAATGCGATAAAGTTCAAGAATTAGTTAATTTTGATAGACTAAACGATGGCAAATTATTGATATTAGATTTTTTAGATTCACTTAACTTTTTCGAAGAACAAACCTAAAGCTAAGCTAGCCAAATTAATAACTAAACTAATATTGAATTGCCACAACGTGACAATAATAAAATAAATTTATTAATGTAACTAATTTTTAATTAGACTAATTACTGCAGCGAAGAGTTTTTGAATTAAATAAAACAATAATTGTAAAAATTAAGTTTTTTCTTTGAATAAATTTCATTTGAAAAAATGAATCAAGAAAATAAAAAATCTATCAGATTTTCAGAATTGAATGATGAAAGAATCAACAAAATAAATGAATATGTTGAGCATCTGATAAAAATANAAGACAATCCTTAATACCTCTAATNAATTAAGAATTCAAATAAAATATCTAACTTAAAAAATATTTTGGAATCAAAATTTAATTTCATTACAAATAACTACAAAGGCAGTTTGATGGAATATTTAACTGAAACATNTCCCTTTTTTTAAAATAATCTAAACTCAGATCAAATACATTCTGAAGAATAAAATTTTAACAGCTTGAGAAGGTTAGAAGCNAATTATGTAAAAGANTTTGTTAAACTAGTAAATAGGAAATAGTTTTTTGAAGAAAACCAACAAAACTTTGTGAATAGTCAAACGTGTAGAGTTCAGTAATAAATACTTAAATTATTTCNTNTTGAATAAAATGAACTATAAAATGTTTTAAAACAATTTCCTGTGTTTGATTTGATANAGATTTAAAGAGGGNATAAGTATCTTGGATCAGCTATTTTTNTAAAAAAGCAATTTTTAAAAGATGGAAACTAACGTCTAGAAATTGCTAAAAATTTGAATAACTAATATGAAATTNCTATCAATTAGGCNNACTATANAGGATNANATGGAGGTNGTAATACAAATTGTATTTCATAAACNTTGGAAAGNAACAAAAAATACAAATTTAGGATATAGTTTAAAAAGGGAAGNCGAGGTNNTTTTAGGATAGGATTAATGTAAAATAAGAATTTTAATAGCCNTAGNNGGATATTTATGANAATATGAGCTACTNACTTGAATAAAAAAATAGTAAGATAAATTATATTGCAGGTACAGGAATAGATAAATTTGTAAAAGGAGATTCTGCTAAATAATAGCTTGATGAAAATACCACTTTAGAGTTGAGAGTTTGGATAGATGGATAGTAACTTNAAGTTTTAGANTACCCTAAATTATAACTATCAAGTTNAANTNAAAGATGAATTTAAATAGAATCTGCTTTAANAAGATCTNTGCTTATATTTAGGTTTATNTGGAGATCAAGATAAATGCATCTTAAAAGAGNCATTTATTGTNGANTAATTTTGAAGGTTAAAATAAGAGTAAAGTTAAAATAAANTTAATGATTTGTGTTAAAANTATTTATACGAAATAATTTTATGAATTTGTANTTTAAGAACGTCATTAAATAATTAATAGAAATTTTATAAAAAATATATATANTAATATTTAAATAAATGTATTTTATTTGTTCATNAATAAGTTNTCATTTTAAGGTNGATAAAATTTGTTTTGNATTCTACTNTNAAAACTATAAAATTATAATCTTCTNTTGCTCAAAGGAATAGTNTTGAATCTTTTTAANANTNATTGCTGCTCAAATAAAATTCTTNGGTNTAANTAAATTATTTNAGGAATGTATAANTAGGATTTATTCGNAGTTAATATTNANATTNNGANATAATTAGTAAATTGATTGTATTAATAANCTAAAATTTAGAACAAAGAAAATGGTTNATNTAAAATTNNNCTAAANAANTTATTAATTCCAAGTNTAAAACCCTAACTNCTCTTTAAAAAATACAGTCTGACTTCCGAGNATCCTAAACCTAATTAAACAAAAAAAAAAAAAAAAAAAAAAAAAAAAAAAAAAAAAAAAAAAAAAAAAAAAAAAAAAAAAAAAAAAAAAAAAAAAAAAAAAAAAAAAAAAAAAACCAAATTAATTCTTATTAAAAAATAANTAATTAATTAANTATTTTTGTAATTTATTAAATTTAGATAAAATTGTTTCTAATTAAATATCAAAAAAAAATAAATGATAAAATAGAATAAATATTTTAANAACTAAAAGATGTAANTATTTATTTTTTAATTAATTACTCTTTAATTTTATTTTNTCATCAAAAAAGTTTTTAAACTCTTCACTTTAANCTCATACTAATCTCGATATTGATCTCCGGTAAATTTAATTTATTATTTGTCTTATTTATTTTATCGTTACTATCAAATTAAATTTTTTTTAAAGAAATATTAATNTNTTTTACATGCTATTTATTTAATAATNTATTTATATTTTTATATTTTTTTTTTCATTTTCAACATTTGCTTNAATTATTTANTAGTTTATTCTTAATTCATATTTTTTNTTCATTTTTCCTTCTAACTAACTAATATTTTCTTCAATATTTAAAAAGTNAAAATTAAATTGGTGNTAAAGGTGCCTCAGGCTTAGGTTCNGCATTAGCAAANTGCACTAATCTCTCAAATTTGACACTTGATTTAAGGTAAAAACAGCTTATTTGTTTTAGATTGTGATATTTTTAATATCAAAATGGATTAAAACAAAAACAATCTTTTTACTTGATTTATTTACTTTTTGATTCTGATTATGTTTTTTAATTTAATTCATTTTTAATTTTCAACATTTGCTTAAATTATTTACTANTTATTCTTAATTCATATTTTTTNTTCATTTTTCCTTTTAACTAATTAATATTTTCTTCAATATTTAAAAAGCGANAATTAAATTGGTGCAAAAGGTGCCTCAGGCTTAGGTTCTGCATTAGCAAAATGCACTAATCTCTCAAATTTGACACTTAATTTTAGGTAAAAACAGCTTATTTGTTTTGGATTGTGATATTTNATATAAAATTGATTTAAAACAAAATAATTTTTTACTTTATTATTNGTTTTTGATTCTGATTATGTTTTTTTAATTTAANTCATTTTCAATTTCACTTTGCTAATTATTTACTTATTTATTCTTAATTCATATTTTTTCTTCATTTTTNCTTCTAANTAACTAATATTTTCTTCAATATTTAAAAAGTTANAATNAAATTGGTGACGAAGGTGCCTCAGGCTTAGGCTCTGCATTAGCAAANTGCACTAATCTCTCAAATTTGACACTTGATTTNAGGTAAAAACAGCTTATTTATTTTAGATTGTGATATTTTTTGATATCAAAATNAATTTAAAACAAAAATAATCTTTTTACTTGATTTATTTTTTTTTTTAATTCGTTTTTNATTTTCAACTTTTGCTTTAATTATTTATTAGTTTCTTATTAATTCATNTTTTTTCTTCATTTTTCTTCTAACTAACTAATTATATTTTCTTTAATATTTAAAAAGCGAAAATTATATTGGTGCAAAAGGTGCCTCAGGCTTAGGTTCTGCATTAGCAAAGTGCACTAATCTCTCAAATTTGACACTTGATTTNAAGTAAAAACAGCTTATTTNTTTTGGATTGTGATATTTTTAATATAAAANTGATTTAAAATAAAAATACCTTTTTATTTATATTTATTTACTTTTTGATTNTTATTATTTTTTTTAATTTAACTNATTTTTAATTATCAAANTTTATTTAAATNATTTACTATTTTATTTTTNTTCATTTTATTTTTAATAAATAATATTTTCTTCAATATTTAAAAGGNATAATAAAATTGGCACTAAAGGTGCCTTAAGCTTAGGTTCAGCATTAGCAAANTGCACTAATCTCTCAAATTTGACACTTAATTTAGGGTAAAAAATAGCTTATNAATTTTAGATTGTGATATTTTTTTATATCAAAATTGATTTAAAACAAATATACATTNTTTATTTGATTTATTTNATNTCTGNTTTTTANTTTTNANTATNTTTTTTAATTTACTANTCATTTTTNATTTTTCATCATTTTCTATTTAACTTAACTT

>T.malaccensis_rnd-3_family-55#LRR_CRS6

TGAATATTTTTAGATTCTACTACTTTGTCTAGAATATTTAAAATGATATTGGNGATTTTTATTGANTGAATCTTCAACCTTANGAATAACATACANTATTCTATTCAAAANNTATNTGAAAATTTTTTATTAAATGTGAATTTATATTNTTCTTNGGTAATNCCTAAATATTCNACAANNAACTCTAAAATTAGATAGGCTAATTCAAATAAATTNTTCTATTACCATTTTCGATATCTTTATTTGGTAAATTTTATAACTTTAAATNTTAGTNAATTTANGTNGTNTTATTAGAATTAATATGAGAATTTTATTTTGATTCACAAAAGTATAAAAAATACGGACTTNNATCATAATACAGTTTGAGTTCNTAACTTAGGTATTTAATTCATTCTTTAGTAAANTTAANTTGNTTAACTAATTANTGCTTTTTAATTTCCAAGATCAAATATAATTTTTTNTACATCCTTAAACGCGAGTTACCAAAATTTAAAGTAATTATTTANAATGTTTTTTAAAGNAGATTGCAAACATTTTTTCCTTATTNTTGTATAGAATAATTGAATGTNTTAAAAANGATNATAATTCTCAACTAAAGTTTAATTGTAACTTGCNATGATACTGGTTTTACTCAAGTTAAAACNTTTTTTTTTATTGCANNTAAATTAGCCTNNTCAGTGAAATACTAAATAGNATTTTNTAAATNTTAATTTCGATATAAACAATTCAATTCTAAACTTTCAATTATCNAAACATAATTTTGTTACTTCTTTAAGTTACTANNTTTTTANTNATTGATATGANCNTANATATNAAAGCTAGCTACNNTNATNTAAGTAAAGCTGTNAGATAGTANATAACCAATCAGNATNTGTGAAACTAAATNTGATTNTNTNAAATANTTAAATANTNATATTAAAAATCATTTATGCTTNCTTGATGAGGAAATAANTTTAATNTTTAAAAAAGTTTCAATTTGGTGATTATTNTGGCAAANAGCNAAGAGGAAAACTNTTGNGGTANTNCTTNGAAAAAATAATAATGAATTATGTTAAAATTAAAAACGAATATTTATTAAACTTGAAGATACTTTATACNTAAANTAGGCCTATAAATAATTTATATTTNTAAAAATTTAAAATTGATAAGAAAANTTAAATTATAAGAAAATTTACACTTTAAATCTCAAGNTTTTTTTAGAAATATAAAAATAAAGAAATATTTATTAAATCTATNATTTNTTTAATAATATAATTTCTATCATAACTATGTNAAATATAAGATTAAATAATTTTTCTAACTCAANTTCANTTTTNACNTCGTTTTTCTCAAATTTTAAACATTAAATTATAATTCTAACCGTTATTTTCGTCAAATAAAATAATTTAAATTNTGAGTATATAATTAATTTACTAAATATATTCTATTCAAAATTTATATAAAATAAAATAAATTTTAAATTTTAAAAAATAAAAAAATAAATTTTTAATAGTCTTTTTATAAATCTACCATTTCGATAAATTTACATTAATAAAAAATAATNATTAATTTATATTCTTAGCTTAATTTTTAGATTTACAAATATAAAAATATAACGATAAATAAATCAATTTATTTTATATTTAAAAAAATTTTTTAATTTATCCATTCTATTTATCTATGTTTATCACTTANGCTTTCATAAAATTTATAGACAACTAATCTTTTAGCTTTAAGGTACTTGCTCAATATCTTTATTTCGTGTGATCCATCAATTTAATTGTCACCTATAAATAGATATTAGAGATAAATTTTAATTAATAAAANAAAAAAAAAATATTTAGTNAAAANTTTGATAAATAAAATAATTTTTATTTATTTTTACGATCTTTATCTTAAAAATATAAAATCAAAATCATAATCTNAANTAAAAATTATATAAATCAAGTAAAAATATTGTTTTATTTTTAATTCATTTTTATATTAATAAAATCTAAANAAATAACTGTTTTTACNNTAAATTAAGTGTCAAATTNGAGAGATTAGTGCANTTNGCTAATGCAGAACCTAAGCCTGAGGCACCTTCGTCGCCAATTTAATTTTCGCTTTTTAAATATTGAAGAAAATATTAGTTAAGAATAAATTATTAAATAATTTAAAGAAATGTCGAAAGTNAAAAATTAAATAAANTAAAAAAGCATAATCAGAATCAAAAAGTAAATAAATCAAGTAAAAATATTGTTTTAGTTTTAATTCATNTTNATATTAATATACAATCTAAAANAAATAAGCTGTTTTTACTAGAGATTAAGTGTCAAATTTGAGAGATTAGTGCANTTTGCTAATNCAGAGCCTAAGCCTGAGGCACCTTCATCACCAATTTAATTGTTACTTTTTAAATATTGAAGAAAATATTAGTTAGTTAGAAGGAAAAATTAGTAAAAAATATGAATTAAAAATAAACTAGTAAATAATTTATATCAGCAAACGTTGAAAATGAAAAATGACTTAAATTAAAAAAGCATAATCAGAATCAAAAAGTAAATCTATCAAGTAAAAAGATTGTTTTAGTTTTAATTCATTTTTATATTAATACAATCCAAAACAAATAAGCTGTTTTTACTAGAGATNAAGTTTCAAATTTGAGAGATTAGTGCAGTTTGCTAATGCAGAGCCTAAGCCTGAGGCACCATTTGCACCAATTTAATTNTCACTTTTTAAATATTGAAGAAAATATTAGTTAGTTAGAAGGAAAAATGAATAAAAAATATGAATTAAGAATAAACTAGTAAATAATTTATATCAGCAAACGTTGAAAATGAAAAATGACTTAAATTAAAAAAGCATAATCAGAATCAAAAAGTAAATCAATCAAGTAAAAAGATTGTTTTAGTTTTAATTNATTTTTATATTAAAAAATATCACAATCCAAAACAAATAAGCTGTTTTTACNCTAAATCAAGTGTCAAATTTGAGAGATTAGTGCANTTTGCTAATGCAGAACCTAAGCCTGAGGCACCTTCGTCACCAATTTAATTTTNACTTTTTAAATATTGAAGAAAATATTAGTTAGTTGAAGGAAAATGAATAAAAAATATGATTAAGAATAAACTANTAAATAATTTAAGTAAGGTTTTAAAATAAAAAATNAGATAAAAAACATAAATATAAATTNGAATAAATAAATAATAATATCTAAGAGAATATTTCTTGAAAAAAAATTTAACTTGATAGTAATNATAAAATAAATAAGAATAATTATNATANATTNAATTTACTCGAGATAAATATGGAGATCAGTATGAGTTTAAAGNGAAGAGTTTAAAAACTTTTTTAATGGTAAAAATAAAATTAAAGAGTAATTAATTAAAAAATAAATACTTACATCTTCTAGCTTGTTAAAATATTTATTCTATTNTATCATTTATTTTGTTNNTATTTAGTTAGAAAAAATTTTATCTAAATTTAATTAATTATAAAAATNTTTAATTAATANTTATTTTTTAATAAGAATNAATTTGGATAGAGAGATATAAATTTTTAAATTGAATCAGATCAAATATATCTTAAATTTTTAAATTTTTNATATTTAAAAAAATATTCGTTAAATTTTAGNTAAATATTAAAAAGATTCAAATAANATTTTTATATTAATTTTAAAAATAAAAAATTTAAAAATAAAATTTATTTATTTAAAAAATATTCTATTTAAATAATAATTAATATTTTTAATNAAAAAATAAATTTAAATAAGANTAATTATTAAATAATAAAAAATTTNATAAATANATTTAATTNAATANTAAAAATAAAATTTAATGATAAACATATTTATTATTATAATTANGATTAAAANATATAAANAANNTCTATATTAATGTNTTTATTTNTTTCACAAATATAAAATNTAAATTTTATCATTCACAATATTTATTATAAATTTTATAATAAAATATTATATTTCACCTTAAAATATATGTAAGTTTTAGCTTAAATAAAATACAACTTCTATATTTTTATCATAAATANATAGTTTTAACTACCAATCTTTTAGCTTTAATGCACTTGCTCAATNTTTTTTACCTTTGTGATTCATTAATTTTATTGCGACTATCAATNTTATGCTTTAGAGATAATNTAAANANTAGAAATCAGATTAAACATTTTAATTACATTTTAANCAAAATATATGGAATNAAAGTATTTAAAAAAANTATTTGAATTGAATTCTTGATGANNTAGTTTTTAACAGAGATTTNTAAAAAAAATATNTTNTTTCGATCTTTATTTTAAAAATGTAAAAATAATTCATTAATNATCAAGTCAATAAAATAAATAANTGAAGNAAAAGAAAAAAAAAAAAAAAAAAAAAAAAAAAAAAAAAAAAAAAAAAAAAAAAAAAAAAAAAAAAAAAAAAAAAAAAAAAAAAAAAAAAAAAAAAAAAAAAAAAAAAAAAAAAAAAAAAAAAAAAAAAAAAAAAAAAAAAAAAAAAAAAAAAAAAAAAAAAAAAAAAAAAAAAAAAAAAAAAAAAAAAAAAAAAAAATTCTATCACCAATTTGATTGTATCTTTTTATATATTGAAGAAAAAATTAATTAGTTAGAAGGAAAAATGAATAAAAAATATGAATTAAGAATAAACAAACTAATAAATAATTTAAGCAAATGTTGAAAATAAAAATGACTTAAATTAAAAAAAAATAAATAATCAGAATCAAAAGCTAAATAAAACAATTAAAAAGATTNTTTTAATATTAAAATGNTATCATAATCCAAAANAAATAACTGTTTTTACCNGAGATCGAGTGTCAAATTTGAAAGATTAATACAATTTGCTAATCCAGAGCCTAAGCCTGATGCNCCTTAGTCACCAATTTAATTTCTACTTTAATTAAATATTAAAAAAGATATTTATGTTAAAATAAAAAACNAATAAAAAATATGAATGAATAAANTAAATTATAATTTATATTANNAAANGTAGAAAATTAAAATCGANTTAAATTAAAAAAACATAATCAGAATAAAAAGTAAATAAATCAAATAAAAAATTNATTTTTTTTAATTCATTTTTATATTAATACGATATAAAATAAATAAACTNTTTTTACTAAAGGTCAAGTGTCAAATTTGAAAGATTAGTGCANTTTGCCAAAGCAGAANCTAAGCCTGATGCACCTTCGTCGCCAATTTAATTGTCGCTTTTNAAATATTGAAGAAAATATTANAAAGTTAGAAAAATAAANAAAAAATATGAATTAAAAATATAAAATTTAAATAATTTAAGTATAAGTTGAAAATAAAAATNATAAATTAAAAAAGCATAATAAATCAAAAATTTNATAAAAGTAAAAATTGTTTTTTTTAATTTGTTTTAATATTAAAAAATATCACAATNTAAAAAAATAAACTATTTTTACTATAAGTTAAGTNTCAAATTTGAGAGATTAGTGCATTTNGCTAAAGNAGAGCCTAAGTCTTATGCACCNTATGCACCGATTTAATTCNANCTTTTTAAATATTTAAAATAAAATNTTAGTTAAAATGAAAAATAATATAAAATAT

>T.malaccensis_rnd-4_family-5#LRR_CRS7

TTGTATTAGTNTAAAAATGAATTATTTTAACTAAAACAATCTTTTTANTTGATTCATATANATTTTGATTCTTATTACGCTTGTTTTAGTTTTTAAAATAAGATCGTTAACATAAATAATCGTTTAATTACTTATCAAAATTTGGTTAAAATATCTCTCTTTCTANTTTTATTGTTATTTATTAATACTTATTCTAAAAAATATTTATTTATAGCTTTAATTANTTTTAATGATTCACATTAATTAAAAATATTGAGCAAATACTTTAAAGCTAAAAAATTAGTTGTCTTAAAAAATAATTTTNTGATAAAACTTAAGTGNTAAACATAGATAAATNTATAACAAATTTCTAAATACTAANTAAAATTATTTATTTATCTNTATTTTCATATTTTTAAAATTAAAAAGTAAACTAAGANATAAATTTATGATTATTTTTATNAATGTAAATTTAATANAATGTAGGATTTATAAAAAGGCTATTAAAAATTTATTTTTTAAAAATTAAAGTTTTATTTTTAATTAGTTGTTATTTAATTTAATAATTTATTTAANTATTAATTTANATACATTATATTTTGTTNTTAAAATTAATACTNAAATATAATATTGTTAAATATTCCAAATNCTAAATTTTTATTTAATCAAATATTTTCAATATAAGAAATCGAAAAAAGAAATATTTGCTCAAGATTACAACGTTGCTTATTTATTAAAAACTTTCTAAATATTTAAATATCTCTTATTTATTAATTTTCTAAATTTATATATTTGATCTCANTTTTATTTATTCTCTCTATCCAAATTAATTCTTATTAAAAAATAAATAATAAATTAAATATTTTTATTAATTAAATTTAGATAAATTGTTTCTAACTAAATATCAAAAAAATAAATGATAAAATAGAATAAATATTTTAACAACTTAAAAGATGTAAGTATTTATTTTTTAATTAATTACTCTTTAATTTTATTTTTNCCATTAAAAAAGTTTTTAAACTCTTNTCTTTAAACTCACACTGATCTCCATATCGATCTCAGGTAAATTAAATTTATAATAATTAATCTTATTTATTTTATAATTGCTATCAAGTTAAATTTTTTTTCAAGAAATATTCTTATAGCTATTATTATTTATTTATTCAAATTTTTATTNATATCAATTATATCTTATTTTTNATTTTCAACCTTGCTTAAATTATTTAATAGTTCTCTATCCTTAATTCATATTTTTTATCGTTTTCCTTCTAACTAACTAATATTTTCTTCAATATTTAAAAAGTNGCAATNAAATTGGTGATGAAGGTNCTTCAGGCTTAGGTTCTGCATTAGCAAACTGCACTAATCTCTCAAATTTAACACTTGACTTNNAGTAAAAANAACTTATTTNTTTTAGATTGTGATATTTTTTAATATCAAAATAAATTAAAACAAAAACAATNTTTTTACTTGATTTATTTACTTTTTGATTCTCATTATATTTTTTAAATTTAAATAATTTTTAATTTTCAACCTTTGCTTNGATTATTTACTAGTTTNTTTTCTTAATTTATTTTTTATTCATTTTTCCATATAACTAACTAATATTTTCTTCAATATTTAAAAAGTGNCAATTAAATTGGCGACGAAGGTGCCTCANGCTTAGGTTCTGTTTAGCAAACTGCGNTAATCTCTCAAATTTGACACTTAATTTAAGGTAATATATTGTGATATTTTTTAATAACAAATTGAATTAAAAAAAATAACATTTTAACTTTATATGTTTTGTTTTTACGTTTTTCAAATTTAAGTTATTTTTTATTTTCAACCTTGCTTAACTAGCTTATTTNTTATTAATTTATATTTTTTTTCANTTTTCCTTATAACTAACTAATATTTTCTTCAATATTTAAAAAGCTGAAATTAAATTGGTGACAAATGGTGCCTCAGGCTTAGGTTCTGGATTAGCAAANTGCACTAATCTCTCAAATTTGACGCTTGATCTCGNGTAAAAACAGCTTATTTGTTTTGGATTGCGATATTTTTTATATCAAAATGAATTAAAACAAAANCAATNTTTTTACTTGATTTATTTACTTTTTGATTCTGATTATGTTTTTTAAATTTAAATNATTTTTATTTTNAACCTTTTCTTAATTATTTACTAGTTTATTCTTTATTCATATTNCTATCTTTTTCCTTCTAACTAACTAATATTTTCTTCAATATTTAAAAAGTCGTAATTAAATTGGTGACGAATGGNGCATCAGGCTTAGGTTCTGGATTAGCAAGCTGCACTAATCTCTCAAATTTGAAACTTNANTTNAGGTAAAAACAACAGTTATTTGNTTTAGATTGTGATACCTTTTAATATCAAAATGAATTAAAACAAAAACAATATTTTTACTTGATTTATTTACTTTTTGATTTTGATTATACATTTTAAATTTAAATCATTTTTAATTNTAAACTTTGTGAANTAATATAATTTATTCTTAATTTATTTTTTTATTCATTTTACCTTCTAATAACTAACTAATATTTTCTTNAATATTTAAAAAGTTAAAATTAAATTAGTNCGNATGGTGCATCAGGNTTAGGTTCTGGTTTAGCAAATTGCACTAANCTTTCAAATTTGATACTCAGCTTATGGTAAAAACACTTATTTTTTTAATTTATGATACTTTTTATTNATTTTTTAAAAAAAAAACATGTTTTTGAACTCTTGACTTTAAACTCACTTACTGATATTGATTCTTATCACGTTTTAAATTAAAGTCATTTTATTTTAANATTTAATTTAATTATTTACTAATTTCTTTGTTNTNAATTTATAATTTTTATTCAATTTTTTTAATAAACTTATTTTAAATATTTAAAAAGCTANAATTANATTGGTGANAANGGTGCCTCAGNCTTAGGTTCTGCNTTAGCAAGCTGCACTAATCTCTCAAATTTGACACTTGACCTCNNGTAAANATAGTTTATTTNTTTTAGATTGTGATATTTTTTAATCAAAATTAATTAAACAAAAACAATTTTTTTATTTGATTTA

>T.malaccensis_rnd-5_family-343#LRR_CRS8

ATTTGAAAATTAAAAATAATTAAATCAATAAAACTTAATTAGAATCAAAAAGTAAATAAATGAGTAAAAAGATTGTTTTTGTTTTAATTTATTTTGATATTAAAAAATATCACGATCTAAAACAAGTAAGCTGTTTTTACCCTAAATCAAGCGTCAAATTTTAGAGATTAGCGCANTTTGCTAATGCAGAACCTAAGCCTGAGGCACCTNCATCACCAATTTNATTGTAACTTTTTAAATATTGAAGAAAATATTAGTTAATTANAACTTTATGAATAAAAAATAGAATTAAGAATAAAGAAAATTTTTGATGTTCAAATCAAAANTTAAAAATAAAAGATTTTAATTAAAANCATAATTAGAATCAAAAATTAAATAAATCAAGTAAAAAGTTTGTTTTTATTTTAANTCATTTTGATATTAAAAAATATCAAAATTAAAATAAATAAGCTGTTTTTACCNTAANTCAAGTGTCAAATTTGANAGATTAGTGCATTTTGCTAAAGCAGAACCTAAGCCTGANGCACCTTCGTCACCAATTTAATTGTAACTTTTTAAATATTNAAGAAAATATTAATTAGTTTGAATAAATGAATAAAAAATTGAATTAAGAATAAAAAAATTAAAAAATAATTTAAAATGGAAAATATTAAATTAAAAAAACATAATCAGAATTAAAAANTAAATAAATCGAGTAAAAATTTATTTTTTTAAATCATTTTNATATTAAAAAATTTTACAATCCAAAACAAATAAGCTGTTTTTACTCTAAATCAAGTGTCAAATTTGAGAGATTAGTGCANTTTGCTAANNCAGAACCTAAGCCTGATGCACCNTTTGCACCAATTTAATTANAACTTTTTAAATATTGAAGAAAATATTAATTTGTTAGAAGGAAAAATGAATAAAAATATAATTAATAATAAACTAATAAATAATTTTAAAATAAAAAATATTAATTTAAAAAACATAANTAGAATTAATAAGTAAATANATNAAATGAAAATTTGAAAAAAATTAATGTAAATAAAATTAATAAAATATATAATTTAATTTACCCGAGATCGATATGGAGATNAGTNTGAGTTTAAAGTNAAGAGTTAAAAACTTTTTTATGGTAAAAATAAAATTAAAGAATAATTAATTAAAAAATAATTGATTACGTCTTCTAATATATTAAAATATTTATTCTATTTTATCATTTATTTTTTTATATTNATATAGAAACGTTTATCGAAATTTAATAAATTATAAAAATTTTTAATTAATAATTAATTTAAATAGAATATAAATTTGGAAAATAGATATAATAGATTCTTCATGTGTGAANTCAGACCNACTATACTTTTCAATTTATTTTATATTTTCTACAAAATTTCATTTAAATTTTATTAATTTTAATATTGGTTAATTGCTTACTCTTTATTAAATAGTAATAATAAATTAAATTATATAATNTAAANNCGATTCTAAANTAGCAATTTGAGATTTAAATGTTTATTTGACAAAGCCTTTGCAAAATACAGTCATATCATAATGACTAGTGATCGAATTTAGTTAATATCGAATATTTTTTTTTTAATAGAAATAAANTCGTAAAATAATATTTAGATTTTTGTTATGCCTACNNTTATTAAATAAATAACCTTAGTTCAAATGGCTAAAAATTTTATTAATTAGATTATTAATCTTTAATTATTTAAATATTTCCTAATTTTATAAATTATTTTTTTTNAATATTACATTATTTATTATTTTACANNGNAANTNTTTGNTAATTTTATTAGAATTTTTTTTTTTTTTTTTTTTTTTTTTTTTTTTTTTTTTTTTTTTTTTTTTTTTTTTTTTTTTTTTTTTTTTTTTTTTTTTTTTTTTTTTTTTTTTTTTTTTTTTTTTTTTTTTTTTTTTTTTTTTTTTTTTTTTTTTTTTTTTTTTTTTTTTTTTTTTTTTTTTTTTTTTTTTTTTTTTTTTTTTTTTTTTTTTTTTTTTTTTTTTTTTTTTTTTTTTTTTTTTTTTTTTTTTTTTTTTTTTTTTTTTTTTTTTTTTTTTTTTTTTTTTTTTTTTTTTTTTTTTTTTTTTTTTTTTTTTTTTTTTTTTTTTTTTTTTTTTTTTTTTTTTTTTTTTTTTTTTTTTTTTTTTTTTTTTTTTTTTTTTTTTTTTTTTTTTTTTTTTTTTTTTTTTTTTTTTTTATTTACTAATNAANNCGTTTACTTTATTAGTATNTTTAAAAAGCTTTTATAATAAATCGNAATTNACGGGNATTACTCTCAATAGGGTTTGTGTTAGAACTTACTATTATCACGACGGTTGTATGGTGTAGCGGTTAGCACCGAGGACTTTGAATCCTCTGACCTGGGTTCGAATCCCAGTACGACCTTTTTATTATTTTTATTTATTTATTTAAAAATTTATTTTTATTANATTGCCTCATAATNATTTTTTAAAATTTTATTTNTTTTANAACAGTAAAAATATTTTAANCAAATTTATTAATTNATGAAGTTAGTTATAGTATTTAAAATAATAATTGACGAAAAATTTTAGATTTCAACTTNTATTTCACGACGGTTGTATGGTGTAGCGGTTAGCACCGAGGACTTTGAATCCTCTGACCTGGGTTCGAATCCCAGTACGACCTTTTTTTAAAATCTTATTCGATTTTTTAATNAGAAATTAATTTTTAAAATATTTTTCGATCAAAATAAATAAATTTTTTGTTAAATAAAGAGATCATTTATTTTAAATTGAATTATTAATTTAAAATAATTATTTATTTCTTGAAAACGAATGAATCAGAATTATTANNAAATANACATGAANGAAANTGCTTTGTGAATTTATTTATCTATTATTAAATTTTTTTTTTTTTTTTTTTTT

>T.malaccensis_rnd-3_family-56#LRR_CRS9

AAGAATAAACTANTAAATAATTAGTAAGTAAAGTTTGAAAATGAAAAATCGGATATAAAAAATTTAAATTTAAATCAGAATAAATAATGANAAAATTTANGAGAATATTTATTCAAAAAATAAATTAAATAGATAATAATATAAAATAAATANTAATAATTTTTATATATTAAATTTACCTGAGATCAATATGAAGATATNTACGAATTTAAAGNTAAGAGTTTAAAAACTTTTTTAATNNTAAAAATAAAATTAAAGAGTAATTAATTACAAAATAAATACTTACATCTTCTAGCTTTAAATATATTTATTCTAAAATATAATTNATTTTATTTTATTTAGATAGAAAAAATTTTATCAAAATTTAATTTCACTTTTTAAATATTNAANAAAATTTATGTTANTTANAANAAAAATAANTANAAATTTATATTCTAAGTTTAATTTTTGNTTTTATAAATATAAAAATAAAGATAAATAAATCAATTTATTTTATATTTAAAAAACGTTTTTTAATTTATCCATTCTATTTATCTATGTTTATAACTTAAGCTTTATCATAANTNTATTNTTTTGACAACTAATCTTTTAGCTTTAAGGTATTTGCTCAATATCTTTAATTCGCGTGATCCATTAATTTAATTANAGCTATAAATAGATATTAGAGATAAGTTTTANCGAATAAAATAAAAGTAAAAAGAAAAAATATTTTTATTAAAACTTTGATAAGTAAAATAGTTTTTGTTTATTTTTATGATCTTTATCTTAAAAATATAAAATTAAAATCATAATCTTAAACAAAAATTATGTAAATCAAGTANAAAGATCGCTTTTATTTTAATTCCTTTTGATATTAAAAAATATCACAATCCAAAATAAATAAGCTGTTTTTACNAGAGATTAAGNGTCAAATTTGAGAGATTAGTGCAGTTTGCTAACGCAGAGCCTAAGCCTGAGGCNCCTTCGTCACCAATTTAATTTCTACTTTTTAAATAATTGAAGAAAATTTTAGTTAGTTAGAAGGAAAAATGAATAAAAAATATGATTAAGAATAAACTAGTAAATAATTTATATCAGTAAATGTTGAAAATAAAAAATGACTTAAATTAAAAAAGCATAATCAGAATAAANAAAGTAAATCAATCAAGTAAAAAGATTGTTTTAGTTTTAATTCATTTTTATATTAATACAATCCAAAACAAATAAGCTATTTTTACCATAAATCAAGCGTCAAATTTGAGAGATTAGTGCAGTTTGCTAATGCAGAGCCTAAGCCTGAGGCACCTTCGTCACCAATTTAATTATNNCTTTTTAAATATTGAAGAAAATATTAGTTAGTTAGAAGGAAAAATAAATTTAAAATAAACTAGTAAATAATTNATATCAGCAAAAGTTGAAAATGAAAAATGACTTAAATTAAAAAAGTATAATCAGAATCAAAAAATAAANTAAATNAATCAAGTAAAAAGATTATTTTAGTTTTAATTCATTTTTATATTAATATAATCCAAAANAAATAAGCTGTTTTTACCNTAAATCAAGTGTCAAATTTGAGAGATTAGCGCAGTTTGCTAATGCAGAACCTAAGCCTGAGGCACCTTCGTCACCAATTTAATTTTNACTTTTTAAATATTGAAGAAAATATTAGTTATTTAGAAGGAAAAATGAANAAAAAATAAACTAATAAATAATTTAAGCAAANNTTGAAAATGAAAAATGATTAAATTAAAAAAAAGTAATTAGAATTAAAAAGTAAATAAATAAATAAAAANATTATTTTAGTTTTAATNAATTTTNATAATAAANAATANAATCCAAAANAAATAAGCTATTTTTACTAGAGATCAAGNGTCAAATTTTAGAGATTAGTGCAGTTTGCTAANGCAGAACCTAAATCTGATGCGCCTTCGTCACTAATTTAATT

>T.malaccensis_rnd-5_family-439#LRR_CRS10

AGTTATAATTAAAAAACAAATTAATCAAATAAATAATTTAGAAATTTAAATTAATTTAATAAGTAAAAATACCTAAATAAAAAATAAATAAACTTTTTTACNNTAGAATAAGTTTCAAATTNGAGAGATTNGCGCATTTTGCTAAACCGGAACCTAAGCCTGATGCGCCTTNATCACCAATTTAATTATTTCTTATTAAAAAATTGAAGTAAAAATTTTTAATTTAAAAAGAATCTTAAATTAAAAANTAAATTAATCAAATAAAAANTAAAAAAATAAAAATTGATTAATTTATTTTTTATATTAAAAATATCTCAATANAAAACAAATAAAATGTTTTTACTNAAGATAAAGTGTCAAATTTNAGAGATTANTGCANTTTGCTAAAGCAGAACCTAAANCTGATGCACCTTCGTCACCAATTTAATTATANCTTTTTAAATATTGAAGAAAATAAGTTAGTTAGAATTTAGAAAGAAAAAATGAAGAAAAAATATGAAANAAAATACATNTTAAATTAAAATTAAAGCAAAATAATTTTATTTTAAAAATTAACTTAAATTAAAAAATAAATCAAAAAATAAAATAAATTAAAAAATAAAAAGAGTTNTTTATTTTAAATTNATTTTTATATTAAAAATATNATAATCCAAAATAAATAAACTGTTTTTACTNNAAATNAAGTNTCAAATTTGAGAGATTAGCGCANTTTGCTAAANCAGAACCTAAGCCTGATGCGCCTTCATCGCCAATTTAATTNCAACTTTTTAAATATTGATAAAGAAAATATAGTTAGTGAATTATTTAGAAGGAAAGATGAAAAAAAAATATGAANAAATAAATATTTTAATTTAATAAAATAAAAATTCAAAAAATAAAAAGAATTTAATTTATTTTTTATATTAAAAATATCTAAATCCAAAATAAATAAACTGTTTTTACCTTAAATNAAGTGTCAAATTTGAGAGATTAGCGCANTTTGCTAAANCAAAACCTAAGCCTGANGCACCTTCTGCACCAATTTNATTNTCACTTTTTAAATATTGAAGAAAATATTAGTTAGTTAGAAGGAAAAATGAAGAAAAATTAAGAAATAAATAATATTAAAATCAAAANTNATTAATAAAAAAATNTTATAATTTAAAAAACATAATCAGAATCAAAAATAAATTAATCAAATAAAAATTAAAAATAAAAATATTTTTTTGTTTTAAATTCATTTTCATATTAAAAATATCACAATCCAAAACAAATAAACTGTTTTTACTNTAAATNAAGTGTCAAATTTGAGAGATTAGTGCAGTTTGCTAAAGCAGAACCTAAGCCTGATGCACCATTTGTCACCAATTTNATTNTNACTTTTTAAATATTGAAAAAAATATAGTTAGTTAGTAGGNAAAAATATGAAATAAAATATTTGTAAAATAATTAGNATAAATAAAAAAATANTTAAATTTAAAAAGCTAATTANAATCAAAAGTAAATAAATNAAATAAAAATAAAAAATAAAAAGAGTTTAATTTATTTTTATATTAAAAATATCACAATCCAAAACAAATAAATTGTTTTACCTTAAATNAAGTNTCAAATTTGAGAGATTAGCGCANTTTGCTAANNCAGAACCTAAGCCTGATGCACCNTTNGCACCAATTTNATTNTAACTTTTTAAATATTGAAGAAAATATATAGTTAGTTTAGAAGGGAAAAATGAAGAAAAATATGAAATAATAAAAATAATTTAAGCAAAGTTGAAAATNAAANATGACTTAAATTAAAAAATAATCGAATTAAAAATAAATAATTAAAAAATAAAAGAGTNTTTTTGTTTTAAATTNATTTTCATATAAAAATATCACAANCCAAANAAATAAACTATTTTTACTTNAGATNAAGTGTCAAATTTGAGAGATTAGCGCANTTTGCTAAACCAGAACCTAANCCTGANGCACCTTCGTCACCAATTTTATTACCACTTTTTAAATATTGAAAAAATATAAGTTAGTTAATTAAGAAGGAAAAATAAAGAAAAATATGAAATAAAATATTTATAAATAATAATTTAAACAAAAAGTAATTATTTTAAAAAATAATTAAATTAAAAATAAATTAATTAATCGAATAAAATCAAATAATAAAATANANTTTAAATTTATATTTTAAAAATATCAAAAAACAAGCAAATAAANTAATTAAAAACTAACGAAATAAAAAGAGTTTAATTTATTTTTTGTGCTAAAAATATCTAAATANAATNNAAATAAATCGTTTTTACTCNAGGTCAAGTGTCAAATTTGAGAGNTTAAAGCANTTTGCTAAANCAGAACCTAAATCTGATACACCTTNATCACCAATTTNATTTTCACTTTTATATATTAAANAAAATATATTAAAAATTTTTAGAAAGTAAAAATGAAGAAAAAATATGAAAAGATAAAAAAATAAAATAAAACTTAATTTATTTTAAATTTATTTTTTGAAATAAAAATATTAAAAAATTAAATAAATAAACTTTTTTACNANAGATNNAGTTTCAAATTTGAGAGATTNACGCATTTTGCTAAACCAGAACCTAAANCTGATGCACCTTCGTNGCCAATTTNATTATCNCTTTTTAAATATTNATAAAAATAATTGTTAATTGATATTAGAAAGAAAAAATGAAGAA

>T.elliotti_rnd-3_family-51#LRR_CRS1

AAAAAAAAAAAAAAAAAAAAAAAAAAAAAAAAAAAAAAAAAAAAAAAAAAAAAAAAAAAAAAAAAAAAAAAAAAAAAAAAAAAAAAAAAAAAAAAAAAAAAAAAAAAAAAAAAAAAAAAAAAAAAAAAAAAAAAAAAAAAAAAAAAAAAAAAAAAAAAAAAAAAAAAAAAAAAAAAAAAAAAAAAAAAAAAAAAAAAAAAAAAAAAAAAAAAAAAAAAAAAAAAAAAAAAAAAAAAAAAAAAAAAAAAAAAAAAAAAAAAAAAAAAAAAAAAAAAAAAAAAAAAAAAAAAAAAAAAAAAAAAAAAAAAAAAAAAAAAAAAAAAAAAAAAAAAAAAAAAAAAAAAAAAAAAAAAAAAAAAAAAAAAAAAAAAAAAAAAAAAAAAAAAAAAAAAAAAAAAAAAAAAAAAAAAAAAAAAAAAAAAAAAAAAAAAAAAAAAAAAAAAAAAAAAAAAAAAAAAAAAAAAAAAAAAAAAAAAAAAAAAAAAAAAAAAAAAAAAAAAAAAAAAAAAAAAAAAAAAAAAAAAAAAAAATAAGAGCTTTGGTTNCTTTAATTTTTCANTTTTATTTTAATANAAACTNAATTTTATTAGAAATAGAAATTAATTTAATTTATTTAATATTTGCAAAATTTTGTTTGAAAAATCTAAATTTTTATATTAATTATTAATTAATTAATAATCAATTTAATAAACANATTTAAAAAAAAAAAATAATGTCGATATAAAATATAAAATATTTTACAGATCTCAGTGATGTACTTCAATTTCTTTNGAGATACAAATATATTTTAATTTTTATNTATTNAAAGTTNCTAAGCTCTAATTTAACTTCTTACTTAAATCTTCATATCGATCTCGGGTAAAATTATTATTTCTTTAAAATAATTACTAAATTTTTGATTTTAACCTCAGGTAAAAATAGTGTTTCAATNTTAATTTGTTTGAATTATTTATTAATTTTTATNTTATTTTTAAATATTTCTTTATTCAATNTTTATTTTNTAATTTTTTAAAGTGATATTGGTGAAATTGGGCGCATAAGATTAGGATCTGGNTTAGCAAAATGCTCTAATCTCTAAACTTTGANTCTTGACCTNAGGTAAAAATAGTGTTTNAATATTAATATGTATTTTTGAATAAATAATGATTTTTATGAGCAACTGACTAATTTAGNTTGGTTTACTTGATATTTGTTTGGCTTTTCATTNATTTTTATTTGTTGTTTTCAGATTTAATTCAAATTTTTATGTTTTTCTCATTCTTTTAAATATTTCTTTATTCAATATTTATTTTTATNATTTTTTAAAAGTGAACAATAATATTGGTGAAATGGGCGCATAAGGATTAGGATCNGAGTTAGCAAAATGCTCTAATCTCTAAACTTTGACTCTTNACCTCAGGTAAAAATAGTGTTTNAATATTAATTATGCATTTTTGAATAAATAAAGANTTTAATTTATGAGCNACTGACTAATTTGGGTTGGTTTACTTGATATTTATTTGTTAATTATTTTTATTTGTTNTTTTCAGATTTAATTCAAATTTTTATNTTCATTTTTNAATATTTCTTTATTCATATTTATTTTTATAATTTTTTAAAAGTNACAATAATATTGGTGAAATGGGTNCATAAGGATTAGGATCTGGNTTAGCAAAATGCTCTAATCTCTAAACTTTGACTCTTNACCTNAAGTAAAAATAGTGTTTCAATATTAATTATGCATTTTTAAATAAAAATTTANTAATGAACAAATTTGAATTNATTAAATTNATTTTTTTTGATTTATTCATTATTTTTATTACTTTTTAAATCAAATTTTATATTTTTCTCATTCTTTTAAATATTTCTTNATTCAATATTTAATTTT

>T.elliotti_rnd-3_family-66#LRR_CRS2

GAAATATTTAGAAGAATGAGAAAGACNTANAAATTTGAATTAAATNTGGGAAAAACAAATAAAAATTAATTGATGAATGAGCGAGCCGAACAAATATCAAGTAAACCAACCAACTCAATTAGTCAGCTACGTGTCACTCATAAATTTAAATCACTTTTATTGATTCCAAAACCATAATTAATATTAAANCACTATTTTTACTCAATGGTCAAGAGTCAAAGTTTAGAGATTAGAGCATTTTGCTAACTCAGATCCTAATCCTTATGCNCCCATTTCACCAATNTTATTCTCACTTTTAAAAAATAAAAAATGAAATATTGAAAAGAATGAGAAAAACATAAAAATTTGAATTAAATCTGAAAANAACAAATAAAAATTAATGAATGAACGAGCCAAACAAATATCAAGTAAACCAACCCAAATTAGTATCAGTCAGTCGCTCATAAATTAAAGTCTTTATTTATTCATTCAAAAATGCATAATTAATATTGAAACACTATTTTTACTTNAGGTNAAGAGTCAAAGTTTAGAGATTAGAGCANTTTGCTAANCCAGATCCTAATCCTTANGCACCCGTTTCACCAATTTTATTTTACCTAAAAAATAAAAAAAATAAATATTNAAAAGAATGAGAAAAACATAAAAATTTATAAAAACGAATAATGAACGGCCAAATAAATATCAAGTAAACCAAGTCGTGCTCATAAATTAAATNAATTATAGTNATTANAAANCATAATTAAAATGGNATTATTTTTAAGCTAAGAATAAAACTNTAGAGATTNGATTATTTTAAAGAAAAATNAATAANTTTACCCGAAATCGATATNAAGATTTNAGTGAGANGTTAAATCAGAGCTTAGTAACTTTTAATATAGTATAATTAAAATATATTTANATCTCGAAAGAAATTGAAGTACATCANTGAGATCTGTAAAATATTTTATATTTTATATCAACATTTATTTNTTTTTCTTTAAATTTTAAATTAATGAATTAATTTATTAANAAATAATATGAAAAAAATTAGATGAANTTTTTTNAAACANAATTTTGTAAAAATAAAAAAAATTGAATTAATCTGATTTTANTANTAAACATTTANATGAAANGGAAATATTTTGGTGGTTAAATAAAAATTAATTCAAAAAGTTTTTTATAAATCTGATAATACATTTAAGTATATGAGGCTAAACCATATTTATTTAAAAAAAAAAAAAAAAAAAAAAAAAAAAAAAAAAAAAAAAAAAAAAAAAAAAAAAAAAAAAAAAAAAAAAAAAAAAAAAAAAAAAAAAAAAAAAAAAAAAAAAAAAAAAAAAAAAAAAAAAAAAAAAAAAAAAAAAAAAAAAAAAAAAAAAAAAAAAAAAAAAAAAAAAAAAAAAAAAAAAAAAAAAAAAAAAAAAAAAAAAAAAAAAAAAAAAAAAAAAAAAATAAAACACCAATAGTAAAAAATAAAAAATATAAAAATTAAATAAAAGTTAANATAGAAANAAANTANTTTCGCTTATTAGNAAAATTAATAAATTAATCTATTTTAAATTAAATAAAAATATTATTTAATTTGATTAAATTAAGACAAAAATTTATTTATTAAGAAGATAGTAATAAGGAGTTTATNACAAAAGACAATATTATNTATTTATAATTTNAAAATAATTAAAATCAATCATTNATCTCTAATAATCATTAANATGTNAATATATAATTTACTAATCTTTTTGCTTTTATAAGTTTTGGTCTTAATCTTATTTTCAATNTTATTTCGCTTTTAAAAAATTGAAAAAATAAATATTGAATAAAGAAATATTGAAAAGAATGAGAAAAACATAAAAATTTGAATTAAATCTNAAAANAACAAATAAATGAATGAGCAAGCCGAATAAATATCAAGTAAACCAACC

>T.elliotti_rnd-5_family-1080#LRR_CRS3

AAAAAAAAAAAAAAAAAAAAAAAAAAAAAAAAAAAAAAAAAAAAAAAAAAAAAAAAAAAAAAAAAAAAAAAAAAAAAAAAAAAAAAAAAAAAAAAAAAAAAAAAAAAAAAAAAAAAAAAAAAAAAAAAAAAAAAAAAAAAAAAAAAAAAAAAAAAAAAAAAAAAAAAAAAAAAAAAAAAAAAAAAAAAAAAAAAAAAAAAAAAAAAAAAAAAAAAAAAAAAAAAAAAAAAAAAAAAAAAAAAAAAAAAAAAAAAAAAAAAAAAAAAAAAAAAAAAAAAAAAAAAAAAAAAAAAAAAAAAAAAAAAAAAAAAAAAAAAAAAAAAAAAAAAAAAAAAAAAAAAAAAAAAAAAAAAAAAAAAAAAAAAAAAAAAAAAAAAAAAAAAAAAAAAAAAAAAAAAAAAAAAAAAAAAAAAAAAAAAAAAAAAAAAAAAAAAAAAAAAAAAAAAAAAAAAAAAAAAAAAAAAAAAAAAAAAAAAAAAAAAAAAAAAAAAAAAAAAAAAAAAAAAAAAAAAAAAAAAAAAAAAAAAAAAAAAAAAAAAAAAAAAAAAAAAAAAAAAAAAAAAAAAAAAAAAAAAAAAAAAAAAAAAAAAAAAAAAAAAAAAAAAAAAAAAAAAAAAAAAAAAAAAAAAAAAAAAAAAAAAAAAAAAAAAAAAAAAAAAAAAAAAAAAAAAAAAAAAAAAAAAAAAAAAAAAAAAAAAAAAAAAAAAAAAAAAAAAAAAAAAAAAAAAAAAAAAAAAAAAAAAAAAAAAAAAAAAAAAAAAAAAAAAAAAAAAAAAAAAAAAAAAAAAAAAAAAAAAAAAAAAAAAAAAAAAAAAAAAAAAAAAAAAAAAAAAAAAAAAAAAAAAAAAAAAAAAAAAAAAAAAAAAAAAAAAAAAAAAAAAAAAAAAAAAAAAAAAAAAAAAAAAAAAAAAAAAAAAAAAAAAAAAAAAAAAAAAAAAAAAAAAAAAAAAAAAAAAAAAAAAAAAAAAAAAAAAAAAAAAAAAAAAAAAAAAAAAAAAAAAAAAAAAAAAAAAAAAAAAAAAAAAAAAAAAAAAAAAAAAAAAAAAAAAAAAAAAAAAAAAAAAAAAAAAAAAAAAAAAAAAAAAAAAAAAAAAAAAAAAAAAAAAAAAAAAAAAAAAAAAAAAAAAAAAAAAAAAAAAAAAAAAAAAAAAAAAAAAAAAAAAAAAAAAAAAAAAAAAAAAAAAAAAAAAAAAAAAAAAAAAAAAAAAAAAAAAAAAAAAAAAAAAAAAAAAAAAAAAAAAAAAAAAAAAAAAAAAAAAAAAAAAAAAAAAAAAAAAAAAAAAAAAAAAAAAAAAAAAAAAAAAAAAAAAAAAAAAAAAAAAAAAAAAAAAAAAAAAAAAAAAAAAAAAAAAAAAAAAAAAAAAAAAAAAAAAAAAAAAAAAAAAAAAAAAAAAAAAAAAAAAAAAAAAAAAAAAAAAAAAAAAAAAAAAAAAAAAAAAAAAAAAAAAAAAAAAAAAAAAAAAAAAAAAAAAAAAAAAAAAAAAAAAAAAAAAAAAAAAAAAAAAAAAAAAAAAAAAAAAAAAAAAAAAAAAAAAAAAAAAAAAAAAAAAAAAAAAAAAAAAAAAAAAAAAAAAAAAAAAAAAAAAAAAAAAAAAAAAAAAAAAAAAAAAAAAAAAAAAAAAAAAAAAAAAAAAAAAAAAAAAAAAAAAAAAAAAAAAAAAAAAAAAAAAAAAAAAAAAAAAAAAAAAAAAAAAAAAAAAAAAAAAAAAAAAAAAAAAAAAAAAAAAAAAAAAAAAAAAAAAAAAAAAAAAAAAAAAAAAAAAAAAAAAAAAAAAAAAAAAAAAAAAAAAAAAAAAAAAAAAAAAAAAAAAAAAAAAAAAAAAAAAAAAAAAAAAAAAAAAAAAAAAAAAAAAAAAAAAAAAAAAAAAAAAAAAAAAAAAAAAAAAAAAAAAAAAAAAAAAAAAAAAAAAAAAAAAAAAAAAAAAAAAAAAAAAAAAAAAAAAAAAAAAAAAAAAAAAAAAAAAAAAAAAAAAAAAAAAAAAAAAAAAAAAAAAAAAAAAAAAAAAAAAAAAAAAAAAAAAAAAAAAAAAAAAAAAAAAAAAAAAAAAAAAAAAAAAAAAAAAAAAAAAAAAAAAAAAAAAAAAAAAAAAAAAAAAAAAAAAAAAAAAAAAAAAAAAAAAAAAAAAAAAAAAAAAAAAAAAAAAAAAAAAAAAAAAAAAAAAAAAAAAAAAAAAAAAAAAAAAAAAAAAAAAAAAAAAAAAAAAAAAAAAAAAAAAAAAAAAAAAAAAAAAAAAAAAAAAAAAAAAAAAAAAAAAAAAAAAAAAAAAAAAAAAAAAAAAAAAAAAAAAAAAAAAAAAAAAAAAAAAAAAAAAAAAAAAAAAAAAAAAAAAAAAAAAAAAAAAAAAAAAAAAAAAAAAAAAAAAAAAAAAAAAAAAAAAAAAAAAAAAAAAAAAAAAAAAAAAAAAAAAAAAAAAAAAAAAAAAAAAAAAAAAAAAAAAAAAAAAAAAAAAAAAAAAAAAAAAAAAAAAAAAAAAAAAAAAAAAAAAAAAAAAAAAAAAAAAAAAAAAAAAAAAAAAAAAAAAAAAAAAAAAAAAAAAAAAAAAAAAAAAAAAAAAAAAAAAAAAAAAAAAAAAAAAAAAAAAAAAAAAAAAAAAAAAAAAAAAAAAAAAAAAAAAAAAAAAAAAAAAAAAAAAAAAAAAAAAAAAAAAAAAAAAAAAAAAAAAAAAAAAAAAAAAAAAAAAAAAAAAAAAAAAAAAAAAAAAAAAAAAAAAAAAAAAAAAAAAAAAAAAAAAAAAAAAAAAAAAAAAAAAAAAAAAAAAAAAAAAAAAAAAAAAAAAAAAAAAAAAAAAAAAAAAAAAAAAAAAAAAAAAAAAAAAAAAAAAAAAAAAAAAAAAAAAAAAAAAAAAAAAAAAAAAAAAAAAAAAAAAAAAAAAAAAAAAAAAAAAAAAAAAAAAAAAAAAAAAAAAATTGAAACACTATGTATAAATATTTTTGAATAAATATTAAATTANCAAAAAATAAGATATTCTAAATAGGATTTTTCTANTTCAAATTCTGATTAATCNTATTNNAAATCANTGAATAGCGATGATTTGGATTATTTGATTNAAAGCATTTATGAAATGAGATAATCGATATTGATTNAAATTTGAAAAAANTTAATTTCAAATTTTTCTTATTTTTATTAATTAATATTTAATATCTTAAAAAATTTTAAAAGAAATAAATGTTGATATAAAATATAAAATATTTTANCGATCTAAAAATGTACTTCAATTTCTTTCGAGATATAAATATATTTTAATNTTTATNTATTNAAAGTTANTAAGCTCTAATTTAACTTCTTACTTAAATCTTAATATTGATTTCGGTAAAATTATATTAACTTCTTTAAAATGATCGAATCTCTAAANTTTGACTTTTAACTTTANGTAAAAATAGTGTTTAATATTAATTACGCTTTTTAATAAATTTAATTTATGATAACTGACTATAATTTGGGTTTTTACTTGATATTTGTTTGGCTCGCTCATTCATTAATTTTTATTTGTTGTTTTCAATAATTCAAATTTTTATGTTTTTCTCATTTTTTNAAATATTTCTTTATTCAATATTTATTTTATAATTTTTAAAAGTNAAAATGANATTGGTGAAAAGAGGTCATAAGGATTAGGATCTGAGTTAGCAAAATGCTCTAATCTCTAAACTTTGANTNTTGATCTTGGGTAAAAACGATGTTTNAATATGAATTAGTTATATTTTGGGATGAATAAAAATGACTTTAATTTGTGAGCNATTAGCTGACTGGCTNATTTGGGNTGNTTNACTTTTGAATTTGGTTNGGCTCTCTTGCTCATTCATTAACTNTTANTTGTTGTTTNCANTNTTTNATTNAAACTTTTATGTTTTTCTNATNCTTTNNAATATTTCTTTATTCAATANTTNTTTNTATACACTTTTTAAAAGNGGGTGTAAAGTTAGTAAATTGGCGAAAAGGAAGTTTAAAAATTAGTATCTGATTTAGNAAAATGCTCAAATCTCTAAACTTTGACTCTAAATCTCGGGTAAAAATGTGTTTCAATNTATTTCGGAATGAACAAAAAAAAATNCTTTATTTTATGAGCGATACNCTTACAAACGNGCTGATTTGGTGNTGGTTTACTCNAATTTTNTTTGNCTCNCTAACTAACTTACTTACTCGTCCATCAATTTTTATTTGTTGCTCTCATGTTGATTTTAGTTTTTTATTTTATGNTTTTCTGTTNNTTTTAAATATTTCTTTATTTAATATTTATTTTTATTATTTTTTAAAAGCGNNAATAAATTGGTGAAATNGGNGCATAAGGANTAGGATCTGGNTTAGCAAAATGCTCTAATCTCTAAANTTTGACTCTTAATCTCGAGTAAAAATATTGTTTCAATATTATAATTATGCATTTTGGAATGAATAATAAGATATAAAATTTAATTTANGATCGACTGANTAACTAATTTTTGTTGGTTTACTTGAATTTTGTTTAGCTCGCTTATTCATTAATTTTTATTTGTTATTTCGGATTGAATTNAAATTTTAATGTTTTCTCATTCTGTTAAATATTTCTTTTACAATATTTATTTTAATAATTCTTTAAAAGTNANAATAATATTGGTGAAATAGGTNCATAAGAATTAGGATCTGAGTTAGCAAAATGCTCTAATCTCTAAACTTTGACTCTTAACCTTAGGTAAAAATANTGTTTCAATATTAATTATGCATTTTGAAGATGGATGAATAAAGAATTTAATTTATGAGCACTGANTACTTTTTGGTTTATTTAGTTTGATTTCTCATTCATTATTNTTATTTGTTATTTTCAGATTTAATTCAAATTTTTANATTTTTTCTTTCTTCGATATTTTTTTATTNAATATTTATTTTTATAATTTTTAAAAGTTNNAATAANATTGGTGAAGNTGGTGCNTAAGGATTAGGATCTGANTTAGCAAAATGCTCTAATCTCTAAACTTTGACCCTTGATCTAGGGTGAAAATAGTGTTTCAATATTAATTACGATTTTTGGAATCAACAAAGNACTTTAATTTACGATTAATTACAATTGGCTGACTAATTTTNGTTGTTTTACTTGATGTTTATTAACACTCGTTTATAAATTTTTATTCGTTNTTTTCATATTTAATTCTAATTACTTTAATTTTTTAAAAGGTGTAATAATATTNGTAAAGTTGGTGCNTAAGGATTAGGATCTGAGTTAGCAAAATGCTCNAATCTCTCAGCTTTGACTCTTGACCTTTNGTAAAAATANTNTTTCAATATTNATTATACATTTTAATGAATAAAGATTTTAATTTACGAAAACTGNTTGATTGATAATTTGGTCGGTTTACTTGATATTTGTTTAGTTTTCATTCATTAATTTTTATTTTTTATTTTCAGATTTNATTCAAATTTTTATGTTTTTTTAAATATTTNTTTATTCAATATTTACTATAATTTTTTAAAAGTAGNAATAATATTGGTGATAGGNGCNTAAGAATTAGGATCTGAGTTAGCAAAATGCTCTAATCTCTAAACTTTGAANCTTNANCTNACGTAAAAATANTTTTCAATATTAATTANATATTTNAAATTATAAAGACTTTAATTTATGAGTAGTTCATTCTAATTTTTTTGCTTNTTGATTTTAGTTTTGTCGCTNATTCATTAATTTTTATTTATTTTTTTAGATTAAATTCTAATTTTTATGCTTTTCTCATTTTTAATATTTCTTTATTTAACATTTATTTTTATAATTTTTAAAAACTGGTNCAATACGCNTTAGTAAAATGTAGGAATTAGNAAGAATAAGNTCCAAACTCTAAGTCTTTGAAATCAAATTGAGNTTAGGTTACTACAAAAGTAGCATGATAACTTTTAATAANATTTCGACTGATAATTATGCACTTTAAAATAAACAAAGAATTTATGAAAANTAAGTCATACAAATNTGGGTTTACGACATATCGGTTAAAATATTAGTTGTGGCTATNTTCTTGCAATCACTGTTANTTCAAATTTGTTAACNTNTCTCAATNNTTTCTNTGTCAATTTANTTGTTTCTTTTTTTATAATTTANTTTTAATAAATAAGTTTTGAATAAAAGGNAAAANANTTTCCTTGAAATGAGTGAAAAAAGATTGCTGTTTAAAACTTTAAAAAGCAAAAAGNTTAGTTAAACTTTCAATAGAATGTTGATNATGTTATTNTCTAAAAACGATATTACATACATCATTTAAGTTTTTTNCTTGTAATTTATTTCTACTTGTTTTGGANNAAANGAATACNTGTCATNTTGTTGATTAAATTTTTAATTCAAAAAGAACTTTNTGACATAAAACTTACTTTTGACTNCTGAAATTCAGAATTNAAANTTTTACATCTTTTTTAGATTTGAAAAACCAGATTTGAGATATATTTTATCTNGAATTATTTTTCTAAAGTTTTGTTTGTAATNTTTAAAANCATTTAATTTATCTTATTGTCAATGNTTTT

>T.elliotti_rnd-5_family-24#LRR_CRS4

TTTTAAATAAAATAATAAATTGTTAAAATAATGAATAAATNAAAGTTACCTNAGGTNAAANNNTAAATTTGTGAGTTGAGTGCANTTACTTAANCCTAANCCNATANCTGATGCTCCTTCATCCCCAATTTAATTTNAACTTTTATTTTNGAAAATAAAATTAAATAAAAACNATCATTATAAATAAAGGTTACTTCATGTNAAATTCTAAATTTGTAAGTTGAGTGCANTTACTTAAGCCTAGTCCTATATCTGATGCTCCTTCATTCCCAATTTAATTTTCGCTTTTATTTTAGAAAATAATGTTAAATATAAATTTTTCAAAAATAATAAATAAAAATAGTTACCNCANATAAAATTCTAAATTTGTAAGTTGAGTGCATTTACTTAAGCCTAATCCTATATCTGATGCTCCTTCATCCCCAATTTAATTNTCGCTTTTATTTTAGAAAATCATATTAAATAAAATTAAAATAAATNTTTTAGGTATTTTTTTATTTTGATTTAAATAAAAATTATGATCAAATAATTANAAAAAATAAAAATTAAATTACTTAATATCAAATTAGGTTAATAAAAAGAGTATTTTATCTCAAAAAAAAGAANNAATAAAATATTTTTTAATATTAATAAATAAGCAAATACATAAAAAAAAACTTTAAATGATTTTATTNTCATATTAGATAAAGATTTTCATAATTTATTNAATTTTTTTACTTTAACTNTAGGTCGATGCTGGTAAGAGNTTTCAAATTTGATTTAATAAACTTTTAATTTAAAAAAAAGTTAGTTTAACAATAAATTAGATAAAAAATAAGTAATACATCCTATAGATTGTAAAATGTTTTACTCTCTTTCTCCATTTTTTTAATCTTTCCTTTCTTTTTTTCTTATTTCCCTTTACTATTTTTANTTAATTAATTTTAAACTAATCAGAAATTAATATCTTTAATTTTTAGAAGATTTTGGGGTAATCCTATTTTTAGAAACATTAATTAATTAGTNTAATAATAAAATAAGAATTTNNTTCANTGATAGATTTTAATTTNAATAGCCTACTTATTTATTTATACTATAAATATNAATAGTTATTTTATNAATAAATATACTTATCGCTTTACTAACTTATTTATTAATTAATAAATGAATTAATTCATTTAATTAATAAAAAANTTATTCTAAATAATAAATTGAANCCTAGATTAAATTATTTGATGTATNTTNATGTTTATCTTAAAACTTTTTTAAAGTNAAATTTNTAANATTTTAAATATATTAAATAATTTTTCTATATTNNATAAAGAAGAAATATTTTAAATTAAATATTTTTTAAAATTGTAATTTANATCATTTANATTATTTATAATAAATTAAATAATACGAANCAATATATTACCATTTATCATCTTAAAGNTTTTTTGGAGATTAATTTAAACATTTTTGAAGTTTATTTAAATTATATTTCTATTTCATTTTATTGTGGTAAAACTTNATAAATGCTTTTATNAAATTTATCAATTTTTAAGTAAACAATTTTAAAAGATAGATACTCACACAAATTTAATATTGAATATTATTTAANTAAATTATACATANACTTATGAATANAATTTTCATTATTCATTAATATTAATTTCAATTTATGTTTAGATTAACTAATCTTTTTATCTTCTATGCTAATCTTTTGTTCTTTGCTTTTTAGTCTTNTCTTATTTTTTAAATTAAAAAATATTAGATTAAATAAATTAAAAATTAGCTTATCTAAATTAAATTTAGATTAATTAATTACTCTACATAAAGATTTAATATCTTTATATTTCTACAGTTTATTATTTCTCTCGATTTTAGAAATTTTTCATAGTTTCTATTATATTTATTTTGTTAATTTATTAAGAGATTTGTTTAAAAATTTATTTTTACNTAAAGCCTAATGTTAAAGTTTTTAGTGAATCGATATTAGAAATATGTTGAGCCATACTACAATCTTCATCTCCAATTTAATTATTGCTTATATTTTAGAAAATCANGTTTAAATAAAATNAATGAAAATTTATAAATTNTTAGNTATTTTAANGTTACNTCATGTNAAATTCTAAATTTGTAAGTTGAGTGCANTTACTTAAGCCTAGTNCTATATCTGATGCTCCTTCATTCCCAATTTAATTNTCNCTTTTATTTAGAAAATAATTTTAAATAAAAAATATTATTAAATTTTTNTAAAATTAAAAAATAAAAGTT

>T.elliotti_rnd-5_family-275#LRR_CRS5

AAAAAAAAATTATAACTTAATTGGNNATCNGGGTTAGCTNAATNNTCTAATCTATAAAGTATAGGNCTCGGTCTCAAAAATAACAAAATTGGTGATCAGGGTACATCAGAATTGGGTTTTAGATTAGCATAATGCTNTAATCTNAAAANTNTANTTCTCGATCTNGGNGGNAACTAAATTGGTAATAAAGGTACATCAGAATTGGGCTCANNATTAGCTAAATGCTCTAATCTATAAAGTATATANCTATGTCTAAGTAATAACTAAATTGGTGATNAGGGTACATCANAATTNAGCTCNGGATTAGGTAAATGCTCTAATCTATAAAGTATAAAACTAGACCTAGACAATAACTAAATTGGGATTAGGGTGCATCGNAATTGGGCTCTGGATTAGCTAAATGCTCTAATCTNTAAAATATANTACTATNTCTNAGNGANAACTAAATTGGTGAATAGGGTGTNTCAGAATTAGGCTCTGGATTAGCTAAATGCTCTAATCTATAAAGTATAATTCTATATCTNTAGAATAACTAAATTGGCGATAAGGGTACGTNAGAGTTGTGTTCTGGATTAGCTAAATGCTCTAATCTATAAAATATAGTTCTANATCTAAACAATAATAATTTAGGCAATAAGGTTTTATCAGAAATGGGNTCTATATTTTCTTAATGCACTAATCTNTTAAATNTTAAATTATTTTTAAATTAGAAAGAATATAATCATGATTTGAAAAAGTTCTCTNATCTCTAACCGTCGAAAAACCTAAGTATTAGCTTTTCTGANTTTTAACAAATGGATGCTTTCTTTTTAAGTTANGCNTTAATTAAGTTTTCTTTTACATAGACTTTGATACTNAATCTAAATNAAAATAGTTTGGATGAGATGGCTAAATCGTTTTTAATGAAAAAAATTTAGAAAATGAAAAGGTTAGTTGTTTTTNAAAAAATTAATTGGTGATTAANATTGAAAAATATNAAATAAATATTAAAAATTTTATATATTATAAATTTANGTAACTAANTAACAANTGAATTTTGTAAAATTCATTAATATATTTTANATGATTAATTGTAAGATATTCGAAAAATTTTAAATAATTTNTTATTATTACTCATAATTTAAAAAATTNTTAAAATTAATTTTATTTTTTATGAAAATTCAATNAAAATAAATTTTTACTTTTTTTAAATACTATAAATAATTTTTATATTTACTTTTGAAATATTTATTTGTCTAAATTTAATTTATTTTTAATTAAATATTTATTAATTTTATTAAAAATAATAANAAAAAAGAATAATGAATATTTTAGAATTCAAATTAATAGTGCAATTTATCAGATTTTTTNAGCACTAATTTATCATAGNATTTGAACATAAATATTCAAATTAGTAAAAGNAAGATTCAAATAAAGCATNATAATTGTTCGGAATCTATTGACAAATAGCTTAAATATTCTTATCTCTACATTTTGAATCTTTATNCCAAAAAAAATTTACTTGGTGATNAGGGACTATCAGAATTATCTTCAAGATTANAGAAATGTACTCATTTTTAGAGTATGAAGTTATATTTNAGAGAAAACTAAATTGGNGATTAGGGTACATCAGAATTGGGCTCTGNATTAGCNAAATGCTCTAATCTATAAAGTNTAGATCTATATCTCNAAAATAACTAAATTGGNGATAAAGGTGCATCAGAATTGGGCTCTGGATTAGCNAAATGCTCTAATCTATAAAGTATAAANCTTAATCTCAGAAATAACTAAATTGGTGATAAGGGTACATCNGANTTGGGCTCTGGATTAGCTNAATGCTCTAATCTCTAAANTATANAATTNAATCTCGGNAATAATTAAATTGGCGATAAGGGTATATCGGAATTGGGTTCTGGATTAGCTAAATGCTCTAACCTATAAAATATAAACTANNTCTAAGNAATAACTAAATTGGTGATAAGGGTGTATTNGANTTAAGTTCTGNNTTAGCTAAATGCTCTAATCTCTAAANTTTAAAATTANACCTCGAGTAATAATTAANTTTGTNGAGGGGACATCAGAATCTGGGTTCNGGATTAGCTAAGCTTGGAAATGCTCTAATNTNTAAAATATNNTNCCAGGTCTNNANAATAACTAAATTGGCGATAAGGGTGCATTAGAGTTGGGATC

>T.pyriformis_rnd-4_family-115#LRR_CRS1

TATNTATTTCTTTGATTTACAGAACCAAATNTTAGAGAAGTCNTAGCAGATCAGTNCTATCTAATCANTTAAAATNGNTGAGAATCAGAAATAGNAATCACTTAATATCTCTCTCATAAACGTAAGCATTNATCANCGCTTATTCAGCATNATTAATCGATTGTAAATAATTAAAATCAGGGNTCAACTTAACGTGTNCTGGAACAGTTTGAAAATCTAGAGTGNAAACCGATNAGTGTGGANGATTGTAGTAAAATACTTTCAACACTTCTGAATATCTCCACGTTTGAGAAATGGTTTGAGCTGATAGGTGTTGGAGCTGCNGGATTAGTNTTAGGTGCNAACAGTTTATCCTAAAGTAGGTCGGTTGTTTTAAAAGTGTAGAAAGTCAATAGCAAATCTNNGGTAGCTCAGGAAGTANNAATAATGAGAGCGTGTCAAATGCCATNAGTGGTNAAGTTTTATGGATACTTTTACTTAGACATTAACAGCAAGGATGATTNTGTGTGCTACGAATTAGAAAAATGTTCATGTATCCATNTTTATTACGAGCCTAGTAATTTCAACTTTAANATACCNTCNNTCCTCAGATTTTTGACTAAAANAACACCTAGATTCAATTGAACTAATTTAAAAGACCTTATTTTGCTCTGTTTTTTTCTCAACTTTTNAAAAAATTGTTCGNCAATATCTTTGACCCTTAATTTATGAGGATTGATAGTATATGAAAATTTNNCNTTTNAACAAATNATAGGTAATCTTGAGCAGTACCTNGAGAGATAACNAAAAGATAATGAATTCAGTGATGAAGTTAAGATGAATATTGCTGTTTAAATGGTGGATGCGATTAACTTTCTTCACTATCACAATATTCTTCACAGAGATCTCAAACTATTAAATTTCTTAGTTGTTGAAAACGATTCNAAATTCCCTNNGATCAAGCTCAGTATATNTTTCCTGANGTATTTTANGATATTCAANATTTTTGAANATGTATTCAAAGACGATTTCGATCAATCTGTTANACTCAAAAGAANAAATCTANNTAGGATTNTGAATTCNNAGATGAGATAGGCGATGTAAAATANTTGNNTNTAAGCACTTTCTCAANNNTAAATAATAGCAAGCGATTTAATACTGAAAAAGTTTGGAACTCCTTTNTTTTTGGCTCCCGAGTATNTGACAACAAGAAAATGCACAAAGGAAGTATGAATTATTCTTTNATCTTAATTTAATTTNGTTTGAGTTAATAAAAACAAAACATAAATTNATAAATATANAGAATGACNTGTTTGTATTAGGGATTTGCCTGTCTNTGGTNGACAATTATCCTAAGTTGAGCAANGATTNTATGAAACGCATAGAAATAATAAATGAATTTGAGATTCCTTACTNCAAAGAAGGAAAATAAGATATCATNAATAGGAAGAGTGATCTNTACTAAATCTTTGTTNAAAATACCCTGGTTTACAATGAAAAATAGAGAGNGNAGCTGAGTANTCTTTTAGAGAGATCAAAATAAANATATTACTCAAAGAATTTCATGATAGATTGTAACGTAAATTGAACGAAAANTATGATNGATATATTATTTTATCGAAAGATTGACATTAAAACCCATATTTAATGATTTTGAGTCTCAATTTCTAGTAAAATANATTTATAACTTTTAATACAAANCTTGTTTCTTAATTAATCTATCTTTCCTTTTTTTATCTTATCTTCAATTTCTCTCTCATCCTCTCATTTTCACACAAATCCTNTTTTAATCTTATTTAATTTCAGAATTCATTCATTTCTTTATCATTTGAAAATAGNTNTAATCAGATAGGTGNNGATGGAGCTAAAGCCATTGGTGAAGGAATCGCTAAATCCTCTACTCTCTCAACTCTCACTCTCGATCTNTAGTAAAATAACTAACTTTCTTTATTAACGATGAATATATTTATTTATATNTTCTTTCCTTATTTTATCTAATTCTCAATCTCTCTCTCTTTCTCTCCCATCCTCTCATTTTCACACAAATNCTATTTTAATTTTATTTAATTTCAGAATTCATTCATTTCTTTATCATTTGAAAATAGGTATAATCAGATAGGTGCAGAAGGTGCTAAAGGCATTGGTGAAGGAATTGGCAAATCCTCTACTCTTTCAACTCTCACTCTCAATCTCNGGTAAAATAACTAACTTTACTTTTAATAATGAAAGTATTTGTTTGAGAGCTTGAATTGATCTCTTCTTTTCTTTTCTTATTTTATCTCACTCTCAATCTCTCTCTTTCTCTGTCGTACTCTCATTTTCGCGTNAATCTATTTTAAATTTATTTAATTTAGTATTCNTTCATTTCTCTTATAAATCTACTTCT

>T.pyriformis_rnd-4_family-309#LRR_CRS2

AAAAAAAAAAAAAAAAAGAAACTTGAATAATAGTTTNTAATTGGAAAACATTCTCAAGATGATCTCGTTTCTTAAATTACAANTTAAANCTTTTTGAATATTATTAATCATATCATCAGAAAATATATACCTTGATANAATATCATGGGAATGAAAANTGAACATTCTATTTTGTGTTGGGTGTAANATAGTTACAAATATGTATTTTGTAAATAACATCACTCTATTTCTATCACCATTAANTATTTCGNGTTTAACCATATCGAATACCTTTAATTAATATTTAAATCANCATATTTNCAAGNCAACTAGTCTTATTATCTTTTAGAATNAAGACCTAACATTAGTATTATAGTAACAGTATTGCTGCTTTTTAATAANAAAAATAAAAAAATAAAATTAGAATAAACGATATAAATTGGAATGAGTTATTACTATATATTAGAGATTGGGGGACTGACCAATCACTAAAAATAGCAATAATAATAAATAGTATTNTGATATTATTATTTGTTATTATTGCTATTTTTAGTGAATTGAGTTANGTCCCNCAATCTCTAATATANATTNGNTTTCGATTTTCGCCGAATTTCTTTGAATTTAATAATGAATATGAAGTGAATTTTATTTTACNTAAGGTTTAGAGTTAGAGAGGTGAGATTCGCGCAGTTTCTTAAGCCAGAACCCAGANCAGAAGCACCCTAANCACCAATTTGATTACTCCTTTTNATTAAGAAGAATAAATAAAATTAGNGATATAAACGATATAAATTTAATTTCAATTTTTATGAATTTCTTTGAATTTAATAAAGAATATAAATGAATTTTATTTTACNAAAGNTTTAGAGTNAGAGAGGTGAGATTCGCGCAGTTTCCTAAGCCAGAACCCAGANCAGAAGCACCTTAATCACCAATTTGATTGCANCTTTTTATTAAGAAGAATAAATAAATAAAATTAGAGATGTATAACGNNATAAATTTGATTTCAATTTTTNCTGAATTTCTTTGAATTTAATAAGAGAA

>T.pyriformis_rnd-4_family-335#LRR_CRS3

AAAAGCAGCAATACAATATTTTTTANTGTACTGANTATAACTTTATCATATTGGTTGTCATAATATCCCCNGCATATTTGAAAGACATNTAACTTTAATAATCATAGCAATTTAACTACTATGNTATGAAATNNGTATATTTGAGTTTTATTATTATTTTGAATAAAGTAATGTACATNTAAAACTCCAGAAATAAACGCTAACAAAAGATAATTTAATTAAAGCNAATTATTTACTTAATNATTACAANAAGAAGAAAAAAAAACAAATTTAANAAGAAAAAAGAATGTTATAATAGAAAAGCAAATACGACAANCTCTAAGATGTAAATGTTATTTTAGCAATCTTATCTCNATAAATATTTATTTTATCTGAAATATATTTAGTTTCTCTCCTCTGATCTTTCCACCCATCTTGANTTANATTTAGATCTCNAGTAATACGCGTTTTTATGAAAATTTAATTAATACCTTTCAGTTAGANCTTAACCGCTATTTATCGATTGATTTCTTCTAGATCTCTCAAAAAGTGTTTTGATTAATTTCGAGATATACTCGAAAATAATTCTTTTGCTTTCTTTTTAAANCTTCTTTAAAATAGAATTTAAAATAAAAGTTANAATGTTTTTAAATAGAATATCATTTTAAAAAAAAAAAAAAAAAAAAAAAAAAAAAAAAAAAAAAAAAAAAAAAAAAAAAAAAAAAAAAAAAAAAAAAAAAAAAAAAAAAAAAAAAAAAAAAAAAAAAAAAAAAAAAAAAAAAAAAACCGANAAATTGGCCTTATANACTATCTTTNTTCTCAAAAGTCGGCTTTTAAATAGAGTATCATTTTTATTATTCAATTCATANTCCTATCAAATCGATAGATTTAGCATTCGAATTATTAAACTCATTTACATATTTTCTGATAAAGTNACGATNANNTTGGNGAGACTGGAGTTNANAATCTTGGGTCTGCATTAAAAAANTGTGTGAATCTNAGTACTCTNACTCTTGACCTCGAGTAAAATATNATTTATCATTTTTTTTATTTTAATTAAAAAATAAATNGATTGATTTAACAAAANCTGATTTATATTTTTTTAATAAAGTATNACAATTGGTGATAATGGAGTTTCAAATCTTGGNTCTGCATTAGNAAANTGTGTGAATCTAAGTACTCTCACTCTTTGCCTCGAGTAAATATTTAATTTAGAATAAATAAATNGATTTATTTAGAAAAAAAATTGATTTATATTTATGTTNATAAAAGTNANAATNAAATTGGTGATGCTGGAGCTTCAGATATTGGNTCTGCATTAGCAAANTGTGTGAATCTAAGTACTCTNANTCTTNACCTCGAGTAAAATATTATTTTATTTTTATTTTTAATTTGATAAATTGATNTATTTACTTAATTATATTTTTAAAGNGANAATNAAATTGGTGATGCTGGAGCTTCAAATCTTGGNTCTGCATTAGCAAACTGTGTGAATCTAAGTACTCTNACTCTTAACCTAGTGTAATTATTCTTTTATTTTTGAATCAATAATNAATCTATTTAAATAAAAATCGATTATATTATTTGNTAATAAAGTNACAATNAAATTGGTGATGCTGGAGCTTCAGATNTTGGANCTGCATTAGCAAACTGTGTGAATCTNAGTACTCTCACTCTTNACCTCGAGTAAATAAATATTATTTCTATTTTAATTTTGA

>T.pyriformis_rnd-5_family-105#LRR_CRS4

AAATAATTATATGCATTTATTNANATATGACTNATTCATAAACTAATTAATCCACAAAGCAANACATCCAGCCNGAGTTTAATCTGAAAAAGAAAAAATATAAAGTAAAAAATTAAGAAAAGNAAGAGAAAACAAAGNAATGGGAAATTTTTGCTCAAAAAGAAAGTTATNATAGAATAAGAAGTNCACCGAAGCAATCCAAGTAGTTCTCGTTTTTCGAATGACCTCCNATTAAACTTTTATTATTTCNCCAACAACAAAAGTTATTGAAGCGCGATTCTTCTTCGCGTCTTNCTTTAGATATCGATCTAACGTAAAAATNGTTTATTAAATTATTTCTTTTAATTTCCATTACTTTTTAGCTCATTGATTATTTTTTTAAAANTTAAGGAAAAGTTCTATNGGTGCAATAAAAGAGAATAGAATCGATTATNAACAACCAAGGTCTATGTCGNCGCANATGGTGCTAAACGACTTGGCGCNGCAATAGGGAAATGCGTGAATCTCACTGNTCTCACTCTTGATCTTCAGTAAAATAATAATTATTTCTTCTTCGTGTTCCATTTTNAAAACAATGAAAGAATTAGAAAGCATTTTGTCAATAAAATATAGTTGTTTATTCTTAAATCTGTCACTCAATTTCTTTGATTTTTTTCAAAACTACCTGAGAAATACCTTTTAATTAATACTCTATACAGCTTCTAAATCTTTTAATTCAATCAGGTAAAATNATATCGAAGANGAAGGTGCTTAATGTCTTGGCGCTGGAATAGGAAAGTGTATTAATCTTAAATCTCTCACTCTGGATCTTGAGNATTATGATTGTAAAATAATGTTAATTTCTCTTTTATAATCTGTCTTAACCTAATGAAAGAATATAATGATATACGATNCACGAAAATACTCTACTAAATTGTTAATATCGTCATAATAATGACTNAATTTAATGCATTTTAATTTTTAAACATTCATCAAAATTTACATAATTATTATGTACTNAGTTTCTATGGTTTGAAGGATANTGNTGTTTCAGCTCTCTTCGCTGGATTAGCNAAATGCGTAAACCTNACTACTCTCAATCTTGACCTGANGTAAAATANAATACATTTCTTCTTCATTTTCGATTTTATAAAAATGAAAGAGTTTNATAGCAGAATTTTGACGAAAACAACAAACAACCAACAAGCCAACTNACAATTGATAAATTTCATTGTCTTCTCTTTTAAACCTGACTTNATTGAAGTTTTTTTTCCTTGAGCCTTTCTAAACAGAGTTTTAAATGTTTAATCGAACGAACTCATACTTATTCTTTTTTATTAAATCTAGAAACCATGGTCTGATATGGNAGGGGNNCCNNNAGNCCTNGNNNCTNNNTNGNNCGNNGNNTGNGTNNCNCCNNCCNCNNCCNGNNTCNNGNNCNCGTTAAATTAATCTCTCATCGTATTCTAATTTTGAAAATAATGTTAAATTACTCTTTCGTTATATTTTATTTTAACAATAATGACATAAATTTGGGAGTGAAAATGTCCAATTTTTAAGAAATTATTGATAAAACAA

>T.pyriformis_rnd-5_family-314#LRR_CRS5

AAAAAAAAAAAAAGCAAAAAAAAAAAGATGTAATTTTTACAAANAAGATGAAAAANTNATTGAGAATTTCAAAAACTCGTAGTCTAATTTGAGAAACTCGAGCTNAANAGANTTAANCCTGANCAAATAATTTTTCGATTAAGNATTGTCTCGCTCGAGCTCGGCTCGAGTTATCGAGCTTTTTGCCCAACAGTCCCTAGTAAGATCTAANTTAGTTATGTGAGATTCTTGCAGTTGGCNAATTCTTTACCTAAATTGGAAGCTCCGTCTTCTAAATTTNATTCCTAGATAAGTTAAAGATANTGTTGTGAGATTCTTGCANTTGGCCAATTCTTCGCCTAAATTTGAAGCACCTTCTTCACCTATAAATTTCTTTTCAGATTTAAATTNAGCGTTGTNAGATTNTNGCANTTNGCCAATTNTTTGCCTAAATTTTAAGCACCACATTTACTAATTTAATTNCTCTNCAGGTCTAAAGTTAGTCGTGTGAGATTCTTGCAGTTGGCCAATTCTTTGCCTAAATTCGAAGCACCATAATCACATATATTTTAATTATTAAGGTTTNAAGTTAGTTGTGTGAGATTCTTGCNGTTGGTCAATTCTTTGCCTAAATTTGAAACACNTTTNTCGCCAATTTAATTATCGTTTAGGTTTAAAGTTAGTTGTGTGAGATTCTCGCANTTNGCCAATTCTTTGCNTAAATTTGAAGCACCACCTTNACCAATTTAATTTTCTTTAAGATTTAAAGTTAACTGTGTGAGATTCTTGCANTTGGCCAATTCTTTNCCTAAATTTGAAGCACCTTCGTCGCCAATTTNATTATANCTAAGATTTAATTTTCCAAGGATGTGAGATTCTTGCANTTNGCCAATTATTTNCCTAAATTTGAAGCNCCATTTTTNCTAATNTAATTNTNNTNNAGGTTTAAAGNTAGTTGTGTGAGATTCTTGCAGTTGGCCAATTCTTTGCCTAAATTTGAAGCACCNNCGTCACCAATTTAATTANNATTAAGATTTAAAGTNAGCTGCGTGAGATTCTCGCAGTTAGCCAATCCTTTACCTANATTTGAGATANTATCACTATTTTAATCATTGCCTAAATTTAAATCTAGTGATGTGAGATTCTTGCAGTTAGCNAATTCTTTACCTAAATTTGAAGCACCTTCGTCNCTAATNTAATTTTCACTAAAATTTAAAGTTAGTTGTTTGAGATTCTCGTANTTAGCCAATTCTTTACCTAAATTTGAAGCTCCTTCTTTCCCTATTTGATTCNTCCCNAGGTTTAAAGTGAGCTCTTGAGATTCTTGCANTTTGCTAATNCTTCGCCTAATTCTGAAGCACCTTCATCACTAATATTATTTTCTCTAGATTTAAAGTTAACTGCTGTGAGATTCTTGCANTTGANNAATCTTCTNACTAAATTTGAGCCCTTTTCCNTCAACATNAAACCTTTTTTCGNAATCCTAAAATATCGNANCTTTNTTAAGGNCTAAAGTGAGCGNTGAGTGAGATTTTTGANNTTNNCCAANTNNTNGCCTAANCNTNNAGCACCATCATCACCACTTTTTTCATAACTAAGGCCCAATGCTAGTTGTGTTAGATTTCTACAGTTGGTCAAATATTTGCATAACTCTAAAAAACCTTA

>T.vorax_rnd-3_family-64#LRR_CRS1

AATCTCACAAGTTTGACTCTTGGACTAAGGTAATAATCTTTATTTGATTAATTTTTTTTTACCACATCTTATTCCTTTCAATCTAAATTTCTTTTTTGTTAATTATATTCTTTGAATAATCTTATAGGGAAAATTAGATAGGTGCAAAGGGTGCTTCTAAACTTGGAGCTGGACTAGGCTAGTGCTCTAATCTCACAAATTTGAGTCTTAACCTCTAGTAATAATCTCGATTAATTGTTTTTTTACAACATCTTACTCCTTTCGATCTAAAAGGTTTAATTCGATAGGTAGAGAGGGTGTTTCTGCACTTGGAGCTGGACTAGGCTAGTGCTCCAATCTCACAAGTTTAAATCTTTATCTTGGGTAATACTCTTTATTTAATTTTTATTTTTAAACTTTTATTAATTTTTTCTTTTTTCTCATCCTTCTTTTCCTTTCAATCTAAATTTCTTTATTGTTGATAATATTCTTTAACTAATCTAAAAGTNNNAATTCGATAGATGCAGAAGGAGCTNCTGAATTAGGAACNGGACTAGGAAANTGCACTAATCTCACNACTTTGANTCTTAACCTCNAGTAATAATCTTGATTAATTNTTTTTTTACAACATCTTACTCCTTTCAATCTAAAAGGTTTAATTNGATNGGTAGAGAGGGTGCTTCTGCACTNGGAGCTGGACTAGGCTAGTGCGCCAATCTCACAACTTTGAATCTCGACCTCGGGTAATAAATTACTCTTTATTTAATTTTTAATTTTTTTTTCACATCATCATTCTTATTCCTTTCAATCTAAATTTCTTATTTGTTGATAATATTCTTTGAATAATCTAAAAGGNNNAATTAGATAGATGNANAAGGTGCTTCTGCANTNGGAACTGGATTAGGCNAGTGCACCAATCTCACAACTTTGANTCTCGACCTCGGGTAATAATCNTTATTTAATTTTTTAATTTTTCTTTTTAAAATTTGATTAATTTTTTNTTTTTTCTCATCATTCTTATTCCTTTCAATCTAAATTTCTTTTTTGTTAATAATATTCTTTNAATAATCTAAAAGGTANAATTAGATAGATGNATAAGGTGCTTCTGGANTAGGAACTGGATTAGGNNAGTGCACCAATCTCACAANTTTGACTCTCGACCTTNNGTAATAAATATCTTTATTTAATTTTTGATTTTTTTTCANATNATTTTATTCCTTTCAATCTAAATTTCTTTTTTGTTAATAATATTCTTTGAATAATCTAAAAGGNACAATTAGATAGGTGCANAGGGTGCTTCTGCACTNGGAGCTGGACTAGGCAAGTGCGCCAATCTCACAAGTTTGACTCTCGANCTCGGGTAATAATCTTTATTTAATTAATTNTTTTTTTACAACATNTTATTCCTTTNAATAATCTAAAAGGTNTAATTAGATAGGTGCAGAGGGTGCTTCTGCACTTGGAGCTGGACTAGGCTAGTGCGCCAATCTCACAAGTTTGANTCTTNATCTCTGGTAATAATATTCTTTATTTAATTTTAATTAATTTTTTTTCACATCATCCTTATTCCTTTCAATCTAAATTTCTTNTTTGTTAATAATATTCTTTGAATAATCTAAAAGGTCGAATTAGATAGATGCAGAGGGTGCTTCTANACTTGGAGCTGGACTAGGCTAGTGCACCAATCTCACAAGTTTGGNTCTTNACCTCGGGTAATAATCTTTATTTAATTTTTGAATTTTTCTTTCAAATCTTCAAATCCTTCTTATTCCTTTCAATCTAAGTTTCTTTTTCGTTAATANTATTCTTTGAATAATCTAAAAGGTANAATTAGATAGATGCAGAGGGTGCTTCTACACTTGGAGCTGGACTAGGCTAGTGTNCCAATCTCACAAGTTTGAATCTTTATCTTNGTAATNNTCTTTATTTAATTTTTGAAATTATTTTCATTTTTTCATCTTATTATTTTCAATATTTTTGAAATTAAATATTTAATAGGAGTATTNGTGAGTTCAACGCATCTAGATTCAAAAAAATAATTAGGCTGACTAAATTGAAAATTNATTTCTGATTTGTTTAATTTTCTAAGTTTAATATTTTAAAAAATAAAAGAATAATTATACTATTTGTTTTTGTTTTGAATTTATAATTTATCTATTTATGTTATTGAATACACACCATTATTTTAAATTAAAATATTTAAAATATATACAATAGTGATTATTTCTTACTCTGGANTCGGGAAATNAAATATAATTTCCAAAAAACNAAACTATAAAATTTATAAACTTTAGTATATTTTCCGAAAAANATTTTTANCGAGATNAAAGCCGCTAAAATATAATGCATTCTCCAAGTAATAATTTGGTAACTCTTTACTCTTTTAATAAAATAAAAATTTANTTGCTCGGATTTTATTTTGANATAATCGACTCGGATTTTTATTACTCGATTTCGAACAAAAAAAAAAAAAAAAAAAAAAAAAAAAAAAAAAATAAANNAGATAAATATTAAAATTACNGGAGGTTGAGANTCAATGAATAGTGAGATTCGNGAAGTTTNCTAATCCATNNGCAGCTAGNNCAGAAACATGCCCTAGCTNCGNAGTATCGATTTATTCCTATNTTATANTTATTNAAA

>T.vorax_rnd-4_family-198#LRR_CRS2

AAAAAAAAAAAAAAAAAAAAATAAAAAATCTATGATTNAGTATAATTTTATTTATTAATTTTATTATGAATGTATATATTTATGTCTTCAAAAAACACACTAACAAAACTNATAACCATCAAATAAAAAATTAATTAATTGGATAACTAAAAAGATAATTAATAAGAAANTAATCATAACAAAAAAAGGAATGTAATAGAATAAGAAATATNATAANTTAGAAGACGTAAATGATATTATTATTAGATAAATACTTATTGAATTTTAATTTTATCATAAATAGTTTTTGTCATCTAACAAGACTGAACTCATATCATTGGAAATTTGTTTAAATTAACGTAACTTCATTTATTANTAAAAAAATAAGTATCGTAAAGTATAGGGATTTGCAAAAAGTTNTTTTATGGAAAAAAAATTTTAAGTGGATATTATGATTGTTAAANATTCGCGAAGGAAAAGTTTCGCGAACCTAAAAAATATTGCCTTCGTCCCAACCTCCGATATGGATTTATTAATAACTTTTCNTTTTAATTATAAAAGTTACGATANCATCGGGNNCGAAGGAGCCATTNGATTAGGGAGAGAAATTAAATATTNCGCAAATCTAAATTCTTTATCTNTTTAACTTAAGTTAAATNAATCTTATTATTTATTTTATTAAATTATTTAAAATTACTGCAATTTGAAAGTAATATTTGATATCTTAATTGAATTTATTAATTNTCTTTTTAATAAAAGTCGNAATAACATTGGGTCAGAAGGAGCCATTGGATTAGGNAGAGAAATTAGANATCGCACAAATCTCAATTCTTTGTCTATTTTACTTATAGTAAAATAATCTTATTAGTATTTTNTTAAATTATTAAATTAATTCTTGTTTCTTTTTAAAACTATTTCTGATTGCTCAATTTCAAATATGCAGTAATTTTCAATCCAATCTCAAAATAGATTTATTAATTTTTCTTTTTATTTAAAGTNGCTGTAACATTGGGCNAGAAGGAGCCNTTGGGTTAGGGANAGAANTTGGAAANTGCACAAATCTCANTTCTTTGAATCTTGCTCTTAAGTATAAATAATTTATTAATTNTTTTATTAAATTATTAAAATTTAATTAAATCTTGCTTCTTTATCAATTTTTGTTAATNTCAACTTAAATTGGAATATTTGGAGCCATTGAATTAGCANAGAATTCGNAAAAATGCATANTCTCAATTNTTTAACTCTTATACTCAGGATGCATATTTAAATAAATTTCTTTAACTGATATTGNCTTATTTATTTTTTGAATAATAGTAGGATATAGATTGGTCCAGAAGGAGCAATNGGNTTAGGGGGAGAACTGAGTAATTGCTCTAATCTCAACTTNTTGACTCTTNATCTTTAGTAAAATAATTCATTATTNTTTTATGAAGTTATTAAGTTAATTCTTGTCTTTTTGCTAAAAATTTTTTAATTTTTATTAGAATTTCTTCAATTNCACAAATCTCAATCNTTTGTCTCNCGTACTATTGTNAAATAATCTTANCAGTGTTGCCTCTTNAANTTATTGATAGCAAAAATTAATTTAATTCTTGCTATTTCCATTTATTGTCATTAATATTTATNTTTAATTAATTTGATTTATTTACNTTTCATTTTTTTATTAAAAGTTNNAATAACATTGGAGCAGAAGGAGCCATTGGATTAGGNAAAGAANTTAGAAANTGCACAAATCTCACTTCTTTGACTCTTAATCTAANGTAAAATTAAATAATTTGTTTTAAATTNTTAAAATTTANTTAACATTTTACTGTTATTCGAATGNTAATTTTCTATTTATCTCAATATGATTTATTAATATTTTTTAATTAAAGGTGCAATAACATTGGGTCAGAAGGAGCCATTGAACTAGGGAGAGAAATTAGATAATCGNTCAAANCTAATTCTTTAACTCTATTCACTGTAGGTTAAATAAATCTTACTAGTNACTATTAAGTTATTTAATTCAAATGCAATNATTCTCAATAATCGATTTATTATATTTTCTATTTNAATAAAAGTGGGAATAACATTGAGTCAGAAGGAGNCATTGAACTAGNNAGNTAAATTAAAAATTCAAAATCTCCATTCTTTGTNTCTNTTGCTATTGAGTAAAATGATCTTTATTAGTATTTTTTCCCATTAAATTATTNCAATTAATCTAAA

>T.vorax_rnd-5_family-772#LRR_CRS3

AAATTTCAAGATAAATNTTTAAAGGAANAATAAGAGATTGGTGCAGAAGGTGCNTCTGCAATNGGGATTGNATTAAGANANTGCGCAAATCTCACNTCNTTGACTCTCTATCTCGAGTAATAATCTATTATTTATTTTTTGAATTTTNTTNTAAAATTTAATTAATTTTTTNTTCCTTAACACCTTATTCCTTTCAATCAAATTTTCTCTTTTGCTAATCTTCCAATTCTCGAACTGAATGATTTAATAGTTATAATTCGATAGATTCAGAAGGTGTTTCTGCACTTGGAACTGGACTCAGCTAGTGCACAAATCTCACAAGTTTGACTCTTTACCTCAAGTAATAATCTTATTTAATTTTTGAATTTTCTTTTAAAAATTTAATTAATTTTTTCTCTTTTAACACCTAATTCCTTTCAATCAAATTTTCTTTTTTGTTAATCTTAATTTTTGAACTGAATAATTTAAAGNTATAATNNGATAGATGNAGAAGGTGCTTCTGCACTNGGAAATGCACTCGGAAATTGCGCAAATCTCACAANTTTGACTCTCAACCTTTAGTAATAATCTTTATTATTTTTCTTTAATTTCTTTTCAAAATCTTATTCCTTTAAATCAAACTTTCTTTTTTTCAATCATAATTTTTTAATTAAATAATCTAAAAGNNATAATTAGATAGATNGAGAAGGTGCTNCTGGACTAGGAANTGCATTNGGAAANTGCGCAAATCTCACANCTTTGACTCTTNACCTNNNGTAAATATTATATTAATTAATATTCNTTTTNTTAACATATTATTTCTTTCAATCAANTTTTCTTTTTTNTTAATCATAATTTTTTAACTGAATAATCTAAAAGTAATAATNAGATAGGCGCANAAGGTGCTTTTGCACTNGGAANTGGACTNAGCNAGTGCACNAATCTCACAAGTTTGACTCTCNGGCTTGGGTAATAACCTCGAGTNAATNTTTGATATTATTCTTTTAAAAATNTTATTAATTTTTTTCTNTTNACATTTATTCCTACCGATTTTCTTATTTTAATTTAATTTTCTACTGAATAATCTAAAAGTNATAATGAAATAANTGNANAAGGTGCTTCTGNACTAGGAACTGCATTAAANAAGTGCACAAATCTCACAAGTTTGACTCTTTACCTCAAGTAAATATCTTATNTAATTTCTGAATTTTCTNTTAAAAATTTGATTAATTTTCTTTTCGTAACANCCTATTCCTTTCCATCAAATTTTCTTAGACATNTTNTTAATCATAATTTTTTAATTAAATAATCTAAAAGNAATAATAGATAGATGCAGAAGGTATTTCTGCANTTGGNACTGGACTNAGCNAGTGCGCAAATCTCACAANTTTGACTCTTTAACTCGNGTAANAATNTTTTATTTAAATTAATTGAATAAATTATTCTTNTTGATTGTTGATTAATTGTATTTATTAAAACTTTTTTCATTTATTATTTTCAATTTTTAAAATTAATATTTAAAAGATATGAAATTGCTNAGTTTGCAACACANCTAGATTGAGAAGAATAATTAGGCTTACTNATTTGAAAATTCTGACGTACCTTCTCAATATTGAAAAAATAAATAAATAATTATAAAATTTGNTTTTGTTTTGAAGTTATTATTTATTTGTATACTCATG

>T.vorax_rnd-5_family-803#LRR_CRS4

AAAAACAATAAATTATTAAGTAATTTTTAATTATTTCTATNAAATCGGAATTTAGATAAAATATGTACTTAAAAAGACTAATAGGAGGATATTAGATTATCGATTATGATTTTTTAAAGAATAATATTAAGAAGGATACTTGTATAATAGTTTTCTTCATTGTTTTACTCTTTTTTCAAGTAAAATCTTGAAAAGAAATAGAAAAAATATATTTTTGTCNTTTTCAAGCTAATATATCTTAAAATAATTTGATTTCACACCTACTATGATTTTNGAATAAACATTTCTCTAAATTTAAAAATTTTAATTCACTNTATATCTATTTGTCTAATATGAGAAAATCTCTTTCTTAAATCAACTAGAATATCCTCATTTAATCTATTTAAANGAAAAAAAATTTATTAATATACAAATTACCTAAATATAATTTTATGAGTTAAAATCAGAGATAATCTCTCAGATATTCAACTCATGCAATCATTTTCNATAATATTTACTNTAAATNTAAGGTTAGCTGAGATAAGTTTTTGAGCTGTGACAACCCCTCACCAAGTTTACTGGCACCATCATCATTAATTTNATTTNNACTATTAACATAATAAATGAAGTAAATTGAAGGAGAGTATTTGTAAGGAGTAAGGCTTTATGAGTTAAAATCAGAGATAATCTCTCAGATATTCAACTCATGCAATCATTTTCCATAATATTTACCTTAAATNTAAGGTTAGCTGAGATAAGTTTTTGAGCTGTGACAACCCCTCACCAAGTTTACTGGCACCATCATCATTAATTTGATTNCCACTATTAACATAATAAATGAAGTAAATTGAAGGAGAGTATTTGTAAGGAGTAAGGCTTTATGAGTTAAAATCAGAGATAATCTCTCAGATATTCAACTCATGCAATCATTTTCCATAATATTTACCCTAAATCTAAGGTTAGCTGAGATAAGTTTTTGAGCTGTGACAACCCCTCACCAAGTTCACTGGTTTTAATAAGAATATTAAATATTATTTAAAAAAAAAAAAAAAAAAAAAAAAAAAAAAAAAAAAAAAAAAAAAAAAAAAAAAAAAAAAAAAAAAAAATAAAACAATGTAATTTATTGAAATAGAATAACATTCGATTCTATTAATTCATAGGAAATATTCAAAGTTATTAAAAAATTACCTAATGTTTTTTTATTCTAATTTCAAGATCATCCAATTTATATTTTATATTGTNGAAAAATTGAANNTTGGATCTTCTGAATATAGCTTCGATAANTTGTTTTTTGTGTCATCATATATTAGAACNTTATTCCTCTCCTATTTCTAGAAGATAGACTATNAGATATCNTTGTTATTTTGCTTAATAACACTAATATTTTAGAATAAGTGAGATAACTCCATTTGTTCCATTTCNGATAATAGCTAATCAGCATTNAGTCTNTCTACTGCATTTTATCTGACCATCTTATACAGAATCTTCTNAGTAAATTCTGATTTATCCTGAATAATGTCTTTATATGAGTCACTTCTAAACCTTCTTCCTTCTCCGACATCTATTTTTCTCCCAATCATAGCCTCATAAATGGTAATTCCTAANGAATACACATCAACCTTNATTAAATAAAATTGAAATAAATTAANATGAGNGATAAATAATAAATACCTACCTTAAAGGAAAANGGTTTGATTTCATTTCTGACTTCATATGGTATATAGTTGAAAGTGCCAATTCCTTATTTCTAAATAATAAATTATTGANTTATTNTTGAAAGAGTGAGTTTGCATTACATTCTGACTTTAAGTGCTTTCAATGCNGATATTCTTGATNATTTTTGAGATTCCAAAATCAGCTTTAATTAATAAGTAAATAATATTAATTAATTATTTGATATTGTTTTTGTATCTATTTACTGTATTTGAAATAATTAGTAGCGGAACAATAAAGGATATTATCTGGTTTAATATCGAAATGGATAACGCCTATCTTTTGCATTTTTTTCAATCCTTTGAGGAGATGTTTTGTCATGTAAGCGATCTAATCTAGCGAGAATTTGTTTTTATGAATCTTGAAGAGATCTGTGAGATTACCTGGTTTTTAAGTTAAAGAATAGCATTTTATATGAATACATATTCTACTGAATGCTAATTATATTACAATCGCATAATTCAGAGACAATAAAGTATGTATTGATTTCTTCCATATAGAAATCATCNAGNATCTAAACAAAGTATTTTGGGTTTGCTAAACGCTTTGAAATATTCACTTCATTTTACAGATACTGTAAATCCTATGGCTTAACAGATACGCACTTNATTGCTACTTTTTGATTGTTCTCATCAACTGCTTAGATCACAATTCCGNATGATCCTGCTCCTATTATCTTCTCAAACATATATTTATTTTACCCATAGTCTGGCCTTTCTTTCAGATAGCGTAACCTCATGTAAGTTAGAAAAGGATCCTAATCATTAGAAATACAGAGTTTTATGAACTCTGGACTCAAATTTTAATCTTCAATTTAGATTTTATCCATATTAATGTNTTATTTACTTATCTACTTTTATCTAATCTTTTCAATTACTTACAAACTGTATTTATTAATTATTNAGAAGAATATGATATTCTAACACCCANTATACCAAACTCTTTGTATGTTATCTTATTATCTATATNAATGGAATATCTATTGTTTTCTTTGTTTCTTTGCAATGAATGTTTAAGAAATATTGAATTTTATTGATATCGTGAACAAATGACATAAACTTGNTTTTTTAAGAATGAACATTTTAAATTTATTTTTATTAATTTTTAAAAATAAAAAAATAATGAAATTTATTAAATTAATAATTTTATNATTTATTTAAATAAAAAGATATGTTTATTAGTGATTAAATATNTTTGAAAATTAATTTAATTAATGATATACACCTTATTCTATAGCAACTTCATGACTTTTAAATAATCAATAACAACGAATAAAAAGGAATCGTAATTTTTTCTTGTATTTCGTAAGAATATTCATAATTCTTGCATTTGATTATTGAATATAATTTTTCTGAATTTACCGATTACTANCAATAATTTAAAAAAGAAATACTAAAAAGATAACAATAAAATTATTTATACTTTGGATTCTCAATAATTAAGACCTATTCTAAAAANTTGTAAATATGCCCTTAAAAATTTGAACTANGATATTNAANGAGTTATNAGNATATTGATTGAANATAAGAGTTAGA

>T.vorax_rnd-5_family-94#LRR_CRS5

AAAAAAAAAAAAAAACACTCTTTATTGANGATACAAATTTTATGAACTTTAGTTCATAATAAATAAATACTTACTCNAGGTCGAGAGANANAGTTGAGAGATTCGCGCACTTTCCTANTTCATTTCCTAANCCTANAGCTCCTTNNTCGCCTACGTTATTNTTCCTTTTAGATTTAANTAAAGAGAATATGAGANNAATTTAAATTATAGAAATTTTGTTGAAAAATAATAAGTTAATAAATAAAATATTAAATTTTAATATATATTTTACCTNAGATCGAGAGNTAAAGNTGTGAGNTTAANGCACTTNCCTAATTCATTTCCTAATCCAAAAGCACCTTNTTCACCAATNCTATTCTCACTTTTAGATTAATAAAAATTATNTTAATGAGAATAAGATNGTAAGAAATATNATTAAAGAAATAATTAANATGTGAAAGATAAAAANATTGAANTTAANANATAACTTTATCANNAAATGCCANNACATAATTNAATATAAATTAATTTTACTTTAGGTCGAGAGACAAAGATGTGAGATTTGTGCACTTTCCTATTCCAATTCCCAATCCTAAGGCNCCTTACACNTCCNATGTCATTCCCCCTTTTAGATACAATTTAAGGAANTTTATAAAAAAAAAAAAAAAAAAAAAAAAAAAAAAAAAAAAAAAAAAAAAAAAAAAAAAAAAAAAAAAAAAAAAAAAAAAAAAAAAAAAAAAAAAAAAAAAAAAAAAAAAAAAAAAAAAAAAAAAAATTNAGTAATAAAGAAGTTANTNTAAATATTTACTTTAGTTAAAGAGTCAAANTGAGAGATTAGTGCACTATTCTAATCCGTTGNCTAATCCAAANACACCTTNTGCGCCTATCTNATTNGANCTTTTANATAATTGNGATATTTACAGAATANNATTGATGAAANATAAGTNTTGATTGTATCATTAAGATGNGAAAAGAGAACAGTTAATCAAAATATTATTAGAAAATNTCAAGAATTGTAATATGAATATTTACGTGATAATNAGATGCNAAAATGTTGTNCGATTCGTGAGCCAGCCTAATNNTNTCCCTGAATCTAGAAGCACCTTATGCATCGANCTCATTATCTCCTATTAAATATTAAATTTCAGAAAATTGAATATATAANTAAATGCAAAAGTNAAAATTATANTTTTGATAGATGCAAANAATACATTAAAAAAAGATAAAATTTGAATATAGATTATTACCCGANGTAAAGATTCAAAGTTGTGAGATTGGTGCANTAGCCTAATCCAATTNCCAATCTAGAAGCACCGTATTCATCAATNTNACTATTCCTTTAGTTTAATGAATTATATTANTGANAGATTAAGAATGGCGAAATAAANTAATTTTGATTGAAAGGAATAGTTAAAAAAAGATNAAAATTTAATTTAATTTATTACTCGAGAATCAAACTTGTGAGATTGGAGCACTAGCCTAGTCGCAACTNCNAGTNCAGAAGCACCCTCTGCACCTATCTCATTATTCCTTTTAGATATTTAAATGATTGGATTAACTTTTGAAGCGGAAATGAATGATATAAGAAAGAAAATTACCTTAGATCTATTTCAAGNTAAANATGCCCNATAAGATGTGATGAAAGAAACTATTATTAAAAAATNAAATATAATAGCAAAAAGTTAAAAAAATAAATATTACTTGTTTTAGATTATTGTATTTATTTATCTATTANAACATTTTTTATTATTTAATGGTTTNTTTTTAAAATTATGATTTCGTTTATTAATAAATCTTTATTAATATCTAATNAT

>T.borealis_rnd-3_family-81#LRR_CRS1

ATTATTTAAGTCAATAAAAAAATTATAGTAAATATTTACCTATAATTAAGTGTCAATTTATTTGCTAAAATATTTTGTTGAATAAACTTTTAATTTATAAATAATATAATTTTTTAATATTATTTNAATAATAAGGTATTTACATTTTTAAATTAGTCTTCTATCATTTTTTATTGTAGAATATTTTTCTACCCTATTGTATTCCTAACAAATTTAAATTAAAATGTTCAATATTTGATAAAAATTAATAAAAGAAAATGTGTTGTCNTTTATAAATTAGAGTTCAAAATTGAAACTTTCTAACAAATACAATATAATGAATTTATAAAATAAAAACATATTGTATAGATTTTTTTTAATTAAACTTCGTTTAATTTAAATATAATTAAATACGTATTGNAAAAATATACTATTTCCTTTTTTATAAAATTNTTTAATAATATTCAATTCAAAATTTGTGGTGTTAAANTTGTTTATAAAATATTTAATATATATTCAAATGTATATTTTACAATAATATAATATTAATCCATTTATTTAAATTAATAATTTATTTCTTATTTAATTATCTATCTTTCATTAGTTAATTCACCATTCTCTATCAAATTTATATTTTAAAAAACATTAAATTATATAAATAATATTTGTTAATTTTTACCTTAACTCTTTCACCACTAATCTCTTNATTCTCATTATTTAAGATTATANAACCTNTAAATTTTCTAATCTTTTATTTNAAATATTTAAAAATTAATTTAATAAAAAATAATAAAATGGATACTATAAATAAATAATGAATAGGCAAAGAAAATAAAATAATATAAAATTTTACTNTAAATNAAGAGTTAATGTAGTAAGTTATGAGCAATTTCCTAANGCAGACCCTAAACCACAAGCACCTTNATCTCCAATGCTATTACTNCTTTTATATTTAAAGAGTTAAGTAAAAAAATAACATAGTAAAGAGAATGAAAAGTGGAAGAAAATAAAAATTATAAATTTAACTTTAATTAAAGAGTTAATNTAGTA

>T.borealis_rnd-4_family-41#LRR_CRS2

CCAGATACTAAACCATAAACACCGTTATCTCCAATNCTATTATCNCTTTTATATTTAAATAGTTAAGTAGATAAATAACAAAATAGAGNGAATNAATTTGTNTAGAAAAGTGGAAGAAAATAAAAATAAAATAAATTACGATAATTCAAGAGTTAATGTAGTAAGTTATGAGCAATTTCCTAATGCAGATCCTAAACCACAAGCACCTTNNTCTCCAATGCTATTNCCGCTTTATATTTAAAGAGTTAAGTAAAAAATAACAAAATAGAAAGAATAAAATTTTTTATAAAAATTGAAAAAAATAATAATAATATAAAATCTTTAATAATAAATTTCTAATTTAANATAAAATNATTTAAAATAATAATTTTTAAGATNANTTTTTNAATAAATAAAAAAAAAATTTTTTAAATTAAAATTTAAAATATATAATTTTACTATAAATTAAGAGTTAATGTAGTAAGTTATGAGCAATTTNCTAATCCNGATCCTAAACCATAAGCGCCNTTATNTTCAATNCTATTATTCCCTTTTATATTTAAAGATTAAGTAAAAAATAATAAAATATAGAGAATANATTTGTTTTAAATAGTGAAAGAAAATAAAATAAAATTTTACTNTAAATTAAGAGTTAATGTAGTAAGTTATGAGCAATTTCCTAATNCAGATCCTAAACCACAAGCACCTTNATCTCCAATNTTATTTTCNCTTTTATATTTAAAGAGTTAAGTAAAAAATAACAAAATAGAGAATATTTNTAATTGAAAAATGAAAATTAATATAAAATTTTACTATAAATNAAGAGTTAATGTAGTAAGTTATGAGCAATTTCCTAATCCAGATCCTAAACCACAAGCACCCTNATCTCCAATNTTATTTTCNCTTTTATATTTAAAGAGTTAAGTAAAAATAACAAAATAGAGAGAATNAATTTGTAATGAAAAGTGGAAGAAAATAAAAATAATATAAAATTTACNNTAAATCAAGAGTTAATGTAGTAAGTTATGAGCAATTTCCTAATCCAGATCCTAAACCACAAGCACCCTNATCTCCAATNCTATTTCCACTTTTATATTTTAAAGANTTAAGTAAAAATAATAAAATTATAGTATAAGTTATTTAGAAAATGTAAGTAATAAAAATAATAAAAATTTTACGAT

>T.borealis_rnd-4_family-43#LRR_CRS3

AGTTATGAGCAATTTCCTAATCCAGATCCTAAACCACAAGCACCCTNATCTCCAATNTTATTTTCNCTTTTATATTTAAAGAGTTAAGAAATAATAAAAATAGAGAGAATAAATTTGTAATGAAAAGTGGAAGAAAATAAAAATAATATAAATTTACCTTAAATCAAGAGTTAATGTAGTAAGTTATGAGCAATTTCCTAATCCAGATCCTAAACCACAAGCACCCTNATCTCCAATNCTATTNTCGCTTTTATATTTAAAGAGTTAANTAATGAAATAATAAAAAGAATAAATAAAATTTTTTATNAAAAGTGNAAGAAAATAATATAATACTGTAAATTTCTTAAATATAAATTTCTAATTTAAGATAAAATAAGTTNAANATAACAATTTTTNTAATTTAAGATAATAATTTAAAAAAAAAAAATTTTTTATTAAATTAGAAGTTTAAAAATATATAATTTTACTATAAATCAAGAGTTAATGTAGTAAGTTATGAGCAATTTCCTAATCCAGATCCTAAACCACAAGCACCCTAATCTCCAATNCTATTNTCNCTTTTATATATTAAGAGTTAANAAANTATAATAAAATAGAGAGAATAAATTTGTAATGAAAAGTGGAAGAAAATAAAATAAATTTTAACTATAAATNAAGAGTTAATGTAGTAAGTTATGAGCAATTTCCTAATCCAGATCCTAAACCACGAGCACCCTNATCTCCAATNTTATTNTNNCTTTTATATTTAAAGAGTTAAGTAAANAATAAAAATAGAGAGAATAAATTTGTTTGTAAAGTGAAAAAAATTTAAAATTTTACNNTAAATCNAGAGTTAATGTAGTAAGTTATGAGCAATTTCCTAATCCAGATCCTAAACCACAAGCACCCTNATCTCCAATACTATTTNANCTTTTATATTTAAAGAAATAAGTAAAAATAATAAAATAGAGAGAATAAATTTGTAATGAAAAGAAGAATAAAATAAATTTAAATTTTACNTCAAATCAAGAGTTAATGTAGTAAGTTATGAGCAATTTCCTAATCCAGATCCTAAACCACAAGCACCCTAATCTCCAATNNTATTTCCACTTTTATATTTAAAGATTTAATTAAATAAAATAAAATAGAGAGAATAAATTTGTTAGAAAAATGAAAGAAATAAAGTAGTATAAAATTTTACTATAA

>T.borealis_rnd-5_family-143#LRR_CRS4

AAATAAAATATTATAAAACTTTACTCTAATTAAAGAGTTAATGTAGTAAGTTATGAGCAATTTCCTAATGCAGACCCTAAACCACAAGCACCTTNATCTCCAATNCTATTNTNNCTATTATATTTAAAAATTAAGTAAAAAAATAACATAGGAGAGAGAATAAATTTGTGNTGAAAAGTGGAAGAAAATAAAATAATATAAAATTTTACNTTAATTNAAGAGTTAATGTAGTAAGTTATGAGCAATTTCCTAANGCAGACCCTAAACCACAAGCACCTTNATCTCCAATGCTATTTCNCCTATTATATTTAAAGANTTAAGTAAAAAAATAACANAGCAGAGAGAATAAATTTGTGTTGAAAAGTAGAAGAAAATAAAATAATATAAAATTTTACNNTAAATNAAGAGTTAATGTAGTAAGTTATNAGCAATTTCCTAANGCAGACCCTAAACCACAAGCACCTTNATCTCCAATGCTATTATNCCTATTATATTTAAAGAATTAAGTAAAAAAATAANATAGCAGAGAGAATAAATTTGTGTTGAAAAGTGGAAGAAAATAAAATAATATAAAATTTACNATAAATCAAGAGTTAATGTAGTAAGTTATGAGCAATTTCCTAAAGCAGACCCTAAACCACAAGCACCTTAATCTCCAATNTTATTNTANCTTTTATATTTAAAGAAATTTTNNAAGTCAATAAAAAAATTANAGTAAATATTTACCTAAATTNAAGTGTCAATTTATTTGCTAAAATATTTTGTTGAATAAACTTTTAATTGATAAATAATATAATTTTTTAATATTATTTTAATAATAAGNTATTTACATTTTTAAATTAGTCTTCTATCATTTTTTATTATAGAATATTTTTCTACCCTATTGTATTCCTAACAAATTTAAATTAAAAAGTTCAATATTTGATAAAAATTAATAATNGTAAATTAGTTATCTTTTATAAATAAGAATTTCAAAATTGAAATTTTCTAACAAATATTAATATTATAATGAATTTATAATANAAAATTATATATACTATNTGGAACAAAAATTNTAACTTTTTNANAATAAAAATTTCATAATTAGAATAATTTATTCTCGTTCTAATTCGAAAAATTATTTTATTTTTCATAAAAATTTAATAATTATATATTATATTATAATTCATTATATTGTATTGGTTAGAAANTTTCAATTTTGAACTCTAATTAATAAAAGTAAAATATTTTCTTTTATTAATTTTTATCAAATATTGAACATTTTAATTTAAATTTGTTAGGAATAACAATAGGGTAGAAAAATATTCTATAATAAAAAATGATAGAAGAANAATTTAAAAATGTAAATGCCATATTATTCAAATAATATTAAANAATTATATTATTTATCAATTAAAAGTTNATTCAACAAAATATTTTAGCAAATTAATTGACACTTGATTCTAGGTAAATTTTACTTTAATTTTTTATTGNCTTAGAAAATTTCTTTAAATATAAAAGTAAAATAGTATTGGAGATTAAGGTGCTTGTCGTTTAGGGTCTGCTTTAGGAAATTGCTCATAACTTACTACATTAACTCTTAAATTAGGGTAAAATTTTATTTTATTCCACTTTTCAANACAAATTTATTCTCTCTCCTATTTTATTTTTTTATTTAACTCTTNAAATATAATAGTNACAATAGCATTGGAGACCAAGGTGCTTGTGGTTTA

>T.canadensis_rnd-2_family-16#LRR_CRS1

TACTATTTATTACTCATTGCAGAAAATAATTGATGCTTTAATTCATTGAATTAATGTAGTATCCACTGGTTCTTATTTTATTATTAATTTTAAANATTTCAAATAAATACTGATAAAGCCTGAAGAATCTAATTAGGAGCCAATATGAAAATAAAATTACTCTATTGATTAGAATAGTACTTAATTAGTAGCCCCAGTTTATTTTTTCTTAATTTGTATCCCTTTATGTATAAATTTTTAAAAATATTTGATNTTTATATAATTATGAATNAATTTTTAAAATATANAATAAAACAAGTTNTAAAACCAAATACTAGCCTGAATGTGGTAATATTTTTAAATTCAATGAATTATTTTATTTTTTCATTCTCAAATTTAAAAATTTAACAACCATGTTTTAATTATAAATTCATTATATTGTATTGGTTAGAAAGTTTCAATTTTGAACTCTAATTTATAAAAGACAANACATTTTCTTTTATTAATTTTTATCAAATATTGAACATTTTAATTTAAATTTGTTAGGAATACAATAGGGTAGAAAAATATTCTACAATAAAAAATGATAGAAGACTAATTTAAAAATGTAAATACCTTATTATTAAAATAATATTAAAAAATTATATTATTTATAAATTAAAAGTTNATTCAACAAAATATTTTAGCAAATAAATTGACACTTAATTATAGGTAAATATTTACTNTNATTTTTTTATTGACTTAAAAATTTCTTTAAATATAANAGCGGCAATAGCATTGGAGATCAAGGTGCTTGTGGTTTAGGGTCTGCTTTAGGAAATTGCTCATAACTTACTACATTAACTCTTNATTTAGAGTAAAATTTTATATTATTTTTATTTTCTTCCACTTTTTAACACAAATTTATTCTCTCTCCTATGTTATTTTTTTACTTAACTCTTTAAATATAATAGGAANAATAGCATTGGAGATCAAGGTGCTTGTGGTTTAGGGTCTGCTTTAGGAAATTGCTCATAACTTACTACATTAACTCTTNATTTAGGGTAAAATTTTATATTATTTTTATTTTCTTCCACTTTTCAACACAAATTTATTCTCTCTCCTATGTTATTTTTTTACTTAACTCTTTAAATATAATAGGAACAATAGCATTGGAGATCAAGGTGCTTGTGGTTTAGGGTCTGCTTTAGGAAATTGCTCATAACTTACTACATTAACTCTTNATTTANGGTAAAATTTTATATTATTTTTATTTTCTTCCACTTTTCAACACAAATTTATTCTCTCTCCTATGTTATTTTTTTACTTAACTCTTTAAATATAATAGNNGTAATAGCATTGGAGATNAAGGTGCTTGTGGTTTAGGGTCTGCTTTAGGAAATTGCTCATAACTTACTACATTAACTCTTTATTTAGAGTAAAATTTTATATTATTTTAAT

>T.canadensis_rnd-3_family-2#LRR_CRS2

AAAAATATTTTATATATTATATTATTTTCTTCCACTTTTCAACACAGAATTTATTCTCTCTGCTATGTTATTTTTTTACTTAACTCNTTAAATATAATAGNTNTAATAGCATTGGAGATGAAGGTGCTTGTGGTTTAGGGTCTGCTTTAGGAAATTGCTCATAACTTACTACATTAACTCTNGATTTAGNGTAAAATTTTATATTATTTTTATTTTTCTTCCACTTTTCAACACAAATTTATTCTCTCTCCTATTTTATTTTTTTACTTAACTCTTTAAATATAATAGNAATAATAGTATTGGAGATNAAGGTGCTTGTGGTTTAGGGTCTGCTTTAGGAAATTGCTCATAACTTACTACATTAACTCTTGANTTAANGTAAAATTTTATATTATTTTATTTTCTTCCACTTTTCAACACAAATTTATTCTCTCTGCTATGTTATTTTTTTACTTAACTCTTTAAATATAATAGGTAGTAATAGCATTGGAGATTAAGGTGCTTGTGGTTTAGGGTCTGCTTTAGGAAATTGCTCATANCTTACTANATTAACTCTTTAATTAGNGTAAAATTTTATATTATTTTATTTTCTTCGCCTATTCATTATTTATTTATAGTATCCATTTATTATTTTTTATTAAATTAATTTTTAAATATTTCAAATAAAAGATTAGAAAATTTATAGGTTATATAATCTTAAATAATGAGAATAAAGAGATTAGTGGTGAAAGAGTTAAGGTAAAAATTAACAAATATTATTTATATAATTTAATGTTTTTTAAAATATAAATTTGATAGAAAATGGTGAATTAACTAATGAAAGATAGATAATTAAATAAGAAATAAATTATTAATTTAAATAAATGGATTAATATANATATTATTGTAAAATATACATTTAAATATATATTGATTAAAATAATTAAAAATAATTTNAACACNACAATTTTGATTTGTATAAAAATTTNTATTAAATCATTTTCATTCTCAAACTTTAATTGAAAAAGTATTTTNTTTTATTAATTTTTATTAAATATTGAATATTTTAATTTAAATTTATTAGGAATACAATAAGNTAGAAAAATACTCTACAATAAAAAATGATAGAAGAATAATTTAAAAATNTAAATACCTTATTATTCAAATAATATTAAAGAATTATATTATTTAAATTAAAAGTTGATTCAATAAAATATTTTANCAAATATATTGAA

>T.canadensis_rnd-4_family-41#LRR_CRS3

AAATTGTAAATAAATAATATAAGTTAANTAAAATNTAATATCTTTTTAGATTAGATAAGAATTTAAAAAAATAATTAATTTTCTCAAACCCTAATCTANATGAATTGCATTATATAATCTCTGATCGTAAATGNGTAAATTTAAAATTAGAAATTAAGTAAATTTAATTAATTCTGATAAAACATAAATATTAAAAATNAGAATTTAAAAAAATAATCAATTTTCTCAAACCCTAATCTAAATGAATTATTATTTTCTGATCATAAATGGTAAATTAAANTTTGAAATTAAGTAAANTTAGATTAATTCTGATAAAATATAGAAGATAAGAATTTAAAAAATAATTAATCTTCTCAAACCCTAATCTANATGAATTATTATTTTCTGATCATAATTGAGTAAATTAAAATTTGAAATTATATAGAAAATTTATATTATTCTGATAAAATATAGATATAAAAAGATAAGAATTTAAAATAATAATTAATTTTCTCAAACCCTAATCTANATGAATTATATTATTTTTGATAAATTAGATTTTGAGATTAAAGAAATAANTTAGATTAATTATAATAAATATTGGTTATAAAAATAAGAATATAATAAATAACTAATTTTATCCCAAATCTATATGAATTACATTATCTGCTTTTCTTTTTGAGTAAATTAAATTTTGAAATTATATAAGATTAATAATATTNNGATAAAACCTTGAATAATAAAAATAAGAATTTAACAAAATAATTAATTTTCTCAAACCCTAATCTANATGAATTTCATTATANAATNNCTGATTATAATAAATAAATTTTGAAATTATTTTAAGTANATTTAAATTATTTTAATAAATATAGATTAATAAAATAAGTTTCNAATATATATTTTACAATAATATAATCATTTATTTANATTNATAATTTATTTCTTATTTCTTTCATNAGTTAATTCACCATTTTCTATCAAATTAATATTTTAAAAAATATTAAATTNAATAAATAATATTTGTTAATTTTTACCTTAATTCTATCACCACTAATCTCTTTATTCTCATTATTTAAGATTANATAGCCTTTAAATTTTCTAATCTTTTATTTGAAATATTTAAAAATTTAATAAAAAATAATAAAATGGATANTATAAATNAATTATGAATGAAAAGNAAATAAAATAATATAAAATTTTACTNTAAATCGAGAGTTAATGTAGTAAGTTANGAGCAATTTCCTAATCCAGATCCTAAACCACAAGCACCCTAATCTCCAATNCTATTATNCCTATTTTTAAATAGTTAAGTAAAAAAATAANATAGTAAAGAAAATAAATTTGTGTTGAAAAGTGGAAGAAAATAAAATAATATNAAATTTTACCNTAAATNAAGAGTTAATGTAGTAAGTTATGAGCAATTTCCTAANNCAGACCCTAAACCACAAGCACCTTAATCTCCAATGCTATTGCTCCTATTATATTTAAAGAGTTAAGTAAAAAAATAAAATAGGAGAGAGAATAAATTTGTGTTGAAAAGTGGAAGAAAATAAAATAATATAAAATTTTACTNTAAATNAAGAGTTAATGTAGTAAGTTATGAGCAATTTCCTAANGCAGACCCTAAACCACAAGCACCCTNATCTCCAATGCTATTATNCCTATTATATTTAAAGAGTTAAGTAAAAAAATAACATAGGAGAGAGAATAAATTTGTGTTGAAAAGTGGAAGAAAATAAAATAATATAAAATTTTACNNTAAATNAAGAGTTAATGTAGTAAGTTATGAGCAATTTCCTAANNCAGATCCTAAACCACAAGCACCTTNATCTCCAATACTATTNTANCTTTTATATTTAAAGANTAAGTAAAAAAAATAAAATAGGAGAGAGAATAAATTTGTGTTG

>T.canadensis_rnd-5_family-174#LRR_CRS4

AAAATAAAATTTAATTAGTNAAAATAAATAAATATCATAAATATTCTTTTATANCTAATAAANTATAATATNATTTTATTTATAATTTAATATTATAATTTAGAAANTATATTAATTTGTAAAAATATTAAATAAAATTATTTAAATATATTTATTAAATTTAGACTTCAATTAATTTTAANTAAAAAAATAAATAAATTATTATATAAAATATAAANNTATTTNGTAAAATTCAATAAAACATTNNTTTATATAAATATAAATAATATATNTAAAATTATTATTAAGATAAACGAATAAAATAATATAATAAAATTTTTTTATTAAATTTTANCAATTAATTTAGTGATAGAATATTTTCNTCAGNATTAATTTAACTCTATTAATTTAGTTCCATTTAAATTTAANTAAGTCAGATTGTTGAGTTTAGATAAGCCTTCTCCTAATTTACTAGCCCCTTCATCACTGATTTGATTNNNNCTTTTACAATTTATAAAAAAGTTAGTTTTCTGATATTTTAGCAATTAATTTAGATAGATTAAATCAGTTTTTGCTCAATATTATATTATGTTATTGAATTAATTACNATAGATCTAAATTTAACTAAGTCAGATTGTTGAGTTTAGATAAGCCTTCTCCTAATTTACTAGCCCCTTCATCACTGATTTGATTGTNACTTTTACAATTNATAAAAAAGTTAATTTTCTGATATTTTAGCAATTTAGATAGATTAAATCAGTTTTGCTCAATATTATATTATGTCATTGAATTAATTACCTAAGATCTAAATTTAACTAAGTNAGATTGTTGAGTTTAGATAAGCCTTCTCCTAATTTACTAGCCCCTTCATCACTGATTTGATTNCCACTTTTACAATTNATAAAAAAGTTAATCTTCTGATATTTTAGCAATTATTTTAGATAGATTAAATCAGTCTTTGCTCAATATTATTATTCAATTAAATTACCTTAGATTTAAATTTAANTAAGTCAAATTNTTGAGTTTAGATAAACCTTCTCCTAATTTGCTAGCCCCTTCATCNCTGATTTTATTTTCACTTTTATAATTTATAAAAAAGTTAGATTAAATCTCTTATTTTTCAATATTTATTTTATTCAATTAATTACCTTAAATATAAATTTGACTAATATAAATTTTGAGTTAAGATAAATTCTCCTAATTTAAATAACCCNTTAATTACTAATTTAATTTCATCTTTTAAAATTTATAAAAAAATTAATTTTTTTANTATTTTATCAATATTNATTTATTCAACTACCTTAAATNTAAATTTAATTNAGTTAAATTATTGAGTTTAGATAAGCCTTCTCCTAATTTACTAGCCCCTTCATNACTGATTTGATTTTCNCTTTTACAATTTATTACAAAAAAGTTAGTTTTCTGATGTTTTAGCAATTAATTTAGAGAAAAAATGAGTTTTTGCTCAATATTTATTTTTGAATTCAATTAATTACTNTAGATCTAAAGTTAACTGAGTCAGATTGTTGAGTTTAGATAAACCTTCTCCTAATTTACTAGCCCCTTCATCACTGATTTGATTNTAACTTTTACAATTTATAAAAAAGTTAGTTTTCTGATGTTTTAGCAANTAATTTAGAGAAAAAATCATTTTTTGCTCAATATTTAATAAATTCAATTAATTACTNTAGACCTAAAGTTAACTAAGTCAGATTGTTGAGTTTAGATAAACCTTCTCCTAATTTAGTAGCCCCTTCATNACTGATTTGATTTTCACTTTTANAAATTATAAAAAAGTTAGTTTTCTGATGATTATGAAATTAATTTAAATTAATTACTATAGATNTAAAGTTAGCTGAGTCAGATTGNTGAGTTTAGATAAACCTTCTCCTAATTTACTAGCCCCTTCATNACTGATTTGATTTTCACTTTTACAATTTATAAAAAAGTTAGTTTTCTGATGATTTAGCAATTAATTTAGAGAAAAAATNATTTTTTGCTCAATATTATTTTATGAAATTCAATTAATTACTNTAGATCTAAANTTAACTGAGTCAGATTGCTGAGTTTAGATAAGCCTTCTCCTAATTTAGTAGCCCCTTCATCACTGATTTGATTTTCGCTTTTACAATTTATAAAAAATGTTAGTTTTCTGATGTTTTAGCAATTAATTTAGAAATTAAATCAGTTTTTTAAATTCTATTAATTACTATAGATTTAAAGTTAACTGAGTNAGATTGTTGAGTTTAGATAAGCCTTCTCCTAATTTACTAGCCCCTTCATCACTGATTTGATTTTNACTTTTACAATTTATAAAGTTAGTTTTCTGATGTTTTAGCAATNATTATTTAAAAATTCAATAGAAAAATTAAATTTTGGAAAAATCTTTATTTATATTATNTTTATGAAATAANTNAGTANAATATAATAAATTATAAATTAAGTAATTTTAAATAAGTTAAATTGNGAGTAAAATAGAATTAAATTAGTTTTATTAAGATATCGGAATTAATGAAAAATTCANAAAAAAATTTAATAATAATATGAGCAAAAAAATAAAAATATTTCAATCCTTATTTCAAATTAGATCAATTTATTTTTAATAGAATATATTAAATTAANTTGTAAATTTTAAAAAATTATTACTTTAAATTTAAATGAAGCCAATTAATGTTAGGATATTGAGAAAGCATAGGAATAATTCCTAAAGAAAATA

>T.shanghaiensis_rnd-4_family-129#LRR_CRS1

AAAATTATTTTTTATTTTTTATTAAAATGCTTTATTGTTTAAAATAATATTTTTNCATAATTAAAATTAATCTCATTTGATATTTAATTAAAATTTTAAAAATNAATTATTTTTTTTCTAAATAAAATAATGCTTTGATTTAAGCAAATATAATCGATTTGGAATAAATCATAAATAAAAATAATATAAATATTAGCATAGTCTTAAATTTAATTTTATTAGAATTAGATTTACTAAATTGAATTAATTTTTATTTTAGAGATATTTAAATACTTTATAAATATTGTATTTAACTATAAAAAAGGATCAAAGTGAATAGGCACTATNATTTGAAAGTTTTATAAATGAAGTAATTCTATGTTATTTTTTAGATATGATTGATAAATAAATNTATTTTTAAAGTATAAAANTGTAGTNATAAATTTCAAAGAGAGTAAGTTAAATCTTAGTTTGAAGTAATTTTTATTTATTTTAATTCATTTNAAAATGATTNTATAAAATTAATACAAATTCATAAAAGTGGAAATCANATTGGTAGAAATGGTGCTAGCAATTTAGCAGATGCAATTTCAAAATGTGAGAATCTCACTTCTTTAACTCTTGATCTANAGTAATTTTTTAATAATTAAATTAATTTATTTANTAATTTAATAAATATTTTATTCAGAATATTATTTTTTTTTATTTNANTCGTCAATTNATTTTAATTTTAATTAATTTAAATATATAAAAAGTGNAATCGAATTGGTGANAANGGTGCTAGTAATTTAGGAACTGCAATTTCAAAATGTNAGAATCTCACTTCTTTAACTCTTGANCTNGGGTAATTTTTTTAAATTATTAAATTTGAATTAATTTAATAAATATTTATTTTTAAAGNTATTATTCTTAAATTTAAATTATNAATTCGTTNTNAATTTTGNTTATTTTTAATTTATCNAAATAANTAAAAGTNACAATCAAATTGGCGANAAAGGTGCNAGCAATTTAGGAACTGCAATTTCAAAATGTGANAATCTCACTTCTTTAANTCTTGATCTAGGGTAATTTTTTTAAAGTTANATTTTTATATTTTAATTTAATNAATTTATTTTAAATTTTGCTTAATTTTAATTTATTTAAANAATAAAAGTNNCAATNNAATCGGTGANANTGGTGCTAGCAATTTAGGAANTGCNATTTCAAAATNTNANAATCTCACTTCTTTAACTCTTGATCTNAGGTAATTTTTTAATTAAATTAATTTATATAAATATTTCGTTCAAGCTATTATTTTCATANATTTAAATCATCNATTNGTTTTAAATTTTACTTAATTATAATTTATCAAAACATATAAAAGTTACAATCGAATTGGTGANANTGGTACTAGCAATTTAGGAACTGCAATTTCAAAATTTGGNAATCTCACTTCTTTAACTCTTGATCTAANGTAATTTTTTTTATTATTAATTATGANTANATTTTTAAACTAATTTAATTTTGATTTTATTTAATTNTGATTCATTTAAATCAATATAAAAGTNACAATNAAATTGGTNANAATGGTGCTAGNAATTTAGGAACTGCAATTTCAAAATNTGANAATCTCACTTCTTTAACTCTTNATCTTAAGTAATTTTTTATATTTAAATTGATTTATGCTTTTAATTTAATAAATATNTTCAAACTATTATNTTCNTATNTTTAAATCATAAATTTATTTTAAATTTTGNTTNATTTTAATTTATCTNAANTACTAAAAGTNNTAATNAAATCGGTGATACTGGTGCTAGNAATTTAGGAANTGCAATTTCAAAATGTCACAATCTCACTNCTTTAAANNTTNATCTAATGTAATTTTTTAATTATTACATTGTTTTAATTTTAAGTTTTATTAAATATTATTTTTATNTTTAAATTATTAAATTCATTTTAAATTTATTATAATTTTAATTTATCTTAAAAAATTTTAGAGACAATAAAATTAGCGAATAAGCTAGTTTTTTAATTACCAAATACTTNAAAAAACTAAAAGACTTGCCGATCTTATACATCTTTNTCTTGATTGAAAAAAATAATAATTAAAATAAAATTATTTCATTTTAAAAAATAATCTAGCAATGATTAAAAACTTTTAATATTAATCATTCCTAAAAATAANATTATATTTACATTTATGAATAATTNTTTGTTAAATTTATNAAAATTAATGAAANTTAAATCATTAATTAAAACAATATTTTATGATTAGTATTTTATATTTACATTATATTTATGTCGAATGTAAAAATACACCAACATTTTTAAATTTNTTTATAATATAAAAATATATTAATTATTAAAATTAATTCGTAGACTTTAATATTATTTATTTAAAATAATTTAATGATAATGGTGCTGAATTTAGGAANTGCAATTTCAAAATGTGAGAATCTNACTTTTTTAACTCTTAATNTANNGTAATTTTTTTATAATTAANTTGATT

>T.shanghaiensis_rnd-4_family-33#LRR_CRS2

AGCTTTGAATTATTANAAATAATAATAAAATTGATAAAAGAAATTAACAATAAATTAGAAATTGACATCAAATNAAANNAGTAAGAATAATTATTTCAAATAAATTACNTNAGANNAAGANTTAAAGAAGAGAAATTAACACATTTCGAAATNTTTTCTNATAATTTACTGCAACCATNTCCACCAATTTNATTAACTTTAAATTATTAGAATATAATAATATAATTAAAAAAATTAATAAATGAATATGGATAAATAGATATTGTTAATATTTTCACTAAATCAATATAATTTATAANGAATTACNATAGATTAAGATTTAAACGAGTGAGATTAACACATTTTGAAATTCGTTCTCCTAATTTANTTACAGCATCATCATNAATTTGATTTTCACTTTNAGTTANTTGAATAATTTAAAATNAANAAAATAAATTNATTAAAGAATATAGAATGAATTGGGAGAAATAAATATCCTTAAACAATTCAGAGTTTTCACATCACATATCATATTATTATATAAAAAATTACCTTAGTTTAAGACTTAAAGAAGTGAGATTCGCACATTTCCAAATATAATTAGCAAATATTAATAATTGATAATAATCACTTTTTTCATTATTATATTTTATTAGATATTCAAATATTTTAATAATAACATAAATAATTTTATGTTAAAATAAGCTCAAAATCATTAAATTATATAAAAAAAATTTTAAAAAAATTTTATAATCAAATTTTAATAAAAAGGAAGAATAAAAAAAAAAAAAAAAAAAAAAAAAAAAAAAAAAAAAAAAAAAAAAAAAAAAAAAAAAAAAAAAAAAAAAAAAAAAAAAAAAAAAAAAAAAAAAAAAAAAAAAAAAAAAAAAAAAAAAAAAAAAAAAAAAAAAAAAAAAAAAAAAAAAAAAAAAAAAAAAAAAAAAAAAAAAAAAAAAAAAAAAAAAAAAAAAAAAAAAAAAAAAAAAAAAAAAAAAAAAAAAAAAAAAAAAAAAAAAAAAAAAAAAAAAAAAAAAAAAATTATGTAAGAATAAAAATTATACATTCAAGTAAAATAGAGATGAATTTATAAAAAGATTAAATAAAAGAGAGTTGAAATTGTAAAAAATTGAATAAATCATATTTTAAGAATATTATTACTTGGAGGTGTATTGGAAGCTAGCTAGTTTAATCAATTAGAAAATNTATTCCATTANTATNTTTTATACTGTTTTATAAANAATTTTATATTAAAATTATAAAAATATGAAAATGAATATAATTAAATAAATTAATAATATTTAAATAAATTACTNNAGATNAAGAGTTAAAGAAGTGAGATTGACACATTTNGAAATNCCTTCTCCTAATTTACTGCAACCATTCTCACCAATTTTATTGTCGCTTTAAATTATTATAATATAAAAATAAAGATAAATAAAATAAATTAATGAATAATATTTATTAAATAGATATTTNTAAATAATGAAGATTTGATAAATTTAAAATTATATAATTTTATAATAAATTACTNNAGATCAAGANTTAAAGAAGTGAGATTGACACATTTCGAAATACCTTCTCCTAATTNANTNNAACCNTCNTCACCAATNTCATTNTTNCTTTAAATTATTATAATATTAGAATGAATATAAATAAAATAAATTAATNAATNAATGAATTNATGAAATAGATATTTNTAAATAATCAGATTGTCAATATAAATNATATTATTTTATAATAAATTACNAGAGATCAAGANTTAAAGAAGTNAGATTNACACATTTCGAAATNNNTTCTCCTAATTTATTGCAACCATTNTCACCAATTTTATTGTTCCTTTAAATTATNATAATATGAAAAGAATATTAATAAAATAAATTAATNAATGAATNAATTNNATNAAATANATATTTATAAATAATAAGATTTTACTTTATAAATCAAATTATTTTATAATAAATTACNNAAGATNAAGANTTAAAGAAGTGAGATTNACACATTTTGAAATNTTTTCTCCTAATTTANTGNAACCATTNTCACCAATTTTATTACTACTTTGAATTATTAAAATATGAGAATNAATATAAATAAAATAAATTAATTAATGAATTGGATTGAATTAAAATAATTAAATATTTTCAAAAAATACTATTTATNATTAAATTTATAATTAAATATTTTTCCNTTTGAGTTAATATAAATNTTAAATTTATTCATGAATAATNTACTCAATTAAAATTTAAATAANTTTATAAATACTTAAAGATCGAATAANANATTATTGACAAATTTCGTAAAAATTNCATTNANCAAAATATTTAAGCAATATCATTANTTGCAGGAATAATNTTAATTATACTGTNTAATNNATTATAAAATATACTGAAAATAAATATTTTCTTAAAATCAATTAAGTTTTAAAATTATATGTGTATTGCTGCAGAATATTATTCAATTCAATTTAATTTAAAATTTAATTACTAAATTAAATTACNTTAGATGAAGANCTAAAGAAGTGAGATTNACACATTTCGAAATACCTTTTCCTAATTNAATACCACCATTNTCATTAATNNTATTACTTTAATTATTTGATATTNTAATAAATAAAAATAAAATAAATACATTTTTTCTGTTAAAATNATAGTTAAANAAATGTTAANTAAATAAATTCATATCTTGAGTCATAATATTANTTTAGATCAAGATCTAAAAAAGTGAGNTATNCACATTNAGAAATACNTTCAATCAATGCCTTAAACCATTATNTCCAATNTTATTNTCACTGTAAATAATTTAGAAATGGAAAATGAANATAAATAAAATAAATTAATTTGAATAAGATAGAATATATNTTTGTTATTATTATAAATGAATNAATATGGANATAANAAATTAAAATNATNTATTTTTAAAAATATTACCTTAGATCAAGATTTAAAGAAGTGAGATTNGCGCATTTNGAAATACCTTCTCCTAATTTAGCTGCACCATTNTCATTAATNTCACANTCCCTTAAAATTATAGAATATTTGAATGAATATAAATAAATTAAATCAGTTATAAANTAAATAGATTCAAATTAAATATGTATTNAATTAAAATTANTTAATAATAAATTATTTTTAATTACTAAAAATTAACGTCTAAGGAAGTCTCATCGAAACTAAGAGATAGTGTTTTCCCCTTTTAATATTAATAAAAAATATAAATCTAATTTTAAAAGAATTAATTATTTATACNTTCTTAGAAAANCACCAGTTTTATTCCATTGTCTGTCCTGATTAATTATCCATTTATTAACCTGTTACTATTATTTTCAAACAAAATTTATTTATTATTATTTATTAGATAATTTAAAAATAANATTAANGTATTTAAAATAATTAATAGACTTTAATNTTAATAATATAATAGCATTTTATTTTTTATTAAAAANTTTAAAAATATTTAAGTTATTTAACTAAAAATAAATAAATGAATTAATAAAATTAAAAATTACNTTAATTTTNTAGCATTTCTCTATTTGTTTTTTTTTTCCACTTTTTTTTTTTTTTTTGCTTTCAGCTATTNTTTTTTCTTCTTAAATAATTAATTATAAAAAAAAATCAAGCTTTTAAAGTACAAAATTTTTTTATTTCATATCTGTGATTAATAGAAATTTCTTCTAATTTAATTTATCCAATTTTATTCAGTCTTTCTATTTTTAATTTAGATATAAGACTTCGCCAGATATGTGATTGTTTTAATTTTATTATAGGGATANAGAAAAAAGAAATTTTGNTCTATTTTTAATCATTCTTAATAAAAGATTAGTATATCCTTACNNATTTCATNATCNCACAAACCTAATAAAAGAAATTAAAATAAAAATAAAAAATAAAAAAAAATAAAAGAGTTTTATTTTACAATAGATTCGTTAATAAGAAAATACTTATATTTTATAATAAGATAAATAATAATAAAATTAAATTTATAAAAAAATAAAATAATATTAATCAATAAATAATAATAAAAAAATCAAGTTTAAATAAAGTNATTAATTTAGATTACAAATATAAACAATACAATAAAACGTAAAATTCAAACAAAAATAAAATAATTTTTATTTTTNATTCATTTATTTCNAATTTTTTAAATTGATTATGTAATTTNAATTTTAAATTAACTTTAAAAAATTTGTTATTTTNATATTTTNATTTCTTTTTATTGTTTAAAATTTGTATTATAATATTTTGTAAATNATTATATAGTTATCAGTATTAGAANTTTATTTTAGTTAATCTTTTTGTTTTATACAGTTTTTGTTTTAATTTACCAAATTNATATTAGCTATAATTATTCTTTTGTAATGANTTATCAGAGTTAAAGAAGTTAGNTTTNCGCNTTTTTCAGTTTTTGTTCTAATTNACTGAAACCATTTGACAAATTTTATTTCCTTTGAATTATTAAAATATAAAATGAATATAAATAAAATAAATTAATGAATTGGATGAAATCGATATTTGTAAATAATNCAGAGTTNTCACATCGCAAATCATATAATTTTATAATAAATTACNATAGATCAAGAGTTAAAGAAGTGAGATTGACACATTTGGAAATANCTTNTCCTAATTCAATGCAACCATTCTCACCAATTTATTATTNCTTTGAATTATGAGAATATGAGAATGAATATAAATAAAATAAATTAATGAATGAATTGGATGAAATAGATATTTGTAAATAATGCAGAGTTGTCACATCACAAATCATATTATTTTGTAATAAATTACGATAGGTCAAGATTTAAAGAAGTGAGATTTCCACATTTGGAAATGCNTTTTCCTAATTCAATGCAACCATTCTCACCAATATTATTGCCCCTTTGAATTATGAGAATATGAGAATGAATATAAATAAAATAAATTAATGAATTGGATGAANTAGATATTTGTAAATAATTCAAAGTTNTCACATCACAAATAATATTATTTTATAATAAATTACNATAGGTNAAGACTTAAAGAAGTGAGATTTACCGCATTTAGAAATGANTTTTCCTAATTCAATGCAACCATTNTCACCAATATTATTATCCCTTTGAATTATGAGAATATAAGAATGAATATAAANAAAATATTTNTTAGTTAATCTTTTTTTTTGTCCAGTATTTGTTTTCAATTTNCCATGAAATAGNTAGGATTTTTATCCTTTTCGAAATATTTATATAATTAAATAATTAATGAATAATTTTTGATATGAAATTAGATATTTATAAATAATTCATAGTTTCACGTCACAAATCGTATTATTTNATAATAATTACTTTAGATTAAGATTTANANGNNTGAGTTNGGGACANTNNGAAATNCCTTCNGCCAATNCNCTGNAGCCNTCCTNNCCAATTGGATTNTCAAANCTNTGAGTNATTTAGATAATAATAATGAAAANGAANAAAATATAACAAATTAATGAANGAATGTATAACTTGAGTTGAGGCGGANTANATATTCGTGNACTCTTATAAANGNANAGNTNTGANATTAAAAGTCCAAATCATATNATTTATTTAGAATTAATTACTCGAGATTAAGAGTTAAAGAAGTGAGATTAGCGCATTTNNAAATNCCTTCTNCTAATTAATAATTCATAATAATCACNTTTTCATTATTATAATATTCGGTATGNAATAATATCGAAATGAAANAATGAATATAATTTTGCAAAAATAAATNTAATNAATTAAGAAATTATTTAAAGAATAAATATTAATAAATAATNATAGTTTAAATATAATCATAATTTTAAATACTATAATTAAACGTTAAAGTAGATNACAAATTCAAACTATAAAAATAAATTAATTTAAATTCTATGGTTNCTATATTTNTATTTTGACAATAGATATCCGTCATNTAAAAAAATATATAATAATTTTTATATTTGNTATTTAAATGATTTATGTAAATAAGTTTGCATAAATGATATTTTNATAATTTTAAGAAAGCGTATTGAAGTTAATCTTTCGTTCAGNTTTTCTTTTAATTTATTTTCCGTTTTNAGACCAAAATTATTACTCCTNTGAATTATGAGAATTTGAGAATCGATAAATAAAATAAATTAATGAATGAATTGNATGAAATCGATATTTGTAAATAATGTATAGTTGTTACATCACAAATCATATTATTTTAAAATAAATTACGANAGATCAAGNTTTAAAGNAGTGAGATTGACACATTTTGAAATTCCTTNTCCTAATTCANTGCAACCATTCTCACCAATTTTATTATTCCTTTAAATTATAGAATATGAGAATGTATAAATAAAATAAATTAATAAATAATAATTAATAAAATGANATAATATTTTAAATAATATAAATTTTTTAAATAAAAAATCATATTATTTTNTAATAAATTACTCAAGATNAAGAGTTAAAGAAGTGAGATTGATACATTTNGAAATTTCTTCTCCTAATTTACTGCAACCNTTATCACCAATNTTATTGTTACTTTAAATTATAANAATTAANATAAATAAAATAAA

>T.shanghaiensis_rnd-5_family-657#LRR_CRS3

AAAAAAAAAAAAAAAAAAAAAAAAAAAAAAAAAAAAAAAAAAAAAAAAAAAAAAAAAAAAAAAAAAAAAAAAAAAAAAAAAAAAAAAAAAGAAGTAAAAAATTAAATAATATTACAGCACTCCTCAATAATAAAAAGGAATAAGAATTATATAAAATAAAAATAGATAATTTTATAGANAGAGTTTATTTTGTTGTCATAATTAAATAAATATGTAAACATAAATAGTAATTTAATTATTAGAGTATATTATTAAATAATGTTATTAGATAATTTAATTCGTTCCGAATNATTTTTATCTGACATTNATTGAAAAGAAGGTTTTTAATTATTTTTAATAGGAATTTCGATAATAAGTTTTCAAATTGAAATAGTCTTTTNATTTTTTTGAGTTTTGATTTTGTGATAATTTACAANTTTATTAGAAATTGAATTTTTTCTATATATTATGTGAGNTTANAATNAAAATAAAATAAAGAAGTAATAAATGNAATCTAAAATNTAGTGTTTAATTGTAATNATTAATTATTTAAATTATAATAAATTAAAAATAATTTATAAAAAATTACANTAAATNNAGATTTAAAATAGTGAGATTCGAACATTTAGAAATTGCTTGTCCTAATGAAGCTGCACCATTCTCACCAATGTTATTNTCCCTTCGAATTATTGATATGTTNAAATTAAAAATCCAAATTAANAATNNATAAANTAAATTTAGATAGTCGTAATTTGATTTAGNTTAAATTATTAAAATTTAATAAATTAAAATATTAAATTAATTACTNTAAATTTAGANTTAAAGTAGTGAGATTTAAANATTTAGAGATTGCTTGTNCTAATGAAGCTACACCNTTTTCACCAATATTATTNTNNCTTTGAATTATTTTTATATTAAAATTAAAAATTAAAATAAAGAATTNATNAATTNAATCTAAACAAATTNTGATTTAATTCTAACNATTAATTNTNAAATTGAATAAATTAAAATAATAAATTACCCTAAATAAAGATTTAAACCAGTNAGATTNTAANATTNAGANATTGCTTGTCCTAATGAAGCTGCACCATTCTCACCAAGTTAATTCGNNCTTTAAATTATTTTATTTATTAAAATTTAAAATTAAAATAAAGAATTCATAAATANAATGATAATANATAGTAACGATTTAATTTAATAATTAATTCTAAAATTATTTAATAAATCAAAATATTTAATAATAAATTACCTTAAATAAGANTTAAAGTAGTNAGATTNAAACANTNCGAAATTGCTTGTCCTAAGAAGCTACACCATNTTCACCAANGTNATTANNCCTTTNAATTATATATTAATTTANAAATTAAAATAAAGAATTGATAAATNAAATCCAAATAGAATTTAANTCTAATAATTAATTATTAAAATTAAATAAAAAATAATTAATTATAAATTACCTTAAATCGANATTTAAAGNAGTGAGATTCANACATTTAGAGATTGCTTGTCCTAATGAAGCTACACCNNNATCACCAATTTTATTNTCACTTTNAATTATTNATATATATTAAAATTATAAAACAAAATAAAGAATTAATNAATCAAATCTAAACAAATTATAATTAATTCTAATNATTAATTGTTAAATTNAATAAATNAAAATATTAATAATAAATTACCCTAAACTAAGATTTAAAGTAGTGAGATTNANACATTTCGANATTGCTTGTCCTAATGAAGCTGNACCATTCTCACCGATNTTATTGCAACTTTNAATTATTGATATAATTAAAATAAAAATGAATNAAAATTAAATCAATCTAAATACTTAATTTAATTTTAAATTATTAAGATTTAATAAANTAAAATTATTAATAATAAGTTACCNTAGATCAAGATTTAAAGTAGTGAGGTTAACACATTTCGAAATAGCTTCTCCTAATNTAACTACACCANNCTAACCAATTTTATTCCANCTTTATAATATTTTAATATTCAAATTAAAAGGAAAATTAATAAAATCATCTTATNAAGTAATTATTTAAANAGAATATTATATTAAAATTGAATAAANAAAATAATTAATTANAAATTACNATAAATCTAGANTTAAAGAAGTTAGATTNACACATTTCGAAATTGCTTTTCCTAATTAANTAGCACCATTCTTATCAATTGATTNNCGCTTTTAGTTATTTTGATTTAAATATAATAAATAAAAAATAATTAGTATAAATCCAATATGATTNAAATCTATTTTCAATTATTATTATNATTAATAAANTTAAATAAAAAATATAAATTACTTTAAATTAAGACTTAAAGAAGTGAGATTNACGCATTTNGAGATTGCTTCTCCTAATTTANCTGCACCATTCTCATTAATNTNATTATANCTTTAAATTTTTATTATATTTTAAATTAATTAAAGAAATATTAAATTAAATACAACAAATTAAATTCAATTNATTCTAAATTATAAAATTAAATAAATTANATTAATGATCTTAATTACTTNAAATCAAGATTTAAAGAAGTGAGATTAGTACATTTAGANATTGCCTGCTAATTAATTACACCATTTTCGCCAATTTCATTGACCTTTCAGTTTTTTAATATTATATAAATCAATAAAATTAGATTANATAAAATTATTAAATTATANAATTTAATAAATNAAAATAATTAATATAAATTACNATAAATTAAGANTTAAAGTAGTGAGATTGANACATTTAGATATTGCTTNTNCTAATGAAACTGCGCCTTNCTCACCAATTTTATTGCGACTTTNAAATATTTATNTATTAAAATGAAAAATTAATTAATTATTGTAAATTAAATNTAAGTATTTTGATTAATATAATTATTAAAATTGAATAAATTAAAATAATTANTAATAAATTACCTTAAATCGAGAGTTAAANNAGTGAGATTCACACATTTCGAAATTGCTTNTCCTAATTAAACTGCACCATATCACCAANTTTATTNTAACTTTTATTTTTTNATATTAAATTAAAATTAATAATNAATTAAGANTTTATAAATAAAAATTATTAATNATTAAAATTAATAAATTAAAATATTAATCATAAATTACCTTAAATTNAGANTTAAAGTAGTGAGATTCANACATTTTGAGATTGCTTGTCCTAATGAAGCTGCACCATTATCACTAATGTATTTNTCCTTTTTATATTNTTATATACGATTAAAATAAGNAATTAATNAATTATTAAAANGAGTTAATTCATAACTAAAATAATTAAAATAATAAATTACCCTAAATTTAGTCTTAAAGTAGTGAGATTCGAACATTTTGAGATTGCTTGTCCTAATGAAGCTGCACCATTCTCGCCAATNTCATTTTTCCTTTGAATTATTATTTTATATTAAAATTAAGAATTAAAATAAAGAATTGATTAACGATGATTTTAATATCTANTATTATGAATAAATAAATCAAACTTAACTTATTNAAAATAAATTACCTTAAATCTAAANTTAAACGAGAATTCGTTATATCTATTCTCACCCTTTTNTCCTATTAAAATAAATCATACATAATTATTAACAATCCAAAATNTAATTACCTNANTTATTAGAACTCTTCAAATTAAATTTCCTCCTCAATATTANATTTATCATTCTAGAATATTAATAATTTTTTAATATAATTTANTTTATATAAATTTATATAAAGTAAAAATAATTTAAATTAACTAAAAATTATTTTAATTTTAATAATACTTAAATTCAATAANAGTAAAATATTTAAAAGTTAATATAATTAAACAATTTAAAATAAATAAAAATTATAATTTACATTAATCTTCCATTTTTAAATTATTTCTATTTTTCTCCTCATTTATTCTTTCTCTATCTTTTTTTTTTTAAATCTTTTTTTATATTAAATTAATTATAANAAATCAAAAAGATTTGATAATCTTAAAATATCAACAAAAATAAAATTTNTATTATCTATTTNATATATTTGATAATT

>T.paravorax_rnd-3_family-7#LRR_CRS1

TATTCNTTTTNTTACACAATANATTTTTAATTTAACATCTTTTNTTTTAAATTATAAATAGGGANAATCAATTGGGTGNAGATGGAGCTACTGGCTTAGGAGAGGAGCTTGCAAAATGCTAGAATCTTAATTCTCTCAATTTGAATTTGAGGTAAAANATTTGAATTNTCTCTTCACATTCGTTTATTCATTTTTTTACACAATACATTTTTAATTTGACCATCTTTTGCTTTAAATTATAAATAGCGATAATGAATTGGGTGNAGATGGAGCTATTGGCTTAGGAAAGGAACTTGCAAAATGCTAGAATCTTAATTCTCTCAATTTGGATTTGTGGTAAAACATTTGAGTTTTCTCTTTAGTTTGGGTGAGCTGAAAATATATGTTATCAGTTTACCCATTCGTTTATTCATTTTTTTACACAATACATTCATTTTTAATTTGACCATCTTTTGCTTTAAATTATAAATAGGNATAATGGATTGGGTGCAGATGGAGCTATTGGCTTAGGAAAGGAACTTGCAAAATGCTAGAATCTTAATTCTCTCAATTTGAATTTGNGGTAAAACATTTGAGTTTTCTCTTTAGTTTGGGTGAGCTGAAAATATATGTTATCAGTTTACCCATTCGTTTATTCATTTTTTTACAACAATACATTTTTAATTTGACCATCTTTTCGCTTTAAATTATAAATAGATGAATGAATTGGGTGCAGATGGAGCTATTGGCTTAGGAAAGGNACTTAAAAATTTGAAAAAGTATCAAAAGAGGATTGTTTTTCTTGAAATTNANTTCTGATTCTGCTAAAATAGATAAAATATTATGAAAATATTTAATTAATGTGAATAATTAGATTAATTCAATTCAACGTATCAATTTTTTTATNTTCCAAAATATGTGAACGCCTAATTCTTAAAATGTATATTTAATCAAAGAATAACAAATGTCATAAATATCTATAAATTGAAATACTTTTGAAAGTGAAGAAGTAGAAATTTATCTGTAAAACTAAAAATTTTAGTTTAAATAAAAGANTCATTACTCTATTGGCTACTTAATTTTATNTTAGTTGATAGTTTAGTATTTTATCTAAGTAATCCTANTTAATTTCTATGATACAGCCAAATTCAAAAAAAAAAAAAAAAAAAAAAAAAAAAAAAAAAAAATACATGTATGTATGTTACTTATTGCAGTACAAATANGCAAGAGAGCGGGAACAAGCAAAAAAAAAAACAATTT

>T.paravorax_rnd-4_family-110#LRR_CRS2

AAAAAAAAAAAAAAAAAAAAAAAAAAAAAAAAAAAAAAAAAAAAAAAAAAAAAAAAAAAAAAAAAAAAAAAAAAAAAAAAAAAAAAAAAAAAAAAAAAAAAAAAAAAAAAAAAAAAAAAAAAAAAAAAAAAAAAAAAAAAAAAAAAAAAAAAAAAAAAAAAAAAAAAAAAAAAAACTTGTATATTCCTTTGCTAAACTATCAATAGCTCCATCTCCATCCAATTCATTCCACCTATATAATTCAAAAGAAAATATGTTTGAATTAATCATGTATTCTATAAAAAAAGAATCTATGAACCCTATGAAAGAGTAATTAAAAATATTTTTTTTCAGATAATTTAAAATAAAGAGGTTGTCCAAATCTTTTACGNTANATTCAAANTGAGANAATNTTAAGATTCGNNCANTTTGCAAGNCCCTCTGCTAAGCCGATAGCCCCATCCCGGAGATTCCCANTTCGTTCCCCCTATTTATAGNTTAAAATGAGCTTGAGTTAGAANAATGTTGCGTAATTTTTTAANANCGNATTNTNTAAAAATGAAACTCGAATATTTTACTNCAAACTCAAATTGAGAGAATNAAGATTCTAGTATTTTGCAAGTTCCTNTNNTAAACCANTAGCTCCTTTTTCACCCNATCTGATTACTCCTATTTATAATTTAAAAGAAAAGATTTTAAATTAATAATTTATTAAATAAAAANAGAGTCATTTAACAATAATAGAATANAATGAATATATTATTTATCTTATTCAAAAATAGANACTTTAGATACTTTACCATAAATCCGAATTGANAANATTAAGATTCTAACATTTTACAAGTTCCTTTCCTAAGCCAATAGCTCCATCTNNATTCAACTTATTTTNCCTATTTATAATTTAAAGTAAAAGATGGAAGTTATTGATANTTATTNAAANAAATATGAATTTAGNGAANGAGGCAAAGNTGAACTGGATGANAAACATACTGATTCCCAATCTGTTTTTAATCNAAACAAAGANANTAGGTGTTCAAATGTTTTACNCCAAGTCCAANNCGAGNNAATTNAGATTNNAACATTTTGCNAGTTCCCTTNCTAAGCCAATNGCTCCATCTNNNCCCAATNNACNNNCCCTATTTNTNATNTAAAGGAGTAGATGNTGGNATTGATATTTATTGTATGAAAAACTAATCTATGAATCCTAAATANATGCAGCAAGGATAACTNGATNAAATGTTNTATTTAAAAAAAGANATATTGTTCAAATGATTTACGCTANATTCAAATTGAGAGAATTAAGATTCTAGTATTTTGCAAGTTCTTTTCCTAAGCCAATAGCACCTTCTNCTCTTAATTCATTCCNACTATTTATAATTTAAAGGAAAAAATTATTTTANTAATATTATCGAATNAAATCAATCTATTTTTTAATGAATGAAGAAATTCAGAATNNCTAGATTCANGTGATTCNAATATGAGAGATATTTAATCAAGGGTTTTACCGCAAATTCAANCTGAGAGANTTNAGGTTCTAGNATTTTGCAAGTTCNTTTNCTAAGCCAATAACCCCNCCNGCNCCCAANTNATTTCCACTACTTNTAATTTCAAAGTAAANGATNTTCGTCAAATTAATTNATAATTTGTTGAATAAATAATGAGTCAATGAATCTTAAACGAAGGATAGATTAATGAGAATATTCNTTTGATTTAAAGTAAATTGTTAAATGCTTTACCCCAAATNCAAACTGAGAGAGTTAAATTTTAGCATTTCGCAAGTTCTTTTCCTAAGACCAANNGCNCCGTCTGCATTCAATCNATTNCNGNNANTTATAANTCAAAAGAAAAGAGGTTGATATTAATNTGGGATNNNTGCATAAACTAGCCAAACCATGAATCCATTCAANGAATANTGAGGGAGTTGNAAGAAAATNCGAATGTTNTTACGTCAAATTCAAATCGAGAGAATTAAGATTCTAGCATTTTGCAAGTTCCTTTCCTAAGCCAACAGCTCCATCTNCATCCGATTNATTCCNNCTATTTATAATTTAAATAAAATATGTTGGCATTATTAATGANATGNCGTAAAGAATGAGTCTTTGAAGATTAAATGAATGAGTAGCGTTTTGAAAANNNNNCANGTATTTTNTTCTNGNTCGAGAAANAGGTATTTGTTCAANTAAAAGTTNNACNCCAANCCNAAANTGAGAGAATNAAGATTCTAGCATTTTGCAAGCTCCTTTNCTAAGCCAATAGCCCCNCCTNCANNCAACCNNTTNNNCCTGTTTATTATAATNTAAANNAAGAGGTGTCGGNGTTATTGATTTATTNAATTGGANAATNAATCNATGAATGCTAAATGAGTATGAGGAGCGAGTTGAATGAGAATGTTCATTATAACATGATTTAAAATAATGAGGTTGCTCAATTGTTTCACGNCAAACCCAAATNGAGAGAATTAAGATTCTAGCATTTTGCAANCCCCTCTCCTAAACCAATAGCNCCAGTTTCCCCANTTNATTACAGCTATTNATAATTTAAAAGATAGAATGGAATTAAAATTCATTGGNTNAAAAATGNATNTACGNNTCTTAAAAGAGTGANGANNNAGTTGAGTGAGAGTANTCNGCAATCTGATTCAAAATAAAGAGGTTGGTCAAATATATTTTACCNTAAATTCAAATCGAGAGAATTAAGATTTTAGCATTTTACGAGTTCGTTTCCTAATCCTAGCTCCATCNTCCTCAATNGATTACGCCTATTTATATAAAAATAGATACTGAATTAATATGCNATTCAGGTTGTATAAAAAAAATTATTTGAACNAGGAGTGAATCGAATAATATTTTTTCATCTAATTTAAAAGGAAAAGGTCATTCAAATGTTTTACATTAAATTCAAANTGAGAGAATTAAGATTCTAGCATTTTGCAAGTTCCTTTCCTAAGCCAATAGCTCCATCTNCACCCAATTCATTNTNCCTATTTATAATTTAAAGGAAAAGATGNTCGAATTAATAACGTATTNTGTAAAAAATGAATCTAAACGAATGAGGGTAAACTGATAACATATATTTTCAGCTAATTTAAATAAAGAGAAATACNACAGACTCAAATTGAGAGAATTAAGATTCTAGCATTTTGCAAGTTCCTTTCCTAAACCAATAGCNCCNTCTCACCTAACTNATTACNACTAATTTTAATTTAAAAAGTGAATTAATATTTATTGTATAAAAATGAATCTATGAATCTTAATTAATAAGAGGGAATTTAATAAAAATATTCTGATTCATAAATAAAGAAGTTNTTCAAATGTTTTTCACTCTAAATTCAAATTGAGAGAANTAAGATTCTAGCATTTTNCAAGCCCCTCTCCTAAGCCAATAGCTCCTTCT

>T.paravorax_rnd-4_family-119#LRR_CRS3

ATTCAAAATAACTCATTCTTATTCATTNCTTTTNTTCANACTTCAATCTTAGATTTTAGATATTTATTTACGATAAATCAAGNGTTAATTAGTTGAGACTAACACATTATTTCAATCCATTTCCTATTGCTTCTCCTCCTTTTTCTCCAATTTTATTNTNCCTTAAAATATGAAATTGAATAATATNATTAAAAATATTATTTAAATCGAATTGTTAAATGAAGAGATTGATTGAATGATGACTCAAGATCTATTTAATCTGCAAGATATATCAATTTAAATATTGCTGTATTCACAATCACTCATTCACTCTTTTATTTCGCTTTTCTATCTCATTCTCTAACTAGCTAGTTATNTATTTACNATAAACCAAGTGTTAATGAGTTCAGGCTAACACATTTCTACAGTCCGNTTCCTATTGCTTCTCCTCCTTTTTCTCCAATNTTATTNTACCTTAAAATGTGAAATTGAATAATATTATTAAAAATATGATTAAATCAATTGTTAAATGAAGAATTGATTGAATGATNACTCAAGATCTATTTAATTGCAAAATATATCAATTTAAATATTTATATTCANAATAACTNATTCACTCNTTTATTTCCTTTTCTACTTCAATCTATGATTTTAGATATTTATTTACNATAAATCAAGNGTTAATTAGTTGAGATTAACACATTNTTNCAGTCCATTTCCTATTGCTTCTCCTCCTTTTTCTCCAATTTTATTNTNCCTTAAAATGTAAAATTGAATAATATNATTAAANATATGATTAAANTAAATTGTTAAATGAAGAGATTGATTGAATGATAATTAAAAATTTATTTAATAAGATATTTCAATTTAAATGTTTTATTCAAAATAACTNATTCANTNATTTATTTGCTTTTCGTCTCATTCTATCTTCAGATTTTGAAAATTTTAGAATATTTATTGTACGATAAATTAAATTTTTGAGTTTANATATGACACATTTTTAGAATATAAATAATAATTTCTACCTATAATCCAAANTTAAAGAAGTAACAGACCTAGCTTTTTTTAAATCCATATTCAGAATTTTAAGTCCGTTCTTGATCAGTAATATTTTNGCTTTTTATAAGAATTTGTAAATAAAATAAGTTAATAAAGTTTCANAATATGATATAATTTAATAGAAATAGGTTGAATTTTACTAATTAAATCAATAAGATTGTAATACAATNTTAGTTGAATTATGAAATTATAAATATATATAGAGTTTAGAATGATAATAAAATTCTTTTGATAATCAGTTATTTAATAGAATTTTTCTTTGGTAATAAACTTAATTGTGCATAAATTAATAGCACTAAATATGATTTATACTATTATATACCTGTTATACTAGTTTTNTGAGTTATTTTTGTTAGCGATTTCTTATCCGCTTACTTAAGGATTACCCTTTTTATTTTTACTACGCAACTTCCCATTTCTCTTTTATAATTAATTAATTAATTGTTGANCTTAAATAAGTTGTATAAATNTTACTTGTTTAATGAAATCAAAAATGAATAGAAGTTAATTATTCGAAAAACCTACTCGATTGACTTGTTGCANGAACCATGTATTTNGGNTCTANTCCGATTAAGATCAATTNCTTAGCT

>T.paravorax_rnd-4_family-122#LRR_CRS4

AATGTATGTCAAGGAAAGTTATTCGCCCGTATAAAAACTTTGTTAATTAAAGAAATAAAAAGAATTATTTTTTTAAATATTAATTGACTGAGAATTTGANTGAAATAAAAATTAAGAAGAAATTGAAAAATATAATGCTTTATTGAGCGAGAGCAGGATCTTTGAATGAGAAATCCTTCTTTTTTGAATTGTTTTAATATTNATAAAAGTAAAAGAATATGTATTTTTCTAATTGTATATTTATTTAAATNATTTGCTCTTATACAGTGATTGAAAAAAAAAATTATATTTTTTTTATTTTTGAAAATTGACTAATCAAATTATCATAATTTTATATATTCAGTTAAGTAGAGAATAGTTGAATATACATTAATTAGTTTTTAAGAATAAGTGCTAACTTTTGGATTAAAAAAAAANTTAAATAAATAATTTTTGACTACAATATTAATNTTCTTTATAATTTTGATCAAAATTGGATATTTTCAAAGAGTTTAATTTTTANTGTTAAATATTGCGAACTCTTTGAGATTCAAGTTATTTATTGGTACTCTNTCGCATATTAATCTTCCATATTGATNTTAACCAGTTTTAATAATAGTTTGGCAGCNTATTTCTCTCTGGGGATATTAATTTAAAGATCTNTCATTCACTAGATCAGATTTCCTCAGAAATTTAGCTTGATTAATTACTTCTCTTGAAAACAATGATGTTGAAAAAGTAGAACTTGAAAAATTAGAAAGAAAAATTAAGTAAAAATTAATNAAACCAATTTGCAAAAAAAAAAAATGGAAGAAAAAATGAACTCTAAATATATTAAAGGAACAATAGAAGAAATNAAATCTCTCACAACTCTTAAGTTAGATCTAAAGTACATTTTTAATATTAGGGGTTATTTGTTTAATAATTCTTTATTTTCGCATCTAAATGGCACCAATAAAATATATTNGCTAAAAAGGCAACAATAATCCTANAATTCATTATTTTATATAAATCAGATAAATCCAAACCCTTTAAATGAATTTATTTATGCAGATTAAATCTTTAAATAAATGAATATAAATAGATTTTTANTATTTAATATATTTTAAAAATAGAGANNATAATATTGGAGANAATGGAGCAAGAGATCTAGGATAAGATCTTGNAAANTGTTCTAATTTGAATTCTCTAACCTAGATGTGTATAAGAGACAGGATTAATAGAATAAAATATTTACNNTAAATNTAAGGTTAGAGAGTTCAAATTAGAACACTTTCCAAGACCTTNTCCTAGATCTCTTGCTCCATTCTCTCCAATATTATTNTNNCTATTTTTNAAAATATATTAAATANTGAAAATCTATTTTTANATTAAATATTTNATTCTATTTAAATTGAATATAAAATATATTTACTNTAAATNTAAGGTTAGAGAGTTCAAATTAGAACACTTTCCAAGACCTTNTCCTAGATCTCTTGCTCCATTNTCTCCAATATTATTNNANCTATTTTTTAAAATATATTAAATAAAGAAAATCTATATTTAAAGTAAATATTTTATTCTATTTAAATTGAGTATAAAATATTCTTATACAAATCTAGGTTAGAGAGTTCAAATTAGAACACTTTCCAAGATCTTTTCCTAGATCTCTTGCTCCATTNTCTCCAATNTTATTNTNACTATTTTTTAAAATATATTAAATAANGAAAATCTATATTTANANTAAATATTTTATTCTATTTAAATCGAATTATCAAATATATTTCTTATCAAATNTAGGTTAGAGAGTTCAAATTAGAACACTTTCCAAGATCTTNTCCTAGATCTCTTGCTCCATTGTCTCCAATATTATTNCATCTATTTTTTAAAATATATTAAATAATGAAAAAGTGTTCTAATTTGAACTCTCTAACCTTANATTTAGAGTAAATATTTTATTCTATTTAAATTGAGTTATCAAATGATATTTATGCAAATTAAATGTTTAAATAAAAATAGATTTTCATTATTTAATATATTTTAAAAAATAGTNANAATAATATTGGAGAGAATGGAGCAAGAGATCTAGGANAAGGTCTTGGAAAGTGTTCTAATCTGAACTCTCTAACCTTANATTTANNGTAAATATTTTATTCTATTTNNATTTGAGTTATCAAATGAATTTATTTATGCAAATTAAATGTTTAA

>T.paravorax_rnd-4_family-164#LRR_CRS5

AAAAAAAAAAAAAAAAAAAAAAAAAAAAAAAAAAAAAAAAAAAAAAAAAAAAAAAAAAAAAAAAAAAAAAAAAAAAAAAAAAAAAAAAAAAAAAAAAAAAAAAAAAAAAAAAAAAAAAAAAAAATATGTTTTNTCATCTGNATTNAAANGAAGAAGTTGTTCAAGTGTNTTACTCCAATGTTNAAATTGAGANAATTATAGAAGATTCNGGCATTNTGCNAGCCCCTTTCCTAAGCCAATAGCTCCATCGTCACCCAATTTATTTTTCCTATTTATAATTTCAAAGAAAGGATGTTGGAATTAATAATTTATAGTCTTAAAAATGAATCTATGAATCTTGTGTGAATGAGTGAGTGAGAATATGTTTTAAAAAAAGAAGTTGTTCAAGTGTTTTACTCAAATTCAAATTGAGAGAATTAAGATTCTAGTATTTTGCAAGTTCCTTTCCTAAGCCAATAACTCCATCATCACCCAATTAGTTATACCTATTTATAATTTGAAAGAAAAGATGTTGGAATAAATAATTTATCAGTCTTAAAAATGAATCTATGAATCTTTTATCGAAATTAGATAAAAAGTAAGGAATTCTTTTCTGAATGCATATATTTTATAATATATGCGTGAGTATCTATCCATCTATCTATCTATCCACCCTTCGATCTTATTTTTCCTATTTATAATTTCAAAGAAAGGATATTGGAAAAAAAAAAAAAAAAAAAAAAAAAAAAAAAAAAAAAAAAAAAAAGAATTGAGTGAGAATATGTTTTAAAAAAAGAAGTTGCTCGAGTGCATTTACCTCAAATTCAAATTGAGAGAATTAAGAAACTCTAGTACTTTGCAAGAAAATTTTTTTCCTAAGCCAACAGCTTCATCTCGTCCAATTTTTTATCTATTATAATTTAAAAGAAAAGAGTTGGAGAATNGATAATTTATAGTTTAAAACGAATCTTAAAANTTCGAATGAGGAAGGAATTGAGTGAGAATATGTTTTAAAATAAAGAAGTTGTTAATTTTCAATTAATTTAAATGAAATCAAATATTTTGAAATGNGGATGNTTTTTGTAACGGTATTCTTTTGTATCTTTGATAATGATCCANTAAATATAATATATACCCTTAGTTAATATCCAAAGATGTAATAGATTTGGTTTACTCTTCTGTAATTTGAATATATTATAATTTCATTTTTGAATTATGTATATACTTTTAGATTTTTTTTTNTAGATTGTATTTATAAATTTTCTCAAATATAGAAGAAGGAATATAAAATCACCCTTTTAAATTATTAATATTTCTGAATAAAATTTAATATCTAGTNAGCGATTTTATTTCAGTAANTNTAAATATAAAANGAACATCCANNCTAACTTCTTTTAAAAACTATAATTTATTTTAACTTTAATCTTTNTANTAATGTTAATTAATTAATTAAATTTAAAAACTTGAAGCAAGTTTTGAATAATTCGNATAGATTAGTAAATGTTACTTTGGTGTTGGTAAAAATTTTGAAATATANAT

>T.paravorax_rnd-4_family-282#LRR_CRS6

AACTCAANTTTCATTTNCNAAAANTATTTAATCAATTTTNTNTNTGAAAAATTATAGGAATTTTANAGGACACGATTATTTNAGTTTAATTTNNGGGACGAACCGTGTGTTATTTNTGATNTATANGNTGGACACCCTNATNTTNAATAATCAATACCCTAAATTCAAAGTAAAAAAANTCAACTTCTAATTTAGCGAATTTCATCTAAGTTCTTCCTCTGAATCCTGGTNCAACCCATTTGTTTTATTTAAAATAATATAGTNTCGTTAATAATTTTAAAAATTAAANTCAAATGCTTTATTAGATTATTTTCANAAATAATTAGANTTATTACCCAAAATNCAAAGTAAGAGAACTCAAATTTNAACGTTTGGCAAGTTCTGTCCCTAAATCTCTAGCTCCAATAGTNCCAATNTTATTCGNNCTNATTAAACAATAAANANATTTCATTAAAAGAAAGNAATGCATTTTTAAATCGAATTNTAAATANATTAGCGTTATAATCAATCTTACTATAGATTCAAAGTAAGAGANTTCAGATTTGAGAATTTGGCNAGTTCTACCCCTAAATCTTTAGCTCCAGCNGCACCAATACTATTTATCTAATTAAATAAATAAATNTCATTAATAATNTCATATATGAATATTAANTATTAGATCGTNAGAATCTAAACATTAATTTTCATCCAAATTATTACTATAGTACAAAGNAAGAGAATTCAAATTCGAGCATTTGGCAAGTTCTACCCCTAAATNTCTAGCTCCANCTNAACCNATACTATTNNANCTAATAAAATAATATCAATTACATAACAAATTTATAAAGAAANTAAGAAAATCAAAATGATTAAGATTCTTAAATCTTCANATTATAAACTTAATTAAATTATTACTTTAGATACAAAGTAAGAGAATTCAAATTTTAGCATTTGGCAAGTTCTATCCCTAAATCTCTAGCTCCAACTGCACCAATACTATTTGATCTAAAGAAAANAAAGTTAAATAAATTGCAATGATCTTAGAATTAAAAAGGAATCATTATAATTTAGACATCAGAATCTAANATTAATTCAGTCTTAACTTTTAATNAAAATTATTACTNTAGATNCAAAGTAAGAGAATTCAAATTTGAGCATTTGGCAAGTTCTATCCCTAAATCTCTAGCTCCANCATCNCCAATANNATTCCCNCTAATTAAACAANAATAAATTTCATTAAAAATNAATCATAGAATTAAAAAGAAAATAATTAATTAATTAATTTATTAATTAGTATTAAGNTCAAACTAAATAACAATTATTACCNTAGATNCAAAGTAAGAGAANTCAAATTTGAGGATTTGGNAAGTTCTATCCCTAAATCTCTAGCTCCATCTCGATCAATATTATTACATCTAATTAANAATAATAAATNTCATTANATTTAAAAGAATTCAAATTTATTAAGAATCTAAATATATTCCATTTTCTAAGATATCAAAATTATTACCTTAAATCCAAAGTAAGAGAATTCAAATTTGAACATTTGGCAANTTCTNTTCCTAAATCTCTAGCNCCANGNGCGCCAATANTATTATNNCTAATNAAAAAATTATTAACAAGAATAATTAAAGTAAANCATAGAATAGAAATTTTATCAATCGTTCATTCNAATTANCTTAACTTTAATTTAAATATTTACCNTAGATCCAANNTAAGANAATTCAAATTNGGGNATTTAGNAAATTCTACCCCTAAATCCNTGGCTCCAATCATCCCAATTTTGTTTANCTAATTAAACAATAAATTNGTTAAAAATCATATAATCAAANTTCATAAATTTTTGAATTTAATATCGAAAGACATTTAAAATATATTTCAGTTATTAAAATTATTACCCTAAATTTAAAGTAAGAGANTTCAATTTNGAANATTTGGNAAGTTCTNTCCCTATTGGTCTAGCTCCAACATCGCNAATATTATTNNAACTAATTAAGAAAGAATTATAAACATATTAAAGTCAATCTAAGAATTAAAGANTAAAATCTATCTAACAATTTTCATAGTTNTTAAATTTTTTACTATAAATATAAAGNAAGAGAATTCAAATTTGAACATTTGGCAAGTTATATCCCTAAATNTCTAGCTCCAGCTTCTCCAATACTATTACCACTAATTAAAAAAAATAATACATTCTAATAAATAATTCCAAAATTAAAATTTTCTTGAATTATTAATCTAATATGTTCTAAATCTTCACAGTAAATAAAATTATTACCNTAGATNCAAAGTAAGAGNNTTCAAATTTTAACATTTGGNAAGTTNTATCCCTAAATCTCTAGCTCCAACTNCACNAATANTATTTATCTAATTATTAATTTTAATTTTGAATTATAAAATTTAAGATTTATTCCTTANGATTNTTCATATCATAAATTTTCAATNTCATATTTTCAAAATTATTACCTTAAATCCAAAGTAAGGAAACTCAAGTTTGANNATTTAGCAAGTTCTATCNCTAAATCTCTAGCTCCTGCACCTCCAATACATTATTCCACTAAATTAAATTAATAATAGAATTAAATAAAATATTTAATTTAAATAGTAAAATCAAATTTCTCATTCTCAGTCTTTCAAATAATCANAATTATTACCTTAAATNCAAAGTAAGAGAACTCAAATTTGAGCATTTGGCAAGTTCTATNCCTAANTCTCTAGCTCCAGTTTCNCCAATATTATTATNNCTAATTNAANAATAAATAAGATNTATCAAAACTAATCATATAATTAGAAAATAATTTCTAAATTAATNGNATTATTGNAGTATAAATATTCTAGCTTANTCAGTNATTTCAAGGTATTTTAAATTCAAATAAGTTTTCAAATTTGGACACTTGGCTATTTCTATTCCTAAATCTTTAGCGCCANCTNTACTAATATTANTATNNTTAATAATAAATTTGTNATAATAATAAGAATCCNACAATTAAGAAATATANTTATTTTCAAAATAATGAAAATATTTCTAAAATTAAAATAGAAAATAATTAAAATCATAAGAGCTAATCCCTCATAAAGGCAAAGTAATTAATATCCTCTATTAAAGATTAAAATCTTCTGAAAATTAAAATCCAAGAAGAAAGATATCAATGAACCTGATAAGTTTTATAGGAATTTTATTCCAAAATCCTTAAATCAAAATCAAATAGGAATTTTTCTTA
